# Differential analyses for RNA-seq: transcript-level estimates improve gene-level inferences

**DOI:** 10.12688/f1000research.7563.2

**Published:** 2016-02-29

**Authors:** Charlotte Soneson, Michael I. Love, Mark D. Robinson

**Affiliations:** 1 Institute for Molecular Life Sciences, University of Zurich, Zurich, 8057, Switzerland; 2 SIB Swiss Institute of Bioinformatics, University of Zurich, Zurich, 8057, Switzerland; 3 Department of Biostatistics and Computational Biology, Dana-Farber Cancer Institute, Boston, MA, 02210, USA; 4 Department of Biostatistics, Harvard TH Chan School of Public Health, Boston, MA, 02115, USA

**Keywords:** RNA-seq, quantification, gene expression, transcriptomics

## Abstract

High-throughput sequencing of cDNA (RNA-seq) is used extensively to characterize the transcriptome of cells. Many transcriptomic studies aim at comparing either abundance levels or the transcriptome composition between given conditions, and as a first step, the sequencing reads must be used as the basis for abundance quantification of transcriptomic features of interest, such as genes or transcripts. Various quantification approaches have been proposed, ranging from simple counting of reads that overlap given genomic regions to more complex estimation of underlying transcript abundances. In this paper, we show that gene-level abundance estimates and statistical inference offer advantages over transcript-level analyses, in terms of performance and interpretability. We also illustrate that the presence of differential isoform usage can lead to inflated false discovery rates in differential gene expression analyses on simple count matrices but that this can be addressed by incorporating offsets derived from transcript-level abundance estimates. We also show that the problem is relatively minor in several real data sets. Finally, we provide an R package ( *tximport*) to help users integrate transcript-level abundance estimates from common quantification pipelines into count-based statistical inference engines.

## Introduction

Quantification and comparison of isoform- or gene-level expression based on high throughput sequencing reads from cDNA (RNA-seq) are arguably among the most common tasks in modern computational molecular biology. Currently, one of the most widely used approaches amounts to defining the genomic locations of a set of non-overlapping targets (typically, genes) and using the number of aligned reads overlapping a target as a measure of its abundance, or expression level. Several software packages have been developed for performing such “simple” counting (e.g., *featureCounts* ^1^ and *HTSeq-count* ^2^). More recently, the field has seen a surge in methods aimed at quantifying the abundances of individual *transcripts* (e.g., *Cufflinks* ^3^, *RSEM* ^4^, *BitSeq* ^5^, *kallisto* ^6^ and *Salmon* ^7^). These methods provide higher resolution than simple counting, and by circumventing the computationally costly read alignment step, some (notably, *kallisto* and *Salmon*) are also considerably faster. However, isoform quantification is more complex than the simple counting, due to the high degree of overlap among transcripts. Currently, there is no consensus regarding the optimal resolution or method for quantification and downstream analysis of transcriptomic output.

Another point of debate is the unit in which abundances are given. The traditional R/FPKM ^8, 9^ (reads/fragments per kilobase per million reads) have been largely superseded by the TPM ^10^ (transcripts per million), since the latter is more consistent across libraries. Regardless, all these units attempt to “correct for” sequencing depth and feature length and thus do not reflect the influence of these on quantification uncertainty. In order to account for these aspects, most statistical tools for analysis of RNA-seq data operate instead on the *count* scale. Most of these tools were designed to be applied to simple read counts, and the degree to which their performance is affected by using fractional estimated counts resulting from portioning reads aligning to multiple transcripts is still an open question. The fact that the most common sequencing protocols provide reads that are much shorter than the average transcript implies that the observed read counts depend on a transcript’s length as well as its abundance; thus, simple counts are arguably less accurate measures than TPMs of the true abundance of RNA molecules from given genes. The use of gene counts as input to statistical tools typically assumes that the length of the expressed part of a gene does not change across samples and thus its impact can be ignored for differential analysis.

In the analysis of transcriptomic data, as for any other application, it is of utmost importance that the question of interest is precisely defined before a computational approach is selected. Often, the interest lies in comparing the transcriptional output between different conditions, and most RNA-seq studies can be classified as either: 1) differential gene expression (DGE) studies, where the overall transcriptional output of each gene is compared between conditions; 2) differential transcript/exon usage (DTU/DEU) studies, where the composition of a gene’s isoform abundance spectrum is compared between conditions, or 3) differential transcript expression (DTE) studies, where the interest lies in whether individual transcripts show differential expression between conditions. DTE analysis results can be represented on the individual transcript level, or aggregated to the gene level, e.g., by evaluating whether *at least one* of the isoforms shows evidence of differential abundance.

In this report, we make and give evidence for three claims: 1) gene-level estimation is considerably more accurate than transcript-level; 2) regardless of the level at which abundance estimation is done, *inferences* at the gene level are appealing in terms of robustness, statistical performance and interpretation; 3) taking advantage of transcript-level abundance estimates when defining or analyzing gene-level abundances leads to improved DGE results compared to simple counting for genes exhibiting DTU. The magnitude of the effect in a given data set thus depends on the extent of DTU, and the global impact is relatively small in several real data sets analyzed in this study.

To facilitate a broad range of analysis choices, depending on the biological question of interest, we provide an R/Bioconductor package, *tximport*, to import transcript lengths and abundance estimates from several popular quantification packages and export (estimated) count matrices and, optionally, average transcript length correction terms (i.e., offsets) that can be used as inputs to common statistical engines, such as *DESeq2* ^11^, *edgeR* ^12^ and *limma* ^13^.

## Data and methods

Throughout this manuscript, we utilize two simulated data sets and four experimental data sets (Bottomly ^14^ [ Data set 3], GSE64570 ^15^ [ Data set 4], GSE69244 ^16^ [ Data set 5], GSE72165 ^17^ [ Data set 6], see Supplementary File 1 for further details) for illustration. Details on the data generation and full records of the analyses are provided in the data sets and Supplementary File 1. The first simulated data set (sim1; Data set 1) is the synthetic human data set from Soneson *et al*. ^18^, comprising 20,410 genes and 145,342 transcripts and is available from ArrayExpress (accession E-MTAB-3766). This data set consists of three biological replicates from each of two simulated conditions, and differential isoform usage was introduced for 1,000 genes by swapping the relative expression levels of the two most dominant isoforms between conditions. For each gene in this data set, the total transcriptional output is the same in the two conditions (i.e., no overall DGE); it is worth noting that this is an extreme situation, but provides a useful test set for contrasting DGE, DTU and DTE. The second simulated data set (sim2; Data set 2) is a synthetic data set comprising the 3,858 genes and 15,677 transcripts from the human chromosome 1. It is available from ArrayExpress with accession E-MTAB-4119. Also here, we simulated two conditions with three biological replicates each. For this data set, we simulated both overall DGE, where all transcripts of the affected gene showed the same fold change between the conditions (420 genes), differential transcript usage (DTU), where the total transcriptional output was kept constant but the relative contribution from the transcripts changed (420 genes) and differential transcript expression (DTE), where the expression of 10% of the transcripts of each affected gene was modified (422 genes, 528 transcripts). The three sets of modified genes were disjoint. Again, this synthetic data set represents an extreme situation compared to most real data sets, but provides a useful test case to identify underlying causes of differences between results from various analysis pipelines.

In addition to Data set 1– Data set 6, which contain all code for reproducing our analyses, further method descriptions are given in Supplementary File 1.

Data set 1 http://dx.doi.org/10.5256/f1000research.7563.d114722
Dataset 1 (html) contains all the R code that was used to perform the analyses and generate the figures for the **sim1** data set ^30^. Click here for additional data file. Copyright: © 2016 Soneson C et al. 2016 Data associated with the article are available under the terms of the Creative Commons Zero "No rights reserved" data waiver (CC0 1.0 Public domain dedication).

Data set 2 http://dx.doi.org/10.5256/f1000research.7563.d114723
Data set 2 (html) contains all the R code that was used to perform the analyses and generate the figures for the **sim2** data set ^31^. Click here for additional data file. Copyright: © 2016 Soneson C et al. 2016 Data associated with the article are available under the terms of the Creative Commons Zero "No rights reserved" data waiver (CC0 1.0 Public domain dedication).

Data set 3 http://dx.doi.org/10.5256/f1000research.7563.d114724
Data set 3 (html) contains all the R code that was used to perform the analyses and generate the figures for the **Bottomly** data set ^32^. Click here for additional data file. Copyright: © 2016 Soneson C et al. 2016 Data associated with the article are available under the terms of the Creative Commons Zero "No rights reserved" data waiver (CC0 1.0 Public domain dedication).

Data set 4 http://dx.doi.org/10.5256/f1000research.7563.d114725
Data set 4 (html) contains all the R code that was used to perform the analyses and generate the figures for the **GSE64570** data set ^33^. Click here for additional data file. Copyright: © 2016 Soneson C et al. 2016 Data associated with the article are available under the terms of the Creative Commons Zero "No rights reserved" data waiver (CC0 1.0 Public domain dedication).

Data set 5 http://dx.doi.org/10.5256/f1000research.7563.d114726
Data set 5 (html) contains all the R code that was used to perform the analyses and generate the figures for the **GSE69244** data set ^34^. Click here for additional data file. Copyright: © 2016 Soneson C et al. 2016 Data associated with the article are available under the terms of the Creative Commons Zero "No rights reserved" data waiver (CC0 1.0 Public domain dedication).

Data set 6 http://dx.doi.org/10.5256/f1000research.7563.d114730
Data set 6 (html) contain all the R code that was used to perform the analyses and generate the figures for the **GSE72165** data set ^35^. Click here for additional data file. Copyright: © 2016 Soneson C et al. 2016 Data associated with the article are available under the terms of the Creative Commons Zero "No rights reserved" data waiver (CC0 1.0 Public domain dedication).

## Gene abundance estimates are more accurate than transcript abundance estimates

**Figure 1 (sim2).**
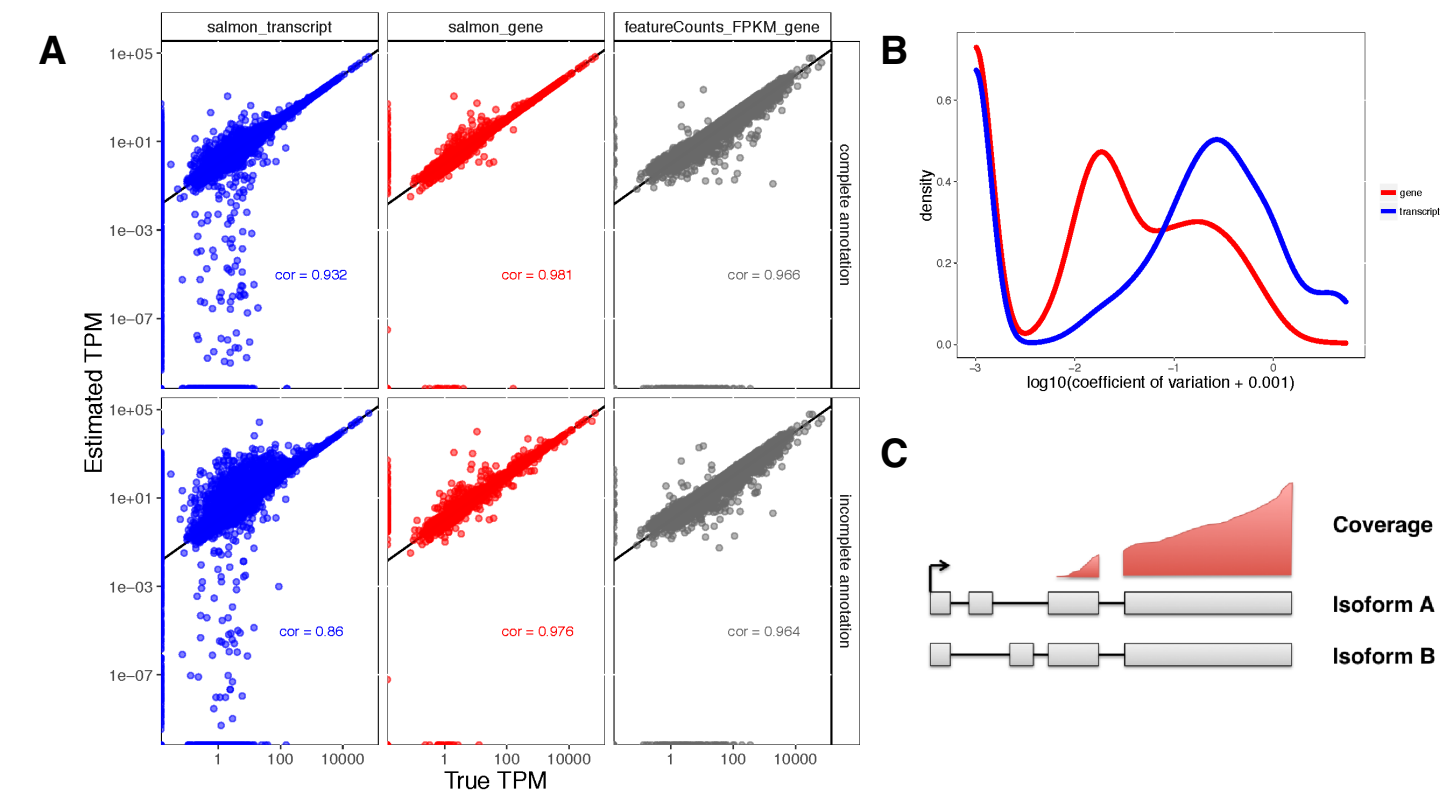
**A**: Accuracy of gene- and transcript-level TPM estimates from *Salmon* and scaled FPKM estimates derived from simple counts from *featureCounts*, in one of the simulated samples (sampleA1). Spearman correlations are indicated in the respective panels. Top row: using the complete annotation. Bottom row: using an incomplete annotation, with 20% of the transcripts randomly removed. Gene-level estimates are more accurate than transcript-level estimates. Gene-level estimates from *Salmon* are more accurate than those from *featureCounts*. **B**: Distribution of the coefficients of variation of gene- and transcript-level abundance estimates from *Salmon*, calculated across 30 bootstrap samples of one of the simulated samples (sampleA1). Gene-level estimates are less variable than transcript-level estimates. **C**: An example of unidentifiable transcript-level estimates, as uneven coverage does not cover the critical regions that would determine the amount that each transcript is expressed, while gene-level estimation is still possible.

To evaluate the accuracy of abundance estimation with transcript and gene resolution, we used the quasi-mapping mode of *Salmon* ^7^ (v0.5.1) to estimate the TPM for each transcript in each of the data sets. Gene-level TPM estimates, representing the overall transcriptional output of each gene, were obtained by summing the corresponding transcript-level TPM estimates. For the two simulated data sets, the true underlying TPM of each feature was known and we could thus evaluate the accuracy of the estimates. Unsurprisingly, gene-level estimates were more accurate than transcript-level estimates ( Figure 1A, Supplementary Figures 4,5). We also derived TPM estimates from simple gene-level counts obtained from traditional alignment of the reads to the genome using STAR followed by counting with *featureCounts*, by dividing the read count for each gene with a reasonable measure of the length of the gene (the length of the union of its exons) and the total number of mapped reads, and scaling the estimates to sum to 1 million. The simple count estimates showed a lower correlation with the true TPMs than the *Salmon* estimates, in line with previous observations ^19^. It is worth noting that we are comparing entire (typical) workflows, and that differences may also occur if the set of reads that STAR is able to align to the genome is not identical to the set of reads that are contributing to the abundance estimation of *Salmon*. However, due to the large fraction of aligned reads and the high mapping rate with *Salmon* (both exceeding 99.8%, more than 95% of the reads were subsequently unambiguously assigned to genes by *featureCounts*), we do not expect this to have a major impact on the results shown in Figure 1A.

Gene-level estimates derived from both simple counts and *Salmon* tended to show a high degree of robustness against incompleteness of the annotation catalog, as evidenced from estimation errors after first removing (at random) 20% of the transcripts ( Figure 1A, see also Supplementary File 1); in contrast, *Salmon*’s transcript estimate accuracies deteriorated. To further compare the merits of genome alignment-based vs alignment-free quantification, especially in their handling of multi-mapping reads, we investigated the accuracy of the abundance estimates within sets of paralogous genes ( Supplementary Figures 1–3). Also here, *Salmon* provided more consistently accurate estimates than STAR+ *featureCounts*. From the bootstrap estimates generated by *Salmon*, we also estimated the coefficient of variation of the abundance estimates. The gene-level estimates showed considerably lower variability than the transcript-level estimates in both simulated and experimental data ( Figure 1B, Supplementary Figures 6,7). Taken together, these observations suggest that the gene-level estimates are more accurate than transcript-level estimates and therefore potentially allow a more accurate and stable statistical analysis. A further argument in favor of gene-level analysis is the unidentifiability of transcript expression that can result from uneven coverage caused by underlying technical biases. While some extent of coverage variability might be alleviated by corrections for sequence- or position-specific biases ^20^, there remain cases where transcript expression cannot be inferred from data ( Figure 1C). Intermediate approaches, grouping together “indistinguishable” features are also conceiveable ^21^, but not yet standard practice.

## DTE is more powerful and easier to interpret on gene level than for individual transcripts

**Figure 2 (sim2).**
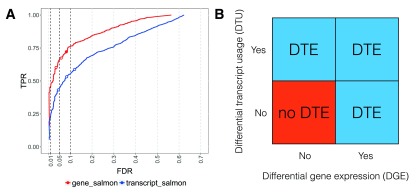
**A**: DTE detection performance on transcript- and gene-level, using *edgeR* applied to transcript-level estimated counts from *Salmon*. The statistical analysis was performed on transcript level and aggregated for each gene using the *perGeneQValue* function from the *DEXSeq* R package; aggregated results show higher detection power. The curves trace out the observed FDR and TPR for each significance cutoff value. The three circles mark the performance at adjusted p-value cutoffs of 0.01, 0.05 and 0.1. **B**: Schematic illustration of different ways in which differential transcript expression (DTE) can arise, in terms of absence or presence of differential gene expression (DGE) and differential transcript usage (DTU).

DTE is concerned with inference of changes in abundance at transcript resolution, and thus invokes a statistical test for each transcript. We argue that this can lead to several complications: the first is conceptual, since the rows (transcripts) in the result table will in many cases not be interpreted independently, since the researcher is often interested in comparing the results for transcripts from the same gene locus, and the second one is more technical, since the number of transcripts is considerably larger than the number of genes, which could lead to lower power due to the portioning of the total set of reads across a larger number of features and a potentially higher multiple testing penalty. We tested for DTE on the simulated data by applying *edgeR* ^12^ to the transcript counts obtained from *Salmon* (the application of count models to *estimated* counts is discussed in the next Section), and represented the results as transcript-level p-values or aggregated these to the gene level by using the *perGeneQValue* function from the *DEXSeq* ^22^ R package. Note that the transcript-level DTE test assesses the null hypothesis that individual transcripts do not change their expression, whereas the gene-level DTE test assesses the null hypothesis that *all* transcripts from a given gene exhibit no change in expression. Framing the DTE question at the gene level results in higher power, without sacrificing false discovery rate control ( Figure 2A). This is not surprising given the different null hypotheses, and, in fact, for many of the genes detected as true positives with the gene-level test, only a subset of the truly changing transcripts were detected ( Supplementary Figure 8). We note that this type of gene-level aggregation may favor genes in which one transcript shows strong changes, and that other approaches to increase power against specific alternatives are conceivable, e.g., capitalizing on the rich collection of methods for gene set analysis.

While DTE analysis is more suitable than DGE analysis for detecting genes with changes in absolute or relative isoform expression but no or only minor change in overall output ( Supplementary Figure 9), we argue that even gene-level DTE results may suffer from lack of interpretability. DTE can manifest in several different ways, as an overall differential expression of the gene or differential relative usage of its transcripts, or a combination of the two ( Figure 2B). We argue that the biological question of interest is in many cases more readily interpretable as a combination of DGE and DTU, rather than DTE. It has been our experience that results reported at the transcript level are still often cast to the gene level (i.e., given a differentially expressed transcript, researchers want to know whether also other isoforms of the gene are changing), suggesting that asking two specific gene-level questions (Is the overall abundance changing? Are the isoform abundances changing proportionally?) trumps the interpretability of one broad question addressing the transcript abundances (Are there changes in any of the isoform expression levels?), despite the increased need for multiple testing correction associated with performing two tests for each gene rather than one. There are of course also situations when a transcript-centric approach provides superior interpretability, for example in targeted experiments where specific isoforms are expected to change due to an administered treatment.

## Incorporating transcript-level estimates leads to more accurate DGE results

DGE (i.e., testing for changes in the overall transcriptional output of a gene) is typically performed by applying a count-based inference method from statistical packages such as *edgeR* ^12^ or *DESeq2* ^11^ to gene counts obtained by read counting software such as *featureCounts* ^1^, *HTSeq-count* ^2^ or functions from the *GenomicAlignments* ^23^ R package. A lot has been written about how simple counting approaches are prone to give erroneous results for genes with changes in relative isoform usage, due to the direct dependence of the observed read count on the transcript length ^24^, and alternatives, such as *Cuffdiff* ^24^, which utilizes estimated transcript abundances, have been proposed. However, the extent of the problem in real data has not been thoroughly investigated. Here, we show that taking advantage of transcript-resolution estimates (e.g., obtained by *Salmon*) in count-based inference methods can lead to improved DGE results. We propose two alternative ways of integrating transcript abundance estimates into the DGE pipeline: to define an “artificial” count matrix, or to calculate offsets that can be used in the statistical modeling of the observed gene counts from, e.g., *featureCounts*. Both approaches are implemented in the accompanying *tximport* R package (available from http://bioconductor.org/packages/tximport).

For the DGE analyses, we defined three different gene-level count matrices for each data set (see also Supplementary File 1): 1) using *featureCounts* from the *Rsubread* ^1^ R package (denoted **featureCounts** below), 2) summing the estimated transcript counts from *Salmon* within genes ( **simplesum**), 3) summing the estimated transcript TPMs from *Salmon* within genes, and multiplying with the total library size in millions ( **scaledTPM**). We note that the scaledTPM values are artificial values, transforming underlying abundance measures to the “count scale” to incorporate the information provided by the sequencing depth. We further used the effective transcript lengths and estimated TPMs from *Salmon* to define average transcript lengths for each gene and each sample (normalization factors) as described in the Supplementary material, to be used as offsets for *edgeR* and *DESeq2* when analyzing the featureCounts and simplesum count matrices ( **featureCounts_avetxl** and **simplesum_avetxl**).

Overall, the counts obtained by all methods were highly correlated ( Supplementary Figures 10–12), which is not surprising since any differences are likely to affect a relatively small subset of the genes. In general, the simplesum and featureCounts matrices led to similar conclusions in all considered data sets, even though there are differences between the two approaches in terms of how multi-mapping reads and reads partly overlapping intronic regions are handled ^25^. Previous studies have also shown that some loss of sensitivity for certain genes may be encountered from discarding multi-mapping fragments, which may be recovered through the use of transcript abundance estimators such as *Salmon* ^21^. The concordance between simplesum and featureCounts results also suggests that statistical methods based on the Negative Binomial assumption are applicable also to summarized, gene-level *estimated* counts, which is further supported by the similarity between the p-value histograms as well as the mean-variance relationships observed with the three types of count matrices ( Supplementary Figures 13–18).

**Figure 3 (sim2).**
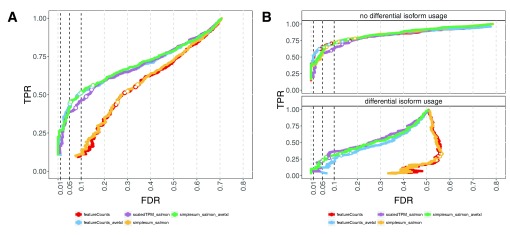
**A**: DGE detection performance of *edgeR* applied to three different count matrices (simplesum, scaledTPM, featureCounts), with or without including an offset representing the average transcript length (for simplesum and featureCounts, avetxl indicates that such offsets were used). Including the offset or using the scaledTPM count matrix leads to improved FDR control compared to using simplesum or featureCounts matrices without offset. The curves trace out the observed FDR and TPR for each significance cutoff value. The three circles mark the performance at adjusted p-value cutoffs of 0.01, 0.05 and 0.1. **B**: stratification of the results in **A** by the presence of differential isoform usage. The improvement in FDR control seen in **A** results from an improved treatment of genes with differential isoform usage, while all methods perform similarly for genes without differential isoform usage.

**Figure 4.**
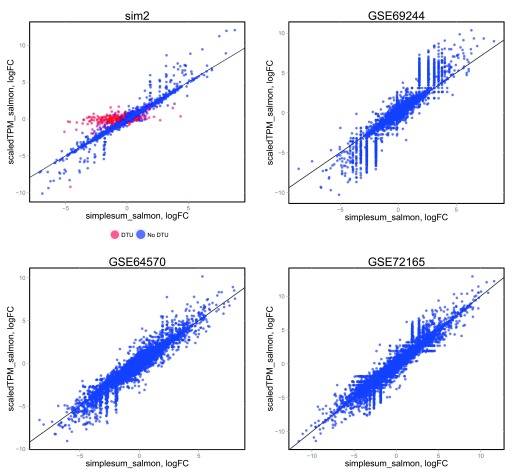
Comparison of log-fold change estimates from *edgeR*, based on **simplesum** and **scaledTPM** count matrices, in four different data sets. For the simulated data set ( **sim2**), where signals have been exaggerated to pinpoint underlying causes of various observations, genes with induced DTU (whose true overall log-fold change is 0) show a clear overestimation of log-fold changes when using **simplesum** counts. However, none of the real data sets contain a similar population of genes, suggesting that for many real data sets, simple gene counting leads to overall similar conclusions as accounting for underlying changes in transcript usage.

Accounting for the potentially varying average transcript length across samples when performing DGE, either in the definition of the count matrix (scaledTPM) or by defining offsets (featureCounts_avetxl, simplesum_avetxl), led to considerably improved false discovery rate (FDR) control compared to using the observed *featureCounts* or aggregated *Salmon* counts directly ( Figure 3A, Table 1). It is important to note that this improvement is entirely attributable to an improved handling of genes with changes in isoform composition between the conditions ( Figure 3B, Supplementary Figure 19), that we purposely introduced strong signals in the simulated data set in order to pinpoint these underlying causes, and that the overall effect in a real data set will depend on the extent to which considerable DTU is present. Experiments on various real data sets ( Supplementary Figure 20) show only small differences in the collections of significant genes found with the simplesum and simplesum_avetxl approaches, suggesting that the extent of the problem in many real data sets is limited, and that most findings obtained with simple counting are not induced by counting artifacts. Further support for this conclusion is shown in Figure 4 (see also Supplementary Figures 21–23 and Supplementary Table 1), where log-fold change estimates from *edgeR*, based on the simplesum and scaledTPM matrices, are contrasted. For the genes with induced DTU in the sim2 data set, log-fold changes based on the simplesum matrix are overestimated, as expected. However, this effect is almost absent in all the real data sets, again highlighting the extreme nature of our simulated data and suggesting that the effect of using different count matrices is considerably smaller for many real data sets. Table 1 further suggests that the lack of error control for simplesum and featureCounts matrices is more pronounced when there is a large difference in length between the differentially used isoforms. In the group with smallest length difference, where the longer differentially used isoform is less than 34% longer than the shorter one, all approaches controlled the type I error satisfactorily. It is worth noting that among all human transcript pairs in which both transcripts belong to the same gene, the median length ratio is 1.85, and for one third of such pairs the longer isoform is less than 38% longer than the shorter one (see Data set 1).

**Table 1 (sim1).** Observed false positive rates from a differential gene expression analysis using *edgeR* applied to various count matrices (with a nominal p-value cutoff at 0.05), limited to genes with true underlying differential isoform usage (recall that no genes are truly differentially expressed in this data set). The results are stratified by “effect size” (the difference in relative abundance between the two differentially used isoforms) and the length ratio between the longer and the shorter of the differentially used isoforms. FPRs below the nominal p-value threshold (0.05) are marked in bold. For more details, see Data set 1.

	simplesum	featureCounts	simplesum_avetxl	featureCounts_avetxl	scaledTPM
[0,0.33), [1,1.34)	**0.019**	**0.019**	**0.023**	**0.023**	**0.023**
[0.33,0.67), [1,1.34)	0.059	0.059	0.059	0.059	0.059
[0.67,1), [1,1.34)	**0.000**	0.053	0.053	0.053	0.053
[0,0.33), [1.34,2.57)	0.075	0.070	0.070	0.065	0.065
[0.33,0.67), [1.34,2.57)	0.240	0.220	**0.050**	**0.033**	0.066
[0.67,1), [1.34,2.57)	0.420	0.540	**0.038**	0.077	**0.038**
[0,0.33), [2.57,35.4]	0.150	0.140	**0.037**	**0.043**	**0.037**
[0.33,0.67), [2.57,35.4]	0.650	0.650	0.060	0.060	**0.034**
[0.67,1), [2.57,35.4]	0.970	0.970	**0.034**	**0.034**	**0.034**

## Discussion

In this article, we have contrasted transcript- and gene-resolution analyses in terms of both abundance estimation and statistical inference, and illustrated that gene-level results are often more accurate, powerful and interpretable than transcript-level results. Not surprisingly, however, accurate transcript-level estimation and inference play an important role in deriving appropriate gene-level results, and it is therefore imperative to continue improving abundance estimation and inference methods applicable to individual transcripts, since misestimation can propagate to the gene level. We have shown that when testing for changes in overall gene expression (DGE), traditional gene counting approaches may lead to an inflated false discovery rate compared to methods aggregating transcript-level TPM values or incorporating correction factors derived from these, for genes where the relative isoform usage differs between the compared conditions. These correction factors can be calculated from the output of transcript abundance programs, using e.g., the provided R package ( *tximport*). It is important to note that the average transcript length offsets must account for the differences in transcript usage between the samples and thus using (sample-independent) exon-union gene lengths will not improve performance.

On the six data sets studied here, simple counting with *featureCounts* led to very similar conclusions as estimated gene counts from *Salmon*, when combined with count-based statistical inference tools such as *edgeR* and *DESeq2*. Moreover, p-value distributions and mean-variance relationships were similar for actual and estimated counts. Taken together, this suggests that the negative binomial assumption made by the count-based tools is flexible enough to accommodate also estimated counts. All evaluated counting approaches, with and without the inclusion of average transcript length offsets, gave comparable DGE results for genes where DTU was not present. Thus, the extent of the FDR inflation in experimental data depends on the extent of DTU between the compared conditions; notably, our simulation introduced rather extreme levels of DTU, hence the inflated FDR, and the difference between the approaches was considerably smaller in real data sets. Recent studies have also shown that many genes express mainly one, dominant isoform ^26^ and for such genes, we expect that simple gene counting will work well.

All evaluations in this study were performed using well-established count-based differential analysis tools. These methods take as input a matrix of counts, which is assumed to correctly represent the origin of each read in a particular set of libraries. However, due to sequence similarities among transcripts or genes, there is often a hidden uncertainty in the feature abundance estimates, even when the set of input reads is fixed. With the development of fast, alignment-free abundance estimation methods, this uncertainty can now be estimated rapidly using bootstrap approaches (see e.g. Figure 1b). Method development is currently underway in the field to account for this uncertainty in the differential expression analysis (e.g., MetaDiff ^27^, sleuth ^6^), which has the potential to improve performance of both DTE and DGE analyses. If such methods are based on (potentially transformed) aggregated transcript counts as gene-level abundance measures, DGE analysis will still be affected by the presence of DTU, and thus could benefit from the inclusion of average transcript length offsets, or by instead using the sum of transcript TPMs as gene abundance measures.

Our results highlight the importance of carefully specifying the question of interest before selecting a statistical approach. Summarization of abundance estimates at the gene level before performing the statistical testing should be the method of choice if the interest is in finding changes in the overall transcriptional output of a gene. However, it is suboptimal if the goal is to identify genes for which *at least one* of the transcripts show differences in transcriptional output, since it may miss genes where two transcripts change in opposite directions, or where a lowly expressed transcript changes. For gene-level detection of DTE (that is, whether any transcript showed a change in expression between the conditions), statistical testing applied to aggregated gene counts led to reduced power and slightly inflated FDR compared to performing the statistical test on the transcript level and aggregating results within genes ( Supplementary Figure 9). Statistical inference on aggregated transcript TPMs (scaledTPM) showed low power for detecting changes that did not affect the overall transcriptional output of the gene, as expected. An alternative to DTE analysis, for potential improved interpretability, is to perform a combination of DGE and DTU analyses, both resulting in gene-level inferences. Table 2 summarizes our results and give suggested workflows for the different types of analyses we have considered.

**Table 2.** Summary of suitable analysis approaches for the three types of comparative analyses discussed in the manuscript (DGE, DTE and DTU).

Task	Input data	Software (examples)	Post-processing
DGE	Aggregated transcript counts + average transcript length offsets, or simple counts + average transcript length offsets	Salmon, kallisto, BitSeq, RSEM	
		tximport	
		DESeq2, edgeR, voom/limma	
DTE	Transcript counts	Salmon, kallisto, BitSeq, RSEM	Optional gene-level aggregation
		tximport	
		DESeq2, edgeR, sleuth, voom/limma	
DTU/DEU	Transcript counts or bin counts, depending on interpretation potential ^18^	Salmon, kallisto, BitSeq, RSEM	Optional gene-level aggregation
		DEXSeq	

Finally, we note that abundance estimation at the gene level can reduce the impact of technical biases on expression levels, which have been shown to lead to estimation errors, such as expression being attributed to the wrong isoform ^28^. Non-uniform coverage from amplification bias or from position bias (3’ coverage bias from poly-(A) selection) can result in unidentifiable transcript-level estimation. While correction of technical artifacts in coverage can be attempted computationally, through estimation of sequence- and position-specific biases ^20^, we note that such errors and estimation problems are also minimized when summarizing expression to the gene level. This being said, there may of course be situations where a direct transcript-level analysis is appropriate. For example, in a cancer setting where a specific deleterious splice variant is of interest (e.g., AR-V7 in prostate cancer ^29^), inferences directly at the transcript level may be preferred. However, while this may be preferred for individual known transcripts, transcriptome-wide differential expression analyses may not be warranted, given the associated multiple testing cost.

## Data availability

The data referenced by this article are under copyright with the following copyright statement: Copyright: © 2016 Soneson C et al.

Data associated with the article are available under the terms of the Creative Commons Zero "No rights reserved" data waiver (CC0 1.0 Public domain dedication). 

*F1000Research*: Dataset 1. Data set 1, 10.5256/f1000research.7563.d114722

*F1000Research*: Dataset 2. Data set 2, 10.5256/f1000research.7563.d114723

*F1000Research*: Dataset 3. Data set 3, 10.5256/f1000research.7563.d114724

*F1000Research*: Dataset 4. Data set 4, 10.5256/f1000research.7563.d114725

*F1000Research*: Dataset 5. Data set 5, 10.5256/f1000research.7563.d114726

*F1000Research*: Dataset 6. Data set 6, 10.5256/f1000research.7563.d114730

## Software availability

### Software access

http://bioconductor.org/packages/tximport

### Source code as at the time of publication

https://github.com/F1000Research/tximport

### Archived source code as at the time of publication

http://dx.doi.org/10.5281/Zenodo.35123

### Software license

*tximport* is released under a GNU Public License (GPL).

## Supplementary material

*Supplementary file 1.*

Supplementary File 1 (pdf) contains more detailed information about the data sets, supplementary methods and supplementary figures referred to in the text.

## References

[1] Liao Y Smyth GK Shi W: featureCounts: an efficient general purpose program for assigning sequence reads to genomic features. *Bioinformatics.* 2014;30(7):923–30. 10.1093/bioinformatics/btt656 24227677

[2] Anders S Pyl PT Huber W: HTSeq--a Python framework to work with high-throughput sequencing data. *Bioinformatics.* 2015;31(2):166–169. 10.1093/bioinformatics/btu638 25260700 PMC4287950

[3] Trapnell C Roberts A Goff L: Differential gene and transcript expression analysis of RNA-seq experiments with TopHat and Cufflinks. *Nat Protoc.* 2012;7(3):562–78. 10.1038/nprot.2012.016 22383036 PMC3334321

[4] Li B Dewey CN: RSEM: accurate transcript quantification from RNA-Seq data with or without a reference genome. *BMC Bioinformatics.* 2011;12:323. 10.1186/1471-2105-12-323 21816040 PMC3163565

[5] Glaus P Honkela A Rattray M: Identifying differentially expressed transcripts from RNA-seq data with biological variation. *Bioinformatics.* 2012;28(13):1721–1728. 10.1093/bioinformatics/bts260 22563066 PMC3381971

[6] Bray N Pimentel H Melsted P: Near-optimal RNA-Seq quantification. *arXiv:1505.02710*. 2015 Reference Source

[7] Patro R Duggal G Kingsford C: Accurate, fast, and model-aware transcript expression quantification with Salmon. *bioRxiv.* 2015 10.1101/021592

[8] Mortazavi A Williams BA McCue K: Mapping and quantifying mammalian transcriptomes by RNA-Seq. *Nat Methods.* 2008;5(7):621–628. 10.1038/nmeth.1226 18516045 PMC13303166

[9] Trapnell C Williams BA Pertea G: Transcript assembly and quantification by RNA-Seq reveals unannotated transcripts and isoform switching during cell differentiation. *Nat Biotechnol.* 2010;28(5):511–515. 10.1038/nbt.1621 20436464 PMC3146043

[10] Wagner GP Kin K Lynch VJ: Measurement of mRNA abundance using RNA-seq data: RPKM measure is inconsistent among samples. *Theory Biosci.* 2012;131(4):281–285. 10.1007/s12064-012-0162-3 22872506

[11] Love MI Huber W Anders S: Moderated estimation of fold change and dispersion for RNA-seq data with DESeq2. *Genome Biol.* 2014;15(12):550. 10.1186/s13059-014-0550-8 25516281 PMC4302049

[12] Robinson MD McCarthy DJ Smyth GK: edgeR: a Bioconductor package for differential expression analysis of digital gene expression data. *Bioinformatics.* 2010;26(1):139–40. 10.1093/bioinformatics/btp616 19910308 PMC2796818

[13] Ritchie ME Phipson B Wu D: *limma* powers differential expression analyses for RNA-sequencing and microarray studies. *Nucleic Acids Res.* 2015;43(7):e47. 10.1093/nar/gkv007 25605792 PMC4402510

[14] Bottomly D Walter NA Hunter JE: Evaluating gene expression in C57BL/6J and DBA/2J mouse striatum using RNA-Seq and microarrays. *PLoS One.* 2011;6(3):e17820. 10.1371/journal.pone.0017820 21455293 PMC3063777

[15] Yang S Marín-Juez R Meijer AH: Common and specific downstream signaling targets controlled by Tlr2 and Tlr5 innate immune signaling in zebrafish. *BMC Genomics.* 2015;16(1):547. 10.1186/s12864-015-1740-9 26208853 PMC4514945

[16] Currais A Goldberg J Farrokhi C: A comprehensive multiomics approach toward understanding the relationship between aging and dementia. *Aging (Albany NY).* 2015;7(11):937–955. 26564964 10.18632/aging.100838 PMC4694064

[17] Chang AJ Ortega FE Riegler J: Oxygen regulation of breathing through an olfactory receptor activated by lactate. *Nature.* 2015;527(7577):240–244. 10.1038/nature15721 26560302 PMC4765808

[18] Soneson C Matthes KL Nowicka M: Differential transcript usage from RNA-seq data: isoform pre-filtering improves performance of count-based methods. *bioRxiv.* 2015 10.1101/025387 PMC4729156 26813113

[19] Kanitz A Gypas F Gruber AJ: Comparative assessment of methods for the computational inference of transcript isoform abundance from RNA-seq data. *Genome Biol.* 2015;16(1):150. 10.1186/s13059-015-0702-5 26201343 PMC4511015

[20] Roberts A Trapnell C Donaghey J: Improving RNA-Seq expression estimates by correcting for fragment bias. *Genome Biol.* 2011;12(3):R22. 10.1186/gb-2011-12-3-r22 21410973 PMC3129672

[21] Robert C Watson M: Errors in RNA-Seq quantification affect genes of relevance to human disease. *Genome Biol.* 2015;16(1):177. 10.1186/s13059-015-0734-x 26335491 PMC4558956

[22] Anders S Reyes A Huber W: Detecting differential usage of exons from RNA-seq data. *Genome Res.* 2012;22(10):2008–17. 10.1101/gr.133744.111 22722343 PMC3460195

[23] Lawrence M Huber W Pagès H: Software for computing and annotating genomic ranges. *PLoS Comput Biol.* 2013;9(8):e1003118. 10.1371/journal.pcbi.1003118 23950696 PMC3738458

[24] Trapnell C Hendrickson DG Sauvageau M: Differential analysis of gene regulation at transcript resolution with RNA-seq. *Nat Biotechnol.* 2013;31(1):46–53. 10.1038/nbt.2450 23222703 PMC3869392

[25] Zhao S Xi L Zhang B: Union Exon Based Approach for RNA-Seq Gene Quantification: To Be or Not to Be? *PLoS One.* 2015;10(11):e0141910. 10.1371/journal.pone.0141910 26559532 PMC4641603

[26] Gonzàlez-Porta M Frankish A Rung J: Transcriptome analysis of human tissues and cell lines reveals one dominant transcript per gene. *Genome Biol.* 2013;14(7):R70. 10.1186/gb-2013-14-7-r70 23815980 PMC4053754

[27] Jia C Guan W Yang A: MetaDiff: differential isoform expression analysis using random-effects meta-regression. *BMC Bioinformatics.* 2015;16(1):208. 10.1186/s12859-015-0623-z 26134005 PMC4489045

[28] Love MI Hogenesch JB Irizarry RA: Modeling of RNA-seq fragment sequence bias reduces systematic errors in transcript abundance estimation. *bioRxiv.* 2015 10.1101/025767 PMC5143225 27669167

[29] Antonarakis ES Lu C Wang H: AR-V7 and resistance to enzalutamide and abiraterone in prostate cancer. *N Engl J Med.* 2014;371(11):1028–38. 10.1056/NEJMoa1315815 25184630 PMC4201502

[30] Soneson C Love MI Robinson MD: Data set 1 in: Differential analyses for RNA-seq: transcript-level estimates improve gene-level inferences. *F1000Research.* 2016 Data Source 10.12688/f1000research.7563.1 PMC4712774 26925227

[31] Soneson C Love MI Robinson MD: Data set 2 in: Differential analyses for RNA-seq: transcript-level estimates improve gene-level inferences. *F1000Research.* 2016 Data Source 10.12688/f1000research.7563.1 PMC4712774 26925227

[32] Soneson C Love MI Robinson MD: Data set 3 in: Differential analyses for RNA-seq: transcript-level estimates improve gene-level inferences. *F1000Research.* 2016 Data Source 10.12688/f1000research.7563.1 PMC4712774 26925227

[33] Soneson C Love MI Robinson MD: Data set 4 in: Differential analyses for RNA-seq: transcript-level estimates improve gene-level inferences. *F1000Research.* 2016 Data Source 10.12688/f1000research.7563.1 PMC4712774 26925227

[34] Soneson C Love MI Robinson MD: Data set 5 in: Differential analyses for RNA-seq: transcript-level estimates improve gene-level inferences. *F1000Research.* 2016 Data Source 10.12688/f1000research.7563.1 PMC4712774 26925227

[35] Soneson C Love MI Robinson MD: Data set 6 in: Differential analyses for RNA-seq: transcript-level estimates improve gene-level inferences. *F1000Research.* 2016 Data Source 10.12688/f1000research.7563.1 PMC4712774 26925227

