# Supplementary material for: Differential analyses for RNA-seq: transcript-level estimates improve gene-level inferences
Source: F1000Res. 2016 Feb 29;4:1521. Originally published 2015 Dec 30. [Version 2] doi: 10.12688/f1000research.7563.2 (PMC4712774; doi:10.12688/f1000research.7563.2)
Supplement: Data set 1 — http://dx.doi.org/10.5256/f1000research.7563.d114722 Dataset 1 (html) contains all the R code that was used to perform the analyses and generate the figures for the sim1 data set 30. [file f1000research-4-8770-s0000.tgz › 4cc0b0d8-4853-4896-b66e-a4715dd3bf7b_sim1-quantification.html]

sim1


# sim1

- Preparation
  - Reference directories and packages
  - Metadata definition
  - Definition of paths to reference files
  - Definition of paths to generated files
  - Salmon index generation and abundance estimation
  - featureCounts read counting
  - Definition of gene-to-transcript mapping
  - Calculation of differences in size between differentially used isoforms
    - For comparison, calculate the distribution of the “lengthratio” score for all transcript pairs from the same gene
  - Summarization of Salmon results and offset estimation
    - Estimation of coefficients of variation for all samples
- Accuracy of abundance estimates
  - All features
  - Only features with positive true and estimated TPMs
  - Consider groups of paralogous genes only
- Variability of transcript and gene TPMs
- Correlation between gene count estimates
  - All genes
  - Only genes with positive estimates with all methods
  - Example of genes with a big difference between simple sum and scaled TPM:
- Differential transcript expression (DTE) analysis on gene and transcript level
  - Comparison of DTE performance on gene and transcript level
- Differential gene expression analysis (edgeR)
  - Diagnostics
  - Comparison of significant genes found with different matrices
  - FPR calculation
- Differential gene expression analysis (DESeq2)
  - Diagnostics
  - Comparison of significant genes found with different matrices
  - FPR calculation
- Help functions
- Session info

The sim1 data set consists of synthetic human, paired-end, 100bp reads from two conditions, each with three samples. The basis for the simulation is Ensembl GRCh37.71. We introduce differential isoform usage for 1,000 genes, as described by Soneson, Matthes et al.

## Preparation

(Back to top)

### Reference directories and packages

(Back to top)

```
basedir <- "/home/charlotte/gene_vs_tx_quantification"
suppressPackageStartupMessages(library(biomaRt))
suppressPackageStartupMessages(library(dplyr))
suppressPackageStartupMessages(library(tximport))
suppressPackageStartupMessages(library(Rsubread))
suppressPackageStartupMessages(library(ggplot2, lib.loc = "/home/charlotte/R/x86_64-pc-linux-gnu-library/3.2"))
suppressPackageStartupMessages(library(iCOBRA))
suppressPackageStartupMessages(library(reshape2))
suppressPackageStartupMessages(library(Hmisc))
suppressPackageStartupMessages(library(DESeq2, lib.loc = "/home/charlotte/R/x86_64-pc-linux-gnu-library/3.2"))
```

```
## Warning: replacing previous import by 'ggplot2::Position' when loading 'DESeq2'
```

```
sumna <- function(w) {
  if (all(is.na(w))) NA
  else sum(w, na.rm = TRUE)
}

muted <- c("#DC050C","#E8601C","#7BAFDE","#1965B0","#B17BA6",
           "#882E72","#F1932D","#F6C141","#F7EE55","#4EB265",
           "#90C987","#CAEDAB","#777777")
```

### Metadata definition

(Back to top)

```
meta <- data.frame(sample = paste0("sample", 1:6),
                   condition = c("A", "A", "A", "B", "B", "B"),
                   stringsAsFactors = FALSE)
rownames(meta) <- meta$sample
meta
```

```
##          sample condition
## sample1 sample1         A
## sample2 sample2         A
## sample3 sample3         A
## sample4 sample4         B
## sample5 sample5         B
## sample6 sample6         B
```

### Definition of paths to reference files

(Back to top)

The following reference files (either pointing to annotation files downloaded from Ensembl or corresponding to files generated by the simulation/pre-processing steps) will be used throughout the analysis.

```
simdir <- "/home/Shared/data/alt_splicing_simulations/Simulation5_Charlotte/hsapiens"
gtf <- paste0(simdir, "/reference_files/Homo_sapiens.GRCh37.71.primary_assembly.protein_coding.gtf")
cdna_fasta <- paste0("/home/Shared/data/alt_splicing_simulations/dte_simulation",
                     "/annotations/wholegenome/Homo_sapiens.GRCh37.71.cdna.all.fa")
truth_tx_sample1_file <- paste0(simdir, "/no_diffexpression/non_null_simulation/1_reads/rsem_files/sample1.txt")
fq_basedir <- paste0(simdir, "/no_diffexpression/non_null_simulation/1_reads/reads")
bam_basedir <- paste0(simdir, "/no_diffexpression/non_null_simulation/1_reads/tophat")
truth_gene_file <- paste0(simdir, "/no_diffexpression/non_null_simulation/3_truth/truth_human_non_null.txt")
truth_tx_file <- paste0(simdir, "/no_diffexpression/non_null_simulation/3_truth/simulation_details_isopct2.txt")
```

### Definition of paths to generated files

(Back to top)

The following files will be generated by the code below.

```
salmon_index <- paste0(basedir, "/annotation/Homo_Sapiens_GRCh37.71/salmon_index/",
                       "Homo_sapiens.GRCh37.71.cdna.all.fa.sidx")
salmon_basedir <- paste0(basedir, "/quantifications/sim1/salmon")
feature_lengths_file <- paste0(basedir, "/annotation/Homo_Sapiens_GRCh37.71/feature_lengths.Rdata")
tx_gene_file <- paste0(basedir, "/annotation/Homo_Sapiens_GRCh37.71/tx_gene_map.Rdata")
cv_salmon_file <- paste0(basedir, "/quantifications/sim1/salmon/cv_salmon.Rdata")
fc_file <- paste0(basedir, "/quantifications/sim1/featureCounts/fc_sim1.Rdata")
isoform_diff_file <- "isoform_differences.Rdata"
```

### Salmon index generation and abundance estimation

(Back to top)

```
cmd <- paste("salmon index -i", salmon_index, "-t", cdna_fasta, "-p 5 --type quasi")
message(cmd)
```

```
## salmon index -i /home/charlotte/gene_vs_tx_quantification/annotation/Homo_Sapiens_GRCh37.71/salmon_index/Homo_sapiens.GRCh37.71.cdna.all.fa.sidx -t /home/Shared/data/alt_splicing_simulations/dte_simulation/annotations/wholegenome/Homo_sapiens.GRCh37.71.cdna.all.fa -p 5 --type quasi
```

```
if (!file.exists(paste0(salmon_index, "/hash.bin"))) {
  system(cmd)
} else {
  message("Salmon index already exists.")
}
```

```
## Salmon index already exists.
```

```
fq_dirs <- list.files(fq_basedir, full.names = TRUE)
fq_dirs <- fq_dirs[file.info(fq_dirs)$isdir]
names(fq_dirs) <- basename(fq_dirs)

for (i in 1:length(fq_dirs)) {
  if (!file.exists(paste0(salmon_basedir, "/", names(fq_dirs)[i], "/quant.sf"))) {
    cmd <- sprintf("salmon quant -i %s -l IU -1 %s -2 %s -p 5 -o %s --numBootstraps=30",
                   salmon_index,
                   paste0("<(gunzip -c ", fq_dirs[i], "/", 
                          gsub("sample", "sample_", names(fq_dirs)[i]), "_1.fq.gz)"),
                   paste0("<(gunzip -c ", fq_dirs[i], "/", 
                          gsub("sample", "sample_", names(fq_dirs)[i]), "_2.fq.gz)"),
                   paste0(salmon_basedir, "/", names(fq_dirs)[i]))
    cat(cmd, "\n")
    system(cmd)
  } else {
    cat("Salmon results for", names(fq_dirs)[i], "already exist.\n")
  }
}
```

```
## Salmon results for sample1 already exist.
## Salmon results for sample2 already exist.
## Salmon results for sample3 already exist.
## Salmon results for sample4 already exist.
## Salmon results for sample5 already exist.
## Salmon results for sample6 already exist.
```

### featureCounts read counting

(Back to top)

```
bamdir <- list.files(bam_basedir, pattern = "sample[0-9]$", full.names = TRUE)
bamdir <- bamdir[file.info(bamdir)$isdir]
bamfiles <- paste0(bamdir, "/accepted_hits.bam")
names(bamfiles) <- basename(bamdir)

if (!file.exists(fc_file)) {
  fc_sim1 <- featureCounts(
    files = bamfiles,
    annot.ext = gtf,
    isGTFAnnotationFile = TRUE,
    GTF.featureType = "exon",
    GTF.attrType = "gene_id",
    useMetaFeatures = TRUE,
    isPairedEnd = TRUE, nthreads = 5, 
    strandSpecific = 0, countMultiMappingReads = FALSE
  )
  colnames(fc_sim1$counts) <- sapply(colnames(fc_sim1$counts), function(w) {
    s <- strsplit(w, "\\.")[[1]]
    s[grep("sample", s)]
  })
  colnames(fc_sim1$stat) <- sapply(colnames(fc_sim1$stat), function(w) {
    s <- strsplit(w, "\\.")[[1]]
    s[grep("sample", s)]
  })
  save(fc_sim1, file = fc_file)
} else {
  message("Loading previously saved file with featureCounts quantifications.")
  load(fc_file)
}
```

```
## Loading previously saved file with featureCounts quantifications.
```

### Definition of gene-to-transcript mapping

(Back to top)

Here we calculate the lengths of all genes and transcript (both defined here by the union of their exons), and define a mapping between gene and transcript identifiers from the reference files.

```
if (!file.exists(feature_lengths_file)) {
  calc_lengths_mapping(gtf = gtf, cdna_fasta = cdna_fasta, 
                       feature_lengths_file = feature_lengths_file, 
                       tx_gene_file = tx_gene_file) 
} else {
  message("feature lengths and tx-to-gene map already calculated.")
}
```

```
## feature lengths and tx-to-gene map already calculated.
```

```
load(feature_lengths_file)
load(tx_gene_file)
```

### Calculation of differences in size between differentially used isoforms

(Back to top)

```
## Calculate the number of basepairs by which the two most abundant
## isoforms differ. 
if (!file.exists(isoform_diff_file)) {
  suppressPackageStartupMessages(library(GenomicFeatures))
  final_summary <- 
    read.delim(paste0(simdir,
                      "/no_diffexpression/non_null_simulation/3_truth/simulation_details_isopct2.txt"),
               header = TRUE, as.is = TRUE)
  tmp <- subset(final_summary, transcript_ds_status == 1) %>% 
    group_by(gene_id) %>%
    summarise(topisoform = transcript_id[which.max(IsoPct)],
              secisoform = ifelse(length(transcript_id) > 1, 
                                  transcript_id[order(IsoPct, 
                                                      decreasing = TRUE)[2]], 
                                  "NA"))
  txdb <- makeTxDbFromGFF(gtf, format = "gtf")
  ebt <- exonsBy(txdb, "tx", use.names = TRUE)
  ebt2 <- ebt[setdiff(c(tmp$topisoform, tmp$secisoform), "NA")]
  xx <- t(sapply(1:nrow(tmp), function(i) {
    if (any(tmp[i, ] == "NA")) 0
    else {
      a <- ebt2[[unlist(tmp[i, "topisoform"])]]
      b <- ebt2[[unlist(tmp[i, "secisoform"])]]
      c(nbrbp = sum(width(GenomicRanges::union(a, b))) - 
          sum(width(GenomicRanges::intersect(a, b))),
        jaccard = (sum(width(GenomicRanges::union(a, b))) - 
                     sum(width(GenomicRanges::intersect(a, b))))/sum(width(GenomicRanges::union(a, b))),
        lengthdiff = abs(sum(width(a)) - sum(width(b))),
        rellengthdiff = (abs(sum(width(a)) - sum(width(b))))/(sum(width(a)) + sum(width(b))),
        lengthratio = max(sum(width(a)), sum(width(b)))/min(sum(width(a)), sum(width(b))))
    }
  }))
  tmp$nbrbp <- xx[, "nbrbp"]
  tmp$jaccard <- xx[, "jaccard"]
  tmp$lengthdiff <- xx[, "lengthdiff"]
  tmp$rellengthdiff <- xx[, "rellengthdiff"]
  tmp$lengthratio <- xx[, "lengthratio"]
  isoform_differences <- as.data.frame(tmp)
  save(isoform_differences, file = isoform_diff_file)
} else {
  message(isoform_diff_file, " already exists. ")
}
```

```
## isoform_differences.Rdata already exists.
```

```
load(isoform_diff_file)
```

#### For comparison, calculate the distribution of the “lengthratio” score for all transcript pairs from the same gene

```
if (!file.exists("isoform_lengthratio_alltxpairs.RData")) {
  txdb <- GenomicFeatures::makeTxDbFromGFF(gtf)
  g <- keys(txdb, "GENEID")
  df <- select(txdb, keys = g, keytype = "GENEID", columns = "TXID")
  ebt <- exonsBy(txdb, by = "tx")
  res <- lapply(g, function(gene) {
    txs <- df$TXID[df$GENEID == gene]
    if (length(txs) == 1) return(NA)
    lengths <- sum(width(ebt[txs]))
    idxs <- combn(lengths, 2)
    2^abs(log2(idxs[1, ]/idxs[2, ]))
  })
  isoform_lengthratio_alltxpairs <- unlist(res)
  save(isoform_lengthratio_alltxpairs, file = "isoform_lengthratio_alltxpairs.RData")
} else {
  load("isoform_lengthratio_alltxpairs.RData")
}
summary(isoform_lengthratio_alltxpairs, na.rm = TRUE)
```

```
##     Min.  1st Qu.   Median     Mean  3rd Qu.     Max.     NA's 
##    1.000    1.235    1.847    3.101    3.694 2414.000     3556
```

```
round(quantile(isoform_lengthratio_alltxpairs, probs = c(0, 1/3, 2/3, 1), na.rm = TRUE), digits = 3)
```

```
##        0% 33.33333% 66.66667%      100% 
##     1.000     1.378     2.880  2413.667
```

### Summarization of Salmon results and offset estimation

(Back to top)

We use the `tximport` package (https://github.com/mikelove/tximport) to generate count matrices and offset matrices (average transcript lengths) from the Salmon transcript-level estimates. We generate two different count matrices (**simplesum** and **scaledTPM**), and additionally create offsets to be used with the **simplesum** matrix.

```
salmon_files <- list.files(salmon_basedir, pattern = "sample", full.names = TRUE)
salmon_files <- salmon_files[file.info(salmon_files)$isdir]
salmon_files <- paste0(salmon_files, "/quant.sf")
salmon_files <- salmon_files[file.exists(salmon_files)]
names(salmon_files) <- basename(gsub("/quant.sf", "", salmon_files))
txi_salmonsimplesum <- tximport(files = salmon_files, type = "salmon", txIn = TRUE,
                                txOut = FALSE, countsFromAbundance = "no", 
                                gene2tx = gene2tx)
```

```
## reading in files
```

```
## 1
```

```
## 2
```

```
## 3
```

```
## 4
```

```
## 5
```

```
## 6
```

```
##
```

```
## summarizing abundance
```

```
## summarizing counts
```

```
## summarizing length
```

```
txi_salmonscaledtpm <- tximport(files = salmon_files, type = "salmon", txIn = TRUE,
                                txOut = FALSE, countsFromAbundance = "scaledTPM", 
                                gene2tx = gene2tx)
```

```
## reading in files
```

```
## 1
```

```
## 2
```

```
## 3
```

```
## 4
```

```
## 5
```

```
## 6
```

```
##
```

```
## summarizing abundance
```

```
## summarizing counts
```

```
## summarizing length
```

```
txi_salmontx <- tximport(files = salmon_files, type = "salmon", txIn = TRUE,
                         txOut = TRUE, countsFromAbundance = "no", gene2tx = gene2tx)
```

```
## reading in files
```

```
## 1
```

```
## 2
```

```
## 3
```

```
## 4
```

```
## 5
```

```
## 6
```

```
##
```

```
salmon_quant <- list(geneCOUNT_sal_simplesum = txi_salmonsimplesum$counts,
                     geneCOUNT_sal_scaledTPM = txi_salmonscaledtpm$counts,
                     avetxlength = txi_salmonsimplesum$length,
                     txTPM_sal = txi_salmontx$abundance,
                     txCOUNT_sal = txi_salmontx$counts,
                     txi_salmonsimplesum = txi_salmonsimplesum,
                     txi_salmonscaledtpm = txi_salmonscaledtpm, 
                     txi_salmontx = txi_salmontx)
```

#### Estimation of coefficients of variation for all samples

(Back to top)

```
## Transcript
salmon_dirs <- list.files(salmon_basedir, pattern = "sample", full.names = TRUE)
salmon_dirs <- salmon_dirs[file.info(salmon_dirs)$isdir]
names(salmon_dirs) <- basename(salmon_dirs)

if (!file.exists(cv_salmon_file)) {
  CV_tx_salmon <- as.data.frame(sapply(salmon_dirs, function(w) {
    tmpdf <- as.data.frame(t(read.delim(paste0(w, "/quant_bootstraps.sf"), 
                                        skip = 11, header = TRUE, as.is = TRUE)))
    tmpcv <- apply(tmpdf, 1, sd)/apply(tmpdf, 1, mean)
    tmpcv[is.na(tmpcv)] <- 0
    names(tmpcv) <- rownames(tmpdf)
    tmpcv
  }))
  CV_tx_salmon$mean_cv <- apply(CV_tx_salmon[, names(salmon_dirs)], 1, mean)
  CV_tx_salmon$min_cv <- apply(CV_tx_salmon[, names(salmon_dirs)], 1, min)
  CV_tx_salmon$max_cv <- apply(CV_tx_salmon[, names(salmon_dirs)], 1, max)
  CV_tx_salmon$median_cv <- apply(CV_tx_salmon[, names(salmon_dirs)], 1, median)
  
  ## Gene
  CV_gene_salmon <- as.data.frame(sapply(salmon_dirs, function(w) {
    tmpdf <- as.data.frame(t(read.delim(paste0(w, "/quant_bootstraps.sf"), 
                                        skip = 11, header = TRUE, as.is = TRUE)))
    tmpdf$gene <- tx2gene$gene[match(rownames(tmpdf), tx2gene$transcript)]
    tmpdf <- tmpdf %>% group_by(gene) %>% summarise_each(funs(sum)) %>% as.data.frame
    rownames(tmpdf) <- tmpdf$gene
    tmpdf$gene <- NULL
    tmpcv <- apply(tmpdf, 1, sd)/apply(tmpdf, 1, mean)
    tmpcv[is.na(tmpcv)] <- 0
    names(tmpcv) <- rownames(tmpdf)
    tmpcv
  }))
  CV_gene_salmon$mean_cv <- apply(CV_gene_salmon[, names(salmon_dirs)], 1, mean)
  CV_gene_salmon$min_cv <- apply(CV_gene_salmon[, names(salmon_dirs)], 1, min)
  CV_gene_salmon$max_cv <- apply(CV_gene_salmon[, names(salmon_dirs)], 1, max)
  CV_gene_salmon$median_cv <- apply(CV_gene_salmon[, names(salmon_dirs)], 1, median)
  
  save(CV_gene_salmon, CV_tx_salmon, file = cv_salmon_file)
} else {
  message("Salmon CVs already exist")
}
```

```
## Salmon CVs already exist
```

```
load(cv_salmon_file)
```

## Accuracy of abundance estimates

(Back to top)

The accuracy of the abundance estimates from Salmon and featureCounts is evaluated by comparing to the true TPMs underlying the simulation.

### All features

(Back to top)

```
## Complete annotation
truth_tx_sample1 <- read.delim(truth_tx_sample1_file, header = TRUE, as.is = TRUE)

sample1_tx_salmon <- Reduce(function(...) merge(..., by = "feature", all = TRUE), 
                            list(data.frame(feature = truth_tx_sample1$transcript_id,
                                            true_tpm = truth_tx_sample1$TPM, 
                                            gene = truth_tx_sample1$gene_id, 
                                            stringsAsFactors = FALSE),
                                 data.frame(feature = rownames(salmon_quant$txTPM_sal),
                                            est_tpm = salmon_quant$txTPM_sal[, "sample1"],
                                            stringsAsFactors = FALSE)))
sample1_tx_salmon$measure <- "salmon_transcript"

sample1_gene_salmon <- sample1_tx_salmon %>% group_by(gene) %>% 
  summarise_each(funs(sumna), -c(feature, measure)) %>% as.data.frame
sample1_gene_salmon$measure <- "salmon_gene"

load(fc_file)
sample1_gene_fc <- Reduce(function(...) merge(..., by = "gene", all = TRUE), 
                          list(sample1_gene_salmon[, c("gene", "true_tpm")], 
                               data.frame(gene = rownames(fc_sim1$counts),
                                          fc_count = fc_sim1$counts[, "sample1"],
                                          stringsAsFactors = FALSE),
                               data.frame(gene = names(gene_length),
                                          gene_length_unionexon = gene_length,
                                          stringsAsFactors = FALSE)))
sample1_gene_fc$fpkm_fc_unionexon <- 
  sample1_gene_fc$fc_count/(sample1_gene_fc$gene_length_unionexon/1e3 *
                              sum(sample1_gene_fc$fc_count, na.rm = TRUE)/1e6)
sample1_gene_fc$est_tpm <-
  sample1_gene_fc$fpkm_fc_unionexon/sum(sample1_gene_fc$fpkm_fc_unionexon, na.rm = TRUE) * 1e6
sample1_gene_fc$measure <- "featureCounts_FPKM_gene"

## Merge into one data frame
df <- rbind(sample1_tx_salmon[, c("measure", "true_tpm", "est_tpm")],
            sample1_gene_salmon[, c("measure", "true_tpm", "est_tpm")],
            sample1_gene_fc[, c("measure", "true_tpm", "est_tpm")])
df$measure <- factor(df$measure, levels = c("salmon_transcript", "salmon_gene", 
                                            "featureCounts_FPKM_gene"))

cors <- df %>% group_by(measure) %>%
  summarise(sp = signif(cor(true_tpm, est_tpm, use = "complete", method = "spearman"), 3))

ggplot(df, aes(x = true_tpm, y = est_tpm, col = measure)) + 
  scale_colour_manual(values = c("blue", "red", "dimgrey"), guide = FALSE) + 
  geom_abline(intercept = 0, slope = 1) + 
  facet_grid(~ measure) + 
  geom_point(alpha = 0.5) + 
  scale_x_log10() + scale_y_log10() + 
  xlab("True TPM") + ylab("Estimated TPM") + 
  plot_theme() + 
  geom_text(x = 3, y = -5, aes(label = paste0("cor = ", sp)), size = 3, data = cors)
```

### Only features with positive true and estimated TPMs

(Back to top)

```
df_sub <- subset(df, true_tpm > 0 & est_tpm > 0)
cors_sub <- df_sub %>% group_by(measure) %>%
  summarise(sp = signif(cor(true_tpm, est_tpm, use = "complete", method = "spearman"), 3))

ggplot(df_sub, aes(x = true_tpm, y = est_tpm, col = measure)) + 
  scale_colour_manual(values = c("blue", "red", "dimgrey"), guide = FALSE) + 
  geom_abline(intercept = 0, slope = 1) + 
  facet_grid(~ measure) + 
  geom_point(alpha = 0.5) + 
  scale_x_log10() + scale_y_log10() + 
  xlab("True TPM") + ylab("Estimated TPM") + 
  plot_theme() + 
  geom_text(x = 3, y = -5, aes(label = paste0("cor = ", sp)), size = 3, data = cors_sub)
```

### Consider groups of paralogous genes only

(Back to top)

```
## Consider only gene-level estimates
dfg <- rbind(sample1_gene_salmon[, c("gene", "measure", "true_tpm", "est_tpm")],
             sample1_gene_fc[, c("gene", "measure", "true_tpm", "est_tpm")])
dfg$measure <- factor(dfg$measure, levels = c("salmon_gene", 
                                              "featureCounts_FPKM_gene"))

## Find paralogous genes using biomaRt
ensembl <- useMart("ENSEMBL_MART_ENSEMBL", host = "www.ensembl.org")
ensembl <- useDataset("hsapiens_gene_ensembl", mart = ensembl)
bm <- getBM(attributes = c("ensembl_gene_id", "hsapiens_paralog_ensembl_gene"),
            filters = "ensembl_gene_id", values = dfg$gene, mart = ensembl)
bm <- bm[which(bm$hsapiens_paralog_ensembl_gene != ""), ]
bm <- subset(bm, hsapiens_paralog_ensembl_gene %in% dfg$gene)

tt <- sort(table(bm$ensembl_gene_id), decreasing = TRUE)
tt <- tt[which(tt >= 10)]
basisgenes <- c()
for (nm in names(tt)) {
  if (!any(c(nm, bm$hsapiens_paralog_ensembl_gene[which(bm$ensembl_gene_id == nm)]) %in% basisgenes)) {
      tmpdf <- subset(dfg, gene %in% c(nm, bm$hsapiens_paralog_ensembl_gene[which(bm$ensembl_gene_id == nm)]))
      if (median(tmpdf$true_tpm) > 5) {
        basisgenes <- c(basisgenes, nm)
      }
  }
}

pdf("paralogs_sim1.pdf", width = 7, height = 5)
paralogs_spearman_salmon <- c()
paralogs_spearman_featurecounts <- c()
random_spearman_salmon <- c()
random_spearman_featurecounts <- c()
paralogs_pearson_salmon <- c()
paralogs_pearson_featurecounts <- c()
random_pearson_salmon <- c()
random_pearson_featurecounts <- c()
for (gn in basisgenes) {
  dfg_sub <- subset(dfg, gene %in% c(gn, bm$hsapiens_paralog_ensembl_gene[which(bm$ensembl_gene_id == gn)]))
  cors_sub <- dfg_sub %>% group_by(measure) %>%
    summarise(sp = signif(cor(true_tpm, est_tpm, use = "complete", method = "spearman"), 3),
              pe = signif(cor(log10(true_tpm + 1), log10(est_tpm + 1), 
                              use = "complete", method = "pearson"), 3))
  paralogs_spearman_salmon <- c(paralogs_spearman_salmon, 
                                cors_sub$sp[which(cors_sub$measure == "salmon_gene")])
  paralogs_spearman_featurecounts <- c(paralogs_spearman_featurecounts, 
                                       cors_sub$sp[which(cors_sub$measure == "featureCounts_FPKM_gene")])
  paralogs_pearson_salmon <- c(paralogs_pearson_salmon, 
                               cors_sub$pe[which(cors_sub$measure == "salmon_gene")])
  paralogs_pearson_featurecounts <- c(paralogs_pearson_featurecounts, 
                                      cors_sub$pe[which(cors_sub$measure == "featureCounts_FPKM_gene")])
  xpos <- 0.25 * min(log10(dfg_sub$true_tpm[dfg_sub$true_tpm > 0])) + 
    0.75 * max(log10(dfg_sub$true_tpm[dfg_sub$true_tpm > 0]))
  ypos <- 0.75 * min(log10(dfg_sub$est_tpm[dfg_sub$est_tpm > 0])) + 
    0.25 * max(log10(dfg_sub$est_tpm[dfg_sub$est_tpm > 0]))
  ypos2 <- 0.8 * min(log10(dfg_sub$est_tpm[dfg_sub$est_tpm > 0])) + 
    0.2 * max(log10(dfg_sub$est_tpm[dfg_sub$est_tpm > 0]))
  print(ggplot(dfg_sub, aes(x = true_tpm, y = est_tpm, col = measure)) + 
          scale_colour_manual(values = c("red", "dimgrey"), guide = FALSE) + 
          geom_abline(intercept = 0, slope = 1) + 
          facet_grid(~ measure) + 
          geom_point(size = 2) + 
          scale_x_log10() + scale_y_log10() + 
          xlab("True TPM") + ylab("Estimated TPM") + 
          plot_theme() + ggtitle(gn) + 
          geom_text(x = xpos, y = ypos, aes(label = paste0("spearman = ", sp)), size = 3, data = cors_sub) + 
          geom_text(x = xpos, y = ypos2, aes(label = paste0("pearson = ", pe)), size = 3, data = cors_sub))
  
  ## Extract a random set of genes of the same size 
  random_genes <- sample(unique(dfg$gene), length(unique(dfg_sub$gene)))
  random_dfg_sub <- subset(dfg, gene %in% random_genes)
  random_cors_sub <- random_dfg_sub %>% group_by(measure) %>%
    summarise(sp = signif(cor(true_tpm, est_tpm, use = "complete", method = "spearman"), 3),
              pe = signif(cor(log10(true_tpm + 1), log10(est_tpm + 1), 
                              use = "complete", method = "spearman"), 3))
  random_spearman_salmon <- c(random_spearman_salmon, 
                              random_cors_sub$sp[which(random_cors_sub$measure == "salmon_gene")])
  random_spearman_featurecounts <- c(random_spearman_featurecounts, 
                                     random_cors_sub$sp[which(random_cors_sub$measure ==
                                                                "featureCounts_FPKM_gene")])
  random_pearson_salmon <- c(random_pearson_salmon, 
                             random_cors_sub$pe[which(random_cors_sub$measure == "salmon_gene")])
  random_pearson_featurecounts <- c(random_pearson_featurecounts, 
                                    random_cors_sub$pe[which(random_cors_sub$measure ==
                                                               "featureCounts_FPKM_gene")])
}
dev.off()
```

```
## pdf 
##   2
```

```
## Plot distribution of correlation coefficients
plot_df <- data.frame(correlation = c(paralogs_spearman_salmon, paralogs_spearman_featurecounts,
                                      random_spearman_salmon, random_spearman_featurecounts,
                                      paralogs_pearson_salmon, paralogs_pearson_featurecounts,
                                      random_pearson_salmon, random_pearson_featurecounts),
                      method = rep(c("salmon_gene", "featureCounts_FPKM_gene", 
                                     "salmon_gene", "featureCounts_FPKM_gene",
                                     "salmon_gene", "featureCounts_FPKM_gene", 
                                     "salmon_gene", "featureCounts_FPKM_gene"),
                                   c(length(paralogs_spearman_salmon), length(paralogs_spearman_featurecounts),
                                     length(random_spearman_salmon), length(random_spearman_featurecounts),
                                     length(paralogs_pearson_salmon), length(paralogs_pearson_featurecounts),
                                     length(random_pearson_salmon), length(random_pearson_featurecounts))),
                      gtype = rep(c("paralogs", "paralogs", 
                                    "random", "random",
                                    "paralogs", "paralogs", 
                                    "random", "random"),
                                  c(length(paralogs_spearman_salmon), length(paralogs_spearman_featurecounts),
                                    length(random_spearman_salmon), length(random_spearman_featurecounts),
                                    length(paralogs_pearson_salmon), length(paralogs_pearson_featurecounts),
                                    length(random_pearson_salmon), length(random_pearson_featurecounts))),
                      ctype = rep(c("spearman", "pearson"),
                                  c(length(paralogs_spearman_salmon) + length(paralogs_spearman_featurecounts) +
                                    length(random_spearman_salmon) + length(random_spearman_featurecounts),
                                    length(paralogs_pearson_salmon) + length(paralogs_pearson_featurecounts) +
                                    length(random_pearson_salmon) + length(random_pearson_featurecounts))),
                      stringsAsFactors = FALSE)
plot_df$method <- factor(plot_df$method, levels = c("salmon_gene", "featureCounts_FPKM_gene"))
ggplot(plot_df, aes(x = correlation, group = method, col = method)) + 
  geom_line(stat = "density", size = 1.5) + 
  facet_grid(ctype ~ gtype) + 
  scale_colour_manual(values = c("red", "dimgrey"), name = "") + 
  xlab("Correlation") + ylab("Density") + 
  plot_theme() + theme(legend.position = "bottom")
```

## Variability of transcript and gene TPMs

(Back to top)

To quantify the variability of the transcript and gene TPM estimates obtained with Salmon, we calculate the coefficients of variation across 30 bootstrap replicates for one of the samples in the data set.

```
sample1_tx_salmon <- merge(sample1_tx_salmon, 
                           data.frame(feature = rownames(CV_tx_salmon),
                                      coefvar = CV_tx_salmon[, "sample1"],
                                      stringsAsFactors = FALSE),
                           by = "feature", all = TRUE)

sample1_gene_salmon <- merge(sample1_gene_salmon, 
                             data.frame(gene = rownames(CV_gene_salmon),
                                        coefvar = CV_gene_salmon[, "sample1"],
                                        stringsAsFactors = FALSE),
                             by = "gene", all = TRUE)

cvs_sal <- data.frame(cvs = c(sample1_tx_salmon$coefvar, sample1_gene_salmon$coefvar), 
                      lev = rep(c("transcript", "gene"), c(nrow(sample1_tx_salmon), 
                                                           nrow(sample1_gene_salmon))))
ggplot(cvs_sal, aes(x = log10(cvs + 0.001), col = lev)) + 
  geom_line(stat = "density", size = 2) + 
  ggtitle("") + 
  xlab("log10(coefficient of variation + 0.001)") + ylab("density") + 
  scale_colour_manual(values = c("red", "blue"), name = "") + 
  plot_theme()
```

## Correlation between gene count estimates

(Back to top)

### All genes

(Back to top)

```
geneCOUNT_fc <- fc_sim1$counts[match(rownames(salmon_quant$geneCOUNT_sal_simplesum),
                                     rownames(fc_sim1$counts)), ]
stopifnot(all(rownames(salmon_quant$geneCOUNT_sal_simplesum) == 
                rownames(salmon_quant$geneCOUNT_sal_scaledTPM)))
sample1 <- cbind(simplesum_salmon = salmon_quant$geneCOUNT_sal_simplesum[, "sample1"], 
                 scaledTPM_salmon = salmon_quant$geneCOUNT_sal_scaledTPM[, "sample1"],
                 featureCounts = geneCOUNT_fc[, "sample1"])
rownames(sample1) <- rownames(salmon_quant$geneCOUNT_sal_simplesum)
sample1 <- log2(sample1 + 1)
pairs(sample1, upper.panel = panel_smooth, lower.panel = panel_cor)
```

### Only genes with positive estimates with all methods

(Back to top)

```
pairs(sample1[which(rowSums(sample1 <= 0) == 0), ], upper.panel = panel_smooth,
      lower.panel = panel_cor)
```

### Example of genes with a big difference between simple sum and scaled TPM:

(Back to top)

```
idx <- rownames(sample1)[order(abs(sample1[, "simplesum_salmon"] - sample1[, "scaledTPM_salmon"]),
                               decreasing = TRUE)[1:5]]
2^sample1[idx, ] - 1
```

```
##                 simplesum_salmon scaledTPM_salmon featureCounts
## ENSG00000267987        18.430100        1399.8485             0
## ENSG00000250273         3.873710         319.4192            NA
## ENSG00000239756         4.244434         227.0874            NA
## ENSG00000228253     14292.000000      600254.7484         12826
## ENSG00000256512         2.399900         139.1988            NA
```

```
subset(tx2gene, gene %in% idx)
```

```
##             transcript            gene
## 140571 ENST00000361851 ENSG00000228253
## 144886 ENST00000594963 ENSG00000267987
## 160898 ENST00000512850 ENSG00000250273
## 180437 ENST00000414166 ENSG00000239756
## 180438 ENST00000442136 ENSG00000239756
## 180439 ENST00000418028 ENSG00000239756
## 180440 ENST00000441437 ENSG00000239756
## 180441 ENST00000453736 ENSG00000239756
## 180442 ENST00000414695 ENSG00000239756
## 180443 ENST00000422985 ENSG00000239756
## 190851 ENST00000543527 ENSG00000256512
```

## Differential transcript expression (DTE) analysis on gene and transcript level

(Back to top)

We perform DTE by applying edgeR to transcript-level counts obtained from Salmon. The p-values from edgeR are then aggregated into gene-wise FDR estimates using the `perGeneQValue` function from `DEXSeq`.

```
res_dte_salmon_tx <- diff_expression_edgeR(counts = salmon_quant$txCOUNT_sal, 
                                           meta = meta, cond_name = "condition", 
                                           sample_name = "sample", 
                                           gene_length_matrix = NULL) 
res_dte_salmon_tx$tt$gene <- as.character(tx2gene$gene[match(rownames(res_dte_salmon_tx$tt), 
                                                             tx2gene$transcript)])

## Summarise on gene level
geneSplit = split(seq(along = res_dte_salmon_tx$tt$gene), res_dte_salmon_tx$tt$gene)
pGene = sapply(geneSplit, function(i) min(res_dte_salmon_tx$tt$PValue[i]))
stopifnot(all(is.finite(pGene)))
theta = unique(sort(pGene))
q = DEXSeq:::perGeneQValueExact(pGene, theta, geneSplit)
qval_dte_salmon_gene = rep(NA_real_, length(pGene))
qval_dte_salmon_gene = q[match(pGene, theta)]
qval_dte_salmon_gene = pmin(1, qval_dte_salmon_gene)
names(qval_dte_salmon_gene) = names(geneSplit)
stopifnot(!any(is.na(qval_dte_salmon_gene)))
```

### Comparison of DTE performance on gene and transcript level

(Back to top)

Here, we compare the DTE performance evaluated on transcript- and gene-level. Note that both the result level (transcript results vs aggregated gene results) and the truth (differentially expressed transcript vs genes with at least one differentially expressed transcript) are different, and the performance is evaluated by comparing each result table to its respective truth.

```
truth_tx <- read.delim(truth_tx_file, header = TRUE, as.is = TRUE)
truth_any <- read.delim(truth_gene_file, header = TRUE, as.is = TRUE)
cobra_dte <- COBRAData(padj = data.frame(transcript_salmon = res_dte_salmon_tx$tt$FDR, 
                                         row.names = rownames(res_dte_salmon_tx$tt)),
                       truth = data.frame(truth_tx[, c("transcript_ds_status", "diff_IsoPct"), 
                                                   drop = FALSE], 
                                          row.names = truth_tx$transcript_id,
                                          stringsAsFactors = FALSE))

tmp <- truth_any[, c("ds_status", "diff_IsoPct"), drop = FALSE]
colnames(tmp) <- c("transcript_ds_status", "diff_IsoPct")
cobra_dte <- COBRAData(padj = data.frame(gene_salmon = qval_dte_salmon_gene, 
                                         row.names = names(qval_dte_salmon_gene)),
                       truth = data.frame(tmp, 
                                          row.names = truth_any$gene,
                                          stringsAsFactors = FALSE),
                       object_to_extend = cobra_dte)
truth(cobra_dte)$diffisopct <- cut2(truth(cobra_dte)$diff_IsoPct, cuts = c(0, 1/3, 2/3, 1))
cobraperf_dte <- calculate_performance(cobra_dte, binary_truth = "transcript_ds_status",
                                       aspects = c("fdrtpr", "fdrtprcurve"),
                                       onlyshared = TRUE)
cobraplot_dte <- prepare_data_for_plot(cobraperf_dte, colorscheme = c("red", "blue"), 
                                       facetted = FALSE)
```

```
fdrtpr(cobraplot_dte)$fullmethod <- gsub("_overall", "", fdrtpr(cobraplot_dte)$fullmethod)
fdrtprcurve(cobraplot_dte)$fullmethod <- gsub("_overall", "", fdrtprcurve(cobraplot_dte)$fullmethod)
plot_fdrtprcurve(cobraplot_dte, pointsize = 2) + plot_theme() + 
  theme(legend.position = "bottom")
```

```
## Stratify by diffisopct
cobraperf2_dte <- calculate_performance(cobra_dte, binary_truth = "transcript_ds_status",
                                        aspects = c("fdrtpr", "fdrtprcurve"),
                                        onlyshared = TRUE, splv = "diffisopct")
cobraplot2_dte <- prepare_data_for_plot(cobraperf2_dte, colorscheme = c("red", "blue"), 
                                        facetted = TRUE)
plot_fdrtprcurve(cobraplot2_dte, pointsize = 2) + plot_theme() + theme(legend.position = "bottom")
```

## Differential gene expression analysis (edgeR)

(Back to top)

Given the gene count matrices defined above (corresponding to the **featureCounts**, **simplesum** and **scaledTPM** matrices mentioned in the manuscript), we apply *edgeR* (see below for *DESeq2* results) to perform differential gene expression. For the **simplesum** and **featureCounts** matrices, we also perform the analyses using the offsets derived from the average transcript lengths (**simplesum\_avetxl**).

```
res_sal_simplesum_edgeR <- diff_expression_edgeR(counts = salmon_quant$geneCOUNT_sal_simplesum, 
                                                 meta = meta, cond_name = "condition", 
                                                 sample_name = "sample", 
                                                 gene_length_matrix = NULL)
res_sal_simplesum_avetxl_edgeR <- diff_expression_edgeR(counts = salmon_quant$geneCOUNT_sal_simplesum, 
                                                        meta = meta, cond_name = "condition", 
                                                        sample_name = "sample", 
                                                        gene_length_matrix = salmon_quant$avetxlength)
res_sal_scaledTPM_edgeR <- diff_expression_edgeR(counts = salmon_quant$geneCOUNT_sal_scaledTPM, 
                                                 meta = meta, cond_name = "condition", 
                                                 sample_name = "sample", 
                                                 gene_length_matrix = NULL)
res_fc_edgeR <- diff_expression_edgeR(counts = geneCOUNT_fc, 
                                      meta = meta, cond_name = "condition", 
                                      sample_name = "sample", 
                                      gene_length_matrix = NULL)
res_fc_avetxl_edgeR <- diff_expression_edgeR(counts = geneCOUNT_fc, 
                                             meta = meta, cond_name = "condition", 
                                             sample_name = "sample", 
                                             gene_length_matrix = salmon_quant$avetxlength)
```

#### Diagnostics

(Back to top)

```
dfh <- data.frame(pvalue = c(res_fc_edgeR$tt$PValue,
                             res_fc_avetxl_edgeR$tt$PValue,
                             res_sal_scaledTPM_edgeR$tt$PValue,
                             res_sal_simplesum_edgeR$tt$PValue, 
                             res_sal_simplesum_avetxl_edgeR$tt$PValue),
                  mth = c(rep("featureCounts", nrow(res_fc_edgeR$tt)),
                          rep("featureCounts_avetxl", nrow(res_fc_avetxl_edgeR$tt)),
                          rep("scaledTPM_salmon", nrow(res_sal_scaledTPM_edgeR$tt)),
                          rep("simplesum_salmon", nrow(res_sal_simplesum_edgeR$tt)),
                          rep("simplesum_salmon_avetxl", nrow(res_sal_simplesum_avetxl_edgeR$tt))))
ggplot(dfh, aes(x = pvalue)) + geom_histogram() + facet_wrap(~mth) + 
  plot_theme() + 
  xlab("p-value") + ylab("count")
```

```
par(mfrow = c(2, 3))
plotBCV(res_fc_edgeR$dge, main = "featureCounts")
plotBCV(res_fc_avetxl_edgeR$dge, main = "featureCounts_avetxl")
plotBCV(res_sal_scaledTPM_edgeR$dge, main = "scaledTPM_salmon")
plotBCV(res_sal_simplesum_edgeR$dge, main = "simplesum_salmon")
plotBCV(res_sal_simplesum_avetxl_edgeR$dge, main = "simplesum_salmon_avetxl")
```

```
par(mfrow = c(2, 3))
plotSmear(res_fc_edgeR$dge, main = "featureCounts", ylim = c(-10, 10))
plotSmear(res_fc_avetxl_edgeR$dge, main = "featureCounts_avetxl", ylim = c(-10, 10))
plotSmear(res_sal_scaledTPM_edgeR$dge, main = "scaledTPM_salmon", ylim = c(-10, 10))
plotSmear(res_sal_simplesum_edgeR$dge, main = "simplesum_salmon", ylim = c(-10, 10))
plotSmear(res_sal_simplesum_avetxl_edgeR$dge, main = "simplesum_salmon_avetxl", ylim = c(-10, 10))
par(mfrow = c(1, 1))
```

### Comparison of significant genes found with different matrices

(Back to top)

```
cobra_edgeR <- COBRAData(padj = data.frame(simplesum_salmon = res_sal_simplesum_edgeR$tt$FDR, 
                                           row.names = rownames(res_sal_simplesum_edgeR$tt)))
cobra_edgeR <- COBRAData(padj = data.frame(scaledTPM_salmon = res_sal_scaledTPM_edgeR$tt$FDR, 
                                           row.names = rownames(res_sal_scaledTPM_edgeR$tt)),
                         object_to_extend = cobra_edgeR)
cobra_edgeR <- COBRAData(padj = data.frame(simplesum_salmon_avetxl = res_sal_simplesum_avetxl_edgeR$tt$FDR, 
                                           row.names = rownames(res_sal_simplesum_avetxl_edgeR$tt)),
                         object_to_extend = cobra_edgeR)
cobra_edgeR <- COBRAData(padj = data.frame(featureCounts = res_fc_edgeR$tt$FDR,
                                           row.names = rownames(res_fc_edgeR$tt)), 
                         object_to_extend = cobra_edgeR)
cobra_edgeR <- COBRAData(padj = data.frame(featureCounts_avetxl = res_fc_avetxl_edgeR$tt$FDR,
                                           row.names = rownames(res_fc_avetxl_edgeR$tt)), 
                         object_to_extend = cobra_edgeR)
truth_gene <- read.delim(truth_gene_file, header = TRUE, as.is = TRUE, row.names = 1)
cobra_edgeR <- COBRAData(truth = truth_gene, object_to_extend = cobra_edgeR)
cobraperf_edgeR <- calculate_performance(cobra_edgeR, aspects = "overlap")
cobraplot1_edgeR <- prepare_data_for_plot(cobraperf_edgeR, incltruth = FALSE, 
                                          colorscheme = muted[c(1, 3, 5, 8, 10)])
plot_overlap(cobraplot1_edgeR, cex = c(1, 0.7, 0.7))
```

### FPR calculation

(Back to top)

```
plot_perftable <- function(cobradata, threshold, splv, axis_size = 15) {
  if (splv != "none")
    trt <- split(truth(cobradata), truth(cobradata)[, splv])
  else
    trt <- list(all = truth(cobradata))
  methods <- colnames(pval(cobradata))
  for (m in seq_along(methods)) {
    A <- do.call(rbind, lapply(trt, function(w) {
      de <- length(which(w$de_status == 1))
      nonde <- length(which(w$de_status == 0))
      TP <- length(intersect(rownames(w)[w$de_status == 1], 
                             rownames(pval(cobradata))[pval(cobradata)[, m] <= threshold]))
      FP <- length(intersect(rownames(w)[w$de_status == 0], 
                             rownames(pval(cobradata))[pval(cobradata)[, m] <= threshold]))
      FN <- length(intersect(rownames(w)[w$de_status == 1], 
                             rownames(pval(cobradata))[pval(cobradata)[, m] > threshold]))
      TN <- length(intersect(rownames(w)[w$de_status == 0], 
                             rownames(pval(cobradata))[pval(cobradata)[, m] > threshold]))
      tot.called <- TP + FP + FN + TN
      tot.status <- de + nonde
      FPR <- signif(FP/(FP + TN), digits = 2)
      c(de = de, nonde = nonde, tot.status = tot.status, 
        TP = TP, FP = FP, FN = FN, TN = TN, FPR = FPR, tot.called = tot.called)
    }))
    B <- melt(A)
    B$fillcolor <- "white"
    B$fillcolor[B$Var2 == "de"] <- "lightgreen"
    B$fillcolor[B$Var2 == "nonde"] <- "pink"
    B$fillcolor[B$Var2 == "FPR"] <- "grey"
    fc <- unique(B$fillcolor)
    names(fc) <- fc
    print(ggplot(B, aes(Var2, Var1, fill = fillcolor)) + 
            geom_tile() + 
            geom_text(aes(label = value), color = "black", size = 5) + 
            scale_fill_manual(values = fc, name = "") + 
            scale_x_discrete(expand = c(0, 0)) + 
            scale_y_discrete(expand = c(0, 0)) + 
            xlab("") + ylab(splv) + 
            geom_vline(xintercept = 3.5, linetype = "dashed") + 
            theme(panel.background = element_rect(fill = NA, colour = NA),
                  legend.position = "none",
                  axis.ticks = element_blank(),
                  plot.title = element_text(size = 20),
                  axis.text.x = element_text(size = axis_size),
                  axis.text.y = element_text(size = axis_size),
                  axis.title.y = element_text(size = axis_size)) + 
            ggtitle(paste0(methods[m], ", threshold ", threshold)))
  }
}  

cobra <- COBRAData(pval = data.frame(simplesum_salmon = res_sal_simplesum_edgeR$tt$PValue, 
                                     row.names = rownames(res_sal_simplesum_edgeR$tt)))
cobra <- COBRAData(pval = data.frame(scaledTPM_salmon = res_sal_scaledTPM_edgeR$tt$PValue, 
                                     row.names = rownames(res_sal_scaledTPM_edgeR$tt)),
                   object_to_extend = cobra)
cobra <- COBRAData(pval = data.frame(simplesum_salmon_avetxl = res_sal_simplesum_avetxl_edgeR$tt$PValue, 
                                     row.names = rownames(res_sal_simplesum_avetxl_edgeR$tt)),
                   object_to_extend = cobra)
cobra <- COBRAData(pval = data.frame(featureCounts = res_fc_edgeR$tt$PValue,
                                     row.names = rownames(res_fc_edgeR$tt)), 
                   object_to_extend = cobra)
cobra <- COBRAData(pval = data.frame(featureCounts_avetxl = res_fc_avetxl_edgeR$tt$PValue,
                                     row.names = rownames(res_fc_avetxl_edgeR$tt)), 
                   object_to_extend = cobra)
truth_gene <- read.delim(truth_gene_file, header = TRUE, as.is = TRUE, row.names = 1)

truth_gene$diffspliced <- rep("no_dtu", nrow(truth_gene))
truth_gene$diffspliced[which(truth_gene$ds_status == 1)] <- "dtu"

truth_gene$diffisopct <- cut2(truth_gene$diff_IsoPct, cuts = c(0, 1/3, 2/3, 1))
truth_gene$diffsp_diffisopct <- interaction(truth_gene$diffspliced, truth_gene$diffisopct)

truth_gene$diffbp <- cut2(truth_gene$diffbp, 
                          cuts = c(100, 1000, max(truth_gene$diffbp, na.rm = TRUE)))
truth_gene$diffsp_diffisopct_diffbp <- droplevels(interaction(interaction(truth_gene$diffspliced, 
                                                                          truth_gene$diffisopct),
                                                              truth_gene$diffbp))

truth_gene$rellengthdiff <- isoform_differences$rellengthdiff[match(rownames(truth_gene), 
                                                                    isoform_differences$gene_id)]
truth_gene$rellengthdiff <- cut2(truth_gene$rellengthdiff, cuts = c(0, 1/3, 2/3, 1))
truth_gene$diffsp_diffisopct_rld <- droplevels(interaction(interaction(truth_gene$diffspliced,
                                                                       truth_gene$diffisopct),
                                                           truth_gene$rellengthdiff))

truth_gene$lengthratio <- isoform_differences$lengthratio[match(rownames(truth_gene), 
                                                                isoform_differences$gene_id)]
truth_gene$lengthratio <- cut2(truth_gene$lengthratio, g = 3)
truth_gene$diffsp_diffisopct_lrt <- droplevels(interaction(interaction(truth_gene$diffspliced,
                                                                       truth_gene$diffisopct),
                                                           truth_gene$lengthratio))

cobra <- COBRAData(truth = truth_gene, object_to_extend = cobra)
```

```
plot_perftable(cobradata = cobra, threshold = 0.05, splv = "diffspliced")
```

```
plot_perftable(cobradata = cobra, threshold = 0.05, splv = "diffisopct")
```

```
plot_perftable(cobradata = cobra, threshold = 0.05, splv = "diffsp_diffisopct")
```

```
plot_perftable(cobradata = cobra, threshold = 0.05, splv = "diffbp")
```

```
plot_perftable(cobradata = cobra, threshold = 0.05, splv = "diffsp_diffisopct_diffbp")
```

```
plot_perftable(cobradata = cobra, threshold = 0.05, splv = "diffsp_diffisopct_rld")
```

```
plot_perftable(cobradata = cobra, threshold = 0.05, splv = "diffsp_diffisopct_lrt")
```

## Differential gene expression analysis (DESeq2)

(Back to top)

```
res_sal_simplesum_deseq2 <- diff_expression_DESeq2(txi = NULL, 
                                                   counts = salmon_quant$geneCOUNT_sal_simplesum, 
                                                   meta = meta, cond_name = "condition", 
                                                   level1 = "A", level2 = "B",
                                                   sample_name = "sample")
res_sal_simplesum_avetxl_deseq2 <- diff_expression_DESeq2(txi = salmon_quant$txi_salmonsimplesum,
                                                          counts = NULL, 
                                                          meta = meta, cond_name = "condition", 
                                                          level1 = "A", level2 = "B",
                                                          sample_name = "sample")
res_sal_scaledTPM_deseq2 <- diff_expression_DESeq2(txi = NULL,
                                                   counts = salmon_quant$geneCOUNT_sal_scaledTPM, 
                                                   meta = meta, cond_name = "condition", 
                                                   level1 = "A", level2 = "B",
                                                   sample_name = "sample")
res_fc_deseq2 <- diff_expression_DESeq2(txi = NULL,
                                        counts = geneCOUNT_fc, 
                                        meta = meta, cond_name = "condition", 
                                        level1 = "A", level2 = "B",
                                        sample_name = "sample")
res_fc_avetxl_deseq2 <- 
  diff_expression_DESeq2(txi = list(counts = geneCOUNT_fc, 
                                    length = salmon_quant$avetxlength[match(rownames(geneCOUNT_fc),
                                                                            rownames(salmon_quant$avetxlength)),
                                                                      match(colnames(geneCOUNT_fc),
                                                                            colnames(salmon_quant$avetxlength))],
                                    countsFromAbundance = "no"),
                         counts = NULL, 
                         meta = meta, cond_name = "condition", 
                         level1 = "A", level2 = "B",
                         sample_name = "sample")
```

#### Diagnostics

(Back to top)

```
dfh <- data.frame(pvalue = c(res_fc_deseq2$res$pvalue,
                             res_fc_avetxl_deseq2$res$pvalue,
                             res_sal_scaledTPM_deseq2$res$pvalue,
                             res_sal_simplesum_deseq2$res$pvalue, 
                             res_sal_simplesum_avetxl_deseq2$res$pvalue),
                  mth = c(rep("featureCounts", nrow(res_fc_deseq2$res)),
                          rep("featureCounts_avetxl", nrow(res_fc_avetxl_deseq2$res)),
                          rep("scaledTPM_salmon", nrow(res_sal_scaledTPM_deseq2$res)),
                          rep("simplesum_salmon", nrow(res_sal_simplesum_deseq2$res)),
                          rep("simplesum_salmon_avetxl", nrow(res_sal_simplesum_avetxl_deseq2$res))))
ggplot(dfh, aes(x = pvalue)) + geom_histogram() + facet_wrap(~mth) + 
  plot_theme() + 
  xlab("p-value") + ylab("count")
```

```
par(mfrow = c(2, 3))
plotDispEsts(res_fc_deseq2$dsd, main = "featureCounts")
plotDispEsts(res_fc_avetxl_deseq2$dsd, main = "featureCounts_avetxl")
plotDispEsts(res_sal_scaledTPM_deseq2$dsd, main = "scaledTPM_salmon")
plotDispEsts(res_sal_simplesum_deseq2$dsd, main = "simplesum_salmon")
plotDispEsts(res_sal_simplesum_avetxl_deseq2$dsd, main = "simplesum_salmon_avetxl")
```

```
par(mfrow = c(2, 3))
DESeq2::plotMA(res_fc_deseq2$dsd, main = "featureCounts")
DESeq2::plotMA(res_fc_avetxl_deseq2$dsd, main = "featureCounts_avetxl")
DESeq2::plotMA(res_sal_scaledTPM_deseq2$dsd, main = "scaledTPM_salmon")
DESeq2::plotMA(res_sal_simplesum_deseq2$dsd, main = "simplesum_salmon")
DESeq2::plotMA(res_sal_simplesum_avetxl_deseq2$dsd, main = "simplesum_salmon_avetxl")
par(mfrow = c(1, 1))
```

### Comparison of significant genes found with different matrices

(Back to top)

```
cobra <- COBRAData(padj = data.frame(simplesum_salmon = res_sal_simplesum_deseq2$res$padj, 
                                     row.names = rownames(res_sal_simplesum_deseq2$res)))
cobra <- COBRAData(padj = data.frame(scaledTPM_salmon = res_sal_scaledTPM_deseq2$res$padj, 
                                     row.names = rownames(res_sal_scaledTPM_deseq2$res)),
                   object_to_extend = cobra)
cobra <- COBRAData(padj = data.frame(simplesum_salmon_avetxl = res_sal_simplesum_avetxl_deseq2$res$padj, 
                                     row.names = rownames(res_sal_simplesum_avetxl_deseq2$res)),
                   object_to_extend = cobra)
cobra <- COBRAData(padj = data.frame(featureCounts = res_fc_deseq2$res$padj,
                                     row.names = rownames(res_fc_deseq2$res)), 
                   object_to_extend = cobra)
cobra <- COBRAData(padj = data.frame(featureCounts_avetxl = res_fc_avetxl_deseq2$res$padj,
                                     row.names = rownames(res_fc_avetxl_deseq2$res)), 
                   object_to_extend = cobra)
truth_gene <- read.delim(truth_gene_file, header = TRUE, as.is = TRUE, row.names = 1)
cobra <- COBRAData(truth = truth_gene, object_to_extend = cobra)
cobraperf <- calculate_performance(cobra, aspects = "overlap")
cobraplot1 <- prepare_data_for_plot(cobraperf, incltruth = FALSE, 
                                    colorscheme = muted[c(1, 3, 5, 8, 10)])
plot_overlap(cobraplot1, cex = c(1, 0.7, 0.7))
```

### FPR calculation

(Back to top)

```
cobra <- COBRAData(pval = data.frame(simplesum_salmon = res_sal_simplesum_deseq2$res$pvalue, 
                                     row.names = rownames(res_sal_simplesum_deseq2$res)))
cobra <- COBRAData(pval = data.frame(scaledTPM_salmon = res_sal_scaledTPM_deseq2$res$pvalue, 
                                     row.names = rownames(res_sal_scaledTPM_deseq2$res)),
                   object_to_extend = cobra)
cobra <- COBRAData(pval = data.frame(simplesum_salmon_avetxl = res_sal_simplesum_avetxl_deseq2$res$pvalue, 
                                     row.names = rownames(res_sal_simplesum_avetxl_deseq2$res)),
                   object_to_extend = cobra)
cobra <- COBRAData(pval = data.frame(featureCounts = res_fc_deseq2$res$pvalue,
                                     row.names = rownames(res_fc_deseq2$res)), 
                   object_to_extend = cobra)
cobra <- COBRAData(pval = data.frame(featureCounts_avetxl = res_fc_avetxl_deseq2$res$pvalue,
                                     row.names = rownames(res_fc_avetxl_deseq2$res)), 
                   object_to_extend = cobra)
truth_gene <- read.delim(truth_gene_file, header = TRUE, as.is = TRUE, row.names = 1)

truth_gene$diffspliced <- rep("no_dtu", nrow(truth_gene))
truth_gene$diffspliced[which(truth_gene$ds_status == 1)] <- "dtu"

truth_gene$diffisopct <- cut2(truth_gene$diff_IsoPct, cuts = c(0, 1/3, 2/3, 1))
truth_gene$diffsp_diffisopct <- interaction(truth_gene$diffspliced, truth_gene$diffisopct)
truth_gene$diffbp <- cut2(truth_gene$diffbp, 
                          cuts = c(100, 1000, max(truth_gene$diffbp, na.rm = TRUE)))
truth_gene$diffsp_diffisopct_diffbp <- droplevels(interaction(interaction(truth_gene$diffspliced, 
                                                                          truth_gene$diffisopct),
                                                              truth_gene$diffbp))

truth_gene$rellengthdiff <- isoform_differences$rellengthdiff[match(rownames(truth_gene), 
                                                                    isoform_differences$gene_id)]
truth_gene$rellengthdiff <- cut2(truth_gene$rellengthdiff, cuts = c(0, 1/3, 2/3, 1))
truth_gene$diffsp_diffisopct_rld <- droplevels(interaction(interaction(truth_gene$diffspliced,
                                                                       truth_gene$diffisopct),
                                                           truth_gene$rellengthdiff))

truth_gene$lengthratio <- isoform_differences$lengthratio[match(rownames(truth_gene), 
                                                              isoform_differences$gene_id)]
truth_gene$lengthratio <- cut2(truth_gene$lengthratio, g = 3)
truth_gene$diffsp_diffisopct_lrt <- droplevels(interaction(interaction(truth_gene$diffspliced,
                                                                       truth_gene$diffisopct),
                                                           truth_gene$lengthratio))

cobra <- COBRAData(truth = truth_gene, object_to_extend = cobra)
```

```
plot_perftable(cobradata = cobra, threshold = 0.05, splv = "diffsp_diffisopct_diffbp")
```

```
plot_perftable(cobradata = cobra, threshold = 0.05, splv = "diffsp_diffisopct_rld")
```

```
plot_perftable(cobradata = cobra, threshold = 0.05, splv = "diffsp_diffisopct_lrt")
```

## Help functions

(Back to top)

```
panel_cor <- function(x, y, digits = 3, cex.cor) {
  ## Panel function to print Pearson and Spearman correlations
  usr <- par("usr")
  on.exit(par(usr))
  par(usr = c(0, 1, 0, 1))
  r1 <- abs(cor(x, y, method = "pearson", use = "complete"))
  txt1 <- format(c(r1, 0.123456789), digits = digits)[1]
  r2 <- abs(cor(x, y, method = "spearman", use = "complete"))
  txt2 <- format(c(r2, 0.123456789), digits = digits)[1]
  text(0.5, 0.35, paste("pearson =", txt1), cex = 1.1)
  text(0.5, 0.65, paste("spearman =", txt2), cex = 1.1)
}
```

```
panel_smooth<-function (x, y, col = "blue", bg = NA, pch = ".", 
                        cex = 0.8, ...) {
  ## Panel function to plot points
  points(x, y, pch = pch, col = col, bg = bg, cex = cex)
}
```

```
plot_theme <- function() {
  ## ggplot2 plotting theme
  theme_grey() +
    theme(legend.position = "right",
          panel.background = element_rect(fill = "white", colour = "black"),
          panel.grid.minor.x = element_blank(),
          panel.grid.minor.y = element_blank(),
          strip.text = element_text(size = 10),
          strip.background = element_rect(fill = NA, colour = "black"),
          axis.text.x = element_text(size = 10),
          axis.text.y = element_text(size = 10),
          axis.title.x = element_text(size = 15),
          axis.title.y = element_text(size = 15),
          plot.title = element_text(colour = "black", size = 20))
}
```

```
calc_lengths_mapping <- function(gtf, cdna_fasta, feature_lengths_file,
                                 tx_gene_file) {
  ## Function to calculate gene and transcript lengths from transcript cDNA 
  ## fasta and gtf file. Also generate mapping between transcript and gene IDs.
  
  suppressPackageStartupMessages(library(GenomicFeatures))
  suppressPackageStartupMessages(library(Biostrings))
  
  ## Gene/transcript lengths ===============================================
  ## Transcripts/genes present in gtf file
  if (!is.null(gtf)) {
    txdb <- makeTxDbFromGFF(gtf, format = "gtf")
    ebg <- exonsBy(txdb, "gene")
    ebt <- exonsBy(txdb, "tx", use.names = TRUE)
    ebg_red <- reduce(ebg)
    gene_length <- sum(width(ebg_red))
    ebt_red <- reduce(ebt)
    tx_length <- sum(width(ebt_red))
  } else {
    tx_length <- c()
    gene_length <- c()
  }  
  
  ## Extend with transcripts from cDNA fasta
  cdna <- readDNAStringSet(gsub("\\.gz", "", cdna_fasta))
  tx_length2 <- width(cdna)
  names(tx_length2) <- sapply(names(cdna), function(i) strsplit(i, " ")[[1]][1])
  tx_length2 <- tx_length2[setdiff(names(tx_length2), names(tx_length))]
  tx_length <- c(tx_length, tx_length2)
  
  ## Gene/transcript mapping ===============================================
  ## Transcripts/genes present in gtf file
  if (!is.null(gtf)) {
    tbg <- transcriptsBy(txdb, "gene")
    tx2gene <- stack(lapply(tbg, function(w) w$tx_name))
    colnames(tx2gene) <- c("transcript", "gene")
  } else {
    tx2gene <- data.frame(transcript = c(), gene = c())
  }  
  
  ## Extend with mappings from cDNA fasta file
  tx <- sapply(names(cdna), function(i) strsplit(i, " ")[[1]][1])
  gn <- sapply(names(cdna), function(i) gsub("gene:", "", strsplit(i, " ")[[1]][4]))
  tx2gene2 <- data.frame(transcript = tx, gene = gn, 
                         stringsAsFactors = FALSE)
  rownames(tx2gene2) <- NULL
  tx2gene2 <- tx2gene2[match(setdiff(tx2gene2$transcript, 
                                     tx2gene$transcript), tx2gene2$transcript), ]
  tx2gene <- rbind(tx2gene, tx2gene2)
  
  gene2tx <- tx2gene[, c("gene", "transcript")]
  
  save(gene_length, tx_length, file = feature_lengths_file)
  save(gene2tx, tx2gene, file = tx_gene_file)
}
```

```
diff_expression_edgeR <- function(counts, meta, cond_name, sample_name, 
                                  gene_length_matrix = NULL) {
  ## Differential expression analysis with edgeR
  
  suppressPackageStartupMessages(library(edgeR))

  ## Prepare DGEList
  counts <- round(counts)
  cts <- counts[rowSums(is.na(counts)) == 0, ]
  cts <- cts[rowSums(cts) != 0, ]
  dge <- 
    DGEList(counts = cts, group = meta[, cond_name][match(colnames(cts), 
                                                          meta[, sample_name])])
  
  ## If average transcript lengths provided, add as offset
  if (!is.null(gene_length_matrix)) {
    egf <- gene_length_matrix[match(rownames(cts), rownames(gene_length_matrix)),
                              match(colnames(cts), colnames(gene_length_matrix))]
    egf <- egf / exp(rowMeans(log(egf)))
    o <- log(calcNormFactors(cts/egf)) + log(colSums(cts/egf))
    dge$offset <- t(t(log(egf)) + o)
  } else {
    dge <- calcNormFactors(dge)
  }
  
  ## Estimate dispersions and fit model
  design <- model.matrix(~dge$samples$group)
  dge <- estimateGLMCommonDisp(dge, design = design)
  dge <- estimateGLMTrendedDisp(dge, design = design)
  dge <- estimateGLMTagwiseDisp(dge, design = design)
  fit <- glmFit(dge, design = design)
  lrt <- glmLRT(fit)
  tt <- topTags(lrt, n = Inf)$table
  return(list(dge = dge, tt = tt))
}
```

```
diff_expression_DESeq2 <- function(txi = NULL, counts, meta, cond_name, 
                                   level1, level2, sample_name) {
  ## Differential expression analysis with DESeq2
  
  suppressPackageStartupMessages(library(DESeq2, 
                                         lib.loc = "/home/charlotte/R/x86_64-pc-linux-gnu-library/3.2"))

  ## If tximport object provided, generate DESeqDataSet from it. Otherwise, 
  ## use the provided count matrix.
  if (!is.null(txi)) {
    txi$counts <- round(txi$counts)
    keep_feat <- rownames(txi$counts[rowSums(is.na(txi$counts)) == 0 & rowSums(txi$counts) != 0, ])
    txi <- lapply(txi, function(w) {
      if (!is.null(dim(w))) w[match(keep_feat, rownames(w)), ]
      else w
      })
    dsd <- DESeqDataSetFromTximport(txi, 
                                    colData = meta[match(colnames(txi$counts), 
                                                         meta[, sample_name]), ],
                                    design = as.formula(paste0("~", cond_name)))
  } else {
    counts <- round(counts)
    cts = counts[rowSums(is.na(counts)) == 0, ]
    cts <- cts[rowSums(cts) != 0, ]
    dsd <- DESeqDataSetFromMatrix(countData = round(cts), 
                                  colData = meta[match(colnames(cts), 
                                                       meta[, sample_name]), ],
                                  design = as.formula(paste0("~", cond_name)))
  }
  
  ## Estimate dispersions and fit model
  dsd <- DESeq(dsd, test = "Wald", fitType = "local", betaPrior = TRUE)
  res <- as.data.frame(results(dsd, contrast = c(cond_name, level2, level1),
                               cooksCutoff = FALSE, independentFiltering = FALSE))
  return(list(dsd = dsd, res = res))
}
```

## Session info

(Back to top)

```
sessionInfo()
```

```
## R version 3.2.2 (2015-08-14)
## Platform: x86_64-pc-linux-gnu (64-bit)
## Running under: Ubuntu 14.04.3 LTS
## 
## locale:
##  [1] LC_CTYPE=C                 LC_NUMERIC=C               LC_TIME=en_CA.UTF-8       
##  [4] LC_COLLATE=en_CA.UTF-8     LC_MONETARY=en_CA.UTF-8    LC_MESSAGES=en_CA.UTF-8   
##  [7] LC_PAPER=en_CA.UTF-8       LC_NAME=C                  LC_ADDRESS=C              
## [10] LC_TELEPHONE=C             LC_MEASUREMENT=en_CA.UTF-8 LC_IDENTIFICATION=C       
## 
## attached base packages:
## [1] parallel  stats4    stats     graphics  grDevices utils     datasets  methods   base     
## 
## other attached packages:
##  [1] edgeR_3.12.0               limma_3.26.3               DESeq2_1.11.6             
##  [4] RcppArmadillo_0.6.400.2.2  Rcpp_0.12.2                SummarizedExperiment_1.0.2
##  [7] Biobase_2.30.0             GenomicRanges_1.22.3       GenomeInfoDb_1.6.2        
## [10] IRanges_2.4.6              S4Vectors_0.8.6            BiocGenerics_0.16.1       
## [13] Hmisc_3.17-1               Formula_1.2-1              survival_2.38-3           
## [16] lattice_0.20-33            reshape2_1.4.1             iCOBRA_0.99.4             
## [19] ggplot2_2.0.0              Rsubread_1.20.2            tximport_0.0.7            
## [22] dplyr_0.4.3                biomaRt_2.26.1            
## 
## loaded via a namespace (and not attached):
##  [1] bitops_1.0-6         RColorBrewer_1.1-2   tools_3.2.2          R6_2.1.1            
##  [5] DT_0.1               rpart_4.1-10         KernSmooth_2.23-15   DBI_0.3.1           
##  [9] lazyeval_0.1.10      colorspace_1.2-6     nnet_7.3-11          gridExtra_2.0.0     
## [13] formatR_1.2.1        labeling_0.3         caTools_1.17.1       scales_0.3.0        
## [17] genefilter_1.52.0    stringr_1.0.0        digest_0.6.9         Rsamtools_1.22.0    
## [21] shinyBS_0.61         foreign_0.8-66       rmarkdown_0.9.2      XVector_0.10.0      
## [25] htmltools_0.3        htmlwidgets_0.5      RSQLite_1.0.0        shiny_0.13.0        
## [29] DEXSeq_1.16.7        hwriter_1.3.2        BiocParallel_1.4.3   gtools_3.5.0        
## [33] acepack_1.3-3.3      RCurl_1.95-4.7       magrittr_1.5         futile.logger_1.4.1 
## [37] munsell_0.4.2        stringi_1.0-1        yaml_2.1.13          zlibbioc_1.16.0     
## [41] gplots_2.17.0        plyr_1.8.3           grid_3.2.2           gdata_2.17.0        
## [45] shinydashboard_0.5.1 Biostrings_2.38.3    splines_3.2.2        annotate_1.48.0     
## [49] locfit_1.5-9.1       knitr_1.12.3         geneplotter_1.48.0   futile.options_1.0.0
## [53] XML_3.98-1.3         evaluate_0.8         latticeExtra_0.6-26  lambda.r_1.1.7      
## [57] httpuv_1.3.3         gtable_0.1.2         assertthat_0.1       mime_0.4            
## [61] xtable_1.8-0         AnnotationDbi_1.32.3 cluster_2.0.3        statmod_1.4.23      
## [65] ROCR_1.0-7
```
