# Supplementary material for: Differential analyses for RNA-seq: transcript-level estimates improve gene-level inferences
Source: F1000Res. 2016 Feb 29;4:1521. Originally published 2015 Dec 30. [Version 2] doi: 10.12688/f1000research.7563.2 (PMC4712774; doi:10.12688/f1000research.7563.2)
Supplement: Data set 2 — http://dx.doi.org/10.5256/f1000research.7563.d114723 Data set 2 (html) contains all the R code that was used to perform the analyses and generate the figures for the sim2 data set 31. [file f1000research-4-8770-s0001.tgz › 4197990b-04cf-48d9-832d-6a18cbd575af_sim2-quantification.html]

sim2


# sim2

- Preparation
  - Reference directories and packages
  - Reference file preparation
    - Reference file subsetting
    - Generation of incomplete annotation
    - Preparation of RSEM files for simulation
    - Generation of STAR index for alignment
  - Simulation
  - Metadata definition
  - Definition of paths to reference files
  - Definition of paths to generated files
  - Definition of gene-to-transcript mapping
  - Summarization of Salmon results and offset estimation
    - Complete annotation
    - Incomplete annotation
    - Estimation of coefficients of variation for all samples
  - Generation of exon bin counts with DEXSeq
- Accuracy of abundance estimates
  - All features
  - Only features with positive true and estimated TPMs
  - All features, after replacing missing estimates with 0
  - Consider groups of paralogous genes only
- Variability of transcript and gene TPMs
- Correlation between gene count estimates
  - All genes
  - Only genes with positive estimates with all methods
  - Example of genes with a big difference between simple sum and scaled TPM
- Differential transcript expression (DTE) analysis on gene and transcript level
  - Comparison of DTE performance on gene and transcript level
    - Exploration of power difference between gene- and transcript-level analyses
  - Evaluation of logFC estimation accuracy (transcripts)
    - All transcripts
    - Only transcripts with true logFC != 0 and true logFC != Inf
- Differential gene expression analysis (edgeR)
  - Diagnostics
  - Comparison of significant genes found with different matrices
  - Comparison to truth
  - Comparison of logFC estimates
    - All methods
    - simplesum vs scaledTPM
  - Evaluation of logFC estimation accuracy (genes)
    - All genes
    - All genes, stratified by presence of differential isoform usage
    - Only genes with true logFC != 0
    - Only genes with true logFC != 0, stratified by presence of differential isoform usage
- Comparison of DTE analysis aggregated on gene level to DGE
- DTU analysis with DEXSeq
  - Application of DEXSeq to Salmon transcript counts
  - DTU analysis on exon bin counts, with DEXSeq
  - Comparison to truth
- Comparison of significant genes from DTE, DTU, DGE
- Differential gene expression analysis (DESeq2)
  - Diagnostics
  - Comparison of significant genes found with different matrices
  - Comparison to truth
  - Comparison of logFC estimates
    - All methods
    - simplesum vs scaledTPM
  - Evaluation of logFC estimation accuracy (genes)
    - All genes
    - All genes, stratified by presence of differential isoform usage
    - Only genes with true logFC != 0
    - Only genes with true logFC != 0, stratified by presence of differential isoform usage
- Differential gene expression analysis (limma-voom)
  - Comparison of significant genes found with different matrices
  - Comparison to truth
- Help functions
- Session info

The sim2 data set consists of synthetic human, paired-end, 100bp reads from two conditions, each with three samples. The basis for the simulation is the human chromosome 1 from Ensembl GRCh37.71. We introduce differential gene expression, differential transcript expression and differential isoform usage.

## Preparation

(Back to top)

### Reference directories and packages

(Back to top)

```
basedir <- "/home/charlotte/gene_vs_tx_quantification"
suppressPackageStartupMessages(library(limma))
suppressPackageStartupMessages(library(edgeR))
suppressPackageStartupMessages(library(dplyr))
suppressPackageStartupMessages(library(tximport))
suppressPackageStartupMessages(library(ggplot2, 
                                       lib.loc = "/home/charlotte/R/x86_64-pc-linux-gnu-library/3.2"))
suppressPackageStartupMessages(library(Hmisc))
suppressPackageStartupMessages(library(iCOBRA, 
                                       lib.loc = "/home/charlotte/R/x86_64-pc-linux-gnu-library/3.2"))
suppressPackageStartupMessages(library(BiocParallel))
suppressPackageStartupMessages(library(DESeq2, lib.loc = "/home/charlotte/R/x86_64-pc-linux-gnu-library/3.2"))
```

```
## Warning: replacing previous import by 'ggplot2::Position' when loading 'DESeq2'
```

```
suppressPackageStartupMessages(library(DEXSeq))
suppressPackageStartupMessages(library(biomaRt))

sumna <- function(w) {
  if (all(is.na(w))) NA
  else sum(w, na.rm = TRUE)
}

muted <- c("#DC050C","#E8601C","#7BAFDE","#1965B0","#B17BA6",
           "#882E72","#F1932D","#F6C141","#F7EE55","#4EB265",
           "#90C987","#CAEDAB","#777777")
```

### Reference file preparation

(Back to top)

Since the simulation is based on chromosome 1 only, we use the following code to subset annotations (downloaded from Ensembl, GRCh37.71) to only this region, as well as to prepare index files for RSEM and STAR. We also generate an incomplete reference catalog, by removing 20% (randomly selected) of the transcripts from the annotation.

#### Reference file subsetting

(Back to top)

```
## Extract only transcripts from chromosome 1 from the transcript fasta file
## Also, generate a transcript-to-gene mapping

library(Biostrings)

cdna <- readDNAStringSet("wholegenome/Homo_sapiens.GRCh37.71.cdna.all.fa", 
                         format = "fasta")
cdna.chr1 <- cdna[grep("GRCh37:1:", names(cdna))]
writeXStringSet(cdna.chr1, filepath = "chr1/Homo_sapiens.GRCh37.71.cdna.chr1.fa",
                format = "fasta")

tx.to.gene <- t(sapply(names(cdna), function(s) {
  s <- strsplit(s, " ")[[1]]
  tx <- s[grep("^ENST", s)]
  gn <- gsub("gene:", "", s[grep("gene:ENSG", s)])
  c(gn, tx)
}))
rownames(tx.to.gene) <- NULL

write.table(tx.to.gene, file = "wholegenome/tx_to_gene.txt", 
            col.names = FALSE, row.names = FALSE, sep = "\t", quote = FALSE)

tx.to.gene.chr1 <- t(sapply(names(cdna.chr1), function(s) {
  s <- strsplit(s, " ")[[1]]
  tx <- s[grep("^ENST", s)]
  gn <- gsub("gene:", "", s[grep("gene:ENSG", s)])
  c(gn, tx)
}))
rownames(tx.to.gene.chr1) <- NULL

write.table(tx.to.gene.chr1, file = "chr1/tx_to_gene_chr1.txt", 
            col.names = FALSE, row.names = FALSE, sep = "\t", quote = FALSE)

## Subset GTF file
library(rtracklayer)
gtff <- import("wholegenome/Homo_sapiens.GRCh37.71.gtf")
gtff2 <- subset(gtff, seqnames == "1")
export(gtff2, "chr1/Homo_sapiens.GRCh37.71.chr1.gtf", format = "gtf")
```

#### Generation of incomplete annotation

(Back to top)

```
## Determine which transcripts to exclude
library(Biostrings)
cdna <- readDNAStringSet("chr1/Homo_sapiens.GRCh37.71.cdna.chr1.fa")
tx <- sapply(strsplit(names(cdna), " "), .subset, 1)
set.seed(1)
keep_tx <- sample(tx, size = 0.8*length(tx))
excl_tx <- setdiff(tx, keep_tx)

## Generate new cDNA fasta
writeXStringSet(cdna[match(keep_tx, tx)], 
                file = "chr1_incomplete/Homo_sapiens.GRCh37.71.cdna.chr1_incomplete80.fa")

## Read and generate new GTF file
library(rtracklayer)
gtf <- import("chr1/Homo_sapiens.GRCh37.71.chr1.gtf")
gtf <- subset(gtf, transcript_id %in% keep_tx)
export(gtf, con = "chr1_incomplete/Homo_sapiens.GRCh37.71.chr1_incomplete80.gtf")

## Generate kallisto index
if (!file.exists("chr1_incomplete/kallisto_index/Homo_sapiens.GRCh37.71.cdna.chr1_incomplete80.idx")) {
  cmd <- "kallisto index -i chr1_incomplete/kallisto_index/Homo_sapiens.GRCh37.71.cdna.chr1_incomplete80.idx chr1_incomplete/Homo_sapiens.GRCh37.71.cdna.chr1_incomplete80.fa"
  cat(cmd, "\n")
  system(cmd)
}

if (!file.exists("chr1_incomplete/STAR_index/SA")) {
  cmd <- "STAR --runMode genomeGenerate --runThreadN 10 --genomeDir chr1_incomplete/STAR_index --genomeFastaFiles chr1/Homo_sapiens.GRCh37.71.dna.chromosome.1.fa --sjdbGTFfile chr1_incomplete/Homo_sapiens.GRCh37.71.chr1_incomplete80.gtf --sjdbOverhang 99"
  message(cmd)
  system(cmd)
}
```

#### Preparation of RSEM files for simulation

(Back to top)

```
RSEM=/home/Shared/data/alt_splicing_simulations/Simulation5_Charlotte/software/rsem-1.2.21

## Prepare the reference files for RSEM
$RSEM/rsem-prepare-reference --bowtie2 \
--transcript-to-gene-map chr1/tx_to_gene_chr1.txt \
chr1/Homo_sapiens.GRCh37.71.cdna.chr1.fa \
chr1/RSEM_index/Homo_sapiens.GRCh37.71.cdna.chr1

## Estimate the model file with RSEM
$RSEM/rsem-calculate-expression \
--paired-end --bowtie2 --seed 123 \
/home/charlotte/dte_simulation/exp_data/E_MTAB_1733/FASTQ/ERS326990_1.fastq.gz \
/home/charlotte/dte_simulation/exp_data/E_MTAB_1733/FASTQ/ERS326990_2.fastq.gz \
chr1/RSEM_index/Homo_sapiens.GRCh37.71.cdna.chr1 \
/home/charlotte/dte_simulation/exp_data/E_MTAB_1733/RSEM/ERS326990/chr1/ERS326990_chr1
```

#### Generation of STAR index for alignment

(Back to top)

```
STAR --runMode genomeGenerate --runThreadN 10 --genomeDir /home/Shared/data/alt_splicing_simulations/dte_simulation/annotations/chr1/STAR_index --genomeFastaFiles /home/Shared/data/alt_splicing_simulations/dte_simulation/annotations/chr1/Homo_sapiens.GRCh37.71.dna.chromosome.1.fa --sjdbGTFfile /home/Shared/data/alt_splicing_simulations/dte_simulation/annotations/chr1/Homo_sapiens.GRCh37.71.chr1.gtf --sjdbOverhang 99
```

### Simulation

(Back to top)

Here, we reproduce the code used for generation of the simulated data, alignment, counting and isoform quantification.

```
library(dplyr)
library(Hmisc)

###############################################################################
## Preparation
###############################################################################
## Get sample to use as basis
s0 <- read.delim("../exp_data/E_MTAB_1733/kallisto/ERS326990/abundance.tsv", 
                 header = TRUE, as.is = TRUE)

## Add gene information
tx_to_gene <- read.delim("../annotations/wholegenome/tx_to_gene.txt", 
                         header = FALSE, as.is = TRUE)
colnames(tx_to_gene) <- c("gene", "transcript")
s0$gene <- tx_to_gene$gene[match(s0$target_id, tx_to_gene$transcript)]

## Calculate the "conversion factor" between TPM and counts. To get the 
## count, take TPM * eff_length * conv_factor
conv_factor <- mean(s0$est_counts/s0$tpm/s0$eff_length, na.rm = TRUE)

## Calculate isoform percentages
s0 <- as.data.frame(s0 %>% group_by(gene) %>% 
                      mutate(isopct = tpm/sum(tpm)) %>% ungroup())
s0$isopct[is.na(s0$isopct)] <- 0

## Generate collection of isoform percentages for genes with 
## given number of isoforms
tmp <- split(s0, s0$gene)
isopcts <- lapply(tmp, function(x) x$isopct)
sums <- sapply(isopcts, sum)
isopcts <- isopcts[sums > 0]
L <- sapply(isopcts, length)
isopcts2 <- split(isopcts, L)
isopcts2 <- lapply(isopcts2, function(y) do.call(rbind, y))

## Summarise data per gene (expression, number of isoforms)
s0.gene <- as.data.frame(s0 %>% group_by(gene) %>% 
                           summarise(tpm = sum(tpm), 
                                     nbr_isoforms = length(target_id)))
s0.gene$eligible <- 1
s0.gene$eligible[s0.gene$tpm < 0.1] <- 0
s0.gene$eligible[s0.gene$tpm > 1000] <- 0

## Only change genes on chromosome 1
chr1.genes <- unique(read.delim("../annotations/chr1/tx_to_gene_chr1.txt", 
                                header = FALSE, as.is = TRUE)[, 1])
s0.gene$eligible[!s0.gene$gene %in% chr1.genes] <- 0
n.genes <- length(chr1.genes)

###############################################################################
## Generate second basis sample (condition 2)
###############################################################################
set.seed(42)

## Generate basis sample for second condition by modifying tpms
s1 <- s0

## Select genes to modify
## DTU: choose among all eligible genes with at least 2 isoforms
dtu <- sample(s0.gene$gene[s0.gene$eligible == 1 & s0.gene$nbr_isoforms > 1], 
              0.109*n.genes)
s0.gene[match(dtu, s0.gene$gene), "eligible"] <- 0

## DTE: choose among all eligible genes with at least 2 isoforms
dte <- sample(s0.gene$gene[s0.gene$eligible == 1 & s0.gene$nbr_isoforms > 1], 
              0.1095*n.genes)
s0.gene[match(dte, s0.gene$gene), "eligible"] <- 0

## DGE: choose among all eligible genes
dge <- sample(s0.gene$gene[s0.gene$eligible == 1], 0.109*n.genes)
s0.gene[match(dge, s0.gene$gene), "eligible"] <- 0

s1l <- split(s1, s1$gene)

## Get all TPMs of genes with DGE and shuffle them
## Shuffling will be done only between neighbouring "bins", to avoid too 
## large fold changes
tpms_dge <- s0.gene[match(dge, s0.gene$gene), "tpm"]
names(tpms_dge) <- dge
tpms_dge <- sort(tpms_dge)
tmp_names <- names(tpms_dge)
tpms_dge1 <- tpms_dge

tpms_dge01 <- tpms_dge[1:(length(tpms_dge)/4)]
tpms_dge02 <- tpms_dge[(length(tpms_dge)/4 + 1):(2*length(tpms_dge)/4)]
tpms_dge03 <- tpms_dge[(2*length(tpms_dge)/4 + 1):(3*length(tpms_dge)/4)]
tpms_dge04 <- tpms_dge[(3*length(tpms_dge)/4 + 1):(4*length(tpms_dge)/4)]
while(max(abs(log2(tpms_dge/tpms_dge1))) > log2(256)  | 
      min(abs(log2(tpms_dge/tpms_dge1))) < 0.55) {
  tpms_dge <- c(sample(tpms_dge02), sample(tpms_dge01),
                sample(tpms_dge04), sample(tpms_dge03))
}
names(tpms_dge) <- tmp_names

## Generate list of all transcripts with DTE
dte_tx <- unlist(lapply(dte, function(g) {
  a <- s1l[[g]]
  i <- which(a$tpm > 0)[sample(length(which(a$tpm > 0)), 
                               min(ceiling(0.1*nrow(a)),
                                   length(which(a$tpm > 0))))]
  a[i, "target_id"]
}))
## Get all TPMs of transcripts with DTE and shuffle them
nbins <- 4
npbin <- length(dte_tx)/nbins
stopifnot(npbin == round(npbin))
idx <- (rep(c((npbin + 1):(2*npbin), 1:npbin), nbins/2)) + 
  rep(2*npbin*(0:(nbins/2 - 1)), each = 2*npbin)
tpms_dte <- s0[match(dte_tx, s0$target_id), "tpm"]
names(tpms_dte) <- dte_tx
tpms_dte <- sort(tpms_dte)
tmp_names <- names(tpms_dte)
tpms_dte <- tpms_dte[idx]
names(tpms_dte) <- tmp_names

## Modify TPM and count info
## DTU
for (g in dtu) {
  a <- s1l[[g]]
  n <- nrow(a)
  isfd <- isopcts2[[as.character(n)]]
  isfd <- sample(isfd[sample(1:nrow(isfd), 1), ])
  tpm <- sum(a$tpm)
  a$isopct <- isfd
  a$tpm <- tpm*a$isopct
  a$est_counts <- a$tpm * a$eff_length * conv_factor
  s1l[[g]] <- a
}

## DTE
for (g in dte) {
  a <- s1l[[g]]
  kp <- intersect(a$target_id, names(tpms_dte))
  a[match(kp, a$target_id), "tpm"] <- tpms_dte[kp]
  a$isopct <- a$tpm/sum(a$tpm)
  a$est_counts <- a$tpm * a$eff_length * conv_factor
  s1l[[g]] <- a
}

## DGE
for (g in dge) {
  a <- s1l[[g]]
  a$tpm <- tpms_dge[g] * a$isopct
  a$est_counts <- a$tpm * a$eff_length * conv_factor
  s1l[[g]] <- a
}

s1 <- unsplit(s1l, s1$gene)

###############################################################################
## Generate individual samples (add variability)
###############################################################################

n_per_group <- 3

stopifnot(all(s0$target_id == s1$target_id))
mp <- readRDS("../annotations/Pickrell.Cheung.Mu.Phi.Estimates.rds")

gr1 <- t(sapply(s0$target_id, function(i) {
  m <- s0$est_counts[match(i, s0$target_id)]
  phi <- mp$pickrell.cheung.phi[which.min(abs(mp$pickrell.cheung.mu - m))]
  rnbinom(n = n_per_group, size = 1/phi, mu = m)
}))
colnames(gr1) <- paste0("A", 1:ncol(gr1))

gr2 <- t(sapply(s1$target_id, function(i) {
  m <- s1$est_counts[match(i, s1$target_id)]
  phi <- mp$pickrell.cheung.phi[which.min(abs(mp$pickrell.cheung.mu - m))]
  rnbinom(n = n_per_group, size = 1/phi, mu = m)
}))
colnames(gr2) <- paste0("B", 1:ncol(gr2))

gr1_tpm <- sweep(gr1, 1, s0$eff_length * conv_factor, "/")
gr2_tpm <- sweep(gr2, 1, s1$eff_length * conv_factor, "/")

gr1_tpm <- sweep(gr1_tpm, 2, colSums(gr1_tpm)/1e6, "/")
gr2_tpm <- sweep(gr2_tpm, 2, colSums(gr2_tpm)/1e6, "/")

all_tpm <- cbind(gr1_tpm, gr2_tpm)

###############################################################################
## Write files for RSEM
###############################################################################
rsem_template_chr1 <- 
  read.delim("../exp_data/E_MTAB_1733/RSEM/ERS326990/chr1/ERS326990_chr1.isoforms.results", 
             header = TRUE, as.is = TRUE)
for (i in 1:ncol(all_tpm)) {
  tmp <- rsem_template_chr1
  tmp$TPM <- all_tpm[match(tmp$transcript_id, rownames(all_tpm)), i]
  tmp$TPM <- tmp$TPM/sum(tmp$TPM)*1e6
  tmp$TPM <- round(tmp$TPM, digits = 2)
  tmp$TPM <- as.character(tmp$TPM)
  tmp$TPM[tmp$TPM == "0"] <- "0.00"
  tmp$expected_count <- as.character(tmp$expected_count)
  tmp$expected_count[tmp$expected_count == "0"] <- "0.00"
  tmp$FPKM <- as.character(tmp$FPKM)
  tmp$FPKM[tmp$FPKM == "0"] <- "0.00"
  tmp$IsoPct <- as.character(tmp$IsoPct)
  tmp$IsoPct[tmp$IsoPct == "0"] <- "0.00"
  write.table(tmp, file = paste0("sim2/chr1/RSEM/sample", 
                                 colnames(all_tpm)[i], "_rsem.txt"), 
              row.names = FALSE, col.names = TRUE, quote = FALSE, sep = "\t")
}

###############################################################################
## Generate truth on gene and transcript level
###############################################################################
## Transcripts
stopifnot(all(s0$target_id == s1$target_id))
truth_tx <- s0
truth_tx$isopct1 <- truth_tx$isopct
truth_tx$isopct <- NULL
truth_tx$isopct2 <- s1$isopct
truth_tx$tpm1 <- truth_tx$tpm
truth_tx$tpm <- NULL
truth_tx$tpm2 <- s1$tpm
truth_tx$status <- 0
truth_tx$status[which(truth_tx$tpm1 != truth_tx$tpm2)] <- 1
truth_tx$logFC <- 0
truth_tx$logFC[which(truth_tx$status == 1)] <- 
  abs(log2(truth_tx$tpm1[which(truth_tx$status == 1)]/
             truth_tx$tpm2[which(truth_tx$status == 1)]))
truth_tx$logFC_cat <- cut2(truth_tx$logFC, 
                           c(0, quantile(truth_tx$logFC[truth_tx$logFC > 0 & is.finite(truth_tx$logFC)],
                                         probs = c(0.25, 0.5, 0.75, 1)), Inf))
truth_tx$avetpm <- apply(truth_tx[, c("tpm1", "tpm2")], 1, mean)
t3 <- truth_tx[, c("target_id", "status", "logFC_cat", "logFC", "avetpm", 
                   "length", "eff_length", "tpm1", "tpm2", "isopct1", "isopct2", "gene")]
colnames(t3) <- c("transcript", "status", "logFC_cat", "logFC", "avetpm", 
                  "length", "eff_length", "tpm1", "tpm2", "isopct1", "isopct2", "gene")

## Subset to chr1
chr1_tx <- rsem_template_chr1$transcript_id
t3t <- t3[match(chr1_tx, t3$transcript), ]
t3t$avetpm_cat <- cut2(t3t$avetpm, 
                       c(0, quantile(t3t$avetpm[t3t$avetpm > 0], 
                                     probs = c(0.25, 0.5, 0.75, 1))))
write.table(t3t, file = "sim2/chr1/truth_transcript.txt", row.names = FALSE,
            col.names = TRUE, quote = FALSE, sep = "\t")

## Genes
t3g <- as.data.frame(t3t %>% group_by(gene) %>% 
                       summarise(tpm1 = sum(tpm1),
                                 tpm2 = sum(tpm2),
                                 status_any = as.numeric(any(status == 1)),
                                 n_isoforms = length(transcript),
                                 diffisouse = as.numeric(any(isopct1 != isopct2))))
t3g$status_overall <- 0
t3g$status_overall[match(c(dge, dte), t3g$gene)] <- 1
t3g$logFC_overall <- 0
t3g$logFC_overall[match(c(dge, dte), t3g$gene)] <- 
  abs(log2(t3g$tpm1[match(c(dge, dte), t3g$gene)]/
             t3g$tpm2[match(c(dge, dte), t3g$gene)]))
t3g$dtu <- 0
t3g$dte <- 0
t3g$dge <- 0
t3g$dtu[match(dtu, t3g$gene)] <- 1
t3g$dte[match(dte, t3g$gene)] <- 1
t3g$dge[match(dge, t3g$gene)] <- 1
t3g$logFC_overall_cat <- 
  cut2(t3g$logFC_overall, 
       c(0, quantile(t3g$logFC_overall[t3g$logFC_overall > 0],
                     probs = c(0.25, 0.5, 0.75, 1))))
t3g$avetpm <- apply(t3g[, c("tpm1", "tpm2")], 1, mean)
t3g$avetpm_cat <- 
  cut2(t3g$avetpm, 
       c(0, quantile(t3g$avetpm[t3g$avetpm > 0], 
                     probs = c(0.25, 0.5, 0.75, 1))))
write.table(t3g, file = "sim2/chr1/truth_gene.txt", row.names = FALSE,
            col.names = TRUE, quote = FALSE, sep = "\t")

###############################################################################
## Simulate FASTQ files
###############################################################################
## chr1
for (i in c(paste0("A", 1:n_per_group), paste0("B", 1:n_per_group))) {
  cmd <- paste0("/home/Shared/data/alt_splicing_simulations/Simulation5_Charlotte/software/rsem-1.2.21/rsem-simulate-reads /home/Shared/data/alt_splicing_simulations/dte_simulation/annotations/chr1/RSEM_index/Homo_sapiens.GRCh37.71.cdna.chr1 ../exp_data/E_MTAB_1733/RSEM/ERS326990/chr1/ERS326990_chr1.stat/ERS326990_chr1.HQ.model sim2/chr1/RSEM/sample", i, "_rsem.txt 0 10000000 sim2/chr1/FASTQ/sample", i, "/sample", i, " --seed 123")
  cat(cmd, "\n")
  system(cmd)
}

###############################################################################
## Run kallisto on simulated FASTQ files
###############################################################################
## chr1
if (!file.exists("../annotations/chr1/kallisto_index/Homo_sapiens.GRCh37.71.cdna.chr1.idx")) {
  cmd <- "kallisto index -i ../annotations/chr1/kallisto_index/Homo_sapiens.GRCh37.71.cdna.chr1.idx ../annotations/chr1/Homo_sapiens.GRCh37.71.cdna.chr1.fa"
  cat(cmd, "\n")
  system(cmd)
}

for (i in c(paste0("A", 1:n_per_group), paste0("B", 1:n_per_group))) {
  cmd <- paste0("kallisto quant -i ../annotations/chr1/kallisto_index/Homo_sapiens.GRCh37.71.cdna.chr1.idx -o sim2/chr1/kallisto/sample", i, " -b 30 sim2/chr1/FASTQ/sample", i, "/sample", i, "_1.fq sim2/chr1/FASTQ/sample", i, "/sample", i, "_2.fq")
  cat(cmd, "\n")
  system(cmd)
}

###############################################################################
## Run salmon on simulated FASTQ files
###############################################################################
## chr1
if (!file.exists("../annotations/chr1/salmon_index/Homo_sapiens.GRCh37.71.cdna.chr1.sidx/hash.bin")) {
  cmd <- "salmon index -i ../annotations/chr1/salmon_index/Homo_sapiens.GRCh37.71.cdna.chr1.sidx -t ../annotations/chr1/Homo_sapiens.GRCh37.71.cdna.chr1.fa -p 5 --type quasi"
  cat(cmd, "\n")
  system(cmd)
}

## Swap order of genes and transcripts in tx_to_gene_chr1.txt
cmd <- "awk '{ t=$1 ; $1=$2; $2=t; print }' ../annotations/chr1/tx_to_gene_chr1.txt > ../annotations/chr1/tx_to_gene_chr1_swapped.txt"
cat(cmd, "\n")
system(cmd)

for (i in c(paste0("A", 1:n_per_group), paste0("B", 1:n_per_group))) {
  cmd <- paste0("salmon quant -i ../annotations/chr1/salmon_index/Homo_sapiens.GRCh37.71.cdna.chr1.sidx -l IU -1 sim2/chr1/FASTQ/sample", i, "/sample", i, "_1.fq -2 sim2/chr1/FASTQ/sample", i, "/sample", i, "_2.fq -p 5 -o sim2/chr1/salmon/sample", i, " -g ../annotations/chr1/tx_to_gene_chr1_swapped.txt --numBootstraps=30")
  cat(cmd, "\n")
  system(cmd)
}

###############################################################################
## Align to genome
###############################################################################
## chr1
fqdir <- list.files("sim2/chr1/FASTQ", full.names = TRUE)
fqdir <- fqdir[file.info(fqdir)$isdir]
for (fq in fqdir) {
  cmd <- paste0("STAR --genomeDir ../annotations/chr1/STAR_index --readFilesIn ",
                paste0(fq, "/", basename(fq), "_1.fq"), " ", 
                paste0(fq, "/", basename(fq), "_2.fq"), " --runThreadN 10 ",
                "--outFileNamePrefix sim2/chr1/BAM/", basename(fq), "/", 
                " --outSAMtype BAM Unsorted")
  cat(cmd, "\n")
  system(cmd)
}

###############################################################################
## Run featureCounts
###############################################################################
## chr1
library(Rsubread)
bamdir <- list.files("sim2/chr1/BAM", full.names = TRUE)
bamdir <- bamdir[file.info(bamdir)$isdir]
bamfiles <- paste0(bamdir, "/Aligned.out.bam")
names(bamfiles) <- basename(bamdir)
fc <- featureCounts(files = bamfiles, isGTFAnnotationFile = TRUE, 
                    annot.ext = "../annotations/chr1/Homo_sapiens.GRCh37.71.chr1.gtf",
                    GTF.featureType = "exon", 
                    GTF.attrType = "gene_id", 
                    useMetaFeatures = TRUE,
                    isPairedEnd = TRUE)
colnames(fc$counts) <- sapply(colnames(fc$counts), function(w) {
  tmp <- strsplit(w, "\\.")[[1]]
  tmp[grep("sample", tmp)]
})
save(fc, file = "sim2/chr1/featureCounts/fc.Rdata")

###############################################################################
## Run featureCounts, including multimappers
###############################################################################
## chr1
library(Rsubread)
bamdir <- list.files("sim2/chr1/BAM", full.names = TRUE)
bamdir <- bamdir[file.info(bamdir)$isdir]
bamfiles <- paste0(bamdir, "/Aligned.out.bam")
names(bamfiles) <- basename(bamdir)
fc_fracmm <- featureCounts(files = bamfiles, isGTFAnnotationFile = TRUE, 
                           annot.ext = "../annotations/chr1/Homo_sapiens.GRCh37.71.chr1.gtf",
                           GTF.featureType = "exon", 
                           GTF.attrType = "gene_id", 
                           useMetaFeatures = TRUE,
                           isPairedEnd = TRUE,
                           countMultiMappingReads = TRUE,
                           fraction = TRUE)
colnames(fc_fracmm$counts) <- sapply(colnames(fc_fracmm$counts), function(w) {
  tmp <- strsplit(w, "\\.")[[1]]
  tmp[grep("sample", tmp)]
})
save(fc_fracmm, file = "sim2/chr1/featureCounts/fc_fracMM.Rdata")

###############################################################################
###############################################################################
## INCOMPLETE ANNOTATION
###############################################################################
###############################################################################

###############################################################################
## Run kallisto on simulated FASTQ files
###############################################################################
## chr1_incomplete
if (!file.exists("../annotations/chr1_incomplete/kallisto_index/Homo_sapiens.GRCh37.71.cdna.chr1_incomplete80.idx")) {
  cmd <- "kallisto index -i ../annotations/chr1_incomplete/kallisto_index/Homo_sapiens.GRCh37.71.cdna.chr1_incomplete80.idx ../annotations/chr1_incomplete/Homo_sapiens.GRCh37.71.cdna.chr1_incomplete80.fa"
  cat(cmd, "\n")
  system(cmd)
}

for (i in c(paste0("A", 1:n_per_group), paste0("B", 1:n_per_group))) {
  cmd <- paste0("kallisto quant -i ../annotations/chr1_incomplete/kallisto_index/Homo_sapiens.GRCh37.71.cdna.chr1_incomplete80.idx -o sim2/chr1_incomplete/kallisto/sample", i, " -b 30 sim2/chr1/FASTQ/sample", i, "/sample", i, "_1.fq sim2/chr1/FASTQ/sample", i, "/sample", i, "_2.fq")
  cat(cmd, "\n")
  system(cmd)
}

###############################################################################
## Run salmon on simulated FASTQ files
###############################################################################
## chr1_incomplete
if (!file.exists("../annotations/chr1_incomplete/salmon_index/Homo_sapiens.GRCh37.71.cdna.chr1_incomplete80.sidx/hash.bin")) {
  cmd <- "salmon index -i ../annotations/chr1_incomplete/salmon_index/Homo_sapiens.GRCh37.71.cdna.chr1_incomplete80.sidx -t ../annotations/chr1_incomplete/Homo_sapiens.GRCh37.71.cdna.chr1_incomplete80.fa -p 5 --type quasi"
  cat(cmd, "\n")
  system(cmd)
}

for (i in c(paste0("A", 1:n_per_group), paste0("B", 1:n_per_group))) {
  cmd <- paste0("salmon quant -i ../annotations/chr1_incomplete/salmon_index/Homo_sapiens.GRCh37.71.cdna.chr1_incomplete80.sidx -l IU -1 sim2/chr1/FASTQ/sample", i, "/sample", i, "_1.fq -2 sim2/chr1/FASTQ/sample", i, "/sample", i, "_2.fq -p 5 -o sim2/chr1_incomplete/salmon/sample", i, " -g ../annotations/chr1/tx_to_gene_chr1_swapped.txt --numBootstraps=30")
  cat(cmd, "\n")
  system(cmd)
}

###############################################################################
## Align to genome
###############################################################################
## chr1_incomplete
fqdir <- list.files("sim2/chr1/FASTQ", full.names = TRUE)
fqdir <- fqdir[file.info(fqdir)$isdir]
for (fq in fqdir) {
  cmd <- paste0("STAR --genomeDir ../annotations/chr1_incomplete/STAR_index --readFilesIn ",
                paste0(fq, "/", basename(fq), "_1.fq"), " ", 
                paste0(fq, "/", basename(fq), "_2.fq"), " --runThreadN 10 ",
                "--outFileNamePrefix sim2/chr1_incomplete/BAM/", basename(fq), "/", 
                " --outSAMtype BAM Unsorted")
  cat(cmd, "\n")
  system(cmd)
}

###############################################################################
## Run featureCounts
###############################################################################
## chr1
library(Rsubread)
bamdir <- list.files("sim2/chr1_incomplete/BAM", full.names = TRUE)
bamdir <- bamdir[file.info(bamdir)$isdir]
bamfiles <- paste0(bamdir, "/Aligned.out.bam")
names(bamfiles) <- basename(bamdir)
fc_incomplete <- featureCounts(files = bamfiles, isGTFAnnotationFile = TRUE, 
                               annot.ext = "../annotations/chr1_incomplete/Homo_sapiens.GRCh37.71.chr1_incomplete80.gtf",
                               GTF.featureType = "exon", 
                               GTF.attrType = "gene_id", 
                               useMetaFeatures = TRUE,
                               isPairedEnd = TRUE)
colnames(fc_incomplete$counts) <- sapply(colnames(fc_incomplete$counts), function(w) {
  tmp <- strsplit(w, "\\.")[[1]]
  tmp[grep("sample", tmp)]
})
save(fc_incomplete, file = "sim2/chr1_incomplete/featureCounts/fc_incomplete.Rdata")
```

### Metadata definition

(Back to top)

The samples are named according to the condition they belong to (“A” or “B”). We define a metadata table mapping samples to the correct condition, to be used for differential analysis.

```
meta <- data.frame(sample = paste0("sample", c("A1", "A2", "A3", "B1", "B2", "B3")),
                   condition = c("A", "A", "A", "B", "B", "B"),
                   stringsAsFactors = FALSE)
rownames(meta) <- meta$sample
meta
```

```
##            sample condition
## sampleA1 sampleA1         A
## sampleA2 sampleA2         A
## sampleA3 sampleA3         A
## sampleB1 sampleB1         B
## sampleB2 sampleB2         B
## sampleB3 sampleB3         B
```

### Definition of paths to reference files

(Back to top)

The following reference files (either pointing to annotation files downloaded from Ensembl or corresponding to files generated by the simulation/pre-processing code above) will be used throughout the analysis.

```
simdir <- "/home/Shared/data/alt_splicing_simulations/dte_simulation"
gtf <- paste0(simdir, "/annotations/chr1/Homo_sapiens.GRCh37.71.chr1.gtf")
cdna_fasta <- paste0(simdir, "/annotations/chr1/Homo_sapiens.GRCh37.71.cdna.chr1.fa")
truth_tx_sampleA1_file <- paste0(simdir, "/sim_data/sim2/chr1/RSEM/sampleA1_rsem.txt")
salmon_basedir <- paste0(simdir, "/sim_data/sim2/chr1/salmon")
fc_file <- paste0(simdir, "/sim_data/sim2/chr1/featureCounts/fc.Rdata")
fc_file_incomplete <- gsub("fc.Rdata", "fc_incomplete.Rdata", gsub("chr1", "chr1_incomplete", fc_file))
truth_gene_file <- paste0(simdir, "/sim_data/sim2/chr1/truth_gene.txt")
truth_tx_file <- paste0(simdir, "/sim_data/sim2/chr1/truth_transcript.txt")
```

### Definition of paths to generated files

(Back to top)

The following files will be generated by the code below.

```
feature_lengths_file <- paste0(basedir, "/annotation/Homo_Sapiens_GRCh37.71_chr1/feature_lengths.Rdata")
tx_gene_file <- paste0(basedir, "/annotation/Homo_Sapiens_GRCh37.71_chr1/tx_gene_map.Rdata")
```

### Definition of gene-to-transcript mapping

(Back to top)

Here we calculate the lengths of all genes and transcript (both defined here by the union of their exons), and define a mapping between gene and transcript identifiers from the reference files.

```
if (!file.exists(feature_lengths_file)) {
  calc_lengths_mapping(gtf = gtf, cdna_fasta = cdna_fasta, 
                       feature_lengths_file = feature_lengths_file, 
                       tx_gene_file = tx_gene_file) 
} else {
  message("feature lengths and tx-to-gene map already calculated.")
}
```

```
## feature lengths and tx-to-gene map already calculated.
```

```
load(feature_lengths_file)
load(tx_gene_file)
```

### Summarization of Salmon results and offset estimation

(Back to top)

We use the `tximport` package (https://github.com/mikelove/tximport) to generate count matrices and offset matrices (average transcript lengths) from the Salmon transcript-level estimates. We generate two different count matrices (**simplesum** and **scaledTPM**), and additionally create offsets to be used with the **simplesum** matrix.

#### Complete annotation

(Back to top)

```
salmon_files <- list.files(salmon_basedir, pattern = "sample", full.names = TRUE)
salmon_files <- salmon_files[file.info(salmon_files)$isdir]
salmon_files <- paste0(salmon_files, "/quant.sf")
salmon_files <- salmon_files[file.exists(salmon_files)]
names(salmon_files) <- basename(gsub("/quant.sf", "", salmon_files))
txi_salmonsimplesum <- tximport(files = salmon_files, type = "salmon", txIn = TRUE,
                                txOut = FALSE, countsFromAbundance = "no", 
                                gene2tx = gene2tx)
```

```
## reading in files
```

```
## 1
```

```
## 2
```

```
## 3
```

```
## 4
```

```
## 5
```

```
## 6
```

```
##
```

```
## summarizing abundance
```

```
## summarizing counts
```

```
## summarizing length
```

```
txi_salmonscaledtpm <- tximport(files = salmon_files, type = "salmon", txIn = TRUE,
                                txOut = FALSE, countsFromAbundance = "scaledTPM", 
                                gene2tx = gene2tx)
```

```
## reading in files
```

```
## 1
```

```
## 2
```

```
## 3
```

```
## 4
```

```
## 5
```

```
## 6
```

```
##
```

```
## summarizing abundance
```

```
## summarizing counts
```

```
## summarizing length
```

```
txi_salmontx <- tximport(files = salmon_files, type = "salmon", txIn = TRUE,
                         txOut = TRUE, countsFromAbundance = "no", gene2tx = gene2tx)
```

```
## reading in files
```

```
## 1
```

```
## 2
```

```
## 3
```

```
## 4
```

```
## 5
```

```
## 6
```

```
##
```

```
salmon_quant <- list(geneCOUNT_sal_simplesum = txi_salmonsimplesum$counts,
                     geneCOUNT_sal_scaledTPM = txi_salmonscaledtpm$counts,
                     avetxlength = txi_salmonsimplesum$length,
                     geneTPM_sal = txi_salmonsimplesum$abundance,
                     txTPM_sal = txi_salmontx$abundance,
                     txCOUNT_sal = txi_salmontx$counts,
                     txi_salmonsimplesum = txi_salmonsimplesum,
                     txi_salmonscaledtpm = txi_salmonscaledtpm,
                     txi_salmontx = txi_salmontx)
```

#### Incomplete annotation

(Back to top)

```
salmon_files <- list.files(gsub("chr1", "chr1_incomplete", salmon_basedir), 
                           pattern = "sample", full.names = TRUE)
salmon_files <- salmon_files[file.info(salmon_files)$isdir]
salmon_files <- paste0(salmon_files, "/quant.sf")
salmon_files <- salmon_files[file.exists(salmon_files)]
names(salmon_files) <- basename(gsub("/quant.sf", "", salmon_files))
txi_salmonsimplesum <- tximport(files = salmon_files, type = "salmon", txIn = TRUE,
                                txOut = FALSE, countsFromAbundance = "no", 
                                gene2tx = gene2tx)
```

```
## reading in files
```

```
## 1
```

```
## 2
```

```
## 3
```

```
## 4
```

```
## 5
```

```
## 6
```

```
##
```

```
## summarizing abundance
```

```
## summarizing counts
```

```
## summarizing length
```

```
txi_salmonscaledtpm <- tximport(files = salmon_files, type = "salmon", txIn = TRUE,
                                txOut = FALSE, countsFromAbundance = "scaledTPM", 
                                gene2tx = gene2tx)
```

```
## reading in files
```

```
## 1
```

```
## 2
```

```
## 3
```

```
## 4
```

```
## 5
```

```
## 6
```

```
##
```

```
## summarizing abundance
```

```
## summarizing counts
```

```
## summarizing length
```

```
txi_salmontx <- tximport(files = salmon_files, type = "salmon", txIn = TRUE,
                         txOut = TRUE, countsFromAbundance = "no", gene2tx = gene2tx)
```

```
## reading in files
```

```
## 1
```

```
## 2
```

```
## 3
```

```
## 4
```

```
## 5
```

```
## 6
```

```
##
```

```
salmon_quant_incomplete <- list(geneCOUNT_sal_simplesum = txi_salmonsimplesum$counts,
                                geneCOUNT_sal_scaledTPM = txi_salmonscaledtpm$counts,
                                avetxlength = txi_salmonsimplesum$length,
                                geneTPM_sal = txi_salmonsimplesum$abundance,
                                txTPM_sal = txi_salmontx$abundance,
                                txCOUNT_sal = txi_salmontx$counts,
                                txi_salmonsimplesum = txi_salmonsimplesum,
                                txi_salmonscaledtpm = txi_salmonscaledtpm,
                                txi_salmontx = txi_salmontx)
```

#### Estimation of coefficients of variation for all samples

(Back to top)

```
salmon_dirs <- list.files(salmon_basedir, pattern = "sample", full.names = TRUE)
salmon_dirs <- salmon_dirs[file.info(salmon_dirs)$isdir]
names(salmon_dirs) <- basename(salmon_dirs)

## Transcript
CV_tx_salmon <- as.data.frame(sapply(salmon_dirs, function(w) {
  tmpdf <- as.data.frame(t(read.delim(paste0(w, "/quant_bootstraps.sf"), 
                                      skip = 12, header = TRUE, as.is = TRUE)))
  tmpcv <- apply(tmpdf, 1, sd)/apply(tmpdf, 1, mean)
  tmpcv[is.na(tmpcv)] <- 0
  names(tmpcv) <- rownames(tmpdf)
  tmpcv
}))
CV_tx_salmon$mean_cv <- apply(CV_tx_salmon[, names(salmon_dirs)], 1, mean)
CV_tx_salmon$min_cv <- apply(CV_tx_salmon[, names(salmon_dirs)], 1, min)
CV_tx_salmon$max_cv <- apply(CV_tx_salmon[, names(salmon_dirs)], 1, max)
CV_tx_salmon$median_cv <- apply(CV_tx_salmon[, names(salmon_dirs)], 1, median)

## Gene
CV_gene_salmon <- as.data.frame(sapply(salmon_dirs, function(w) {
  tmpdf <- as.data.frame(t(read.delim(paste0(w, "/quant_bootstraps.sf"), 
                                      skip = 12, header = TRUE, as.is = TRUE)))
  tmpdf$gene <- tx2gene$gene[match(rownames(tmpdf), tx2gene$transcript)]
  tmpdf <- tmpdf %>% group_by(gene) %>% summarise_each(funs(sum)) %>% as.data.frame
  rownames(tmpdf) <- tmpdf$gene
  tmpdf$gene <- NULL
  tmpcv <- apply(tmpdf, 1, sd)/apply(tmpdf, 1, mean)
  tmpcv[is.na(tmpcv)] <- 0
  names(tmpcv) <- rownames(tmpdf)
  tmpcv
}))
CV_gene_salmon$mean_cv <- apply(CV_gene_salmon[, names(salmon_dirs)], 1, mean)
CV_gene_salmon$min_cv <- apply(CV_gene_salmon[, names(salmon_dirs)], 1, min)
CV_gene_salmon$max_cv <- apply(CV_gene_salmon[, names(salmon_dirs)], 1, max)
CV_gene_salmon$median_cv <- apply(CV_gene_salmon[, names(salmon_dirs)], 1, median)
```

### Generation of exon bin counts with DEXSeq

(Back to top)

```
cmd <- paste0("python /home/Shared/Rlib/release-3.1-lib/DEXSeq/", 
              "python_scripts/dexseq_prepare_annotation.py --aggregate='no' ",
              gtf, " ", gsub("gtf$", "flattened.nomerge.gff", gtf))
message(cmd)
```

```
## python /home/Shared/Rlib/release-3.1-lib/DEXSeq/python_scripts/dexseq_prepare_annotation.py --aggregate='no' /home/Shared/data/alt_splicing_simulations/dte_simulation/annotations/chr1/Homo_sapiens.GRCh37.71.chr1.gtf /home/Shared/data/alt_splicing_simulations/dte_simulation/annotations/chr1/Homo_sapiens.GRCh37.71.chr1.flattened.nomerge.gff
```

```
if (!file.exists(gsub("gtf$", "flattened.nomerge.gff", gtf))) {
  system(cmd)
} else {
  message("Flattened annotation already exists.")
}
```

```
## Flattened annotation already exists.
```

```
for (i in c("A1", "A2", "A3", "B1", "B2", "B3")) {
  cmd <- paste0("python /home/Shared/Rlib/release-3.1-lib/DEXSeq/python_scripts/dexseq_count.py ", 
                "-p yes -r name -s no -f bam ",
                gsub("gtf$", "flattened.nomerge.gff", gtf), " ", 
                paste0(simdir, "/sim_data/sim2/chr1/BAM/sample", i, "/Aligned.out.bam"), " ",
                paste0(basedir, "/quantifications/sim2/dexseq_exon/sample", i, ".txt"))
  message(cmd)
  if (!file.exists(paste0(basedir, "/quantifications/sim2/dexseq_exon/sample", i, ".txt")))
    system(cmd)
  else
    message("DEXSeq counts for sample ", i, " already calculated.")
}
```

```
## python /home/Shared/Rlib/release-3.1-lib/DEXSeq/python_scripts/dexseq_count.py -p yes -r name -s no -f bam /home/Shared/data/alt_splicing_simulations/dte_simulation/annotations/chr1/Homo_sapiens.GRCh37.71.chr1.flattened.nomerge.gff /home/Shared/data/alt_splicing_simulations/dte_simulation/sim_data/sim2/chr1/BAM/sampleA1/Aligned.out.bam /home/charlotte/gene_vs_tx_quantification/quantifications/sim2/dexseq_exon/sampleA1.txt
```

```
## DEXSeq counts for sample A1 already calculated.
```

```
## python /home/Shared/Rlib/release-3.1-lib/DEXSeq/python_scripts/dexseq_count.py -p yes -r name -s no -f bam /home/Shared/data/alt_splicing_simulations/dte_simulation/annotations/chr1/Homo_sapiens.GRCh37.71.chr1.flattened.nomerge.gff /home/Shared/data/alt_splicing_simulations/dte_simulation/sim_data/sim2/chr1/BAM/sampleA2/Aligned.out.bam /home/charlotte/gene_vs_tx_quantification/quantifications/sim2/dexseq_exon/sampleA2.txt
```

```
## DEXSeq counts for sample A2 already calculated.
```

```
## python /home/Shared/Rlib/release-3.1-lib/DEXSeq/python_scripts/dexseq_count.py -p yes -r name -s no -f bam /home/Shared/data/alt_splicing_simulations/dte_simulation/annotations/chr1/Homo_sapiens.GRCh37.71.chr1.flattened.nomerge.gff /home/Shared/data/alt_splicing_simulations/dte_simulation/sim_data/sim2/chr1/BAM/sampleA3/Aligned.out.bam /home/charlotte/gene_vs_tx_quantification/quantifications/sim2/dexseq_exon/sampleA3.txt
```

```
## DEXSeq counts for sample A3 already calculated.
```

```
## python /home/Shared/Rlib/release-3.1-lib/DEXSeq/python_scripts/dexseq_count.py -p yes -r name -s no -f bam /home/Shared/data/alt_splicing_simulations/dte_simulation/annotations/chr1/Homo_sapiens.GRCh37.71.chr1.flattened.nomerge.gff /home/Shared/data/alt_splicing_simulations/dte_simulation/sim_data/sim2/chr1/BAM/sampleB1/Aligned.out.bam /home/charlotte/gene_vs_tx_quantification/quantifications/sim2/dexseq_exon/sampleB1.txt
```

```
## DEXSeq counts for sample B1 already calculated.
```

```
## python /home/Shared/Rlib/release-3.1-lib/DEXSeq/python_scripts/dexseq_count.py -p yes -r name -s no -f bam /home/Shared/data/alt_splicing_simulations/dte_simulation/annotations/chr1/Homo_sapiens.GRCh37.71.chr1.flattened.nomerge.gff /home/Shared/data/alt_splicing_simulations/dte_simulation/sim_data/sim2/chr1/BAM/sampleB2/Aligned.out.bam /home/charlotte/gene_vs_tx_quantification/quantifications/sim2/dexseq_exon/sampleB2.txt
```

```
## DEXSeq counts for sample B2 already calculated.
```

```
## python /home/Shared/Rlib/release-3.1-lib/DEXSeq/python_scripts/dexseq_count.py -p yes -r name -s no -f bam /home/Shared/data/alt_splicing_simulations/dte_simulation/annotations/chr1/Homo_sapiens.GRCh37.71.chr1.flattened.nomerge.gff /home/Shared/data/alt_splicing_simulations/dte_simulation/sim_data/sim2/chr1/BAM/sampleB3/Aligned.out.bam /home/charlotte/gene_vs_tx_quantification/quantifications/sim2/dexseq_exon/sampleB3.txt
```

```
## DEXSeq counts for sample B3 already calculated.
```

## Accuracy of abundance estimates

(Back to top)

The accuracy of the abundance estimates from Salmon and featureCounts is evaluated by comparing to the true TPMs underlying the simulation.

### All features

(Back to top)

```
## Complete annotation
truth_tx_sampleA1 <- read.delim(truth_tx_sampleA1_file, header = TRUE, as.is = TRUE)
## Add number of transcripts per gene
truth_tx_sampleA1 <- truth_tx_sampleA1 %>% group_by(gene_id) %>% 
  mutate(nbr_tx = length(transcript_id)) %>% ungroup() %>% as.data.frame()

sampleA1_tx_salmon <- Reduce(function(...) merge(..., by = "feature", all = TRUE), 
                             list(data.frame(feature = truth_tx_sampleA1$transcript_id,
                                             true_tpm = truth_tx_sampleA1$TPM, 
                                             gene = truth_tx_sampleA1$gene_id, 
                                             nbr_tx = truth_tx_sampleA1$nbr_tx,
                                             stringsAsFactors = FALSE),
                                  data.frame(feature = rownames(salmon_quant$txTPM_sal),
                                             est_tpm = salmon_quant$txTPM_sal[, "sampleA1"],
                                             stringsAsFactors = FALSE)))
sampleA1_tx_salmon$measure <- "salmon_transcript"
sampleA1_tx_salmon$annotation <- "complete annotation"

sampleA1_gene_salmon <- sampleA1_tx_salmon %>% group_by(gene) %>% 
  summarise(true_tpm = sumna(true_tpm), est_tpm = sumna(est_tpm), nbr_tx = nbr_tx[1]) %>% as.data.frame
# sampleA1_gene_salmon <- sampleA1_tx_salmon %>% group_by(gene) %>% 
#   summarise_each(funs(sumna), -c(feature, measure, annotation, nbr_tx)) %>% as.data.frame
sampleA1_gene_salmon$measure <- "salmon_gene"
sampleA1_gene_salmon$annotation <- "complete annotation"

load(fc_file)
sampleA1_gene_fc <- Reduce(function(...) merge(..., by = "gene", all.x = TRUE), 
                           list(sampleA1_gene_salmon[, c("gene", "true_tpm", "nbr_tx")], 
                                data.frame(gene = rownames(fc$counts),
                                           fc_count = fc$counts[, "sampleA1"],
                                           stringsAsFactors = FALSE),
                                data.frame(gene = names(gene_length),
                                           gene_length_unionexon = gene_length,
                                           stringsAsFactors = FALSE)))
sampleA1_gene_fc$fpkm_fc_unionexon <- 
  sampleA1_gene_fc$fc_count/(sampleA1_gene_fc$gene_length_unionexon/1e3 *
                               sum(sampleA1_gene_fc$fc_count, na.rm = TRUE)/1e6)
sampleA1_gene_fc$est_tpm <-
  sampleA1_gene_fc$fpkm_fc_unionexon/sum(sampleA1_gene_fc$fpkm_fc_unionexon, na.rm = TRUE) * 1e6
sampleA1_gene_fc$measure <- "featureCounts_FPKM_gene"
sampleA1_gene_fc$annotation <- "complete annotation"

## Incomplete annotation
truth_tx_sampleA1 <- read.delim(truth_tx_sampleA1_file, header = TRUE, as.is = TRUE)
## Add number of transcripts per gene
truth_tx_sampleA1 <- truth_tx_sampleA1 %>% group_by(gene_id) %>% 
  mutate(nbr_tx = length(transcript_id)) %>% ungroup() %>% as.data.frame()

sampleA1_tx_salmon_i <- Reduce(function(...) merge(..., by = "feature", all = TRUE), 
                               list(data.frame(feature = truth_tx_sampleA1$transcript_id,
                                               true_tpm = truth_tx_sampleA1$TPM, 
                                               gene = truth_tx_sampleA1$gene_id, 
                                               nbr_tx = truth_tx_sampleA1$nbr_tx, 
                                               stringsAsFactors = FALSE),
                                    data.frame(feature = rownames(salmon_quant_incomplete$txTPM_sal),
                                               est_tpm = salmon_quant_incomplete$txTPM_sal[, "sampleA1"],
                                               stringsAsFactors = FALSE)))
sampleA1_tx_salmon_i$measure <- "salmon_transcript"
sampleA1_tx_salmon_i$annotation <- "incomplete annotation"

sampleA1_gene_salmon_i <- sampleA1_tx_salmon_i %>% group_by(gene) %>% 
  summarise(true_tpm = sumna(true_tpm), est_tpm = sumna(est_tpm), nbr_tx = nbr_tx[1]) %>% as.data.frame
# sampleA1_gene_salmon_i <- sampleA1_tx_salmon_i %>% group_by(gene) %>% 
#   summarise_each(funs(sumna), -c(feature, measure, annotation)) %>% as.data.frame
sampleA1_gene_salmon_i$measure <- "salmon_gene"
sampleA1_gene_salmon_i$annotation <- "incomplete annotation"

load(fc_file_incomplete)
sampleA1_gene_fc_i <- Reduce(function(...) merge(..., by = "gene", all.x = TRUE), 
                             list(sampleA1_gene_salmon_i[, c("gene", "true_tpm", "nbr_tx")], 
                                  data.frame(gene = rownames(fc_incomplete$counts),
                                             fc_count = fc_incomplete$counts[, "sampleA1"],
                                             stringsAsFactors = FALSE),
                                  data.frame(gene = names(gene_length),
                                             gene_length_unionexon = gene_length,
                                             stringsAsFactors = FALSE)))
sampleA1_gene_fc_i$fpkm_fc_unionexon <- 
  sampleA1_gene_fc_i$fc_count/(sampleA1_gene_fc_i$gene_length_unionexon/1e3 *
                                 sum(sampleA1_gene_fc_i$fc_count, na.rm = TRUE)/1e6)
sampleA1_gene_fc_i$est_tpm <-
  sampleA1_gene_fc_i$fpkm_fc_unionexon/sum(sampleA1_gene_fc_i$fpkm_fc_unionexon, na.rm = TRUE) * 1e6
sampleA1_gene_fc_i$measure <- "featureCounts_FPKM_gene"
sampleA1_gene_fc_i$annotation <- "incomplete annotation"

## Merge into one data frame
df <- rbind(sampleA1_tx_salmon[, c("measure", "annotation", "nbr_tx", "true_tpm", "est_tpm")],
            sampleA1_gene_salmon[, c("measure", "annotation", "nbr_tx", "true_tpm", "est_tpm")],
            sampleA1_gene_fc[, c("measure", "annotation", "nbr_tx", "true_tpm", "est_tpm")],
            sampleA1_tx_salmon_i[, c("measure", "annotation", "nbr_tx", "true_tpm", "est_tpm")],
            sampleA1_gene_salmon_i[, c("measure", "annotation", "nbr_tx", "true_tpm", "est_tpm")],
            sampleA1_gene_fc_i[, c("measure", "annotation", "nbr_tx", "true_tpm", "est_tpm")])
df$measure <- factor(df$measure, levels = c("salmon_transcript", "salmon_gene", 
                                            "featureCounts_FPKM_gene"))

cors <- df %>% group_by(measure, annotation) %>%
  summarise(sp = signif(cor(true_tpm, est_tpm, use = "complete", method = "spearman"), 3))

ggplot(df, aes(x = true_tpm, y = est_tpm, col = measure)) + 
  scale_colour_manual(values = c("blue", "red", "dimgrey"), guide = FALSE) + 
  geom_abline(intercept = 0, slope = 1) + 
  facet_grid(annotation ~ measure) + 
  geom_point(alpha = 0.5) + 
  scale_x_log10() + scale_y_log10() + 
  xlab("True TPM") + ylab("Estimated TPM") + 
  plot_theme() + 
  geom_text(x = 3, y = -5, aes(label = paste0("cor = ", sp)), size = 3, data = cors)
```

Split by number of transcripts

```
df2 <- subset(df, annotation == "complete annotation")
df2$nbr_tx_cat <- Hmisc::cut2(df2$nbr_tx, g = 3)
cors2 <- df2 %>% group_by(measure, nbr_tx_cat) %>% 
  summarise(sp = signif(cor(true_tpm, est_tpm, use = "complete", method = "spearman"), 3))

ggplot(df2, aes(x = true_tpm, y = est_tpm, col = measure)) + 
  scale_colour_manual(values = c("blue", "red", "dimgrey"), guide = FALSE) + 
  geom_abline(intercept = 0, slope = 1) + 
  facet_grid(nbr_tx_cat ~ measure) + 
  geom_point(alpha = 0.5) + 
  scale_x_log10() + scale_y_log10() + 
  xlab("True TPM") + ylab("Estimated TPM") + 
  plot_theme() + 
  geom_text(x = 3, y = -5, aes(label = paste0("cor = ", sp)), size = 3, data = cors2)
```

### Only features with positive true and estimated TPMs

(Back to top)

```
df_sub <- subset(df, true_tpm > 0 & est_tpm > 0)
cors_sub <- df_sub %>% group_by(measure, annotation) %>%
  summarise(sp = signif(cor(true_tpm, est_tpm, use = "complete", method = "spearman"), 3))

ggplot(df_sub, aes(x = true_tpm, y = est_tpm, col = measure)) + 
  scale_colour_manual(values = c("blue", "red", "dimgrey"), guide = FALSE) + 
  geom_abline(intercept = 0, slope = 1) + 
  facet_grid(annotation ~ measure) + 
  geom_point(alpha = 0.5) + 
  scale_x_log10() + scale_y_log10() + 
  xlab("True TPM") + ylab("Estimated TPM") + 
  plot_theme() + 
  geom_text(x = 3, y = -5, aes(label = paste0("cor = ", sp)), size = 3, data = cors_sub)
```

### All features, after replacing missing estimates with 0

(Back to top)

```
df_sub <- df
df_sub$est_tpm[is.na(df_sub$est_tpm)] <- 0
cors_sub <- df_sub %>% group_by(measure, annotation) %>%
  summarise(sp = signif(cor(true_tpm, est_tpm, use = "complete", method = "spearman"), 3))

ggplot(df_sub, aes(x = true_tpm, y = est_tpm, col = measure)) + 
  scale_colour_manual(values = c("blue", "red", "dimgrey"), guide = FALSE) + 
  geom_abline(intercept = 0, slope = 1) + 
  facet_grid(annotation ~ measure) + 
  geom_point(alpha = 0.5) + 
  scale_x_log10() + scale_y_log10() + 
  xlab("True TPM") + ylab("Estimated TPM") + 
  plot_theme() + 
  geom_text(x = 3, y = -5, aes(label = paste0("cor = ", sp)), size = 3, data = cors_sub)
```

### Consider groups of paralogous genes only

```
## Consider only gene-level estimates
dfg <- rbind(sampleA1_gene_salmon[, c("gene", "measure", "annotation", "true_tpm", "est_tpm")],
             sampleA1_gene_fc[, c("gene", "measure", "annotation", "true_tpm", "est_tpm")])
dfg$measure <- factor(dfg$measure, levels = c("salmon_gene", 
                                             "featureCounts_FPKM_gene"))

## Find paralogous genes using biomaRt
ensembl <- useMart("ENSEMBL_MART_ENSEMBL", host = "www.ensembl.org")
ensembl <- useDataset("hsapiens_gene_ensembl", mart = ensembl)
bm <- getBM(attributes = c("ensembl_gene_id", "hsapiens_paralog_ensembl_gene"),
            filters = "ensembl_gene_id", values = dfg$gene, mart = ensembl)
bm <- bm[which(bm$hsapiens_paralog_ensembl_gene != ""), ]
bm <- subset(bm, hsapiens_paralog_ensembl_gene %in% dfg$gene)

tt <- sort(table(bm$ensembl_gene_id), decreasing = TRUE)
tt <- tt[which(tt >= 5)]
basisgenes <- c()
for (nm in names(tt)) {
  if (!any(c(nm, bm$hsapiens_paralog_ensembl_gene[which(bm$ensembl_gene_id == nm)]) %in% basisgenes)) {
      tmpdf <- subset(dfg, gene %in% c(nm, bm$hsapiens_paralog_ensembl_gene[which(bm$ensembl_gene_id == nm)]))
      if (median(tmpdf$true_tpm) > 5) {
        basisgenes <- c(basisgenes, nm)
      }
  }
}

pdf("paralogs_sim2.pdf", width = 7, height = 5)
for (gn in basisgenes) {
  dfg_sub <- subset(dfg, gene %in% c(gn, bm$hsapiens_paralog_ensembl_gene[which(bm$ensembl_gene_id == gn)]))
  cors_sub <- dfg_sub %>% group_by(measure) %>%
    summarise(sp = signif(cor(true_tpm, est_tpm, use = "complete", method = "spearman"), 3),
              pe = signif(cor(log10(true_tpm + 1), log10(est_tpm + 1), 
                              use = "complete", method = "pearson"), 3))
  xpos <- 0.25 * min(log10(dfg_sub$true_tpm[dfg_sub$true_tpm > 0])) + 
    0.75 * max(log10(dfg_sub$true_tpm[dfg_sub$true_tpm > 0]))
  ypos <- 0.75 * min(log10(dfg_sub$est_tpm[dfg_sub$est_tpm > 0])) + 
    0.25 * max(log10(dfg_sub$est_tpm[dfg_sub$est_tpm > 0]))
  ypos2 <- 0.8 * min(log10(dfg_sub$est_tpm[dfg_sub$est_tpm > 0])) + 
    0.2 * max(log10(dfg_sub$est_tpm[dfg_sub$est_tpm > 0]))
  print(ggplot(dfg_sub, aes(x = true_tpm, y = est_tpm, col = measure)) + 
          scale_colour_manual(values = c("red", "dimgrey"), guide = FALSE) + 
          geom_abline(intercept = 0, slope = 1) + 
          facet_grid(annotation ~ measure) + 
          geom_point(size = 2) + 
          scale_x_log10() + scale_y_log10() + 
          xlab("True TPM") + ylab("Estimated TPM") + 
          plot_theme() + ggtitle(gn) + 
          geom_text(x = xpos, y = ypos, aes(label = paste0("spearman = ", sp)), size = 3, data = cors_sub) + 
          geom_text(x = xpos, y = ypos2, aes(label = paste0("pearson = ", pe)), size = 3, data = cors_sub))
}
dev.off()
```

```
## pdf 
##   2
```

## Variability of transcript and gene TPMs

(Back to top)

To quantify the variability of the transcript and gene TPM estimates obtained with Salmon, we calculate the coefficients of variation across 30 bootstrap replicates for one of the samples in the data set.

```
sampleA1_tx_salmon <- merge(sampleA1_tx_salmon, 
                            data.frame(feature = rownames(CV_tx_salmon),
                                       coefvar = CV_tx_salmon[, "sampleA1"],
                                       stringsAsFactors = FALSE),
                            by = "feature", all = TRUE)

sampleA1_gene_salmon <- merge(sampleA1_gene_salmon, 
                              data.frame(gene = rownames(CV_gene_salmon),
                                         coefvar = CV_gene_salmon[, "sampleA1"],
                                         stringsAsFactors = FALSE),
                              by = "gene", all = TRUE)

cvs_sal <- data.frame(cvs = c(sampleA1_tx_salmon$coefvar, sampleA1_gene_salmon$coefvar), 
                      lev = rep(c("transcript", "gene"), c(nrow(sampleA1_tx_salmon), nrow(sampleA1_gene_salmon))))
ggplot(cvs_sal, aes(x = log10(cvs + 0.001), col = lev)) + 
  geom_line(stat = "density", size = 2) + 
  ggtitle("") + 
  xlab("log10(coefficient of variation + 0.001)") + ylab("density") + 
  scale_colour_manual(values = c("red", "blue"), name = "") + 
  plot_theme()
```

## Correlation between gene count estimates

(Back to top)

### All genes

(Back to top)

```
geneCOUNT_fc <- fc$counts[match(rownames(salmon_quant$geneCOUNT_sal_simplesum),
                                rownames(fc$counts)), ]
stopifnot(all(rownames(salmon_quant$geneCOUNT_sal_simplesum) == 
                rownames(salmon_quant$geneCOUNT_sal_scaledTPM)))
sampleA1 <- cbind(simplesum_salmon = salmon_quant$geneCOUNT_sal_simplesum[, "sampleA1"], 
                  scaledTPM_salmon = salmon_quant$geneCOUNT_sal_scaledTPM[, "sampleA1"],
                  featureCounts = geneCOUNT_fc[, "sampleA1"])
rownames(sampleA1) <- rownames(salmon_quant$geneCOUNT_sal_simplesum)
sampleA1 <- log2(sampleA1 + 1)
pairs(sampleA1, upper.panel = panel_smooth, lower.panel = panel_cor)
```

### Only genes with positive estimates with all methods

(Back to top)

```
pairs(sampleA1[which(rowSums(sampleA1 <= 0) == 0), ], upper.panel = panel_smooth,
      lower.panel = panel_cor)
```

### Example of genes with a big difference between simple sum and scaled TPM

(Back to top)

```
idx <- rownames(sampleA1)[order(abs(sampleA1[, "simplesum_salmon"] - sampleA1[, "scaledTPM_salmon"]), 
                                decreasing = TRUE)[1:5]]
2^sampleA1[idx, , drop = FALSE] - 1
```

```
##                 simplesum_salmon scaledTPM_salmon featureCounts
## ENSG00000268552         85.84020        5045.5211             0
## ENSG00000230953         12.00000         640.2443            12
## ENSG00000230777          2.00000         117.5560             2
## ENSG00000236876          9.00000         337.4714             9
## ENSG00000223804         64.39892        1917.5005            13
```

```
subset(tx2gene, gene %in% idx)
```

```
##            transcript            gene
## 13728 ENST00000440199 ENSG00000223804
## 13729 ENST00000445753 ENSG00000223804
## 13730 ENST00000418192 ENSG00000223804
## 13731 ENST00000440862 ENSG00000223804
## 13732 ENST00000429388 ENSG00000223804
## 14916 ENST00000452777 ENSG00000230777
## 14966 ENST00000420575 ENSG00000230953
## 15984 ENST00000424567 ENSG00000236876
## 15985 ENST00000536927 ENSG00000236876
## 17201 ENST00000601459 ENSG00000268552
```

## Differential transcript expression (DTE) analysis on gene and transcript level

(Back to top)

We perform DTE by applying *edgeR* to transcript-level counts obtained from Salmon. The p-values from edgeR are then aggregated into gene-wise FDR estimates using the `perGeneQValue` function from *DEXSeq*.

```
res_dte_salmon_tx <- diff_expression_edgeR(counts = salmon_quant$txCOUNT_sal, 
                                           meta = meta, cond_name = "condition", 
                                           sample_name = "sample", 
                                           gene_length_matrix = NULL) 
res_dte_salmon_tx$tt$gene <- as.character(tx2gene$gene[match(rownames(res_dte_salmon_tx$tt), 
                                                             tx2gene$transcript)])

## Summarise on gene level
geneSplit = split(seq(along = res_dte_salmon_tx$tt$gene), res_dte_salmon_tx$tt$gene)
pGene = sapply(geneSplit, function(i) min(res_dte_salmon_tx$tt$PValue[i]))
stopifnot(all(is.finite(pGene)))
theta = unique(sort(pGene))
q = DEXSeq:::perGeneQValueExact(pGene, theta, geneSplit)
qval_dte_salmon_gene = rep(NA_real_, length(pGene))
qval_dte_salmon_gene = q[match(pGene, theta)]
qval_dte_salmon_gene = pmin(1, qval_dte_salmon_gene)
names(qval_dte_salmon_gene) = names(geneSplit)
stopifnot(!any(is.na(qval_dte_salmon_gene)))
```

### Comparison of DTE performance on gene and transcript level

(Back to top)

Here, we compare the DTE performance evaluated on transcript- and gene-level. Note that both the result level (transcript results vs aggregated gene results) and the truth (differentially expressed transcript vs genes with at least one differentially expressed transcript) are different, and the performance is evaluated by comparing each result table to its respective truth.

```
truth_tx <- read.delim(truth_tx_file, header = TRUE, as.is = TRUE)
truth_any <- read.delim(truth_gene_file, header = TRUE, as.is = TRUE)
cobra_dte <- COBRAData(padj = data.frame(transcript_salmon = res_dte_salmon_tx$tt$FDR, 
                                         row.names = rownames(res_dte_salmon_tx$tt)),
                       truth = data.frame(truth_tx[, "status", drop = FALSE], 
                                          row.names = truth_tx$transcript,
                                          stringsAsFactors = FALSE))

tmp <- truth_any[, "status_any", drop = FALSE]
colnames(tmp) <- "status"
cobra_dte <- COBRAData(padj = data.frame(gene_salmon = qval_dte_salmon_gene, 
                                         row.names = names(qval_dte_salmon_gene)),
                       truth = data.frame(tmp, 
                                          row.names = truth_any$gene,
                                          stringsAsFactors = FALSE),
                       object_to_extend = cobra_dte)
cobraperf_dte <- calculate_performance(cobra_dte, binary_truth = "status",
                                       aspects = c("fdrtpr", "fdrtprcurve"),
                                       onlyshared = TRUE)
cobraplot_dte <- prepare_data_for_plot(cobraperf_dte, colorscheme = c("red", "blue"), 
                                       facetted = FALSE)

fdrtpr(cobraplot_dte)$fullmethod <- gsub("_overall", "", fdrtpr(cobraplot_dte)$fullmethod)
fdrtprcurve(cobraplot_dte)$fullmethod <- gsub("_overall", "", fdrtprcurve(cobraplot_dte)$fullmethod)
plot_fdrtprcurve(cobraplot_dte, pointsize = 2, xaxisrange = c(0, 0.7)) + 
  theme(legend.position = "bottom", legend.text = element_text(size = 20))
```

#### Exploration of power difference between gene- and transcript-level analyses

(Back to top)

```
g2tx <- gene2tx
g2tx$txstatus <- truth(cobra_dte)$status[match(g2tx$transcript, rownames(truth(cobra_dte)))]
g2tx$txsign <- as.numeric(padj(cobra_dte)$transcript_salmon[match(g2tx$transcript, 
                                                                  rownames(padj(cobra_dte)))] <= 0.05)
g2tx$txsign[which(is.na(g2tx$txsign))] <- 0
nbrtx <- g2tx %>% group_by(gene) %>% summarise(nbr_tx = length(transcript),
                                               nbr_truedetx = sum(txstatus),
                                               nbr_signtx = sum(txsign),
                                               nbr_tptx = sum(txstatus*txsign)) %>% as.data.frame()

G <- merge(nbrtx, truth(cobra_dte), by.x = "gene", by.y = 0)
colnames(G)[colnames(G) == "status"] <- "gene_truedte"
G$gene_sign <- as.numeric(padj(cobra_dte)$gene_salmon[match(G$gene, rownames(padj(cobra_dte)))] <= 0.05)
G$gene_sign[which(is.na(G$gene_sign))] <- 0
G$gene_tp <- G$gene_sign*G$gene_truedte

G$frac_tptx <- G$nbr_tptx/G$nbr_truedetx
G$frac_tptx[which(is.na(G$frac_tptx))] <- 0

ggplot(subset(G, gene_tp == 1), aes(x = frac_tptx)) + geom_bar() + 
  plot_theme() + xlab("Fraction true positive transcripts") + 
  ggtitle("True positive genes")
```

### Evaluation of logFC estimation accuracy (transcripts)

(Back to top)

#### All transcripts

(Back to top)

```
cobra <- COBRAData(score = data.frame(transcript_salmon = abs(res_dte_salmon_tx$tt$logFC), 
                                      row.names = rownames(res_dte_salmon_tx$tt)))
truth_tx <- read.delim(truth_tx_file, header = TRUE, as.is = TRUE)
cobra <- COBRAData(truth = data.frame(truth_tx, row.names = truth_tx$transcript,
                                      stringsAsFactors = FALSE), object_to_extend = cobra)
cobraperf <- iCOBRA::calculate_performance(cobra, cont_truth = "logFC")
cobraplot1 <- iCOBRA::prepare_data_for_plot(cobraperf, incltruth = TRUE, facetted = FALSE,
                                            colorscheme = muted[c(1, 3, 5, 8, 10)])
corr(cobraplot1)$fullmethod <- gsub("_overall", "", corr(cobraplot1)$fullmethod)
deviation(cobraplot1)$fullmethod <- gsub("_overall", "", deviation(cobraplot1)$fullmethod)
scatter(cobraplot1)$fullmethod <- gsub("_overall", "", scatter(cobraplot1)$fullmethod)
```

```
iCOBRA::plot_corr(cobraplot1, pointsize = 5, corrtype = "spearman") + theme(legend.position = "bottom")
```

```
corr(cobraplot1)[, c("basemethod", "splitval", "PEARSON", "SPEARMAN")]
```

```
##          basemethod splitval   PEARSON  SPEARMAN
## 1 transcript_salmon  overall 0.4878148 0.4911797
```

```
iCOBRA::plot_deviation(cobraplot1) + theme(legend.position = "bottom")
```

```
iCOBRA::plot_scatter(cobraplot1, pointsize = 2) + theme(legend.position = "bottom") + 
  geom_abline(intercept = 0, slope = 1) + geom_point(size = 2)
```

#### Only transcripts with true logFC != 0 and true logFC != Inf

(Back to top)

```
################################################################################
## Remove genes with true logFC = 0 or Inf
keep <- rownames(truth(cobra))[which(!truth(cobra)$logFC %in% c(0, Inf))]
cobrasub <- cobra[keep, ]
cobraperf <- iCOBRA::calculate_performance(cobrasub, cont_truth = "logFC")
cobraplot1 <- iCOBRA::prepare_data_for_plot(cobraperf, incltruth = TRUE, facetted = FALSE,
                                            colorscheme = muted[c(1, 3, 5, 8, 10)])
corr(cobraplot1)$fullmethod <- gsub("_overall", "", corr(cobraplot1)$fullmethod)
deviation(cobraplot1)$fullmethod <- gsub("_overall", "", deviation(cobraplot1)$fullmethod)
scatter(cobraplot1)$fullmethod <- gsub("_overall", "", scatter(cobraplot1)$fullmethod)
```

```
iCOBRA::plot_corr(cobraplot1, pointsize = 5, corrtype = "spearman") + theme(legend.position = "bottom")
```

```
corr(cobraplot1)[, c("basemethod", "splitval", "PEARSON", "SPEARMAN")]
```

```
##          basemethod splitval   PEARSON  SPEARMAN
## 1 transcript_salmon  overall 0.5142394 0.7338135
```

```
iCOBRA::plot_deviation(cobraplot1) + theme(legend.position = "bottom")
```

```
iCOBRA::plot_scatter(cobraplot1, pointsize = 2) + theme(legend.position = "bottom") + 
  geom_abline(intercept = 0, slope = 1) + geom_point(size = 2)
```

## Differential gene expression analysis (edgeR)

(Back to top)

Given the gene count matrices defined above (corresponding to the **featureCounts**, **simplesum** and **scaledTPM** matrices mentioned in the manuscript), we apply *edgeR* (see below for *DESeq2* results) to perform differential gene expression. For the **simplesum** and **featureCounts** matrices, we also perform the analyses using the offsets derived from the average transcript lengths (**simplesum\_avetxl**).

```
res_sal_simplesum_edgeR <- diff_expression_edgeR(counts = salmon_quant$geneCOUNT_sal_simplesum, 
                                                 meta = meta, cond_name = "condition", 
                                                 sample_name = "sample", 
                                                 gene_length_matrix = NULL)
res_sal_simplesum_avetxl_edgeR <- diff_expression_edgeR(counts = salmon_quant$geneCOUNT_sal_simplesum, 
                                                        meta = meta, cond_name = "condition", 
                                                        sample_name = "sample", 
                                                        gene_length_matrix = salmon_quant$avetxlength)
res_sal_scaledTPM_edgeR <- diff_expression_edgeR(counts = salmon_quant$geneCOUNT_sal_scaledTPM, 
                                                 meta = meta, cond_name = "condition", 
                                                 sample_name = "sample", 
                                                 gene_length_matrix = NULL)
res_fc_edgeR <- diff_expression_edgeR(counts = geneCOUNT_fc, 
                                      meta = meta, cond_name = "condition", 
                                      sample_name = "sample", 
                                      gene_length_matrix = NULL)
res_fc_avetxl_edgeR <- diff_expression_edgeR(counts = geneCOUNT_fc, 
                                             meta = meta, cond_name = "condition", 
                                             sample_name = "sample", 
                                             gene_length_matrix = salmon_quant$avetxlength)
```

#### Diagnostics

(Back to top)

```
dfh <- data.frame(pvalue = c(res_fc_edgeR$tt$PValue,
                             res_fc_avetxl_edgeR$tt$PValue,
                             res_sal_scaledTPM_edgeR$tt$PValue,
                             res_sal_simplesum_edgeR$tt$PValue, 
                             res_sal_simplesum_avetxl_edgeR$tt$PValue),
                  mth = c(rep("featureCounts", nrow(res_fc_edgeR$tt)),
                          rep("featureCounts_avetxl", nrow(res_fc_avetxl_edgeR$tt)),
                          rep("scaledTPM_salmon", nrow(res_sal_scaledTPM_edgeR$tt)),
                          rep("simplesum_salmon", nrow(res_sal_simplesum_edgeR$tt)),
                          rep("simplesum_salmon_avetxl", nrow(res_sal_simplesum_avetxl_edgeR$tt))))
ggplot(dfh, aes(x = pvalue)) + geom_histogram() + facet_wrap(~mth) + 
  plot_theme() + 
  xlab("p-value") + ylab("count")
```

```
par(mfrow = c(2, 3))
plotBCV(res_fc_edgeR$dge, main = "featureCounts")
plotBCV(res_fc_avetxl_edgeR$dge, main = "featureCounts_avetxl")
plotBCV(res_sal_scaledTPM_edgeR$dge, main = "scaledTPM_salmon")
plotBCV(res_sal_simplesum_edgeR$dge, main = "simplesum_salmon")
plotBCV(res_sal_simplesum_avetxl_edgeR$dge, main = "simplesum_salmon_avetxl")
```

```
par(mfrow = c(2, 3))
plotSmear(res_fc_edgeR$dge, main = "featureCounts", ylim = c(-11, 11))
plotSmear(res_fc_avetxl_edgeR$dge, main = "featureCounts_avetxl", ylim = c(-11, 11))
plotSmear(res_sal_scaledTPM_edgeR$dge, main = "scaledTPM_salmon", ylim = c(-11, 11))
plotSmear(res_sal_simplesum_edgeR$dge, main = "simplesum_salmon", ylim = c(-11, 11))
plotSmear(res_sal_simplesum_avetxl_edgeR$dge, main = "simplesum_salmon_avetxl", ylim = c(-11, 11))

par(mfrow = c(1, 1))
```

### Comparison of significant genes found with different matrices

(Back to top)

```
cobra_edgeR <- COBRAData(padj = data.frame(simplesum_salmon = res_sal_simplesum_edgeR$tt$FDR, 
                                           row.names = rownames(res_sal_simplesum_edgeR$tt)))
cobra_edgeR <- COBRAData(padj = data.frame(scaledTPM_salmon = res_sal_scaledTPM_edgeR$tt$FDR, 
                                           row.names = rownames(res_sal_scaledTPM_edgeR$tt)),
                         object_to_extend = cobra_edgeR)
cobra_edgeR <- COBRAData(padj = data.frame(simplesum_salmon_avetxl = res_sal_simplesum_avetxl_edgeR$tt$FDR, 
                                           row.names = rownames(res_sal_simplesum_avetxl_edgeR$tt)),
                         object_to_extend = cobra_edgeR)
cobra_edgeR <- COBRAData(padj = data.frame(featureCounts = res_fc_edgeR$tt$FDR,
                                           row.names = rownames(res_fc_edgeR$tt)), 
                         object_to_extend = cobra_edgeR)
cobra_edgeR <- COBRAData(padj = data.frame(featureCounts_avetxl = res_fc_avetxl_edgeR$tt$FDR,
                                           row.names = rownames(res_fc_avetxl_edgeR$tt)), 
                         object_to_extend = cobra_edgeR)
truth_gene <- read.delim(truth_gene_file, header = TRUE, as.is = TRUE, row.names = 1)
cobra_edgeR <- COBRAData(truth = truth_gene, object_to_extend = cobra_edgeR)
cobraperf_edgeR <- calculate_performance(cobra_edgeR, binary_truth = "status_overall")
```

```
cobraplot1_edgeR <- prepare_data_for_plot(cobraperf_edgeR, incltruth = FALSE, 
                                          colorscheme = muted[c(1, 3, 5, 8, 10)])
plot_overlap(cobraplot1_edgeR, cex = c(1, 0.7, 0.7))
```

### Comparison to truth

(Back to top)

```
## FDR/TPR and FPC
cobraplot2_edgeR <- prepare_data_for_plot(cobraperf_edgeR, incltruth = TRUE, 
                                          colorscheme = muted[c(1, 3, 5, 8, 10, 13)],
                                          facetted = FALSE)
fdrtpr(cobraplot2_edgeR)$fullmethod <- gsub("_overall", "", fdrtpr(cobraplot2_edgeR)$fullmethod)
fdrtprcurve(cobraplot2_edgeR)$fullmethod <- gsub("_overall", "", fdrtprcurve(cobraplot2_edgeR)$fullmethod)
plot_fdrtprcurve(cobraplot2_edgeR, pointsize = 3, xaxisrange = c(0, 0.8), 
                 linewidth = 2) + 
  theme(legend.position = "bottom") + guides(colour = guide_legend(nrow = 2))
```

```
fpc(cobraplot2_edgeR)$fullmethod <- gsub("_overall", "", fpc(cobraplot2_edgeR)$fullmethod)
plot_fpc(cobraplot2_edgeR) + plot_theme() + 
  theme(legend.position = "bottom") + guides(colour = guide_legend(nrow = 2))
```

```
## Stratify by diff isoform usage
cobraperf3_edgeR <- calculate_performance(cobra_edgeR, binary_truth = "status_overall",
                                          splv = "diffisouse", maxsplit = 4)
cobraplot3_edgeR <- prepare_data_for_plot(cobraperf3_edgeR, incltruth = TRUE, 
                                          incloverall = FALSE, 
                                          colorscheme = muted[c(1, 3, 5, 8, 10, 13)])
levels(fdrtpr(cobraplot3_edgeR)$splitval)[levels(fdrtpr(cobraplot3_edgeR)$splitval) == "diffisouse:0"] <- "no differential isoform usage"
levels(fdrtpr(cobraplot3_edgeR)$splitval)[levels(fdrtpr(cobraplot3_edgeR)$splitval) == "diffisouse:1"] <- "differential isoform usage"
levels(fdrtprcurve(cobraplot3_edgeR)$splitval)[levels(fdrtprcurve(cobraplot3_edgeR)$splitval) == "diffisouse:0"] <- "no differential isoform usage"
levels(fdrtprcurve(cobraplot3_edgeR)$splitval)[levels(fdrtprcurve(cobraplot3_edgeR)$splitval) == "diffisouse:1"] <- "differential isoform usage"
plot_fdrtprcurve(cobraplot3_edgeR, pointsize = 2, xaxisrange = c(0, 0.8),
                 linewidth = 2) + 
  theme(legend.position = "bottom") + guides(colour = guide_legend(nrow = 2))
```

### Comparison of logFC estimates

(Back to top)

#### All methods

(Back to top)

```
df1 <- Reduce(function(...) merge(..., by = "gene", all = TRUE), 
              list(data.frame(gene = rownames(res_fc_edgeR$tt),
                              featureCounts = res_fc_edgeR$tt$logFC,
                              stringsAsFactors = FALSE),
                   data.frame(gene = rownames(res_fc_avetxl_edgeR$tt),
                              featureCounts_avetxl = res_fc_avetxl_edgeR$tt$logFC, 
                              stringsAsFactors = FALSE),
                   data.frame(gene = rownames(res_sal_scaledTPM_edgeR$tt),
                              scaledTPM_salmon = res_sal_scaledTPM_edgeR$tt$logFC,
                              stringsAsFactors = FALSE),
                   data.frame(gene = rownames(res_sal_simplesum_edgeR$tt),
                              simplesum_salmon = res_sal_simplesum_edgeR$tt$logFC, 
                              stringsAsFactors = FALSE),
                   data.frame(gene = rownames(res_sal_simplesum_avetxl_edgeR$tt),
                              simplesum_salmon_avetxl =
                                res_sal_simplesum_avetxl_edgeR$tt$logFC,
                              stringsAsFactors = FALSE)))
rownames(df1) <- df1$gene
df1$gene <- NULL
pairs(df1, upper.panel = panel_smooth, lower.panel = panel_cor)
```

#### simplesum vs scaledTPM

(Back to top)

```
truth_gene <- read.delim(truth_gene_file, header = TRUE, as.is = TRUE, row.names = 1)
df2 <- Reduce(function(...) merge(..., by = "gene", all = TRUE), 
              list(data.frame(gene = rownames(salmon_quant$geneTPM_sal),
                              salmon_quant$geneTPM_sal,
                              stringsAsFactors = FALSE),
                   data.frame(gene = rownames(truth_gene), 
                              DTU = c("No DTU", "DTU")[truth_gene$dtu + 1],
                              DTE = c("No DTE", "DTE")[truth_gene$dte + 1],
                              DGE = c("No DGE", "DGE")[truth_gene$dge + 1],
                              stringsAsFactors = FALSE), 
                   data.frame(gene = rownames(res_sal_scaledTPM_edgeR$tt),
                              scaledTPM_salmon_logFC = res_sal_scaledTPM_edgeR$tt$logFC,
                              scaledTPM_salmon_logCPM = res_sal_scaledTPM_edgeR$tt$logCPM, 
                              stringsAsFactors = FALSE),
                   data.frame(gene = rownames(res_sal_simplesum_edgeR$tt),
                              simplesum_salmon_logFC = res_sal_simplesum_edgeR$tt$logFC, 
                              simplesum_salmon_logCPM = res_sal_simplesum_edgeR$tt$logCPM, 
                              stringsAsFactors = FALSE),
                   data.frame(gene = rownames(res_sal_simplesum_avetxl_edgeR$tt),
                              simplesum_salmon_avetxl_logFC =
                                res_sal_simplesum_avetxl_edgeR$tt$logFC,
                              simplesum_salmon_avetxl_logCPM = 
                                res_sal_simplesum_avetxl_edgeR$tt$logCPM, 
                              stringsAsFactors = FALSE)))
rownames(df2) <- df2$gene
df2$gene <- NULL
df2$scaledTPM_salmon_logCPMbinary <- Hmisc::cut2(df2$scaledTPM_salmon_logCPM, g = 2)
df2$simplesum_salmon_logCPMbinary <- Hmisc::cut2(df2$simplesum_salmon_logCPM, g = 2)
df2$simplesum_salmon_avetxl_logCPMbinary <- Hmisc::cut2(df2$simplesum_salmon_avetxl_logCPM, g = 2)
df2$categ <- rep("Not differential", nrow(df2))
df2$categ[df2$DTU == "DTU"] <- "DTU"
df2$categ[df2$DTE == "DTE"] <- "DTE"
df2$categ[df2$DGE == "DGE"] <- "DGE"
df2$sumA <- df2$sampleA1 + df2$sampleA2 + df2$sampleA3
df2$sumB <- df2$sampleB1 + df2$sampleB2 + df2$sampleB3
df2$allzero_onecond <- "expressed in both groups"
df2$allzero_onecond[union(which(df2$sumA == 0), which(df2$sumB == 0))] <- "expressed in one group"
```

```
ggplot(df2, aes(x = simplesum_salmon_logFC, y = scaledTPM_salmon_logFC, col = DTU)) + 
  geom_abline(intercept = 0, slope = 1) + 
  geom_point(size = 2, alpha = 0.5) + 
  plot_theme() + ggtitle("sim2") + theme(legend.position = "bottom") + 
  scale_color_manual(values = c("red", "blue"), name = "") + 
  xlab("simplesum_salmon, logFC") + ylab("scaledTPM_salmon, logFC") + 
  guides(colour = guide_legend(override.aes = list(size = 7)))
```

```
ggplot(df2, aes(x = simplesum_salmon_logFC, y = scaledTPM_salmon_logFC, col = categ)) + 
  geom_abline(intercept = 0, slope = 1) + 
  geom_point(size = 2, alpha = 0.5) + 
  plot_theme() + ggtitle("sim2") + theme(legend.position = "bottom") + 
  scale_color_manual(values = c("red", "blue", "green", "dimgrey"), name = "") + 
  xlab("simplesum_salmon, logFC") + ylab("scaledTPM_salmon, logFC") + 
  guides(colour = guide_legend(override.aes = list(size = 7)))
```

```
ggplot(df2, aes(x = simplesum_salmon_logFC, y = scaledTPM_salmon_logFC, col = allzero_onecond)) + 
  geom_abline(intercept = 0, slope = 1) + 
  geom_point(size = 2, alpha = 0.5) + 
  plot_theme() + ggtitle("sim2") + theme(legend.position = "bottom") + 
  scale_color_manual(values = c("blue", "red"), name = "") + 
  xlab("simplesum_salmon, logFC") + ylab("scaledTPM_salmon, logFC") + 
  guides(colour = guide_legend(override.aes = list(size = 7)))
```

```
ggplot(subset(df2, !is.na(scaledTPM_salmon_logCPMbinary)), 
       aes(x = simplesum_salmon_logFC, y = scaledTPM_salmon_logFC, 
           col = scaledTPM_salmon_logCPMbinary)) + 
  geom_abline(intercept = 0, slope = 1) + 
  geom_point(size = 2, alpha = 0.5) + 
  facet_wrap(~scaledTPM_salmon_logCPMbinary) + 
  plot_theme() + ggtitle("sim2") + theme(legend.position = "bottom") + 
  xlab("simplesum_salmon, logFC") + ylab("scaledTPM_salmon, logFC") + 
  scale_color_manual(values = c("red", "blue"), name = "scaledTPM_salmon, logCPM") + 
  guides(colour = guide_legend(override.aes = list(size = 7)))
```

### Evaluation of logFC estimation accuracy (genes)

(Back to top)

#### All genes

(Back to top)

```
cobra <- COBRAData(score = data.frame(simplesum_salmon = abs(res_sal_simplesum_edgeR$tt$logFC), 
                                      row.names = rownames(res_sal_simplesum_edgeR$tt)))
cobra <- COBRAData(score = data.frame(scaledTPM_salmon = abs(res_sal_scaledTPM_edgeR$tt$logFC), 
                                      row.names = rownames(res_sal_scaledTPM_edgeR$tt)),
                   object_to_extend = cobra)
cobra <- COBRAData(score = data.frame(simplesum_salmon_avetxl = abs(res_sal_simplesum_avetxl_edgeR$tt$logFC), 
                                      row.names = rownames(res_sal_simplesum_avetxl_edgeR$tt)),
                   object_to_extend = cobra)
cobra <- COBRAData(score = data.frame(featureCounts = abs(res_fc_edgeR$tt$logFC),
                                      row.names = rownames(res_fc_edgeR$tt)), 
                   object_to_extend = cobra)
cobra <- COBRAData(score = data.frame(featureCounts_avetxl = abs(res_fc_avetxl_edgeR$tt$logFC),
                                      row.names = rownames(res_fc_avetxl_edgeR$tt)), 
                   object_to_extend = cobra)
truth_gene <- read.delim(truth_gene_file, header = TRUE, as.is = TRUE, row.names = 1)
cobra <- COBRAData(truth = truth_gene, object_to_extend = cobra)
cobraperf <- iCOBRA::calculate_performance(cobra, cont_truth = "logFC_overall")
cobraplot1 <- iCOBRA::prepare_data_for_plot(cobraperf, incltruth = TRUE, facetted = FALSE,
                                            colorscheme = muted[c(1, 3, 5, 8, 10)])
corr(cobraplot1)$fullmethod <- gsub("_overall", "", corr(cobraplot1)$fullmethod)
deviation(cobraplot1)$fullmethod <- gsub("_overall", "", deviation(cobraplot1)$fullmethod)
scatter(cobraplot1)$fullmethod <- gsub("_overall", "", scatter(cobraplot1)$fullmethod)
```

```
iCOBRA::plot_corr(cobraplot1, pointsize = 5, corrtype = "spearman") + theme(legend.position = "bottom") + 
  guides(colour = guide_legend(nrow = 2))
```

```
corr(cobraplot1)[, c("basemethod", "splitval", "PEARSON", "SPEARMAN")]
```

```
##                basemethod splitval   PEARSON  SPEARMAN
## 1           featureCounts  overall 0.6637244 0.4336520
## 2    featureCounts_avetxl  overall 0.7062586 0.4870360
## 3        scaledTPM_salmon  overall 0.5579301 0.4449023
## 4        simplesum_salmon  overall 0.6288003 0.4078021
## 5 simplesum_salmon_avetxl  overall 0.6651315 0.4558745
```

```
iCOBRA::plot_deviation(cobraplot1) + theme(legend.position = "bottom") + 
  guides(colour = guide_legend(nrow = 2))
```

```
iCOBRA::plot_scatter(cobraplot1, pointsize = 2) + theme(legend.position = "bottom") + 
  guides(colour = guide_legend(nrow = 2)) + geom_abline(intercept = 0, slope = 1) + geom_point(size = 2)
```

#### All genes, stratified by presence of differential isoform usage

(Back to top)

```
## Stratify by differential isoform usage
cobraperf2 <- iCOBRA::calculate_performance(cobra, cont_truth = "logFC_overall", 
                                            splv = "diffisouse")
cobraplot2 <- iCOBRA::prepare_data_for_plot(cobraperf2, incltruth = TRUE, facetted = TRUE,
                                            incloverall = FALSE, 
                                            colorscheme = muted[c(1, 3, 5, 8, 10)])
levels(corr(cobraplot2)$splitval)[levels(corr(cobraplot2)$splitval) == "diffisouse:0"] <- 
  "no differential isoform usage"
levels(corr(cobraplot2)$splitval)[levels(corr(cobraplot2)$splitval) == "diffisouse:1"] <-
  "differential isoform usage"
levels(deviation(cobraplot2)$splitval)[levels(deviation(cobraplot2)$splitval) == "diffisouse:0"] <-
  "no differential isoform usage"
levels(deviation(cobraplot2)$splitval)[levels(deviation(cobraplot2)$splitval) == "diffisouse:1"] <- 
  "differential isoform usage"
levels(scatter(cobraplot2)$splitval)[levels(scatter(cobraplot2)$splitval) == "diffisouse:0"] <-
  "no differential isoform usage"
levels(scatter(cobraplot2)$splitval)[levels(scatter(cobraplot2)$splitval) == "diffisouse:1"] <- 
  "differential isoform usage"
```

```
iCOBRA::plot_corr(cobraplot2, pointsize = 5, corrtype = "spearman") + theme(legend.position = "bottom") + 
  guides(colour = guide_legend(nrow = 2))
```

```
corr(cobraplot2)[, c("basemethod", "splitval", "PEARSON", "SPEARMAN")]
```

```
##                 basemethod                      splitval   PEARSON  SPEARMAN
## 1            featureCounts no differential isoform usage 0.7035890 0.5856803
## 2            featureCounts    differential isoform usage 0.4942483 0.1140712
## 4     featureCounts_avetxl no differential isoform usage 0.7024402 0.5848455
## 5     featureCounts_avetxl    differential isoform usage 0.7269604 0.4459979
## 7         scaledTPM_salmon no differential isoform usage 0.5275662 0.5182567
## 8         scaledTPM_salmon    differential isoform usage 0.7990480 0.4988737
## 10        simplesum_salmon no differential isoform usage 0.6434089 0.5440508
## 11        simplesum_salmon    differential isoform usage 0.5462938 0.1284064
## 13 simplesum_salmon_avetxl no differential isoform usage 0.6421550 0.5420244
## 14 simplesum_salmon_avetxl    differential isoform usage 0.8311927 0.4702301
```

```
iCOBRA::plot_deviation(cobraplot2) + theme(legend.position = "bottom") + 
  guides(colour = guide_legend(nrow = 2))
```

```
iCOBRA::plot_scatter(cobraplot2, pointsize = 2, doflip = TRUE) + theme(legend.position = "bottom") + 
  guides(colour = guide_legend(nrow = 2)) + geom_abline(intercept = 0, slope = 1) + geom_point(size = 2)
```

#### Only genes with true logFC != 0

(Back to top)

```
################################################################################
## Remove genes with true logFC = 0
keep <- rownames(truth(cobra))[which(truth(cobra)$logFC_overall != 0)]
cobrasub <- cobra[keep, ]
cobraperf <- iCOBRA::calculate_performance(cobrasub, cont_truth = "logFC_overall")
cobraplot1 <- iCOBRA::prepare_data_for_plot(cobraperf, incltruth = TRUE, facetted = FALSE,
                                            colorscheme = muted[c(1, 3, 5, 8, 10)])
corr(cobraplot1)$fullmethod <- gsub("_overall", "", corr(cobraplot1)$fullmethod)
deviation(cobraplot1)$fullmethod <- gsub("_overall", "", deviation(cobraplot1)$fullmethod)
scatter(cobraplot1)$fullmethod <- gsub("_overall", "", scatter(cobraplot1)$fullmethod)
```

```
iCOBRA::plot_corr(cobraplot1, pointsize = 5, corrtype = "spearman") + theme(legend.position = "bottom") + 
  guides(colour = guide_legend(nrow = 2))
```

```
corr(cobraplot1)[, c("basemethod", "splitval", "PEARSON", "SPEARMAN")]
```

```
##                basemethod splitval   PEARSON  SPEARMAN
## 1           featureCounts  overall 0.8633384 0.8764170
## 2    featureCounts_avetxl  overall 0.8797643 0.8990422
## 3        scaledTPM_salmon  overall 0.8561199 0.8932697
## 4        simplesum_salmon  overall 0.8742231 0.8758923
## 5 simplesum_salmon_avetxl  overall 0.8894210 0.8943751
```

```
iCOBRA::plot_deviation(cobraplot1) + theme(legend.position = "bottom") + 
  guides(colour = guide_legend(nrow = 2))
```

```
iCOBRA::plot_scatter(cobraplot1, pointsize = 2) + theme(legend.position = "bottom") + 
  guides(colour = guide_legend(nrow = 2)) + geom_abline(intercept = 0, slope = 1) + geom_point(size = 2)
```

#### Only genes with true logFC != 0, stratified by presence of differential isoform usage

(Back to top)

```
## Stratify by diff isoform usage
cobraperf2 <- iCOBRA::calculate_performance(cobrasub, cont_truth = "logFC_overall", 
                                            splv = "diffisouse")
cobraplot2 <- iCOBRA::prepare_data_for_plot(cobraperf2, incltruth = TRUE, facetted = TRUE, 
                                            incloverall = FALSE, 
                                            colorscheme = muted[c(1, 3, 5, 8, 10)])
levels(corr(cobraplot2)$splitval)[levels(corr(cobraplot2)$splitval) == "diffisouse:0"] <- 
  "no differential isoform usage"
levels(corr(cobraplot2)$splitval)[levels(corr(cobraplot2)$splitval) == "diffisouse:1"] <-
  "differential isoform usage"
levels(deviation(cobraplot2)$splitval)[levels(deviation(cobraplot2)$splitval) == "diffisouse:0"] <-
  "no differential isoform usage"
levels(deviation(cobraplot2)$splitval)[levels(deviation(cobraplot2)$splitval) == "diffisouse:1"] <- 
  "differential isoform usage"
levels(scatter(cobraplot2)$splitval)[levels(scatter(cobraplot2)$splitval) == "diffisouse:0"] <-
  "no differential isoform usage"
levels(scatter(cobraplot2)$splitval)[levels(scatter(cobraplot2)$splitval) == "diffisouse:1"] <- 
  "differential isoform usage"
```

```
iCOBRA::plot_corr(cobraplot2, pointsize = 5, corrtype = "spearman") + theme(legend.position = "bottom") + 
  guides(colour = guide_legend(nrow = 2))
```

```
corr(cobraplot2)[, c("basemethod", "splitval", "PEARSON", "SPEARMAN")]
```

```
##                 basemethod                      splitval   PEARSON  SPEARMAN
## 1            featureCounts no differential isoform usage 0.7994075 0.8339107
## 2            featureCounts    differential isoform usage 0.8160137 0.7252924
## 4     featureCounts_avetxl no differential isoform usage 0.8026411 0.8389572
## 5     featureCounts_avetxl    differential isoform usage 0.8656760 0.7884148
## 7         scaledTPM_salmon no differential isoform usage 0.7499904 0.8163444
## 8         scaledTPM_salmon    differential isoform usage 0.9150220 0.7961684
## 10        simplesum_salmon no differential isoform usage 0.8032161 0.8226264
## 11        simplesum_salmon    differential isoform usage 0.8588051 0.7348913
## 13 simplesum_salmon_avetxl no differential isoform usage 0.8058894 0.8282040
## 14 simplesum_salmon_avetxl    differential isoform usage 0.9132133 0.7831298
```

```
iCOBRA::plot_deviation(cobraplot2) + theme(legend.position = "bottom") + 
  guides(colour = guide_legend(nrow = 2))
```

```
iCOBRA::plot_scatter(cobraplot2, pointsize = 2, doflip = TRUE) + theme(legend.position = "bottom") + 
  guides(colour = guide_legend(nrow = 2)) + geom_abline(intercept = 0, slope = 1) + geom_point(size = 2)
```

## Comparison of DTE analysis aggregated on gene level to DGE

(Back to top)

A gene is considered truly differential if any of its transcripts are differential.

```
cobra <- COBRAData(padj = data.frame(simplesum_salmon_dge = res_sal_simplesum_edgeR$tt$FDR, 
                                     row.names = rownames(res_sal_simplesum_edgeR$tt)))
cobra <- COBRAData(padj = data.frame(scaledTPM_salmon_dge = res_sal_scaledTPM_edgeR$tt$FDR, 
                                     row.names = rownames(res_sal_scaledTPM_edgeR$tt)),
                   object_to_extend = cobra)
cobra <- COBRAData(padj = data.frame(simplesum_salmon_avetxl_dge = res_sal_simplesum_avetxl_edgeR$tt$FDR, 
                                     row.names = rownames(res_sal_simplesum_avetxl_edgeR$tt)),
                   object_to_extend = cobra)
cobra <- COBRAData(padj = data.frame(featureCounts_dge = res_fc_edgeR$tt$FDR,
                                     row.names = rownames(res_fc_edgeR$tt)), 
                   object_to_extend = cobra)
cobra <- COBRAData(padj = data.frame(featureCounts_avetxl_dge = res_fc_avetxl_edgeR$tt$FDR,
                                     row.names = rownames(res_fc_avetxl_edgeR$tt)), 
                   object_to_extend = cobra)
cobra <- COBRAData(padj = data.frame(salmon_dte = qval_dte_salmon_gene,
                                     row.names = names(qval_dte_salmon_gene)),
                   object_to_extend = cobra)
truth_gene <- read.delim(truth_gene_file, header = TRUE, as.is = TRUE, row.names = 1)
cobra <- COBRAData(truth = truth_gene, object_to_extend = cobra)
cobraperf <- calculate_performance(cobra, binary_truth = "status_any")

cobraplot <- prepare_data_for_plot(cobraperf, incltruth = FALSE, 
                                   colorscheme = muted[c(1, 3, 5, 8, 10, 13)],
                                   facetted = FALSE)
fdrtpr(cobraplot)$fullmethod <- gsub("_overall", "", fdrtpr(cobraplot)$fullmethod)
fdrtprcurve(cobraplot)$fullmethod <- gsub("_overall", "", fdrtprcurve(cobraplot)$fullmethod)
plot_fdrtprcurve(cobraplot, pointsize = 2, xaxisrange = c(0, 0.7)) + 
  theme(legend.position = "bottom") + guides(colour = guide_legend(nrow = 2))
```

## DTU analysis with DEXSeq

(Back to top)

### Application of DEXSeq to Salmon transcript counts

(Back to top)

```
BPPARAM = MulticoreParam(6)
stopifnot(all(colnames(salmon_quant$txCOUNT_sal) == rownames(meta)))
dxd_sal <- DEXSeqDataSet(countData = round(salmon_quant$txCOUNT_sal), 
                         sampleData = meta, 
                         design = ~sample + exon + condition:exon,
                         featureID = rownames(salmon_quant$txCOUNT_sal),
                         groupID = tx2gene$gene[match(rownames(salmon_quant$txCOUNT_sal),
                                                      tx2gene$transcript)])
dxd_sal <- estimateSizeFactors(dxd_sal)
dxd_sal <- estimateDispersions(dxd_sal, BPPARAM = BPPARAM)
```

```
## using supplied model matrix 
## using supplied model matrix 
## using supplied model matrix 
## using supplied model matrix 
## using supplied model matrix 
## using supplied model matrix 
## using supplied model matrix 
## using supplied model matrix 
## using supplied model matrix 
## using supplied model matrix 
## using supplied model matrix 
## using supplied model matrix
```

```
plotDispEsts(dxd_sal)
```

```
dxd_sal <- testForDEU(dxd_sal, BPPARAM = BPPARAM)
```

```
## using supplied model matrix 
## using supplied model matrix 
## using supplied model matrix 
## using supplied model matrix 
## using supplied model matrix 
## using supplied model matrix
```

```
dxr_sal <- DEXSeqResults(dxd_sal)
qval_dtu_salmon <- perGeneQValue(dxr_sal)
```

### DTU analysis on exon bin counts, with DEXSeq

(Back to top)

```
BPPARAM = MulticoreParam(6)
dexseqfiles <- list.files(paste0(basedir, "/quantifications/sim2/dexseq_exon"), 
                          full.names = TRUE, pattern = "sample")
names(dexseqfiles) <- gsub("\\.txt$", "", basename(dexseqfiles))
dxd_exon <- DEXSeqDataSetFromHTSeq(countfiles = dexseqfiles, 
                                   sampleData = meta[match(names(dexseqfiles), 
                                                           meta$sample), ],
                                   design = ~sample + exon + condition:exon)
dxd_exon <- estimateSizeFactors(dxd_exon)
dxd_exon <- estimateDispersions(dxd_exon, BPPARAM = BPPARAM)
```

```
## using supplied model matrix 
## using supplied model matrix 
## using supplied model matrix 
## using supplied model matrix 
## using supplied model matrix 
## using supplied model matrix 
## using supplied model matrix 
## using supplied model matrix 
## using supplied model matrix 
## using supplied model matrix 
## using supplied model matrix 
## using supplied model matrix
```

```
plotDispEsts(dxd_exon)
```

```
dxd_exon <- testForDEU(dxd_exon, BPPARAM = BPPARAM)
```

```
## using supplied model matrix 
## using supplied model matrix 
## using supplied model matrix 
## using supplied model matrix 
## using supplied model matrix 
## using supplied model matrix
```

```
dxr_exon <- DEXSeqResults(dxd_exon)
qval_dtu_dexseq_exon <- perGeneQValue(dxr_exon)
```

### Comparison to truth

(Back to top)

```
cobra <- COBRAData(padj = data.frame(salmon_dexseq = qval_dtu_salmon,
                                     row.names = names(qval_dtu_salmon),
                                     stringsAsFactors = FALSE))
cobra <- COBRAData(padj = data.frame(dexseq = qval_dtu_dexseq_exon,
                                     row.names = names(qval_dtu_dexseq_exon),
                                     stringsAsFactors = FALSE),
                   object_to_extend = cobra)
truth_gene <- read.delim(truth_gene_file, header = TRUE, as.is = TRUE, row.names = 1)
cobra <- COBRAData(truth = truth_gene, object_to_extend = cobra)
cobraperf <- calculate_performance(cobra, binary_truth = "diffisouse")
cobraplot <- prepare_data_for_plot(cobraperf, incltruth = TRUE, 
                                   colorscheme = c("blue", "red", "green"),
                                   facetted = FALSE)
fdrtpr(cobraplot)$fullmethod <- gsub("_overall", "", fdrtpr(cobraplot)$fullmethod)
fdrtprcurve(cobraplot)$fullmethod <- gsub("_overall", "", fdrtprcurve(cobraplot)$fullmethod)
```

```
plot_fdrtprcurve(cobraplot, pointsize = 2) + 
  theme(legend.position = "bottom") + guides(colour = guide_legend(nrow = 1))
```

```
plot_overlap(cobraplot, cex = c(1, 0.7, 0.7))
```

## Comparison of significant genes from DTE, DTU, DGE

(Back to top)

```
cobra <- COBRAData(padj = data.frame(salmon_dte = qval_dte_salmon_gene, 
                                     row.names = names(qval_dte_salmon_gene)))
cobra <- COBRAData(padj = data.frame(salmon_dexseq_dtu = qval_dtu_salmon, 
                                     row.names = names(qval_dtu_salmon)), 
                   object_to_extend = cobra)
cobra <- COBRAData(padj = data.frame(simplesum_salmon_avetxl_dge = res_sal_simplesum_avetxl_edgeR$tt$FDR, 
                                     row.names = rownames(res_sal_simplesum_avetxl_edgeR$tt)), 
                   object_to_extend = cobra)
cobraperf <- calculate_performance(cobra, aspects = "overlap", thr_venn = 0.05)
cobraplot <- prepare_data_for_plot(cobraperf)
plot_overlap(cobraplot, cex = c(1, 0.7, 0.7))
```

## Differential gene expression analysis (DESeq2)

(Back to top)

```
res_sal_simplesum_deseq2 <- diff_expression_DESeq2(txi = NULL,
                                                   counts = salmon_quant$geneCOUNT_sal_simplesum, 
                                                   meta = meta, cond_name = "condition", 
                                                   level1 = "A", level2 = "B", 
                                                   sample_name = "sample")
res_sal_simplesum_avetxl_deseq2 <- diff_expression_DESeq2(txi = salmon_quant$txi_salmonsimplesum,
                                                          counts = NULL, 
                                                          meta = meta, cond_name = "condition", 
                                                          level1 = "A", level2 = "B", 
                                                          sample_name = "sample")
res_sal_scaledTPM_deseq2 <- diff_expression_DESeq2(txi = NULL,
                                                   counts = salmon_quant$geneCOUNT_sal_scaledTPM, 
                                                   meta = meta, cond_name = "condition", 
                                                   level1 = "A", level2 = "B", 
                                                   sample_name = "sample")
res_fc_deseq2 <- diff_expression_DESeq2(txi = NULL,
                                        counts = geneCOUNT_fc, 
                                        meta = meta, cond_name = "condition", 
                                        level1 = "A", level2 = "B", 
                                        sample_name = "sample")
res_fc_avetxl_deseq2 <- 
  diff_expression_DESeq2(txi = list(counts = geneCOUNT_fc, 
                                    length = salmon_quant$avetxlength[match(rownames(geneCOUNT_fc),
                                                                            rownames(salmon_quant$avetxlength)),
                                                                      match(colnames(geneCOUNT_fc),
                                                                            colnames(salmon_quant$avetxlength))],
                                    countsFromAbundance = "no"),
                         counts = NULL, 
                         meta = meta, cond_name = "condition", 
                         level1 = "A", level2 = "B", 
                         sample_name = "sample")
```

#### Diagnostics

(Back to top)

```
dfh <- data.frame(pvalue = c(res_fc_deseq2$res$pvalue,
                             res_fc_avetxl_deseq2$res$pvalue,
                             res_sal_scaledTPM_deseq2$res$pvalue,
                             res_sal_simplesum_deseq2$res$pvalue, 
                             res_sal_simplesum_avetxl_deseq2$res$pvalue),
                  mth = c(rep("featureCounts", nrow(res_fc_deseq2$res)),
                          rep("featureCounts_avetxl", nrow(res_fc_avetxl_deseq2$res)),
                          rep("scaledTPM_salmon", nrow(res_sal_scaledTPM_deseq2$res)),
                          rep("simplesum_salmon", nrow(res_sal_simplesum_deseq2$res)),
                          rep("simplesum_salmon_avetxl", nrow(res_sal_simplesum_avetxl_deseq2$res))))
ggplot(dfh, aes(x = pvalue)) + geom_histogram() + facet_wrap(~mth) + 
  plot_theme() + 
  xlab("p-value") + ylab("count")
```

```
par(mfrow = c(2, 3))
plotDispEsts(res_fc_deseq2$dsd, main = "featureCounts")
plotDispEsts(res_fc_avetxl_deseq2$dsd, main = "featureCounts_avetxl")
plotDispEsts(res_sal_scaledTPM_deseq2$dsd, main = "scaledTPM_salmon")
plotDispEsts(res_sal_simplesum_deseq2$dsd, main = "simplesum_salmon")
plotDispEsts(res_sal_simplesum_avetxl_deseq2$dsd, main = "simplesum_salmon_avetxl")
```

```
par(mfrow = c(2, 3))
DESeq2::plotMA(res_fc_deseq2$dsd, main = "featureCounts")
DESeq2::plotMA(res_fc_avetxl_deseq2$dsd, main = "featureCounts_avetxl")
DESeq2::plotMA(res_sal_scaledTPM_deseq2$dsd, main = "scaledTPM_salmon")
DESeq2::plotMA(res_sal_simplesum_deseq2$dsd, main = "simplesum_salmon")
DESeq2::plotMA(res_sal_simplesum_avetxl_deseq2$dsd, main = "simplesum_salmon_avetxl")

par(mfrow = c(1, 1))
```

### Comparison of significant genes found with different matrices

(Back to top)

```
cobra_deseq2 <- COBRAData(padj = data.frame(simplesum_salmon = res_sal_simplesum_deseq2$res$padj, 
                                            row.names = rownames(res_sal_simplesum_deseq2$res)))
cobra_deseq2 <- COBRAData(padj = data.frame(scaledTPM_salmon = res_sal_scaledTPM_deseq2$res$padj, 
                                            row.names = rownames(res_sal_scaledTPM_deseq2$res)),
                          object_to_extend = cobra_deseq2)
cobra_deseq2 <- COBRAData(padj = data.frame(simplesum_salmon_avetxl = res_sal_simplesum_avetxl_deseq2$res$padj, 
                                            row.names = rownames(res_sal_simplesum_avetxl_deseq2$res)),
                          object_to_extend = cobra_deseq2)
cobra_deseq2 <- COBRAData(padj = data.frame(featureCounts = res_fc_deseq2$res$padj,
                                            row.names = rownames(res_fc_deseq2$res)), 
                          object_to_extend = cobra_deseq2)
cobra_deseq2 <- COBRAData(padj = data.frame(featureCounts_avetxl = res_fc_avetxl_deseq2$res$padj,
                                            row.names = rownames(res_fc_avetxl_deseq2$res)), 
                          object_to_extend = cobra_deseq2)
truth_gene <- read.delim(truth_gene_file, header = TRUE, as.is = TRUE, row.names = 1)
cobra_deseq2 <- COBRAData(truth = truth_gene, object_to_extend = cobra_deseq2)
cobraperf_deseq2 <- calculate_performance(cobra_deseq2, binary_truth = "status_overall")
cobraplot1_deseq2 <- prepare_data_for_plot(cobraperf_deseq2, incltruth = FALSE, 
                                           colorscheme = muted[c(1, 3, 5, 8, 10)])
```

```
plot_overlap(cobraplot1_deseq2, cex = c(1, 0.7, 0.7))
```

### Comparison to truth

(Back to top)

```
## FDR/TPR and FPC
cobraplot2_deseq2 <- prepare_data_for_plot(cobraperf_deseq2, incltruth = TRUE, 
                                           colorscheme = muted[c(1, 3, 5, 8, 10, 13)],
                                           facetted = FALSE)
fdrtpr(cobraplot2_deseq2)$fullmethod <- gsub("_overall", "", fdrtpr(cobraplot2_deseq2)$fullmethod)
fdrtprcurve(cobraplot2_deseq2)$fullmethod <- gsub("_overall", "", fdrtprcurve(cobraplot2_deseq2)$fullmethod)
plot_fdrtprcurve(cobraplot2_deseq2, pointsize = 2, xaxisrange = c(0, 0.8)) + 
  theme(legend.position = "bottom") + guides(colour = guide_legend(nrow = 2))
```

```
fpc(cobraplot2_deseq2)$fullmethod <- gsub("_overall", "", fpc(cobraplot2_deseq2)$fullmethod)
plot_fpc(cobraplot2_deseq2) + plot_theme() + 
  theme(legend.position = "bottom") + guides(colour = guide_legend(nrow = 2))
```

```
## Stratify by diff isoform usage
cobraperf3_deseq2 <- calculate_performance(cobra_deseq2, binary_truth = "status_overall",
                                           splv = "diffisouse", maxsplit = 4)
cobraplot3_deseq2 <- prepare_data_for_plot(cobraperf3_deseq2, incltruth = TRUE, 
                                           incloverall = FALSE, 
                                           colorscheme = muted[c(1, 3, 5, 8, 10, 13)])
levels(fdrtpr(cobraplot3_deseq2)$splitval)[levels(fdrtpr(cobraplot3_deseq2)$splitval) == "diffisouse:0"] <- "no differential isoform usage"
levels(fdrtpr(cobraplot3_deseq2)$splitval)[levels(fdrtpr(cobraplot3_deseq2)$splitval) == "diffisouse:1"] <- "differential isoform usage"
levels(fdrtprcurve(cobraplot3_deseq2)$splitval)[levels(fdrtprcurve(cobraplot3_deseq2)$splitval) == "diffisouse:0"] <- "no differential isoform usage"
levels(fdrtprcurve(cobraplot3_deseq2)$splitval)[levels(fdrtprcurve(cobraplot3_deseq2)$splitval) == "diffisouse:1"] <- "differential isoform usage"
plot_fdrtprcurve(cobraplot3_deseq2, pointsize = 2, xaxisrange = c(0, 0.8)) + 
  theme(legend.position = "bottom") + guides(colour = guide_legend(nrow = 2))
```

### Comparison of logFC estimates

(Back to top)

#### All methods

(Back to top)

```
df1 <- Reduce(function(...) merge(..., by = "gene", all = TRUE), 
              list(data.frame(gene = rownames(res_fc_deseq2$res),
                              featureCounts = res_fc_deseq2$res$log2FoldChange,
                              stringsAsFactors = FALSE),
                   data.frame(gene = rownames(res_fc_avetxl_deseq2$res),
                              featureCounts_avetxl = res_fc_avetxl_deseq2$res$log2FoldChange, 
                              stringsAsFactors = FALSE),
                   data.frame(gene = rownames(res_sal_scaledTPM_deseq2$res),
                              scaledTPM_salmon = res_sal_scaledTPM_deseq2$res$log2FoldChange,
                              stringsAsFactors = FALSE),
                   data.frame(gene = rownames(res_sal_simplesum_deseq2$res),
                              simplesum_salmon = res_sal_simplesum_deseq2$res$log2FoldChange, 
                              stringsAsFactors = FALSE),
                   data.frame(gene = rownames(res_sal_simplesum_avetxl_deseq2$res),
                              simplesum_salmon_avetxl =
                                res_sal_simplesum_avetxl_deseq2$res$log2FoldChange,
                              stringsAsFactors = FALSE)))
rownames(df1) <- df1$gene
df1$gene <- NULL
pairs(df1, upper.panel = panel_smooth, lower.panel = panel_cor)
```

#### simplesum vs scaledTPM

(Back to top)

```
truth_gene <- read.delim(truth_gene_file, header = TRUE, as.is = TRUE, row.names = 1)
df2 <- Reduce(function(...) merge(..., by = "gene", all = TRUE), 
              list(data.frame(gene = rownames(salmon_quant$geneTPM_sal),
                              salmon_quant$geneTPM_sal,
                              stringsAsFactors = FALSE),
                   data.frame(gene = rownames(truth_gene), 
                              DTU = c("No DTU", "DTU")[truth_gene$dtu + 1],
                              DTE = c("No DTE", "DTE")[truth_gene$dte + 1],
                              DGE = c("No DGE", "DGE")[truth_gene$dge + 1],
                              stringsAsFactors = FALSE), 
                   data.frame(gene = rownames(res_sal_scaledTPM_deseq2$res),
                              scaledTPM_salmon_logFC = res_sal_scaledTPM_deseq2$res$log2FoldChange,
                              scaledTPM_salmon_basemean = res_sal_scaledTPM_deseq2$res$baseMean, 
                              stringsAsFactors = FALSE),
                   data.frame(gene = rownames(res_sal_simplesum_deseq2$res),
                              simplesum_salmon_logFC = res_sal_simplesum_deseq2$res$log2FoldChange, 
                              simplesum_salmon_basemean = res_sal_simplesum_deseq2$res$baseMean, 
                              stringsAsFactors = FALSE),
                   data.frame(gene = rownames(res_sal_simplesum_avetxl_deseq2$res),
                              simplesum_salmon_avetxl_logFC =
                                res_sal_simplesum_avetxl_deseq2$res$log2FoldChange,
                              simplesum_salmon_avetxl_basemean = 
                                res_sal_simplesum_avetxl_deseq2$res$baseMean, 
                              stringsAsFactors = FALSE)))
rownames(df2) <- df2$gene
df2$gene <- NULL
df2$scaledTPM_salmon_basemeanbinary <- Hmisc::cut2(df2$scaledTPM_salmon_basemean, g = 2)
df2$simplesum_salmon_basemeanbinary <- Hmisc::cut2(df2$simplesum_salmon_basemean, g = 2)
df2$simplesum_salmon_avetxl_basemeanbinary <- Hmisc::cut2(df2$simplesum_salmon_avetxl_basemean, g = 2)
df2$categ <- rep("Not differential", nrow(df2))
df2$categ[df2$DTU == "DTU"] <- "DTU"
df2$categ[df2$DTE == "DTE"] <- "DTE"
df2$categ[df2$DGE == "DGE"] <- "DGE"
df2$sumA <- df2$sampleA1 + df2$sampleA2 + df2$sampleA3
df2$sumB <- df2$sampleB1 + df2$sampleB2 + df2$sampleB3
df2$allzero_onecond <- "expressed in both groups"
df2$allzero_onecond[union(which(df2$sumA == 0), which(df2$sumB == 0))] <- "expressed in one group"
```

```
ggplot(df2, aes(x = simplesum_salmon_logFC, y = scaledTPM_salmon_logFC, col = DTU)) + 
  geom_abline(intercept = 0, slope = 1) + 
  geom_point(size = 2, alpha = 0.5) + 
  plot_theme() + ggtitle("sim2") + theme(legend.position = "bottom") + 
  scale_color_manual(values = c("red", "blue"), name = "") + 
  xlab("simplesum_salmon, logFC") + ylab("scaledTPM_salmon, logFC") + 
  guides(colour = guide_legend(override.aes = list(size = 7)))
```

```
ggplot(df2, aes(x = simplesum_salmon_logFC, y = scaledTPM_salmon_logFC, col = categ)) + 
  geom_abline(intercept = 0, slope = 1) + 
  geom_point(size = 2, alpha = 0.5) + 
  plot_theme() + ggtitle("sim2") + theme(legend.position = "bottom") + 
  scale_color_manual(values = c("red", "blue", "green", "dimgrey"), name = "") + 
  xlab("simplesum_salmon, logFC") + ylab("scaledTPM_salmon, logFC") + 
  guides(colour = guide_legend(override.aes = list(size = 7)))
```

```
ggplot(df2, aes(x = simplesum_salmon_logFC, y = scaledTPM_salmon_logFC, col = allzero_onecond)) + 
  geom_abline(intercept = 0, slope = 1) + 
  geom_point(size = 2, alpha = 0.5) + 
  plot_theme() + ggtitle("sim2") + theme(legend.position = "bottom") + 
  scale_color_manual(values = c("blue", "red"), name = "") + 
  xlab("simplesum_salmon, logFC") + ylab("scaledTPM_salmon, logFC") + 
  guides(colour = guide_legend(override.aes = list(size = 7)))
```

```
ggplot(subset(df2, !is.na(scaledTPM_salmon_basemeanbinary)), 
       aes(x = simplesum_salmon_logFC, y = scaledTPM_salmon_logFC, 
           col = scaledTPM_salmon_basemeanbinary)) + 
  geom_abline(intercept = 0, slope = 1) + 
  geom_point(size = 2, alpha = 0.5) + 
  facet_wrap(~scaledTPM_salmon_basemeanbinary) + 
  plot_theme() + ggtitle("sim2") + theme(legend.position = "bottom") + 
  xlab("simplesum_salmon, logFC") + ylab("scaledTPM_salmon, logFC") + 
  scale_color_manual(values = c("red", "blue"), name = "scaledTPM_salmon, base mean") + 
  guides(colour = guide_legend(override.aes = list(size = 7)))
```

### Evaluation of logFC estimation accuracy (genes)

(Back to top)

#### All genes

(Back to top)

```
cobra <- COBRAData(score = data.frame(simplesum_salmon = abs(res_sal_simplesum_deseq2$res$log2FoldChange), 
                                      row.names = rownames(res_sal_simplesum_deseq2$res)))
cobra <- COBRAData(score = data.frame(scaledTPM_salmon = abs(res_sal_scaledTPM_deseq2$res$log2FoldChange), 
                                      row.names = rownames(res_sal_scaledTPM_deseq2$res)),
                   object_to_extend = cobra)
cobra <- COBRAData(score = data.frame(simplesum_salmon_avetxl =
                                        abs(res_sal_simplesum_avetxl_deseq2$res$log2FoldChange), 
                                      row.names = rownames(res_sal_simplesum_avetxl_deseq2$res)),
                   object_to_extend = cobra)
cobra <- COBRAData(score = data.frame(featureCounts = abs(res_fc_deseq2$res$log2FoldChange),
                                      row.names = rownames(res_fc_deseq2$res)), 
                   object_to_extend = cobra)
cobra <- COBRAData(score = data.frame(featureCounts_avetxl = abs(res_fc_avetxl_deseq2$res$log2FoldChange),
                                      row.names = rownames(res_fc_avetxl_deseq2$res)), 
                   object_to_extend = cobra)
truth_gene <- read.delim(truth_gene_file, header = TRUE, as.is = TRUE, row.names = 1)
cobra <- COBRAData(truth = truth_gene, object_to_extend = cobra)
cobraperf <- iCOBRA::calculate_performance(cobra, cont_truth = "logFC_overall")
cobraplot1 <- iCOBRA::prepare_data_for_plot(cobraperf, incltruth = TRUE, facetted = FALSE,
                                            colorscheme = muted[c(1, 3, 5, 8, 10)])
corr(cobraplot1)$fullmethod <- gsub("_overall", "", corr(cobraplot1)$fullmethod)
deviation(cobraplot1)$fullmethod <- gsub("_overall", "", deviation(cobraplot1)$fullmethod)
scatter(cobraplot1)$fullmethod <- gsub("_overall", "", scatter(cobraplot1)$fullmethod)
```

```
iCOBRA::plot_corr(cobraplot1, pointsize = 5, corrtype = "spearman") + theme(legend.position = "bottom") + 
  guides(colour = guide_legend(nrow = 2))
```

```
corr(cobraplot1)[, c("basemethod", "splitval", "PEARSON", "SPEARMAN")]
```

```
##                basemethod splitval   PEARSON  SPEARMAN
## 1           featureCounts  overall 0.7612140 0.4742037
## 2    featureCounts_avetxl  overall 0.8223537 0.5314520
## 3        scaledTPM_salmon  overall 0.7730250 0.5086579
## 4        simplesum_salmon  overall 0.7441198 0.4547910
## 5 simplesum_salmon_avetxl  overall 0.8011819 0.5077898
```

```
iCOBRA::plot_deviation(cobraplot1) + theme(legend.position = "bottom") + 
  guides(colour = guide_legend(nrow = 2))
```

```
iCOBRA::plot_scatter(cobraplot1, pointsize = 2) + theme(legend.position = "bottom") + 
  guides(colour = guide_legend(nrow = 2)) + geom_abline(intercept = 0, slope = 1) + geom_point(size = 2)
```

#### All genes, stratified by presence of differential isoform usage

(Back to top)

```
## Stratify by differential isoform usage
cobraperf2 <- iCOBRA::calculate_performance(cobra, cont_truth = "logFC_overall", 
                                            splv = "diffisouse")
cobraplot2 <- iCOBRA::prepare_data_for_plot(cobraperf2, incltruth = TRUE, facetted = TRUE,
                                            incloverall = FALSE, 
                                            colorscheme = muted[c(1, 3, 5, 8, 10)])
levels(corr(cobraplot2)$splitval)[levels(corr(cobraplot2)$splitval) == "diffisouse:0"] <- 
  "no differential isoform usage"
levels(corr(cobraplot2)$splitval)[levels(corr(cobraplot2)$splitval) == "diffisouse:1"] <-
  "differential isoform usage"
levels(deviation(cobraplot2)$splitval)[levels(deviation(cobraplot2)$splitval) == "diffisouse:0"] <-
  "no differential isoform usage"
levels(deviation(cobraplot2)$splitval)[levels(deviation(cobraplot2)$splitval) == "diffisouse:1"] <- 
  "differential isoform usage"
levels(scatter(cobraplot2)$splitval)[levels(scatter(cobraplot2)$splitval) == "diffisouse:0"] <-
  "no differential isoform usage"
levels(scatter(cobraplot2)$splitval)[levels(scatter(cobraplot2)$splitval) == "diffisouse:1"] <- 
  "differential isoform usage"
```

```
iCOBRA::plot_corr(cobraplot2, pointsize = 5, corrtype = "spearman") + theme(legend.position = "bottom") + 
  guides(colour = guide_legend(nrow = 2))
```

```
corr(cobraplot2)[, c("basemethod", "splitval", "PEARSON", "SPEARMAN")]
```

```
##                 basemethod                      splitval   PEARSON  SPEARMAN
## 1            featureCounts no differential isoform usage 0.8288448 0.6326767
## 2            featureCounts    differential isoform usage 0.5212447 0.1205842
## 4     featureCounts_avetxl no differential isoform usage 0.8310185 0.6345294
## 5     featureCounts_avetxl    differential isoform usage 0.7821129 0.4530669
## 7         scaledTPM_salmon no differential isoform usage 0.7640564 0.5934580
## 8         scaledTPM_salmon    differential isoform usage 0.8307163 0.5052982
## 10        simplesum_salmon no differential isoform usage 0.7900246 0.5991675
## 11        simplesum_salmon    differential isoform usage 0.5556445 0.1326059
## 13 simplesum_salmon_avetxl no differential isoform usage 0.7940672 0.6002799
## 14 simplesum_salmon_avetxl    differential isoform usage 0.8480329 0.4755535
```

```
iCOBRA::plot_deviation(cobraplot2) + theme(legend.position = "bottom") + 
  guides(colour = guide_legend(nrow = 2))
```

```
iCOBRA::plot_scatter(cobraplot2, pointsize = 2, doflip = TRUE) + theme(legend.position = "bottom") + 
  guides(colour = guide_legend(nrow = 2)) + geom_abline(intercept = 0, slope = 1) + geom_point(size = 2)
```

#### Only genes with true logFC != 0

(Back to top)

```
################################################################################
## Remove genes with true logFC = 0
keep <- rownames(truth(cobra))[which(truth(cobra)$logFC_overall != 0)]
cobrasub <- cobra[keep, ]
cobraperf <- iCOBRA::calculate_performance(cobrasub, cont_truth = "logFC_overall")
cobraplot1 <- iCOBRA::prepare_data_for_plot(cobraperf, incltruth = TRUE, facetted = FALSE,
                                            colorscheme = muted[c(1, 3, 5, 8, 10)])
corr(cobraplot1)$fullmethod <- gsub("_overall", "", corr(cobraplot1)$fullmethod)
deviation(cobraplot1)$fullmethod <- gsub("_overall", "", deviation(cobraplot1)$fullmethod)
scatter(cobraplot1)$fullmethod <- gsub("_overall", "", scatter(cobraplot1)$fullmethod)
```

```
iCOBRA::plot_corr(cobraplot1, pointsize = 5, corrtype = "spearman") + theme(legend.position = "bottom") + 
  guides(colour = guide_legend(nrow = 2))
```

```
corr(cobraplot1)[, c("basemethod", "splitval", "PEARSON", "SPEARMAN")]
```

```
##                basemethod splitval   PEARSON  SPEARMAN
## 1           featureCounts  overall 0.8742009 0.8780341
## 2    featureCounts_avetxl  overall 0.8925442 0.9007627
## 3        scaledTPM_salmon  overall 0.8842811 0.8911180
## 4        simplesum_salmon  overall 0.8752962 0.8744314
## 5 simplesum_salmon_avetxl  overall 0.8916919 0.8933705
```

```
iCOBRA::plot_deviation(cobraplot1) + theme(legend.position = "bottom") + 
  guides(colour = guide_legend(nrow = 2))
```

```
iCOBRA::plot_scatter(cobraplot1, pointsize = 2) + theme(legend.position = "bottom") + 
  guides(colour = guide_legend(nrow = 2)) + geom_abline(intercept = 0, slope = 1) + geom_point(size = 2)
```

#### Only genes with true logFC != 0, stratified by presence of differential isoform usage

(Back to top)

```
## Stratify by diff isoform usage
cobraperf2 <- iCOBRA::calculate_performance(cobrasub, cont_truth = "logFC_overall", 
                                            splv = "diffisouse")
cobraplot2 <- iCOBRA::prepare_data_for_plot(cobraperf2, incltruth = TRUE, facetted = TRUE, 
                                            incloverall = FALSE, 
                                            colorscheme = muted[c(1, 3, 5, 8, 10)])
levels(corr(cobraplot2)$splitval)[levels(corr(cobraplot2)$splitval) == "diffisouse:0"] <- 
  "no differential isoform usage"
levels(corr(cobraplot2)$splitval)[levels(corr(cobraplot2)$splitval) == "diffisouse:1"] <-
  "differential isoform usage"
levels(deviation(cobraplot2)$splitval)[levels(deviation(cobraplot2)$splitval) == "diffisouse:0"] <-
  "no differential isoform usage"
levels(deviation(cobraplot2)$splitval)[levels(deviation(cobraplot2)$splitval) == "diffisouse:1"] <- 
  "differential isoform usage"
levels(scatter(cobraplot2)$splitval)[levels(scatter(cobraplot2)$splitval) == "diffisouse:0"] <-
  "no differential isoform usage"
levels(scatter(cobraplot2)$splitval)[levels(scatter(cobraplot2)$splitval) == "diffisouse:1"] <- 
  "differential isoform usage"
```

```
iCOBRA::plot_corr(cobraplot2, pointsize = 5, corrtype = "spearman") + theme(legend.position = "bottom") + 
  guides(colour = guide_legend(nrow = 2))
```

```
corr(cobraplot2)[, c("basemethod", "splitval", "PEARSON", "SPEARMAN")]
```

```
##                 basemethod                      splitval   PEARSON  SPEARMAN
## 1            featureCounts no differential isoform usage 0.8152888 0.8516040
## 2            featureCounts    differential isoform usage 0.8359298 0.7265373
## 4     featureCounts_avetxl no differential isoform usage 0.8197844 0.8578961
## 5     featureCounts_avetxl    differential isoform usage 0.8906274 0.7877366
## 7         scaledTPM_salmon no differential isoform usage 0.8022713 0.8332482
## 8         scaledTPM_salmon    differential isoform usage 0.9121384 0.7938213
## 10        simplesum_salmon no differential isoform usage 0.8099607 0.8341344
## 11        simplesum_salmon    differential isoform usage 0.8568606 0.7346615
## 13 simplesum_salmon_avetxl no differential isoform usage 0.8136126 0.8408756
## 14 simplesum_salmon_avetxl    differential isoform usage 0.9107156 0.7826426
```

```
iCOBRA::plot_deviation(cobraplot2) + theme(legend.position = "bottom") + 
  guides(colour = guide_legend(nrow = 2))
```

```
iCOBRA::plot_scatter(cobraplot2, pointsize = 2, doflip = TRUE) + theme(legend.position = "bottom") + 
  guides(colour = guide_legend(nrow = 2)) + geom_abline(intercept = 0, slope = 1) + geom_point(size = 2)
```

## Differential gene expression analysis (limma-voom)

(Back to top)

Given the gene count matrices defined above (corresponding to the **featureCounts**, **simplesum** and **scaledTPM** matrices mentioned in the manuscript), we apply *limma-voom* to perform differential gene expression. For the **simplesum** and **featureCounts** matrices, we also perform the analyses using the offsets derived from the average transcript lengths (**simplesum\_avetxl**).

```
res_sal_simplesum_voom <- diff_expression_voom(counts = salmon_quant$geneCOUNT_sal_simplesum, 
                                               meta = meta, cond_name = "condition", 
                                               sample_name = "sample", 
                                               gene_length_matrix = NULL)
res_sal_simplesum_avetxl_voom <- diff_expression_voom(counts = salmon_quant$geneCOUNT_sal_simplesum, 
                                                      meta = meta, cond_name = "condition", 
                                                      sample_name = "sample", 
                                                      gene_length_matrix = salmon_quant$avetxlength)
res_sal_scaledTPM_voom <- diff_expression_voom(counts = salmon_quant$geneCOUNT_sal_scaledTPM, 
                                               meta = meta, cond_name = "condition", 
                                               sample_name = "sample", 
                                               gene_length_matrix = NULL)
res_fc_voom <- diff_expression_voom(counts = geneCOUNT_fc, 
                                    meta = meta, cond_name = "condition", 
                                    sample_name = "sample", 
                                    gene_length_matrix = NULL)
res_fc_avetxl_voom <- diff_expression_voom(counts = geneCOUNT_fc, 
                                           meta = meta, cond_name = "condition", 
                                           sample_name = "sample", 
                                           gene_length_matrix = salmon_quant$avetxlength)
```

### Comparison of significant genes found with different matrices

(Back to top)

```
cobra_voom <- COBRAData(padj = data.frame(simplesum_salmon = res_sal_simplesum_voom$tt$adj.P.Val, 
                                          row.names = rownames(res_sal_simplesum_voom$tt)))
cobra_voom <- COBRAData(padj = data.frame(scaledTPM_salmon = res_sal_scaledTPM_voom$tt$adj.P.Val, 
                                          row.names = rownames(res_sal_scaledTPM_voom$tt)),
                        object_to_extend = cobra_voom)
cobra_voom <- COBRAData(padj = data.frame(simplesum_salmon_avetxl = res_sal_simplesum_avetxl_voom$tt$adj.P.Val, 
                                          row.names = rownames(res_sal_simplesum_avetxl_voom$tt)),
                        object_to_extend = cobra_voom)
cobra_voom <- COBRAData(padj = data.frame(featureCounts = res_fc_voom$tt$adj.P.Val,
                                          row.names = rownames(res_fc_voom$tt)), 
                        object_to_extend = cobra_voom)
cobra_voom <- COBRAData(padj = data.frame(featureCounts_avetxl = res_fc_avetxl_voom$tt$adj.P.Val,
                                          row.names = rownames(res_fc_avetxl_voom$tt)), 
                        object_to_extend = cobra_voom)
truth_gene <- read.delim(truth_gene_file, header = TRUE, as.is = TRUE, row.names = 1)
cobra_voom <- COBRAData(truth = truth_gene, object_to_extend = cobra_voom)
cobraperf_voom <- calculate_performance(cobra_voom, binary_truth = "status_overall")
```

```
cobraplot1_voom <- prepare_data_for_plot(cobraperf_voom, incltruth = FALSE, 
                                         colorscheme = muted[c(1, 3, 5, 8, 10)])
plot_overlap(cobraplot1_voom, cex = c(1, 0.7, 0.7))
```

### Comparison to truth

(Back to top)

```
## FDR/TPR and FPC
cobraplot2_voom <- prepare_data_for_plot(cobraperf_voom, incltruth = TRUE, 
                                         colorscheme = muted[c(1, 3, 5, 8, 10, 13)],
                                         facetted = FALSE)
fdrtpr(cobraplot2_voom)$fullmethod <- gsub("_overall", "", fdrtpr(cobraplot2_voom)$fullmethod)
fdrtprcurve(cobraplot2_voom)$fullmethod <- gsub("_overall", "", fdrtprcurve(cobraplot2_voom)$fullmethod)
plot_fdrtprcurve(cobraplot2_voom, pointsize = 2, xaxisrange = c(0, 0.8)) + 
  theme(legend.position = "bottom") + guides(colour = guide_legend(nrow = 2))
```

```
fpc(cobraplot2_voom)$fullmethod <- gsub("_overall", "", fpc(cobraplot2_voom)$fullmethod)
plot_fpc(cobraplot2_voom) + plot_theme() + 
  theme(legend.position = "bottom") + guides(colour = guide_legend(nrow = 2))
```

```
## Stratify by diff isoform usage
cobraperf3_voom <- calculate_performance(cobra_voom, binary_truth = "status_overall",
                                         splv = "diffisouse", maxsplit = 4)
cobraplot3_voom <- prepare_data_for_plot(cobraperf3_voom, incltruth = TRUE, 
                                         incloverall = FALSE, 
                                         colorscheme = muted[c(1, 3, 5, 8, 10, 13)])
levels(fdrtpr(cobraplot3_voom)$splitval)[levels(fdrtpr(cobraplot3_voom)$splitval) == "diffisouse:0"] <- "no differential isoform usage"
levels(fdrtpr(cobraplot3_voom)$splitval)[levels(fdrtpr(cobraplot3_voom)$splitval) == "diffisouse:1"] <- "differential isoform usage"
levels(fdrtprcurve(cobraplot3_voom)$splitval)[levels(fdrtprcurve(cobraplot3_voom)$splitval) == "diffisouse:0"] <- "no differential isoform usage"
levels(fdrtprcurve(cobraplot3_voom)$splitval)[levels(fdrtprcurve(cobraplot3_voom)$splitval) == "diffisouse:1"] <- "differential isoform usage"
plot_fdrtprcurve(cobraplot3_voom, pointsize = 2, xaxisrange = c(0, 0.8)) + 
  theme(legend.position = "bottom") + guides(colour = guide_legend(nrow = 2))
```

## Help functions

(Back to top)

```
panel_cor <- function(x, y, digits = 3, cex.cor) {
  ## Panel function to print Pearson and Spearman correlations
  usr <- par("usr")
  on.exit(par(usr))
  par(usr = c(0, 1, 0, 1))
  r1 <- abs(cor(x, y, method = "pearson", use = "complete"))
  txt1 <- format(c(r1, 0.123456789), digits = digits)[1]
  r2 <- abs(cor(x, y, method = "spearman", use = "complete"))
  txt2 <- format(c(r2, 0.123456789), digits = digits)[1]
  text(0.5, 0.35, paste("pearson =", txt1), cex = 1.1)
  text(0.5, 0.65, paste("spearman =", txt2), cex = 1.1)
}
```

```
panel_smooth<-function (x, y, col = "blue", bg = NA, pch = ".", 
                        cex = 0.8, ...) {
  ## Panel function to plot points
  points(x, y, pch = pch, col = col, bg = bg, cex = cex)
}
```

```
plot_theme <- function() {
  ## ggplot2 plotting theme
  theme_grey() +
    theme(legend.position = "right",
          panel.background = element_rect(fill = "white", colour = "black"),
          panel.grid.minor.x = element_blank(),
          panel.grid.minor.y = element_blank(),
          strip.text = element_text(size = 10),
          strip.background = element_rect(fill = NA, colour = "black"),
          axis.text.x = element_text(size = 10),
          axis.text.y = element_text(size = 10),
          axis.title.x = element_text(size = 15),
          axis.title.y = element_text(size = 15),
          plot.title = element_text(colour = "black", size = 20))
}
```

```
calc_lengths_mapping <- function(gtf, cdna_fasta, feature_lengths_file,
                                 tx_gene_file) {
  ## Function to calculate gene and transcript lengths from transcript cDNA 
  ## fasta and gtf file. Also generate mapping between transcript and gene IDs.
  
  suppressPackageStartupMessages(library(GenomicFeatures))
  suppressPackageStartupMessages(library(Biostrings))
  
  ## Gene/transcript lengths ===============================================
  ## Transcripts/genes present in gtf file
  if (!is.null(gtf)) {
    txdb <- makeTxDbFromGFF(gtf, format = "gtf")
    ebg <- exonsBy(txdb, "gene")
    ebt <- exonsBy(txdb, "tx", use.names = TRUE)
    ebg_red <- reduce(ebg)
    gene_length <- sum(width(ebg_red))
    ebt_red <- reduce(ebt)
    tx_length <- sum(width(ebt_red))
  } else {
    tx_length <- c()
    gene_length <- c()
  }  
  
  ## Extend with transcripts from cDNA fasta
  cdna <- readDNAStringSet(gsub("\\.gz", "", cdna_fasta))
  tx_length2 <- width(cdna)
  names(tx_length2) <- sapply(names(cdna), function(i) strsplit(i, " ")[[1]][1])
  tx_length2 <- tx_length2[setdiff(names(tx_length2), names(tx_length))]
  tx_length <- c(tx_length, tx_length2)
  
  ## Gene/transcript mapping ===============================================
  ## Transcripts/genes present in gtf file
  if (!is.null(gtf)) {
    tbg <- transcriptsBy(txdb, "gene")
    tx2gene <- stack(lapply(tbg, function(w) w$tx_name))
    colnames(tx2gene) <- c("transcript", "gene")
  } else {
    tx2gene <- data.frame(transcript = c(), gene = c())
  }  
  
  ## Extend with mappings from cDNA fasta file
  tx <- sapply(names(cdna), function(i) strsplit(i, " ")[[1]][1])
  gn <- sapply(names(cdna), function(i) gsub("gene:", "", strsplit(i, " ")[[1]][4]))
  tx2gene2 <- data.frame(transcript = tx, gene = gn, 
                         stringsAsFactors = FALSE)
  rownames(tx2gene2) <- NULL
  tx2gene2 <- tx2gene2[match(setdiff(tx2gene2$transcript, 
                                     tx2gene$transcript), tx2gene2$transcript), ]
  tx2gene <- rbind(tx2gene, tx2gene2)
  
  gene2tx <- tx2gene[, c("gene", "transcript")]
  
  save(gene_length, tx_length, file = feature_lengths_file)
  save(gene2tx, tx2gene, file = tx_gene_file)
}
```

```
diff_expression_edgeR <- function(counts, meta, cond_name, sample_name, 
                                  gene_length_matrix = NULL) {
  ## Differential expression analysis with edgeR
  
  suppressPackageStartupMessages(library(edgeR))

  ## Prepare DGEList
  counts <- round(counts)
  cts <- counts[rowSums(is.na(counts)) == 0, ]
  cts <- cts[rowSums(cts) != 0, ]
  dge <- 
    DGEList(counts = cts, group = meta[, cond_name][match(colnames(cts), 
                                                          meta[, sample_name])])
  
  ## If average transcript lengths provided, add as offset
  if (!is.null(gene_length_matrix)) {
    egf <- gene_length_matrix[match(rownames(cts), rownames(gene_length_matrix)),
                              match(colnames(cts), colnames(gene_length_matrix))]
    egf <- egf / exp(rowMeans(log(egf)))
    o <- log(calcNormFactors(cts/egf)) + log(colSums(cts/egf))
    dge$offset <- t(t(log(egf)) + o)
  } else {
    dge <- calcNormFactors(dge)
  }
  
  ## Estimate dispersions and fit model
  design <- model.matrix(~dge$samples$group)
  dge <- estimateGLMCommonDisp(dge, design = design)
  dge <- estimateGLMTrendedDisp(dge, design = design)
  dge <- estimateGLMTagwiseDisp(dge, design = design)
  fit <- glmFit(dge, design = design)
  lrt <- glmLRT(fit)
  tt <- topTags(lrt, n = Inf)$table
  return(list(dge = dge, tt = tt))
}
```

```
diff_expression_DESeq2 <- function(txi = NULL, counts, meta, cond_name, 
                                   level1, level2, sample_name) {
  ## Differential expression analysis with DESeq2
  
  suppressPackageStartupMessages(library(DESeq2, 
                                         lib.loc = "/home/charlotte/R/x86_64-pc-linux-gnu-library/3.2"))

  ## If tximport object provided, generate DESeqDataSet from it. Otherwise, 
  ## use the provided count matrix.
  if (!is.null(txi)) {
    txi$counts <- round(txi$counts)
    keep_feat <- rownames(txi$counts[rowSums(is.na(txi$counts)) == 0 & rowSums(txi$counts) != 0, ])
    txi <- lapply(txi, function(w) {
      if (!is.null(dim(w))) w[match(keep_feat, rownames(w)), ]
      else w
      })
    dsd <- DESeqDataSetFromTximport(txi, 
                                    colData = meta[match(colnames(txi$counts), 
                                                         meta[, sample_name]), ],
                                    design = as.formula(paste0("~", cond_name)))
  } else {
    counts <- round(counts)
    cts = counts[rowSums(is.na(counts)) == 0, ]
    cts <- cts[rowSums(cts) != 0, ]
    dsd <- DESeqDataSetFromMatrix(countData = round(cts), 
                                  colData = meta[match(colnames(cts), 
                                                       meta[, sample_name]), ],
                                  design = as.formula(paste0("~", cond_name)))
  }
  
  ## Estimate dispersions and fit model
  dsd <- DESeq(dsd, test = "Wald", fitType = "local", betaPrior = TRUE)
  res <- as.data.frame(results(dsd, contrast = c(cond_name, level2, level1),
                               cooksCutoff = FALSE, independentFiltering = FALSE))
  return(list(dsd = dsd, res = res))
}
```

```
## Slight modification of limma::voom(), allowing offsets to be taken into account 
myvoom <- function (counts, design = NULL, lib.size = NULL, 
                    normalize.method = "none",
                    plot = FALSE, span = 0.5, ...) {
  library(limma)
  out <- list()
  if (is(counts, "DGEList")) {
    out$genes <- counts$genes
    out$targets <- counts$samples
    if (is.null(design) && diff(range(as.numeric(counts$sample$group))) >
        0)
      design <- model.matrix(~group, data = counts$samples)
    if (is.null(lib.size))
      lib.size <- exp(t(counts$offset))   ## modification
    counts <- counts$counts
  }
  else {
    isExpressionSet <- suppressPackageStartupMessages(is(counts,
                                                         "ExpressionSet"))
    if (isExpressionSet) {
      if (length(Biobase::fData(counts)))
        out$genes <- Biobase::fData(counts)
      if (length(Biobase::pData(counts)))
        out$targets <- Biobase::pData(counts)
      counts <- Biobase::exprs(counts)
    }
    else {
      counts <- as.matrix(counts)
    }
  }
  if (is.null(design)) {
    design <- matrix(1, ncol(counts), 1)
    rownames(design) <- colnames(counts)
    colnames(design) <- "GrandMean"
  }
  if (is.null(lib.size))
    lib.size <- colSums(counts)
  y <- t(log2(t(counts + 0.5)/(lib.size + 1) * 1e+06))
  y <- limma::normalizeBetweenArrays(y, method = normalize.method)
  fit <- limma::lmFit(y, design, ...)
  if (is.null(fit$Amean))
    fit$Amean <- rowMeans(y, na.rm = TRUE)
  sx <- fit$Amean + colMeans(log2(lib.size + 1)) - log2(1e+06)
  sy <- sqrt(fit$sigma)
  allzero <- rowSums(counts) == 0
  if (any(allzero)) {
    sx <- sx[!allzero]
    sy <- sy[!allzero]
  }
  l <- lowess(sx, sy, f = span)
  if (plot) {
    plot(sx, sy, xlab = "log2( count size + 0.5 )", ylab = "Sqrt( standard deviation )",
         pch = 16, cex = 0.25)
    title("voom: Mean-variance trend")
    lines(l, col = "red")
  }
  f <- approxfun(l, rule = 2)
  if (fit$rank < ncol(design)) {
    j <- fit$pivot[1:fit$rank]
    fitted.values <- fit$coef[, j, drop = FALSE] %*% t(fit$design[,
                                                                  j, drop = FALSE])
  }
  else {
    fitted.values <- fit$coef %*% t(fit$design)
  }
  fitted.cpm <- 2^fitted.values
  fitted.count <- 1e-06 * t(t(fitted.cpm) * (lib.size + 1))
  fitted.logcount <- log2(fitted.count)
  w <- 1/f(fitted.logcount)^4
  dim(w) <- dim(fitted.logcount)
  out$E <- y
  out$weights <- w
  out$design <- design
  if (is.null(out$targets))
    out$targets <- data.frame(lib.size = lib.size)
  else out$targets$lib.size <- lib.size
  new("EList", out)
}
```

```
diff_expression_voom <- function(counts, meta, cond_name, sample_name, 
                                 gene_length_matrix = NULL) {
  ## Differential expression analysis with voom
  
  suppressPackageStartupMessages(library(limma))
  suppressPackageStartupMessages(library(edgeR))
  
  ## Prepare DGEList
  counts <- round(counts)
  cts <- counts[rowSums(is.na(counts)) == 0, ]
  cts <- cts[rowSums(cts) != 0, ]
  dge <- 
    DGEList(counts = cts, group = meta[, cond_name][match(colnames(cts), 
                                                          meta[, sample_name])])
  design <- model.matrix(~dge$samples$group)
  
  ## If average transcript lengths provided, add as offset
  if (!is.null(gene_length_matrix)) {
    egf <- gene_length_matrix[match(rownames(cts), rownames(gene_length_matrix)),
                              match(colnames(cts), colnames(gene_length_matrix))]
    egf <- egf / exp(rowMeans(log(egf)))
    o <- log(calcNormFactors(cts/egf)) + log(colSums(cts/egf))
    dge$offset <- t(t(log(egf)) + o)
    v <- myvoom(dge, design = design)
  } else {
    dge <- calcNormFactors(dge)
    v <- voom(dge, design = design)
  }
  
  ## Transform and fit model
  fit <- lmFit(v, design = design)
  fit <- eBayes(fit)
  tt <- topTable(fit, n = Inf)
  return(list(dge = dge, tt = tt))
}
```

## Session info

(Back to top)

```
sessionInfo()
```

```
## R version 3.2.2 (2015-08-14)
## Platform: x86_64-pc-linux-gnu (64-bit)
## Running under: Ubuntu 14.04.4 LTS
## 
## locale:
##  [1] LC_CTYPE=C                 LC_NUMERIC=C               LC_TIME=en_CA.UTF-8       
##  [4] LC_COLLATE=en_CA.UTF-8     LC_MONETARY=en_CA.UTF-8    LC_MESSAGES=en_CA.UTF-8   
##  [7] LC_PAPER=en_CA.UTF-8       LC_NAME=C                  LC_ADDRESS=C              
## [10] LC_TELEPHONE=C             LC_MEASUREMENT=en_CA.UTF-8 LC_IDENTIFICATION=C       
## 
## attached base packages:
## [1] parallel  stats4    stats     graphics  grDevices utils     datasets  methods   base     
## 
## other attached packages:
##  [1] biomaRt_2.26.1             DEXSeq_1.16.7              DESeq2_1.11.6             
##  [4] RcppArmadillo_0.6.400.2.2  Rcpp_0.12.2                SummarizedExperiment_1.0.2
##  [7] Biobase_2.30.0             GenomicRanges_1.22.3       GenomeInfoDb_1.6.2        
## [10] IRanges_2.4.6              S4Vectors_0.8.6            BiocGenerics_0.16.1       
## [13] BiocParallel_1.4.3         iCOBRA_0.99.7              Hmisc_3.17-1              
## [16] Formula_1.2-1              survival_2.38-3            lattice_0.20-33           
## [19] ggplot2_2.0.0              tximport_0.0.7             dplyr_0.4.3               
## [22] edgeR_3.12.0               limma_3.26.3              
## 
## loaded via a namespace (and not attached):
##  [1] splines_3.2.2        gtools_3.5.0         shiny_0.13.0         assertthat_0.1      
##  [5] statmod_1.4.23       latticeExtra_0.6-26  Rsamtools_1.22.0     yaml_2.1.13         
##  [9] RSQLite_1.0.0        digest_0.6.9         RColorBrewer_1.1-2   XVector_0.10.0      
## [13] colorspace_1.2-6     htmltools_0.3        httpuv_1.3.3         plyr_1.8.3          
## [17] XML_3.98-1.3         genefilter_1.52.0    zlibbioc_1.16.0      xtable_1.8-0        
## [21] scales_0.3.0         gdata_2.17.0         annotate_1.48.0      DT_0.1              
## [25] ROCR_1.0-7           lazyeval_0.1.10      nnet_7.3-11          magrittr_1.5        
## [29] mime_0.4             evaluate_0.8         gplots_2.17.0        hwriter_1.3.2       
## [33] foreign_0.8-66       shinydashboard_0.5.1 tools_3.2.2          formatR_1.2.1       
## [37] stringr_1.0.0        locfit_1.5-9.1       munsell_0.4.2        cluster_2.0.3       
## [41] Biostrings_2.38.3    AnnotationDbi_1.32.3 lambda.r_1.1.7       caTools_1.17.1      
## [45] RCurl_1.95-4.7       futile.logger_1.4.1  grid_3.2.2           htmlwidgets_0.5     
## [49] labeling_0.3         bitops_1.0-6         shinyBS_0.61         rmarkdown_0.9.2     
## [53] gtable_0.1.2         DBI_0.3.1            reshape2_1.4.1       R6_2.1.1            
## [57] gridExtra_2.0.0      knitr_1.12.3         futile.options_1.0.0 KernSmooth_2.23-15  
## [61] stringi_1.0-1        geneplotter_1.48.0   rpart_4.1-10         acepack_1.3-3.3
```
