# Supplementary material for: Differential analyses for RNA-seq: transcript-level estimates improve gene-level inferences
Source: F1000Res. 2016 Feb 29;4:1521. Originally published 2015 Dec 30. [Version 2] doi: 10.12688/f1000research.7563.2 (PMC4712774; doi:10.12688/f1000research.7563.2)
Supplement: Data set 3 — http://dx.doi.org/10.5256/f1000research.7563.d114724 Data set 3 (html) contains all the R code that was used to perform the analyses and generate the figures for the Bottomly data set 32. [file f1000research-4-8770-s0002.tgz › 5c81409a-b7af-47a2-9a47-d46fec5a7603_bottomly-quantification.html]

Bottomly


# Bottomly

- Preparation
  - Reference directories and packages
  - FASTQ file download
  - Metadata definition
  - Reference file preparation and index building
  - Definition of gene-to-transcript mapping
  - Read alignment
  - featureCounts read counting
  - featureCounts read counting with fractional assignment of multimapping reads
- Salmon abundance quantification
  - Summarization of Salmon results and offset estimation
    - Estimation of coefficients of variation for all samples
- Variability of transcript and gene TPMs
- Correlation between gene count estimates
  - All genes
  - Only genes with positive estimates with all methods
  - Example of genes with a big difference between simple sum and scaled TPM:
- Differential expression analysis (edgeR)
  - Diagnostics
  - Comparison of significant genes found with different matrices
  - Comparison of logFC estimates - all methods
  - Comparison of logFC estimates - simplesum vs scaledTPM
- DTU analysis on Salmon counts, with DEXSeq
- Differential transcript expression (DTE) analysis on Salmon counts
  - Comparison of significant genes from DTE, DTU, DGE (scaled TPM)
- Differential expression analysis (DESeq2)
  - Diagnostics
  - Comparison of significant genes found with different matrices
  - Comparison of logFC estimates - all methods
  - Comparison of logFC estimates - simplesum vs scaledTPM
- Help functions
- Session info

This report outlines the analysis of a subset of the Bottomly data set, consisting of 11 replicates of striatal tissue from DBA/2J mice, and 10 replicates of striatal tissue from C57BL/6J mice.

## Preparation

(Back to top)

### Reference directories and packages

(Back to top)

```
basedir <- "/home/charlotte/gene_vs_tx_quantification"
refdir <- "/home/Shared/data/annotation"
suppressPackageStartupMessages(library(Rsubread))
suppressPackageStartupMessages(library(tximport))
suppressPackageStartupMessages(library(iCOBRA))
suppressPackageStartupMessages(library(ggplot2))
suppressPackageStartupMessages(library(dplyr))
suppressPackageStartupMessages(library(BiocParallel))
suppressPackageStartupMessages(library(DESeq2, lib.loc = "/home/charlotte/R/x86_64-pc-linux-gnu-library/3.2"))
suppressPackageStartupMessages(library(DEXSeq))
```

### FASTQ file download

(Back to top)

The code below downloads the FASTQ files for the six samples from the SRA.

```
fastq_files <- c(paste0("ftp://ftp.ddbj.nig.ac.jp/ddbj_database/dra/", 
                        "fastq/SRA026/SRA026846/SRX0334", 
                        setdiff(72:94, c(77, 87)), "/SRR0992", 
                        23:43, ".fastq.bz2"))
for (fq in fastq_files) {
  if (!file.exists(paste0(basedir, "/data/bottomly/fastq/", basename(fq)))) {
    cmd <- paste0("wget -P ", basedir, "/data/bottomly/fastq ", fq)
    message(cmd)
    system(cmd)
  } else {
    message(paste0(fq, " is already downloaded."))
  }
}
```

```
## ftp://ftp.ddbj.nig.ac.jp/ddbj_database/dra/fastq/SRA026/SRA026846/SRX033472/SRR099223.fastq.bz2 is already downloaded.
## ftp://ftp.ddbj.nig.ac.jp/ddbj_database/dra/fastq/SRA026/SRA026846/SRX033473/SRR099224.fastq.bz2 is already downloaded.
## ftp://ftp.ddbj.nig.ac.jp/ddbj_database/dra/fastq/SRA026/SRA026846/SRX033474/SRR099225.fastq.bz2 is already downloaded.
## ftp://ftp.ddbj.nig.ac.jp/ddbj_database/dra/fastq/SRA026/SRA026846/SRX033475/SRR099226.fastq.bz2 is already downloaded.
## ftp://ftp.ddbj.nig.ac.jp/ddbj_database/dra/fastq/SRA026/SRA026846/SRX033476/SRR099227.fastq.bz2 is already downloaded.
## ftp://ftp.ddbj.nig.ac.jp/ddbj_database/dra/fastq/SRA026/SRA026846/SRX033478/SRR099228.fastq.bz2 is already downloaded.
## ftp://ftp.ddbj.nig.ac.jp/ddbj_database/dra/fastq/SRA026/SRA026846/SRX033479/SRR099229.fastq.bz2 is already downloaded.
## ftp://ftp.ddbj.nig.ac.jp/ddbj_database/dra/fastq/SRA026/SRA026846/SRX033480/SRR099230.fastq.bz2 is already downloaded.
## ftp://ftp.ddbj.nig.ac.jp/ddbj_database/dra/fastq/SRA026/SRA026846/SRX033481/SRR099231.fastq.bz2 is already downloaded.
## ftp://ftp.ddbj.nig.ac.jp/ddbj_database/dra/fastq/SRA026/SRA026846/SRX033482/SRR099232.fastq.bz2 is already downloaded.
## ftp://ftp.ddbj.nig.ac.jp/ddbj_database/dra/fastq/SRA026/SRA026846/SRX033483/SRR099233.fastq.bz2 is already downloaded.
## ftp://ftp.ddbj.nig.ac.jp/ddbj_database/dra/fastq/SRA026/SRA026846/SRX033484/SRR099234.fastq.bz2 is already downloaded.
## ftp://ftp.ddbj.nig.ac.jp/ddbj_database/dra/fastq/SRA026/SRA026846/SRX033485/SRR099235.fastq.bz2 is already downloaded.
## ftp://ftp.ddbj.nig.ac.jp/ddbj_database/dra/fastq/SRA026/SRA026846/SRX033486/SRR099236.fastq.bz2 is already downloaded.
## ftp://ftp.ddbj.nig.ac.jp/ddbj_database/dra/fastq/SRA026/SRA026846/SRX033488/SRR099237.fastq.bz2 is already downloaded.
## ftp://ftp.ddbj.nig.ac.jp/ddbj_database/dra/fastq/SRA026/SRA026846/SRX033489/SRR099238.fastq.bz2 is already downloaded.
## ftp://ftp.ddbj.nig.ac.jp/ddbj_database/dra/fastq/SRA026/SRA026846/SRX033490/SRR099239.fastq.bz2 is already downloaded.
## ftp://ftp.ddbj.nig.ac.jp/ddbj_database/dra/fastq/SRA026/SRA026846/SRX033491/SRR099240.fastq.bz2 is already downloaded.
## ftp://ftp.ddbj.nig.ac.jp/ddbj_database/dra/fastq/SRA026/SRA026846/SRX033492/SRR099241.fastq.bz2 is already downloaded.
## ftp://ftp.ddbj.nig.ac.jp/ddbj_database/dra/fastq/SRA026/SRA026846/SRX033493/SRR099242.fastq.bz2 is already downloaded.
## ftp://ftp.ddbj.nig.ac.jp/ddbj_database/dra/fastq/SRA026/SRA026846/SRX033494/SRR099243.fastq.bz2 is already downloaded.
```

### Metadata definition

(Back to top)

```
meta <- read.delim(paste0(basedir, "/data/bottomly/bottomly_phenodata.txt"), 
                   header = TRUE, as.is = TRUE)
rownames(meta) <- meta$srr.id
meta
```

```
##           sample.id    srr.id num.tech.reps   strain experiment.number lane.number
## SRR099230 SRX033480 SRR099230             1 C57BL/6J                 6           1
## SRR099237 SRX033488 SRR099237             1 C57BL/6J                 7           1
## SRR099231 SRX033481 SRR099231             1 C57BL/6J                 6           2
## SRR099238 SRX033489 SRR099238             1 C57BL/6J                 7           2
## SRR099232 SRX033482 SRR099232             1 C57BL/6J                 6           3
## SRR099239 SRX033490 SRR099239             1 C57BL/6J                 7           3
## SRR099233 SRX033483 SRR099233             1 C57BL/6J                 6           5
## SRR099227 SRX033476 SRR099227             1 C57BL/6J                 4           6
## SRR099228 SRX033478 SRR099228             1 C57BL/6J                 4           7
## SRR099229 SRX033479 SRR099229             1 C57BL/6J                 4           8
## SRR099223 SRX033472 SRR099223             1   DBA/2J                 4           1
## SRR099224 SRX033473 SRR099224             1   DBA/2J                 4           2
## SRR099225 SRX033474 SRR099225             1   DBA/2J                 4           3
## SRR099226 SRX033475 SRR099226             1   DBA/2J                 4           5
## SRR099240 SRX033491 SRR099240             1   DBA/2J                 7           5
## SRR099234 SRX033484 SRR099234             1   DBA/2J                 6           6
## SRR099241 SRX033492 SRR099241             1   DBA/2J                 7           6
## SRR099235 SRX033485 SRR099235             1   DBA/2J                 6           7
## SRR099242 SRX033493 SRR099242             1   DBA/2J                 7           7
## SRR099236 SRX033486 SRR099236             1   DBA/2J                 6           8
## SRR099243 SRX033494 SRR099243             1   DBA/2J                 7           8
```

### Reference file preparation and index building

(Back to top)

```
cdna_fasta <- paste0(refdir, "/Mouse/Ensembl_GRCm38.82/cDNA/Mus_musculus.GRCm38.cdna.all.fa.gz")
salmon_index <- paste0(basedir, "/annotation/Mouse_Ensembl_GRCm38.82/salmon_index/Mus_musculus.GRCm38.82.gtf.sidx")
genome_fasta <- paste0(refdir, "/Mouse/Ensembl_GRCm38.82/genome/Mus_musculus.GRCm38.dna.toplevel.fa")
gtf <- paste0(refdir, "/Mouse/Ensembl_GRCm38.82/GTF/Mus_musculus.GRCm38.82.gtf")
star_index_dir <- paste0(basedir, "/annotation/Mouse_Ensembl_GRCm38.82/STAR_125")

## Download reference files if needed
############################### GENOME FASTA ##############################
if (!file.exists(genome_fasta)) {
  cmd <- paste0("wget -P ", refdir, "/Mouse/Ensembl_GRCm38.82/genome ", 
                "ftp://ftp.ensembl.org/pub/release-82/fasta/mus_musculus/", 
                "dna/Mus_musculus.GRCm38.dna.toplevel.fa.gz")
  message(cmd)
  system(cmd)
  cmd <- paste0("gunzip ", genome_fasta, ".gz")
  message(cmd)
  system(cmd)
} else {
  message(paste0("Reference genome is already downloaded."))
}
```

```
## Reference genome is already downloaded.
```

```
############################### cDNA FASTA ################################
if (!file.exists(cdna_fasta)) {
  cmd <- paste0("wget -P ", refdir, "/Mouse/Ensembl_GRCm38.82/cDNA ", 
                "ftp://ftp.ensembl.org/pub/release-82/fasta/mus_musculus/", 
                "cdna/Mus_musculus.GRCm38.cdna.all.fa.gz")
  message(cmd)
  system(cmd)
  cmd <- paste0("gunzip -c ", cdna_fasta, "> ", gsub("\\.gz$", "", cdna_fasta))
  message(cmd)
  system(cmd)
} else {
  message(paste0("Reference cDNA fasta is already downloaded."))
}
```

```
## Reference cDNA fasta is already downloaded.
```

```
#################################### GTF ###################################
if (!file.exists(gtf)) {
  cmd <- paste0("wget -P ", refdir, "/Mouse/Ensembl_GRCm38.82/GTF ", 
                "ftp://ftp.ensembl.org/pub/release-82/gtf/mus_musculus/", 
                "Mus_musculus.GRCm38.82.gtf.gz")
  message(cmd)
  system(cmd)
  cmd <- paste0("gunzip ", gtf, ".gz")
  message(cmd)
  system(cmd)
} else {
  message(paste0("Reference GTF file is already downloaded."))
}
```

```
## Reference GTF file is already downloaded.
```

```
## Build STAR index
cmd <- paste0("STAR --runMode genomeGenerate --runThreadN 10 ",
              "--genomeDir ", star_index_dir, " --genomeFastaFiles ",
              genome_fasta, " --sjdbGTFfile ", gtf, " --sjdbOverhang 125")
message(cmd)
```

```
## STAR --runMode genomeGenerate --runThreadN 10 --genomeDir /home/charlotte/gene_vs_tx_quantification/annotation/Mouse_Ensembl_GRCm38.82/STAR_125 --genomeFastaFiles /home/Shared/data/annotation/Mouse/Ensembl_GRCm38.82/genome/Mus_musculus.GRCm38.dna.toplevel.fa --sjdbGTFfile /home/Shared/data/annotation/Mouse/Ensembl_GRCm38.82/GTF/Mus_musculus.GRCm38.82.gtf --sjdbOverhang 125
```

```
if (!file.exists(paste0(star_index_dir, "/SA"))) {
  system(cmd)
} else {
  message(paste0("STAR index already exists."))
}
```

```
## STAR index already exists.
```

```
## Build Salmon index
cmd <- paste("salmon index -i", salmon_index, "-t", gsub("\\.gz$", "", cdna_fasta), "-p 5 --type quasi")
message(cmd)
```

```
## salmon index -i /home/charlotte/gene_vs_tx_quantification/annotation/Mouse_Ensembl_GRCm38.82/salmon_index/Mus_musculus.GRCm38.82.gtf.sidx -t /home/Shared/data/annotation/Mouse/Ensembl_GRCm38.82/cDNA/Mus_musculus.GRCm38.cdna.all.fa -p 5 --type quasi
```

```
if (!file.exists(paste0(salmon_index, "/hash.bin"))) {
  system(cmd)
} else {
  message("Salmon index already exists.")
}
```

```
## Salmon index already exists.
```

```
feature_lengths_file <- paste0(basedir, "/annotation/Mouse_Ensembl_GRCm38.82/feature_lengths.Rdata")
tx_gene_file <- paste0(basedir, "/annotation/Mouse_Ensembl_GRCm38.82/tx_gene_map.Rdata")
salmon_basedir <- paste0(basedir, "/quantifications/bottomly/salmon")
cv_salmon_file <- paste0(basedir, "/quantifications/bottomly/salmon/cv_salmon.Rdata")
```

### Definition of gene-to-transcript mapping

(Back to top)

```
## Calculate gene and transcript lengths, get gene-transcript mapping
if (!file.exists(feature_lengths_file)) {
  calc_lengths_mapping(gtf = gtf, cdna_fasta = cdna_fasta, 
                       feature_lengths_file = feature_lengths_file, 
                       tx_gene_file = tx_gene_file) 
} else {
  message("feature lengths and tx-to-gene map already calculated.")
}
```

```
## feature lengths and tx-to-gene map already calculated.
```

```
load(feature_lengths_file)
load(tx_gene_file)
```

### Read alignment

(Back to top)

```
fqfiles <- list.files(paste0(basedir, "/data/bottomly/fastq"), full.names = TRUE)
for (fq in fqfiles) {
  if (!file.exists(paste0(basedir, "/data/bottomly/bam/", 
                          gsub("\\.fastq.bz2", "", basename(fq)), "/Aligned.out.bam"))) {
    cmd <- paste0("STAR --genomeDir ", star_index_dir, " --readFilesIn ",
                  fq, " --runThreadN 10 ",
                  "--outFileNamePrefix ", basedir, "/data/bottomly/bam/", 
                  gsub("\\.fastq.bz2", "", basename(fq)), "/", 
                  " --outSAMtype BAM Unsorted --readFilesCommand bunzip2 -c")
    cat(cmd, "\n")
    system(cmd)
  } else {
    message(paste0("Sample ", basename(fq), " is already aligned."))
  }
}
```

```
## Sample SRR099223.fastq.bz2 is already aligned.
## Sample SRR099224.fastq.bz2 is already aligned.
## Sample SRR099225.fastq.bz2 is already aligned.
## Sample SRR099226.fastq.bz2 is already aligned.
## Sample SRR099227.fastq.bz2 is already aligned.
## Sample SRR099228.fastq.bz2 is already aligned.
## Sample SRR099229.fastq.bz2 is already aligned.
## Sample SRR099230.fastq.bz2 is already aligned.
## Sample SRR099231.fastq.bz2 is already aligned.
## Sample SRR099232.fastq.bz2 is already aligned.
## Sample SRR099233.fastq.bz2 is already aligned.
## Sample SRR099234.fastq.bz2 is already aligned.
## Sample SRR099235.fastq.bz2 is already aligned.
## Sample SRR099236.fastq.bz2 is already aligned.
## Sample SRR099237.fastq.bz2 is already aligned.
## Sample SRR099238.fastq.bz2 is already aligned.
## Sample SRR099239.fastq.bz2 is already aligned.
## Sample SRR099240.fastq.bz2 is already aligned.
## Sample SRR099241.fastq.bz2 is already aligned.
## Sample SRR099242.fastq.bz2 is already aligned.
## Sample SRR099243.fastq.bz2 is already aligned.
```

### featureCounts read counting

(Back to top)

```
bamdir <- list.files(paste0(basedir, "/data/bottomly/bam"), full.names = TRUE)
bamdir <- bamdir[file.info(bamdir)$isdir]
bamfiles <- paste0(bamdir, "/Aligned.out.bam")
names(bamfiles) <- basename(bamdir)

fc_bottomly_file <- paste0(basedir, "/quantifications/bottomly/featureCounts/fc_bottomly.Rdata")
if (!file.exists(fc_bottomly_file)) {
  fc_bottomly <- Rsubread::featureCounts(
    files = bamfiles,
    annot.ext = gtf,
    isGTFAnnotationFile = TRUE,
    GTF.featureType = "exon",
    GTF.attrType = "gene_id",
    useMetaFeatures = TRUE,
    isPairedEnd = FALSE, nthreads = 5, 
    strandSpecific = 0, countMultiMappingReads = FALSE
  )
  colnames(fc_bottomly$counts) <- sapply(colnames(fc_bottomly$counts), function(w) {
    s <- strsplit(w, "\\.")[[1]]
    s[grep("SRR", s)]
  })
  colnames(fc_bottomly$stat) <- sapply(colnames(fc_bottomly$stat), function(w) {
    s <- strsplit(w, "\\.")[[1]]
    s[grep("SRR", s)]
  })
  save(fc_bottomly, file = fc_bottomly_file)
} else {
  message("Loading previously saved file with featureCounts quantifications.")
  load(fc_bottomly_file)
}
```

```
## Loading previously saved file with featureCounts quantifications.
```

### featureCounts read counting with fractional assignment of multimapping reads

(Back to top)

```
bamdir <- list.files(paste0(basedir, "/data/bottomly/bam"), full.names = TRUE)
bamdir <- bamdir[file.info(bamdir)$isdir]
bamfiles <- paste0(bamdir, "/Aligned.out.bam")
names(bamfiles) <- basename(bamdir)

fc_bottomly_file_fracmm <- paste0(basedir, "/quantifications/bottomly/featureCounts/fc_bottomly_fracMM.Rdata")
if (!file.exists(fc_bottomly_file_fracmm)) {
  fc_bottomly_fracmm <- Rsubread::featureCounts(
    files = bamfiles,
    annot.ext = gtf,
    isGTFAnnotationFile = TRUE,
    GTF.featureType = "exon",
    GTF.attrType = "gene_id",
    useMetaFeatures = TRUE,
    isPairedEnd = FALSE, nthreads = 5, 
    strandSpecific = 0, countMultiMappingReads = TRUE,
    fraction = TRUE
  )
  colnames(fc_bottomly_fracmm$counts) <- sapply(colnames(fc_bottomly_fracmm$counts), function(w) {
    s <- strsplit(w, "\\.")[[1]]
    s[grep("SRR", s)]
  })
  colnames(fc_bottomly_fracmm$stat) <- sapply(colnames(fc_bottomly_fracmm$stat), function(w) {
    s <- strsplit(w, "\\.")[[1]]
    s[grep("SRR", s)]
  })
  save(fc_bottomly_fracmm, file = fc_bottomly_file_fracmm)
} else {
  message("Loading previously saved file with featureCounts quantifications.")
  load(fc_bottomly_file_fracmm)
}
```

```
## Loading previously saved file with featureCounts quantifications.
```

## Salmon abundance quantification

(Back to top)

```
fqs <- list.files(paste0(basedir, "/data/bottomly/fastq"), full.names = TRUE)
names(fqs) <- gsub("\\.fastq.bz2", "", basename(fqs))

for (i in 1:length(fqs)) {
  if (!file.exists(paste0(salmon_basedir, "/", names(fqs)[i], "/quant.sf"))) {
    cmd <- sprintf("bash -c 'salmon quant -i %s -l U -r %s -p 5 -o %s --numBootstraps=30'",
                   salmon_index,
                   paste0("<(bzcat ", fqs[i], ")"),
                   paste0(salmon_basedir, "/", names(fqs)[i]))
    cat(cmd, "\n")
    system(cmd)
  } else {
    cat("Salmon results for", names(fqs)[i], "already exist.\n")
  }
}
```

```
## Salmon results for SRR099223 already exist.
## Salmon results for SRR099224 already exist.
## Salmon results for SRR099225 already exist.
## Salmon results for SRR099226 already exist.
## Salmon results for SRR099227 already exist.
## Salmon results for SRR099228 already exist.
## Salmon results for SRR099229 already exist.
## Salmon results for SRR099230 already exist.
## Salmon results for SRR099231 already exist.
## Salmon results for SRR099232 already exist.
## Salmon results for SRR099233 already exist.
## Salmon results for SRR099234 already exist.
## Salmon results for SRR099235 already exist.
## Salmon results for SRR099236 already exist.
## Salmon results for SRR099237 already exist.
## Salmon results for SRR099238 already exist.
## Salmon results for SRR099239 already exist.
## Salmon results for SRR099240 already exist.
## Salmon results for SRR099241 already exist.
## Salmon results for SRR099242 already exist.
## Salmon results for SRR099243 already exist.
```

### Summarization of Salmon results and offset estimation

(Back to top)

We use the `tximport` package (https://github.com/mikelove/tximport) to generate count matrices and offset matrices (average transcript lengths) from the Salmon transcript-level estimates. We generate two different count matrices (**simplesum** and **scaledTPM**), and additionally create offsets to be used with the **simplesum** matrix.

```
salmon_files <- list.files(salmon_basedir, pattern = "SRR", full.names = TRUE)
salmon_files <- salmon_files[file.info(salmon_files)$isdir]
salmon_files <- paste0(salmon_files, "/quant.sf")
salmon_files <- salmon_files[file.exists(salmon_files)]
names(salmon_files) <- basename(gsub("/quant.sf", "", salmon_files))
txi_salmonsimplesum <- tximport(files = salmon_files, type = "salmon", txIn = TRUE,
                                txOut = FALSE, countsFromAbundance = "no", 
                                gene2tx = gene2tx)
```

```
## reading in files
## 1 2 3 4 5 6 7 8 9 10 11 12 13 14 15 16 17 18 19 20 21 
## summarizing abundance
## summarizing counts
## summarizing length
```

```
txi_salmonscaledtpm <- tximport(files = salmon_files, type = "salmon", txIn = TRUE,
                                txOut = FALSE, countsFromAbundance = "scaledTPM", 
                                gene2tx = gene2tx)
```

```
## reading in files
## 1 2 3 4 5 6 7 8 9 10 11 12 13 14 15 16 17 18 19 20 21 
## summarizing abundance
## summarizing counts
## summarizing length
```

```
txi_salmontx <- tximport(files = salmon_files, type = "salmon", txIn = TRUE,
                         txOut = TRUE, countsFromAbundance = "no", gene2tx = gene2tx)
```

```
## reading in files
## 1 2 3 4 5 6 7 8 9 10 11 12 13 14 15 16 17 18 19 20 21
```

```
salmon_quant <- list(geneCOUNT_sal_simplesum = txi_salmonsimplesum$counts,
                     geneCOUNT_sal_scaledTPM = txi_salmonscaledtpm$counts,
                     avetxlength = txi_salmonsimplesum$length,
                     geneTPM_sal = txi_salmonsimplesum$abundance,
                     txTPM_sal = txi_salmontx$abundance,
                     txCOUNT_sal = txi_salmontx$counts,
                     txi_salmonsimplesum = txi_salmonsimplesum,
                     txi_salmonscaledtpm = txi_salmonscaledtpm,
                     txi_salmontx = txi_salmontx)
```

#### Estimation of coefficients of variation for all samples

(Back to top)

```
## Transcript
salmon_dirs <- list.files(salmon_basedir, pattern = "SRR", full.names = TRUE)
salmon_dirs <- salmon_dirs[file.info(salmon_dirs)$isdir]
names(salmon_dirs) <- basename(salmon_dirs)

if (!file.exists(cv_salmon_file)) {
  CV_tx_salmon <- as.data.frame(sapply(salmon_dirs, function(w) {
    tmpdf <- as.data.frame(t(read.delim(paste0(w, "/quant_bootstraps.sf"), 
                                        skip = 10, header = TRUE, as.is = TRUE)))
    tmpcv <- apply(tmpdf, 1, sd)/apply(tmpdf, 1, mean)
    tmpcv[is.na(tmpcv)] <- 0
    names(tmpcv) <- rownames(tmpdf)
    tmpcv
  }))
  CV_tx_salmon$mean_cv <- apply(CV_tx_salmon[, names(salmon_dirs)], 1, mean)
  CV_tx_salmon$min_cv <- apply(CV_tx_salmon[, names(salmon_dirs)], 1, min)
  CV_tx_salmon$max_cv <- apply(CV_tx_salmon[, names(salmon_dirs)], 1, max)
  CV_tx_salmon$median_cv <- apply(CV_tx_salmon[, names(salmon_dirs)], 1, median)
  
  ## Gene
  CV_gene_salmon <- as.data.frame(sapply(salmon_dirs, function(w) {
    tmpdf <- as.data.frame(t(read.delim(paste0(w, "/quant_bootstraps.sf"), 
                                        skip = 10, header = TRUE, as.is = TRUE)))
    tmpdf$gene <- tx2gene$gene[match(rownames(tmpdf), tx2gene$transcript)]
    tmpdf <- tmpdf %>% group_by(gene) %>% summarise_each(funs(sum)) %>% as.data.frame
    rownames(tmpdf) <- tmpdf$gene
    tmpdf$gene <- NULL
    tmpcv <- apply(tmpdf, 1, sd)/apply(tmpdf, 1, mean)
    tmpcv[is.na(tmpcv)] <- 0
    names(tmpcv) <- rownames(tmpdf)
    tmpcv
  }))
  CV_gene_salmon$mean_cv <- apply(CV_gene_salmon[, names(salmon_dirs)], 1, mean)
  CV_gene_salmon$min_cv <- apply(CV_gene_salmon[, names(salmon_dirs)], 1, min)
  CV_gene_salmon$max_cv <- apply(CV_gene_salmon[, names(salmon_dirs)], 1, max)
  CV_gene_salmon$median_cv <- apply(CV_gene_salmon[, names(salmon_dirs)], 1, median)
  
  save(CV_gene_salmon, CV_tx_salmon, file = cv_salmon_file)
} else {
  message("Salmon CVs already exist.")
}
```

```
## Salmon CVs already exist.
```

```
load(cv_salmon_file)
```

## Variability of transcript and gene TPMs

(Back to top)

```
cvs_sal <- data.frame(cvs = c(CV_tx_salmon$SRR099223, CV_gene_salmon$SRR099223), 
                      lev = rep(c("transcript", "gene"), c(nrow(CV_tx_salmon), nrow(CV_gene_salmon))))
ggplot(cvs_sal, aes(x = log10(cvs + 0.001), col = lev)) + 
  geom_line(stat = "density", size = 2) + 
  ggtitle("") + 
  xlab("log10(coefficient of variation + 0.001)") + ylab("density") + 
  scale_colour_manual(values = c("red", "blue"), name = "") + 
  plot_theme()
```

## Correlation between gene count estimates

(Back to top)

### All genes

(Back to top)

```
load(fc_bottomly_file)
load(fc_bottomly_file_fracmm)
geneCOUNT_fc <- fc_bottomly$counts[match(rownames(salmon_quant$geneCOUNT_sal_simplesum),
                                         rownames(fc_bottomly$counts)), ]
geneCOUNT_fcmm <- fc_bottomly_fracmm$counts[match(rownames(salmon_quant$geneCOUNT_sal_simplesum),
                                                  rownames(fc_bottomly_fracmm$counts)), ]
stopifnot(all(rownames(salmon_quant$geneCOUNT_sal_simplesum) == 
                rownames(salmon_quant$geneCOUNT_sal_scaledTPM)))
SRR099223 <- cbind(simplesum_salmon = salmon_quant$geneCOUNT_sal_simplesum[, "SRR099223"], 
                   scaledTPM_salmon = salmon_quant$geneCOUNT_sal_scaledTPM[, "SRR099223"],
                   featureCounts = geneCOUNT_fc[, "SRR099223"],
                   featureCountsMM = geneCOUNT_fcmm[, "SRR099223"])
rownames(SRR099223) <- rownames(salmon_quant$geneCOUNT_sal_simplesum)
SRR099223 <- log2(SRR099223 + 1)
pairs(SRR099223, upper.panel = panel_smooth, lower.panel = panel_cor)
```

### Only genes with positive estimates with all methods

(Back to top)

```
pairs(SRR099223[which(rowSums(SRR099223 <= 0) == 0), ], upper.panel = panel_smooth,
      lower.panel = panel_cor)
```

### Example of genes with a big difference between simple sum and scaled TPM:

(Back to top)

```
idx <- rownames(SRR099223)[order(abs(SRR099223[, "simplesum_salmon"] - 
                                       SRR099223[, "scaledTPM_salmon"]), decreasing = TRUE)[1:5]]
2^SRR099223[idx, ] - 1
```

```
##                    simplesum_salmon scaledTPM_salmon featureCounts featureCountsMM
## ENSMUSG00000064356        1208.9800        60104.854             0          387.50
## ENSMUSG00000106666          38.4926         1913.670             0           29.00
## ENSMUSG00000070834          28.5044         1417.112             0           14.50
## ENSMUSG00000082127          51.9810         2544.111             0            1.83
## ENSMUSG00000074846          23.5224         1169.421             0            0.00
```

```
subset(tx2gene, gene %in% idx)
```

```
##                transcript               gene
## 74708  ENSMUST00000082407 ENSMUSG00000064356
## 78060  ENSMUST00000118674 ENSMUSG00000070834
## 80558  ENSMUST00000188689 ENSMUSG00000074846
## 85443  ENSMUST00000121365 ENSMUSG00000082127
## 110140 ENSMUST00000199590 ENSMUSG00000106666
```

## Differential expression analysis (edgeR)

(Back to top)

Given the gene count matrices defined above (corresponding to the **featureCounts**, **simplesum** and **scaledTPM** matrices mentioned in the manuscript), we apply *edgeR* (see below for *DESeq2* results) to perform differential gene expression. For the **simplesum** and **featureCounts** matrices, we also perform the analyses using the offsets derived from the average transcript lengths (**simplesum\_avetxl**).

```
res_sal_simplesum_edgeR <- diff_expression_edgeR(counts = salmon_quant$geneCOUNT_sal_simplesum, 
                                                 meta = meta, cond_name = "strain", 
                                                 sample_name = "srr.id", 
                                                 gene_length_matrix = NULL)
res_sal_simplesum_avetxl_edgeR <- diff_expression_edgeR(counts = salmon_quant$geneCOUNT_sal_simplesum, 
                                                        meta = meta, cond_name = "strain", 
                                                        sample_name = "srr.id", 
                                                        gene_length_matrix = salmon_quant$avetxlength)
res_sal_scaledTPM_edgeR <- diff_expression_edgeR(counts = salmon_quant$geneCOUNT_sal_scaledTPM, 
                                                 meta = meta, cond_name = "strain", 
                                                 sample_name = "srr.id", 
                                                 gene_length_matrix = NULL)
res_fc_edgeR <- diff_expression_edgeR(counts = geneCOUNT_fc, 
                                      meta = meta, cond_name = "strain", 
                                      sample_name = "srr.id", 
                                      gene_length_matrix = NULL)
res_fc_avetxl_edgeR <- diff_expression_edgeR(counts = geneCOUNT_fc, 
                                             meta = meta, cond_name = "strain", 
                                             sample_name = "srr.id", 
                                             gene_length_matrix = salmon_quant$avetxlength)
```

#### Diagnostics

(Back to top)

```
dfh <- data.frame(pvalue = c(res_fc_edgeR$tt$PValue,
                             res_fc_avetxl_edgeR$tt$PValue,
                             res_sal_scaledTPM_edgeR$tt$PValue,
                             res_sal_simplesum_edgeR$tt$PValue, 
                             res_sal_simplesum_avetxl_edgeR$tt$PValue),
                  mth = c(rep("featureCounts", nrow(res_fc_edgeR$tt)),
                          rep("featureCounts_avetxl", nrow(res_fc_avetxl_edgeR$tt)),
                          rep("scaledTPM_salmon", nrow(res_sal_scaledTPM_edgeR$tt)),
                          rep("simplesum_salmon", nrow(res_sal_simplesum_edgeR$tt)),
                          rep("simplesum_salmon_avetxl", nrow(res_sal_simplesum_avetxl_edgeR$tt))))
ggplot(dfh, aes(x = pvalue)) + geom_histogram() + facet_wrap(~mth) + 
  plot_theme() + 
  xlab("p-value") + ylab("count")
```

```
par(mfrow = c(2, 3))
plotBCV(res_fc_edgeR$dge, main = "featureCounts")
plotBCV(res_fc_avetxl_edgeR$dge, main = "featureCounts_avetxl")
plotBCV(res_sal_scaledTPM_edgeR$dge, main = "scaledTPM_salmon")
plotBCV(res_sal_simplesum_edgeR$dge, main = "simplesum_salmon")
plotBCV(res_sal_simplesum_avetxl_edgeR$dge, main = "simplesum_salmon_avetxl")
```

```
par(mfrow = c(2, 3))
plotSmear(res_fc_edgeR$dge, main = "featureCounts", ylim = c(-18, 18))
plotSmear(res_fc_avetxl_edgeR$dge, main = "featureCounts_avetxl", ylim = c(-18, 18))
plotSmear(res_sal_scaledTPM_edgeR$dge, main = "scaledTPM_salmon", ylim = c(-18, 18))
plotSmear(res_sal_simplesum_edgeR$dge, main = "simplesum_salmon", ylim = c(-18, 18))
plotSmear(res_sal_simplesum_avetxl_edgeR$dge, main = "simplesum_salmon_avetxl", ylim = c(-18, 18))
par(mfrow = c(1, 1))
```

### Comparison of significant genes found with different matrices

(Back to top)

```
cobra_edgeR <- COBRAData(padj = data.frame(simplesum_salmon = res_sal_simplesum_edgeR$tt$FDR, 
                                           row.names = rownames(res_sal_simplesum_edgeR$tt)))
cobra_edgeR <- COBRAData(padj = data.frame(scaledTPM_salmon = res_sal_scaledTPM_edgeR$tt$FDR, 
                                           row.names = rownames(res_sal_scaledTPM_edgeR$tt)),
                         object_to_extend = cobra_edgeR)
cobra_edgeR <- COBRAData(padj = data.frame(simplesum_salmon_avetxl = res_sal_simplesum_avetxl_edgeR$tt$FDR, 
                                           row.names = rownames(res_sal_simplesum_avetxl_edgeR$tt)),
                         object_to_extend = cobra_edgeR)
cobra_edgeR <- COBRAData(padj = data.frame(featureCounts = res_fc_edgeR$tt$FDR,
                                           row.names = rownames(res_fc_edgeR$tt)), 
                         object_to_extend = cobra_edgeR)
cobra_edgeR <- COBRAData(padj = data.frame(featureCounts_avetxl = res_fc_avetxl_edgeR$tt$FDR,
                                           row.names = rownames(res_fc_avetxl_edgeR$tt)), 
                         object_to_extend = cobra_edgeR)
cobraperf_edgeR <- calculate_performance(cobra_edgeR, aspects = "overlap", thr_venn = 0.05)
cobraplot1_edgeR <- prepare_data_for_plot(cobraperf_edgeR, incltruth = FALSE, 
                                          colorscheme = c("blue", "cyan", "black", "green", "red"))
plot_overlap(cobraplot1_edgeR, cex = c(1, 0.7, 0.7))
```

### Comparison of logFC estimates - all methods

(Back to top)

```
df1 <- Reduce(function(...) merge(..., by = "gene", all = TRUE), 
              list(data.frame(gene = rownames(res_sal_scaledTPM_edgeR$tt),
                              scaledTPM_salmon = res_sal_scaledTPM_edgeR$tt$logFC,
                              stringsAsFactors = FALSE),
                   data.frame(gene = rownames(res_sal_simplesum_edgeR$tt),
                              simplesum_salmon = res_sal_simplesum_edgeR$tt$logFC, 
                              stringsAsFactors = FALSE),
                   data.frame(gene = rownames(res_sal_simplesum_avetxl_edgeR$tt),
                              simplesum_salmon_avetxl =
                                res_sal_simplesum_avetxl_edgeR$tt$logFC,
                              stringsAsFactors = FALSE)))
rownames(df1) <- df1$gene
df1$gene <- NULL
pairs(df1, upper.panel = panel_smooth, lower.panel = panel_cor)
```

### Comparison of logFC estimates - simplesum vs scaledTPM

(Back to top)

```
df2 <- Reduce(function(...) merge(..., by = "gene", all = TRUE), 
              list(data.frame(gene = rownames(salmon_quant$geneTPM_sal),
                              salmon_quant$geneTPM_sal,
                              stringsAsFactors = FALSE),
                   data.frame(gene = rownames(res_sal_scaledTPM_edgeR$tt),
                              scaledTPM_salmon_logFC = res_sal_scaledTPM_edgeR$tt$logFC,
                              scaledTPM_salmon_logCPM = res_sal_scaledTPM_edgeR$tt$logCPM, 
                              stringsAsFactors = FALSE),
                   data.frame(gene = rownames(res_sal_simplesum_edgeR$tt),
                              simplesum_salmon_logFC = res_sal_simplesum_edgeR$tt$logFC, 
                              simplesum_salmon_logCPM = res_sal_simplesum_edgeR$tt$logCPM, 
                              stringsAsFactors = FALSE),
                   data.frame(gene = rownames(res_sal_simplesum_avetxl_edgeR$tt),
                              simplesum_salmon_avetxl_logFC =
                                res_sal_simplesum_avetxl_edgeR$tt$logFC,
                              simplesum_salmon_avetxl_logCPM = 
                                res_sal_simplesum_avetxl_edgeR$tt$logCPM, 
                              stringsAsFactors = FALSE)))
rownames(df2) <- df2$gene
df2$gene <- NULL
df2$scaledTPM_salmon_logCPMbinary <- Hmisc::cut2(df2$scaledTPM_salmon_logCPM, g = 2)
df2$simplesum_salmon_logCPMbinary <- Hmisc::cut2(df2$simplesum_salmon_logCPM, g = 2)
df2$simplesum_salmon_avetxl_logCPMbinary <- Hmisc::cut2(df2$simplesum_salmon_avetxl_logCPM, g = 2)
df2$sumA <- rowSums(df2[, meta$srr.id[meta$strain == "DBA/2J"]])
df2$sumB <- rowSums(df2[, meta$srr.id[meta$strain == "C57BL/6J"]])
df2$allzero_onecond <- "expressed in both groups"
df2$allzero_onecond[union(which(df2$sumA == 0), which(df2$sumB == 0))] <- "expressed in one group"
df2$onecol <- rep("", nrow(df2))

ggplot(df2, aes(x = simplesum_salmon_logFC, y = scaledTPM_salmon_logFC, col = onecol)) + 
  geom_abline(intercept = 0, slope = 1) + 
  geom_point(size = 2, alpha = 0.5) + 
  plot_theme() + ggtitle("Bottomly") + theme(legend.position = "bottom") + 
  scale_color_manual(values = c("blue"), name = "") + 
  theme(legend.background = element_rect(fill = "white"), legend.key = element_blank()) + 
  xlab("simplesum_salmon, logFC") + ylab("scaledTPM_salmon, logFC") + 
  guides(colour = guide_legend(override.aes = list(size = 0)))
```

```
ggplot(df2, aes(x = simplesum_salmon_logFC, y = scaledTPM_salmon_logFC, col = allzero_onecond)) + 
  geom_abline(intercept = 0, slope = 1) + 
  geom_point(size = 2, alpha = 0.5) + 
  plot_theme() + ggtitle("Bottomly") + theme(legend.position = "bottom") + 
  scale_color_manual(values = c("blue", "red"), name = "") + 
  xlab("simplesum_salmon, logFC") + ylab("scaledTPM_salmon, logFC") + 
  guides(colour = guide_legend(override.aes = list(size = 7)))
```

```
ggplot(subset(df2, !is.na(scaledTPM_salmon_logCPMbinary)), 
       aes(x = simplesum_salmon_logFC, y = scaledTPM_salmon_logFC, 
           col = scaledTPM_salmon_logCPMbinary)) + 
  geom_abline(intercept = 0, slope = 1) + 
  geom_point(size = 2, alpha = 0.5) + 
  facet_wrap(~scaledTPM_salmon_logCPMbinary) + 
  plot_theme() + ggtitle("Bottomly") + theme(legend.position = "bottom") + 
  xlab("simplesum_salmon, logFC") + ylab("scaledTPM_salmon, logFC") + 
  scale_color_manual(values = c("red", "blue"), name = "scaledTPM_salmon, logCPM") + 
  guides(colour = guide_legend(override.aes = list(size = 7)))
```

## DTU analysis on Salmon counts, with DEXSeq

(Back to top)

```
BPPARAM = MulticoreParam(6)
dxd <- DEXSeqDataSet(countData = round(salmon_quant$txCOUNT_sal), sampleData = meta, 
                     design = ~sample + exon + strain:exon,
                     featureID = rownames(salmon_quant$txCOUNT_sal),
                     groupID = tx2gene$gene[match(rownames(salmon_quant$txCOUNT_sal), 
                                                  tx2gene$transcript)])
dxd <- estimateSizeFactors(dxd)
dxd <- estimateDispersions(dxd, BPPARAM = BPPARAM)
```

```
## using supplied model matrix 
## using supplied model matrix 
## using supplied model matrix 
## using supplied model matrix 
## using supplied model matrix 
## using supplied model matrix 
## using supplied model matrix 
## using supplied model matrix 
## using supplied model matrix 
## using supplied model matrix 
## using supplied model matrix 
## using supplied model matrix
```

```
plotDispEsts(dxd)
```

```
dxd <- testForDEU(dxd, BPPARAM = BPPARAM)
```

```
## using supplied model matrix 
## using supplied model matrix 
## using supplied model matrix 
## using supplied model matrix 
## using supplied model matrix 
## using supplied model matrix
```

```
dxr <- DEXSeqResults(dxd)
qval_dtu_salmon <- perGeneQValue(dxr)
```

## Differential transcript expression (DTE) analysis on Salmon counts

(Back to top)

```
res_dte_salmon_tx <- diff_expression_edgeR(counts = salmon_quant$txCOUNT_sal, 
                                           meta = meta, cond_name = "strain", 
                                           sample_name = "srr.id", 
                                           gene_length_matrix = NULL) 
res_dte_salmon_tx$tt$gene <- as.character(tx2gene$gene[match(rownames(res_dte_salmon_tx$tt), 
                                                             tx2gene$transcript)])

## Summarise on gene level
geneSplit = split(seq(along = res_dte_salmon_tx$tt$gene), res_dte_salmon_tx$tt$gene)
pGene = sapply(geneSplit, function(i) min(res_dte_salmon_tx$tt$PValue[i]))
stopifnot(all(is.finite(pGene)))
theta = unique(sort(pGene))
q = DEXSeq:::perGeneQValueExact(pGene, theta, geneSplit)
qval_dte_salmon_gene = rep(NA_real_, length(pGene))
qval_dte_salmon_gene = q[match(pGene, theta)]
qval_dte_salmon_gene = pmin(1, qval_dte_salmon_gene)
names(qval_dte_salmon_gene) = names(geneSplit)
stopifnot(!any(is.na(qval_dte_salmon_gene)))
```

### Comparison of significant genes from DTE, DTU, DGE (scaled TPM)

(Back to top)

```
qval_dge <- res_sal_scaledTPM_edgeR$tt$FDR
names(qval_dge) <- rownames(res_sal_scaledTPM_edgeR$tt)

cobra <- COBRAData(padj = data.frame(DTE = qval_dte_salmon_gene, row.names = names(qval_dte_salmon_gene)))
cobra <- COBRAData(padj = data.frame(DTU = qval_dtu_salmon, row.names = names(qval_dtu_salmon)), 
                   object_to_extend = cobra)
cobra <- COBRAData(padj = data.frame(DGE = qval_dge, row.names = names(qval_dge)), 
                   object_to_extend = cobra)
cobraperf <- calculate_performance(cobra, aspects = "overlap", thr_venn = 0.05)
cobraplot <- prepare_data_for_plot(cobraperf)
plot_overlap(cobraplot)
```

## Differential expression analysis (DESeq2)

(Back to top)

```
res_sal_simplesum_deseq2 <- diff_expression_DESeq2(txi = NULL,
                                                   counts = salmon_quant$geneCOUNT_sal_simplesum, 
                                                   meta = meta, cond_name = "strain", 
                                                   level1 = "C57BL/6J", level2 = "DBA/2J", 
                                                   sample_name = "srr.id")
res_sal_simplesum_avetxl_deseq2 <- diff_expression_DESeq2(txi = salmon_quant$txi_salmonsimplesum,
                                                          counts = NULL, 
                                                          meta = meta, cond_name = "strain", 
                                                          level1 = "C57BL/6J", level2 = "DBA/2J", 
                                                          sample_name = "srr.id")
res_sal_scaledTPM_deseq2 <- diff_expression_DESeq2(txi = NULL,
                                                   counts = salmon_quant$geneCOUNT_sal_scaledTPM, 
                                                   meta = meta, cond_name = "strain", 
                                                   level1 = "C57BL/6J", level2 = "DBA/2J", 
                                                   sample_name = "srr.id")
res_fc_deseq2 <- diff_expression_DESeq2(txi = NULL,
                                        counts = geneCOUNT_fc, 
                                        meta = meta, cond_name = "strain", 
                                        level1 = "C57BL/6J", level2 = "DBA/2J", 
                                        sample_name = "srr.id")
res_fc_avetxl_deseq2 <- 
  diff_expression_DESeq2(txi = list(counts = geneCOUNT_fc, 
                                    length = salmon_quant$avetxlength[match(rownames(geneCOUNT_fc),
                                                                            rownames(salmon_quant$avetxlength)),
                                                                      match(colnames(geneCOUNT_fc),
                                                                            colnames(salmon_quant$avetxlength))],
                                    countsFromAbundance = "no"),
                         counts = NULL,
                         meta = meta, cond_name = "strain", 
                         level1 = "C57BL/6J", level2 = "DBA/2J", 
                         sample_name = "srr.id")
```

### Diagnostics

(Back to top)

```
dfh <- data.frame(pvalue = c(res_fc_deseq2$res$pvalue,
                             res_fc_avetxl_deseq2$res$pvalue,
                             res_sal_scaledTPM_deseq2$res$pvalue,
                             res_sal_simplesum_deseq2$res$pvalue, 
                             res_sal_simplesum_avetxl_deseq2$res$pvalue),
                  mth = c(rep("featureCounts", nrow(res_fc_deseq2$res)),
                          rep("featureCounts_avetxl", nrow(res_fc_avetxl_deseq2$res)),
                          rep("scaledTPM_salmon", nrow(res_sal_scaledTPM_deseq2$res)),
                          rep("simplesum_salmon", nrow(res_sal_simplesum_deseq2$res)),
                          rep("simplesum_salmon_avetxl", nrow(res_sal_simplesum_avetxl_deseq2$res))))
ggplot(dfh, aes(x = pvalue)) + geom_histogram() + facet_wrap(~mth) + 
  plot_theme() + 
  xlab("p-value") + ylab("count")
```

```
par(mfrow = c(2, 3))
plotDispEsts(res_fc_deseq2$dsd, main = "featureCounts")
plotDispEsts(res_fc_avetxl_deseq2$dsd, main = "featureCounts_avetxl")
plotDispEsts(res_sal_scaledTPM_deseq2$dsd, main = "scaledTPM_salmon")
plotDispEsts(res_sal_simplesum_deseq2$dsd, main = "simplesum_salmon")
plotDispEsts(res_sal_simplesum_avetxl_deseq2$dsd, main = "simplesum_salmon_avetxl")
```

```
par(mfrow = c(2, 3))
DESeq2::plotMA(res_fc_deseq2$dsd, main = "featureCounts")
DESeq2::plotMA(res_fc_avetxl_deseq2$dsd, main = "featureCounts_avetxl")
DESeq2::plotMA(res_sal_scaledTPM_deseq2$dsd, main = "scaledTPM_salmon")
DESeq2::plotMA(res_sal_simplesum_deseq2$dsd, main = "simplesum_salmon")
DESeq2::plotMA(res_sal_simplesum_avetxl_deseq2$dsd, main = "simplesum_salmon_avetxl")
par(mfrow = c(1, 1))
```

### Comparison of significant genes found with different matrices

(Back to top)

```
cobra_deseq2 <- COBRAData(padj = data.frame(simplesum_salmon = res_sal_simplesum_deseq2$res$padj, 
                                            row.names = rownames(res_sal_simplesum_deseq2$res)))
cobra_deseq2 <- COBRAData(padj = data.frame(scaledTPM_salmon = res_sal_scaledTPM_deseq2$res$padj, 
                                            row.names = rownames(res_sal_scaledTPM_deseq2$res)),
                          object_to_extend = cobra_deseq2)
cobra_deseq2 <- COBRAData(padj = data.frame(simplesum_salmon_avetxl = res_sal_simplesum_avetxl_deseq2$res$padj, 
                                            row.names = rownames(res_sal_simplesum_avetxl_deseq2$res)),
                          object_to_extend = cobra_deseq2)
cobra_deseq2 <- COBRAData(padj = data.frame(featureCounts = res_fc_deseq2$res$padj,
                                            row.names = rownames(res_fc_deseq2$res)), 
                          object_to_extend = cobra_deseq2)
cobra_deseq2 <- COBRAData(padj = data.frame(featureCounts_avetxl = res_fc_avetxl_deseq2$res$padj,
                                            row.names = rownames(res_fc_avetxl_deseq2$res)), 
                          object_to_extend = cobra_deseq2)
cobraperf_deseq2 <- calculate_performance(cobra_deseq2, aspects = "overlap", thr_venn = 0.05)
cobraplot1_deseq2 <- prepare_data_for_plot(cobraperf_deseq2, incltruth = FALSE, 
                                           colorscheme = c("blue", "cyan", "black", "green", "red"))
plot_overlap(cobraplot1_deseq2, cex = c(1, 0.7, 0.7))
```

### Comparison of logFC estimates - all methods

(Back to top)

```
df1 <- Reduce(function(...) merge(..., by = "gene", all = TRUE), 
              list(data.frame(gene = rownames(res_sal_scaledTPM_deseq2$res),
                              scaledTPM_salmon = res_sal_scaledTPM_deseq2$res$log2FoldChange,
                              stringsAsFactors = FALSE),
                   data.frame(gene = rownames(res_sal_simplesum_deseq2$res),
                              simplesum_salmon = res_sal_simplesum_deseq2$res$log2FoldChange, 
                              stringsAsFactors = FALSE),
                   data.frame(gene = rownames(res_sal_simplesum_avetxl_deseq2$res),
                              simplesum_salmon_avetxl =
                                res_sal_simplesum_avetxl_deseq2$res$log2FoldChange,
                              stringsAsFactors = FALSE)))
rownames(df1) <- df1$gene
df1$gene <- NULL
pairs(df1, upper.panel = panel_smooth, lower.panel = panel_cor)
```

### Comparison of logFC estimates - simplesum vs scaledTPM

(Back to top)

```
df2 <- Reduce(function(...) merge(..., by = "gene", all = TRUE), 
              list(data.frame(gene = rownames(salmon_quant$geneTPM_sal),
                              salmon_quant$geneTPM_sal,
                              stringsAsFactors = FALSE),
                   data.frame(gene = rownames(res_sal_scaledTPM_deseq2$res),
                              scaledTPM_salmon_logFC = res_sal_scaledTPM_deseq2$res$log2FoldChange,
                              scaledTPM_salmon_basemean = res_sal_scaledTPM_deseq2$res$baseMean, 
                              stringsAsFactors = FALSE),
                   data.frame(gene = rownames(res_sal_simplesum_deseq2$res),
                              simplesum_salmon_logFC = res_sal_simplesum_deseq2$res$log2FoldChange, 
                              simplesum_salmon_basemean = res_sal_simplesum_deseq2$res$baseMean, 
                              stringsAsFactors = FALSE),
                   data.frame(gene = rownames(res_sal_simplesum_avetxl_deseq2$res),
                              simplesum_salmon_avetxl_logFC =
                                res_sal_simplesum_avetxl_deseq2$res$log2FoldChange,
                              simplesum_salmon_avetxl_basemean = 
                                res_sal_simplesum_avetxl_deseq2$res$baseMean, 
                              stringsAsFactors = FALSE)))
rownames(df2) <- df2$gene
df2$gene <- NULL
df2$scaledTPM_salmon_basemeanbinary <- Hmisc::cut2(df2$scaledTPM_salmon_basemean, g = 2)
df2$simplesum_salmon_basemeanbinary <- Hmisc::cut2(df2$simplesum_salmon_basemean, g = 2)
df2$simplesum_salmon_avetxl_basemeanbinary <- Hmisc::cut2(df2$simplesum_salmon_avetxl_basemean, g = 2)
df2$sumA <- rowSums(df2[, meta$srr.id[meta$strain == "DBA/2J"]])
df2$sumB <- rowSums(df2[, meta$srr.id[meta$strain == "C57BL/6J"]])
df2$allzero_onecond <- "expressed in both groups"
df2$allzero_onecond[union(which(df2$sumA == 0), which(df2$sumB == 0))] <- "expressed in one group"
df2$onecol <- rep("", nrow(df2))

ggplot(df2, aes(x = simplesum_salmon_logFC, y = scaledTPM_salmon_logFC, col = onecol)) + 
  geom_abline(intercept = 0, slope = 1) + 
  geom_point(size = 2, alpha = 0.5) + 
  plot_theme() + ggtitle("Bottomly") + theme(legend.position = "bottom") + 
  scale_color_manual(values = c("blue"), name = "") + 
  theme(legend.background = element_rect(fill = "white"), legend.key = element_blank()) + 
  xlab("simplesum_salmon, logFC") + ylab("scaledTPM_salmon, logFC") + 
  guides(colour = guide_legend(override.aes = list(size = 0)))
```

```
ggplot(df2, aes(x = simplesum_salmon_logFC, y = scaledTPM_salmon_logFC, col = allzero_onecond)) + 
  geom_abline(intercept = 0, slope = 1) + 
  geom_point(size = 2, alpha = 0.5) + 
  plot_theme() + ggtitle("Bottomly") + theme(legend.position = "bottom") + 
  scale_color_manual(values = c("blue", "red"), name = "") + 
  xlab("simplesum_salmon, logFC") + ylab("scaledTPM_salmon, logFC") + 
  guides(colour = guide_legend(override.aes = list(size = 7)))
```

```
ggplot(subset(df2, !is.na(scaledTPM_salmon_basemeanbinary)), 
       aes(x = simplesum_salmon_logFC, y = scaledTPM_salmon_logFC, 
           col = scaledTPM_salmon_basemeanbinary)) + 
  geom_abline(intercept = 0, slope = 1) + 
  geom_point(size = 2, alpha = 0.5) + 
  facet_wrap(~scaledTPM_salmon_basemeanbinary) + 
  plot_theme() + ggtitle("Bottomly") + theme(legend.position = "bottom") + 
  xlab("simplesum_salmon, logFC") + ylab("scaledTPM_salmon, logFC") + 
  scale_color_manual(values = c("red", "blue"), name = "scaledTPM_salmon, base mean") + 
  guides(colour = guide_legend(override.aes = list(size = 7)))
```

## Help functions

(Back to top)

```
panel_cor <- function(x, y, digits = 3, cex.cor) {
  ## Panel function to print Pearson and Spearman correlations
  usr <- par("usr")
  on.exit(par(usr))
  par(usr = c(0, 1, 0, 1))
  r1 <- abs(cor(x, y, method = "pearson", use = "complete"))
  txt1 <- format(c(r1, 0.123456789), digits = digits)[1]
  r2 <- abs(cor(x, y, method = "spearman", use = "complete"))
  txt2 <- format(c(r2, 0.123456789), digits = digits)[1]
  text(0.5, 0.35, paste("pearson =", txt1), cex = 1.1)
  text(0.5, 0.65, paste("spearman =", txt2), cex = 1.1)
}
```

```
panel_smooth<-function (x, y, col = "blue", bg = NA, pch = ".", 
                        cex = 0.8, ...) {
  ## Panel function to plot points
  points(x, y, pch = pch, col = col, bg = bg, cex = cex)
}
```

```
plot_theme <- function() {
  ## ggplot2 plotting theme
  theme_grey() +
    theme(legend.position = "right",
          panel.background = element_rect(fill = "white", colour = "black"),
          panel.grid.minor.x = element_blank(),
          panel.grid.minor.y = element_blank(),
          strip.text = element_text(size = 10),
          strip.background = element_rect(fill = NA, colour = "black"),
          axis.text.x = element_text(size = 10),
          axis.text.y = element_text(size = 10),
          axis.title.x = element_text(size = 15),
          axis.title.y = element_text(size = 15),
          plot.title = element_text(colour = "black", size = 20))
}
```

```
calc_lengths_mapping <- function(gtf, cdna_fasta, feature_lengths_file,
                                 tx_gene_file) {
  ## Function to calculate gene and transcript lengths from transcript cDNA 
  ## fasta and gtf file. Also generate mapping between transcript and gene IDs.
  
  suppressPackageStartupMessages(library(GenomicFeatures))
  suppressPackageStartupMessages(library(Biostrings))
  
  ## Gene/transcript lengths ===============================================
  ## Transcripts/genes present in gtf file
  if (!is.null(gtf)) {
    txdb <- makeTxDbFromGFF(gtf, format = "gtf")
    ebg <- exonsBy(txdb, "gene")
    ebt <- exonsBy(txdb, "tx", use.names = TRUE)
    ebg_red <- reduce(ebg)
    gene_length <- sum(width(ebg_red))
    ebt_red <- reduce(ebt)
    tx_length <- sum(width(ebt_red))
  } else {
    tx_length <- c()
    gene_length <- c()
  }  
  
  ## Extend with transcripts from cDNA fasta
  cdna <- readDNAStringSet(gsub("\\.gz", "", cdna_fasta))
  tx_length2 <- width(cdna)
  names(tx_length2) <- sapply(names(cdna), function(i) strsplit(i, " ")[[1]][1])
  tx_length2 <- tx_length2[setdiff(names(tx_length2), names(tx_length))]
  tx_length <- c(tx_length, tx_length2)
  
  ## Gene/transcript mapping ===============================================
  ## Transcripts/genes present in gtf file
  if (!is.null(gtf)) {
    tbg <- transcriptsBy(txdb, "gene")
    tx2gene <- stack(lapply(tbg, function(w) w$tx_name))
    colnames(tx2gene) <- c("transcript", "gene")
  } else {
    tx2gene <- data.frame(transcript = c(), gene = c())
  }  
  
  ## Extend with mappings from cDNA fasta file
  tx <- sapply(names(cdna), function(i) strsplit(i, " ")[[1]][1])
  gn <- sapply(names(cdna), function(i) gsub("gene:", "", strsplit(i, " ")[[1]][4]))
  tx2gene2 <- data.frame(transcript = tx, gene = gn, 
                         stringsAsFactors = FALSE)
  rownames(tx2gene2) <- NULL
  tx2gene2 <- tx2gene2[match(setdiff(tx2gene2$transcript, 
                                     tx2gene$transcript), tx2gene2$transcript), ]
  tx2gene <- rbind(tx2gene, tx2gene2)
  
  gene2tx <- tx2gene[, c("gene", "transcript")]
  
  save(gene_length, tx_length, file = feature_lengths_file)
  save(gene2tx, tx2gene, file = tx_gene_file)
}
```

```
diff_expression_edgeR <- function(counts, meta, cond_name, sample_name, 
                                  gene_length_matrix = NULL) {
  ## Differential expression analysis with edgeR
  
  suppressPackageStartupMessages(library(edgeR))

  ## Prepare DGEList
  counts <- round(counts)
  cts <- counts[rowSums(is.na(counts)) == 0, ]
  cts <- cts[rowSums(cts) != 0, ]
  dge <- 
    DGEList(counts = cts, group = meta[, cond_name][match(colnames(cts), 
                                                          meta[, sample_name])])
  
  ## If average transcript lengths provided, add as offset
  if (!is.null(gene_length_matrix)) {
    egf <- gene_length_matrix[match(rownames(cts), rownames(gene_length_matrix)),
                              match(colnames(cts), colnames(gene_length_matrix))]
    egf <- egf / exp(rowMeans(log(egf)))
    o <- log(calcNormFactors(cts/egf)) + log(colSums(cts/egf))
    dge$offset <- t(t(log(egf)) + o)
  } else {
    dge <- calcNormFactors(dge)
  }
  
  ## Estimate dispersions and fit model
  design <- model.matrix(~dge$samples$group)
  dge <- estimateGLMCommonDisp(dge, design = design)
  dge <- estimateGLMTrendedDisp(dge, design = design)
  dge <- estimateGLMTagwiseDisp(dge, design = design)
  fit <- glmFit(dge, design = design)
  lrt <- glmLRT(fit)
  tt <- topTags(lrt, n = Inf)$table
  return(list(dge = dge, tt = tt))
}
```

```
diff_expression_DESeq2 <- function(txi = NULL, counts, meta, cond_name, 
                                   level1, level2, sample_name) {
  ## Differential expression analysis with DESeq2
  
  suppressPackageStartupMessages(library(DESeq2, 
                                         lib.loc = "/home/charlotte/R/x86_64-pc-linux-gnu-library/3.2"))

  ## If tximport object provided, generate DESeqDataSet from it. Otherwise, 
  ## use the provided count matrix.
  if (!is.null(txi)) {
    txi$counts <- round(txi$counts)
    keep_feat <- rownames(txi$counts[rowSums(is.na(txi$counts)) == 0 & rowSums(txi$counts) != 0, ])
    txi <- lapply(txi, function(w) {
      if (!is.null(dim(w))) w[match(keep_feat, rownames(w)), ]
      else w
      })
    dsd <- DESeqDataSetFromTximport(txi, 
                                    colData = meta[match(colnames(txi$counts), 
                                                         meta[, sample_name]), ],
                                    design = as.formula(paste0("~", cond_name)))
  } else {
    counts <- round(counts)
    cts = counts[rowSums(is.na(counts)) == 0, ]
    cts <- cts[rowSums(cts) != 0, ]
    dsd <- DESeqDataSetFromMatrix(countData = round(cts), 
                                  colData = meta[match(colnames(cts), 
                                                       meta[, sample_name]), ],
                                  design = as.formula(paste0("~", cond_name)))
  }
  
  ## Estimate dispersions and fit model
  dsd <- DESeq(dsd, test = "Wald", fitType = "local", betaPrior = TRUE)
  res <- as.data.frame(results(dsd, contrast = c(cond_name, level2, level1),
                               cooksCutoff = FALSE, independentFiltering = FALSE))
  return(list(dsd = dsd, res = res))
}
```

## Session info

(Back to top)

```
sessionInfo()
```

```
## R version 3.2.2 (2015-08-14)
## Platform: x86_64-pc-linux-gnu (64-bit)
## Running under: Ubuntu 14.04.3 LTS
## 
## locale:
##  [1] LC_CTYPE=C                 LC_NUMERIC=C               LC_TIME=en_CA.UTF-8       
##  [4] LC_COLLATE=en_CA.UTF-8     LC_MONETARY=en_CA.UTF-8    LC_MESSAGES=en_CA.UTF-8   
##  [7] LC_PAPER=en_CA.UTF-8       LC_NAME=C                  LC_ADDRESS=C              
## [10] LC_TELEPHONE=C             LC_MEASUREMENT=en_CA.UTF-8 LC_IDENTIFICATION=C       
## 
## attached base packages:
## [1] parallel  stats4    stats     graphics  grDevices utils     datasets  methods   base     
## 
## other attached packages:
##  [1] edgeR_3.12.0               limma_3.26.3               DEXSeq_1.16.2             
##  [4] DESeq2_1.11.6              RcppArmadillo_0.6.200.2.0  Rcpp_0.12.2               
##  [7] SummarizedExperiment_1.0.1 Biobase_2.30.0             GenomicRanges_1.22.1      
## [10] GenomeInfoDb_1.6.1         IRanges_2.4.4              S4Vectors_0.8.3           
## [13] BiocGenerics_0.16.1        BiocParallel_1.4.0         dplyr_0.4.3               
## [16] ggplot2_1.0.1              iCOBRA_0.99.3              tximport_0.0.7            
## [19] Rsubread_1.20.2           
## 
## loaded via a namespace (and not attached):
##  [1] bitops_1.0-6         RColorBrewer_1.1-2   tools_3.2.2          R6_2.1.1            
##  [5] DT_0.1               rpart_4.1-10         KernSmooth_2.23-15   Hmisc_3.17-0        
##  [9] DBI_0.3.1            colorspace_1.2-6     nnet_7.3-10          gridExtra_2.0.0     
## [13] formatR_1.2.1        labeling_0.3         caTools_1.17.1       scales_0.3.0        
## [17] genefilter_1.52.0    stringr_1.0.0        digest_0.6.8         Rsamtools_1.22.0    
## [21] shinyBS_0.61         foreign_0.8-65       rmarkdown_0.8.1      XVector_0.10.0      
## [25] htmltools_0.2.6      htmlwidgets_0.5      RSQLite_1.0.0        shiny_0.12.2        
## [29] hwriter_1.3.2        gtools_3.5.0         acepack_1.3-3.3      RCurl_1.95-4.7      
## [33] magrittr_1.5         Formula_1.2-1        futile.logger_1.4.1  munsell_0.4.2       
## [37] proto_0.3-10         stringi_1.0-1        yaml_2.1.13          MASS_7.3-43         
## [41] zlibbioc_1.16.0      gplots_2.17.0        plyr_1.8.3           grid_3.2.2          
## [45] gdata_2.17.0         shinydashboard_0.5.1 lattice_0.20-33      Biostrings_2.38.2   
## [49] splines_3.2.2        annotate_1.48.0      locfit_1.5-9.1       knitr_1.11          
## [53] geneplotter_1.48.0   reshape2_1.4.1       biomaRt_2.26.0       futile.options_1.0.0
## [57] XML_3.98-1.3         evaluate_0.8         latticeExtra_0.6-26  lambda.r_1.1.7      
## [61] httpuv_1.3.3         gtable_0.1.2         assertthat_0.1       mime_0.4            
## [65] xtable_1.8-0         survival_2.38-3      AnnotationDbi_1.32.0 cluster_2.0.3       
## [69] statmod_1.4.22       ROCR_1.0-7
```
