# Supplementary material for: Differential analyses for RNA-seq: transcript-level estimates improve gene-level inferences
Source: F1000Res. 2016 Feb 29;4:1521. Originally published 2015 Dec 30. [Version 2] doi: 10.12688/f1000research.7563.2 (PMC4712774; doi:10.12688/f1000research.7563.2)
Supplement: Data set 6 — http://dx.doi.org/10.5256/f1000research.7563.d114730 Data set 6 (html) contain all the R code that was used to perform the analyses and generate the figures for the GSE72165 data set 35. [file f1000research-4-8770-s0005.tgz › b0434f4d-253a-4998-8f44-ae109a76678c_gse72165_quantification.html]

GSE72165


# GSE72165

- Preparation
  - Reference directories and packages
  - Metadata definition
  - FASTQ file download
  - Reference file preparation and index building
  - Definition of gene-to-transcript mapping
- Salmon abundance quantification
  - Summarization of Salmon results and offset estimation
- Differential expression analysis
  - edgeR
    - Diagnostics
    - Comparison of significant genes found with different matrices
    - Comparison of logFC estimates - all methods
    - Comparison of logFC estimates - simplesum vs scaledTPM
  - DESeq2
    - Diagnostics
    - Comparison of significant genes found with different matrices
    - Comparison of logFC estimates - all methods
    - Comparison of logFC estimates - simplesum vs scaledTPM
- DTU analysis on Salmon counts, with DEXSeq
- Help functions
- Session info

This report outlines the analysis of the GSE72165 data set, consisting of 3 samples from the carotid body and 3 samples from the adrenal medulla in mice.

## Preparation

(Back to top)

### Reference directories and packages

(Back to top)

```
basedir <- "/home/charlotte/gene_vs_tx_quantification"
refdir <- "/home/Shared/data/annotation"
suppressPackageStartupMessages(library(ggplot2))
suppressPackageStartupMessages(library(tximport))
suppressPackageStartupMessages(library(iCOBRA))
suppressPackageStartupMessages(library(dplyr))
suppressPackageStartupMessages(library(BiocParallel))
suppressPackageStartupMessages(library(DESeq2, 
                                         lib.loc = "/home/charlotte/R/x86_64-pc-linux-gnu-library/3.2"))
suppressPackageStartupMessages(library(DEXSeq))
```

### Metadata definition

(Back to top)

A metadata table was generated using the information provided in Gene Expression Omnibus.

```
meta <- read.delim(paste0(basedir, "/data/gse72165/gse72165_phenodata.txt"), 
                   header = TRUE, as.is = TRUE)
rownames(meta) <- meta$srr.id
meta
```

```
##                gsm.id     srr.id condition
## SRR2172551 GSM1856457 SRR2172551   carotid
## SRR2172552 GSM1856458 SRR2172552   carotid
## SRR2172553 GSM1856459 SRR2172553   carotid
## SRR2172554 GSM1856460 SRR2172554   medulla
## SRR2172555 GSM1856461 SRR2172555   medulla
## SRR2172556 GSM1856462 SRR2172556   medulla
```

### FASTQ file download

(Back to top)

The code below downloads the FASTQ files for the six samples from the SRA.

```
fastq_files <- c(paste0("ftp://ftp.sra.ebi.ac.uk/vol1/fastq/SRR217/00", 
                        c(1:6), "/", 
                        meta$srr.id, "/", meta$srr.id, ".fastq.gz"))
for (fq in fastq_files) {
  if (!file.exists(paste0(basedir, "/data/gse72165/fastq/", basename(fq)))) {
    cmd <- paste0("wget -P ", basedir, "/data/gse72165/fastq ", fq)
    message(cmd)
    system(cmd)
  } else {
    message(paste0(fq, " is already downloaded."))
  }
}
```

```
## ftp://ftp.sra.ebi.ac.uk/vol1/fastq/SRR217/001/SRR2172551/SRR2172551.fastq.gz is already downloaded.
```

```
## ftp://ftp.sra.ebi.ac.uk/vol1/fastq/SRR217/002/SRR2172552/SRR2172552.fastq.gz is already downloaded.
```

```
## ftp://ftp.sra.ebi.ac.uk/vol1/fastq/SRR217/003/SRR2172553/SRR2172553.fastq.gz is already downloaded.
```

```
## ftp://ftp.sra.ebi.ac.uk/vol1/fastq/SRR217/004/SRR2172554/SRR2172554.fastq.gz is already downloaded.
```

```
## ftp://ftp.sra.ebi.ac.uk/vol1/fastq/SRR217/005/SRR2172555/SRR2172555.fastq.gz is already downloaded.
```

```
## ftp://ftp.sra.ebi.ac.uk/vol1/fastq/SRR217/006/SRR2172556/SRR2172556.fastq.gz is already downloaded.
```

### Reference file preparation and index building

(Back to top)

We use the `GRCm38.82` Mus musculus Ensembl annotation for the analysis. Here, we download the fasta file with the cDNA sequences and build an index for quantification with Salmon.

```
cdna_fasta <- paste0(refdir, "/Mouse/Ensembl_GRCm38.82/cDNA/Mus_musculus.GRCm38.cdna.all.fa.gz")
salmon_index <- paste0(basedir, "/annotation/Mouse_Ensembl_GRCm38.82/salmon_index/Mus_musculus.GRCm38.82.gtf.sidx")

############################### cDNA FASTA ################################
if (!file.exists(cdna_fasta)) {
  cmd <- paste0("wget -P ", refdir, "/Mouse/Ensembl_GRCm38.82/cDNA ", 
                "ftp://ftp.ensembl.org/pub/release-82/fasta/mus_musculus/", 
                "cdna/Mus_musculus.GRCm38.cdna.all.fa.gz")
  message(cmd)
  system(cmd)
  cmd <- paste0("gunzip -c ", cdna_fasta, "> ", gsub("\\.gz$", "", cdna_fasta))
  message(cmd)
  system(cmd)
} else {
  message(paste0("Reference cDNA fasta is already downloaded."))
}
```

```
## Reference cDNA fasta is already downloaded.
```

```
## Build Salmon index
cmd <- paste("salmon index -i", salmon_index, "-t", gsub("\\.gz$", "", cdna_fasta), "-p 5 --type quasi")
message(cmd)
```

```
## salmon index -i /home/charlotte/gene_vs_tx_quantification/annotation/Mouse_Ensembl_GRCm38.82/salmon_index/Mus_musculus.GRCm38.82.gtf.sidx -t /home/Shared/data/annotation/Mouse/Ensembl_GRCm38.82/cDNA/Mus_musculus.GRCm38.cdna.all.fa -p 5 --type quasi
```

```
if (!file.exists(paste0(salmon_index, "/hash.bin"))) {
  system(cmd)
} else {
  message("Salmon index already exists.")
}
```

```
## Salmon index already exists.
```

### Definition of gene-to-transcript mapping

(Back to top)

Next, we derive a mapping between transcript and gene identifiers from the cDNA file downloaded above.

```
feature_lengths_file <- paste0(basedir, "/annotation/Mouse_Ensembl_GRCm38.82/feature_lengths.Rdata")
tx_gene_file <- paste0(basedir, "/annotation/Mouse_Ensembl_GRCm38.82/tx_gene_map.Rdata")
```

```
## Calculate gene and transcript lengths, get gene-transcript mapping
if (!file.exists(feature_lengths_file)) {
  calc_lengths_mapping(gtf = NULL, cdna_fasta = cdna_fasta, 
                       feature_lengths_file = feature_lengths_file, 
                       tx_gene_file = tx_gene_file) 
} else {
  message("feature lengths and tx-to-gene map already calculated.")
}
```

```
## feature lengths and tx-to-gene map already calculated.
```

```
load(tx_gene_file)
```

## Salmon abundance quantification

(Back to top)

Next, we use Salmon to calculate transcript abundance estimates for each of the six samples.

```
salmon_basedir <- paste0(basedir, "/quantifications/gse72165/salmon")

fqs <- list.files(paste0(basedir, "/data/gse72165/fastq"), full.names = TRUE)
names(fqs) <- gsub("\\.fastq.gz", "", basename(fqs))

for (i in 1:length(fqs)) {
  if (!file.exists(paste0(salmon_basedir, "/", names(fqs)[i], "/quant.sf"))) {
    cmd <- sprintf("bash -c 'salmon quant -i %s -l U -r %s -p 5 -o %s'",
                   salmon_index,
                   paste0("<(gunzip -c ", fqs[i], ")"),
                   paste0(salmon_basedir, "/", names(fqs)[i]))
    cat(cmd, "\n")
    system(cmd)
  } else {
    cat("Salmon results for", names(fqs)[i], "already exist.\n")
  }
}
```

```
## Salmon results for SRR2172551 already exist.
## Salmon results for SRR2172552 already exist.
## Salmon results for SRR2172553 already exist.
## Salmon results for SRR2172554 already exist.
## Salmon results for SRR2172555 already exist.
## Salmon results for SRR2172556 already exist.
```

### Summarization of Salmon results and offset estimation

(Back to top)

We use the `tximport` package (https://github.com/mikelove/tximport) to generate count matrices and offset matrices (average transcript lengths) from the Salmon transcript-level estimates. We generate two different count matrices (**simplesum** and **scaledTPM**), and additionally create offsets to be used with the **simplesum** matrix.

```
salmon_files <- list.files(salmon_basedir, pattern = "SRR", full.names = TRUE)
salmon_files <- salmon_files[file.info(salmon_files)$isdir]
salmon_files <- paste0(salmon_files, "/quant.sf")
salmon_files <- salmon_files[file.exists(salmon_files)]
names(salmon_files) <- basename(gsub("/quant.sf", "", salmon_files))
txi_salmonsimplesum <- tximport(files = salmon_files, type = "salmon", txIn = TRUE,
                                txOut = FALSE, countsFromAbundance = "no", 
                                gene2tx = gene2tx)
```

```
## reading in files
```

```
## 1
```

```
## 2
```

```
## 3
```

```
## 4
```

```
## 5
```

```
## 6
```

```
##
```

```
## summarizing abundance
```

```
## summarizing counts
```

```
## summarizing length
```

```
txi_salmonscaledtpm <- tximport(files = salmon_files, type = "salmon", txIn = TRUE,
                                txOut = FALSE, countsFromAbundance = "scaledTPM", 
                                gene2tx = gene2tx)
```

```
## reading in files
```

```
## 1
```

```
## 2
```

```
## 3
```

```
## 4
```

```
## 5
```

```
## 6
```

```
##
```

```
## summarizing abundance
```

```
## summarizing counts
```

```
## summarizing length
```

```
txi_salmontx <- tximport(files = salmon_files, type = "salmon", txIn = TRUE,
                         txOut = TRUE, countsFromAbundance = "no", gene2tx = gene2tx)
```

```
## reading in files
```

```
## 1
```

```
## 2
```

```
## 3
```

```
## 4
```

```
## 5
```

```
## 6
```

```
##
```

```
salmon_quant <- list(geneCOUNT_sal_simplesum = txi_salmonsimplesum$counts,
                     geneCOUNT_sal_scaledTPM = txi_salmonscaledtpm$counts,
                     avetxlength = txi_salmonsimplesum$length,
                     geneTPM_sal = txi_salmonsimplesum$abundance,
                     txTPM_sal = txi_salmontx$abundance,
                     txCOUNT_sal = txi_salmontx$counts,
                     txi_salmonsimplesum = txi_salmonsimplesum,
                     txi_salmonscaledtpm = txi_salmonscaledtpm,
                     txi_salmontx = txi_salmontx)
```

## Differential expression analysis

(Back to top)

Given the gene count matrices defined above we apply *edgeR* and *DESeq2* to perform differential gene expression. For the **simplesum** matrix, we also apply *edgeR* and *DESeq2* using the offsets derived from the average transcript lengths (**simplesum\_avetxl**).

### edgeR

(Back to top)

```
res_sal_simplesum_edgeR <- diff_expression_edgeR(counts = salmon_quant$geneCOUNT_sal_simplesum, 
                                                 meta = meta, cond_name = "condition", 
                                                 sample_name = "srr.id", 
                                                 gene_length_matrix = NULL)
res_sal_simplesum_avetxl_edgeR <- diff_expression_edgeR(counts = salmon_quant$geneCOUNT_sal_simplesum, 
                                                        meta = meta, cond_name = "condition", 
                                                        sample_name = "srr.id", 
                                                        gene_length_matrix = salmon_quant$avetxlength)
res_sal_scaledTPM_edgeR <- diff_expression_edgeR(counts = salmon_quant$geneCOUNT_sal_scaledTPM, 
                                                 meta = meta, cond_name = "condition", 
                                                 sample_name = "srr.id", 
                                                 gene_length_matrix = NULL)
```

#### Diagnostics

(Back to top)

```
dfh <- data.frame(pvalue = c(res_sal_scaledTPM_edgeR$tt$PValue,
                             res_sal_simplesum_avetxl_edgeR$tt$PValue,
                             res_sal_simplesum_edgeR$tt$PValue),
                  mth = c(rep("scaledTPM_salmon, edgeR", nrow(res_sal_scaledTPM_edgeR$tt)),
                          rep("simplesum_salmon_avetxl, edgeR", nrow(res_sal_simplesum_avetxl_edgeR$tt)),
                          rep("simplesum_salmon, edgeR", nrow(res_sal_simplesum_edgeR$tt))))
ggplot(dfh, aes(x = pvalue)) + geom_histogram() + facet_wrap(~mth) + 
  plot_theme() + 
  xlab("p-value") + ylab("count")
```

```
par(mfrow = c(1, 3))
plotBCV(res_sal_scaledTPM_edgeR$dge, main = "scaledTPM_salmon, edgeR")
plotBCV(res_sal_simplesum_avetxl_edgeR$dge, main = "simplesum_salmon_avetxl, edgeR")
plotBCV(res_sal_simplesum_edgeR$dge, main = "simplesum_salmon, edgeR")
```

```
par(mfrow = c(1, 3))
plotSmear(res_sal_scaledTPM_edgeR$dge, main = "scaledTPM_salmon, edgeR", ylim = c(-13, 13))
plotSmear(res_sal_simplesum_avetxl_edgeR$dge, main = "simplesum_salmon_avetxl, edgeR", ylim = c(-13, 13))
plotSmear(res_sal_simplesum_edgeR$dge, main = "simplesum_salmon, edgeR", ylim = c(-13, 13))
```

```
par(mfrow = c(1, 1))
```

#### Comparison of significant genes found with different matrices

(Back to top)

```
cobra_edgeR <- COBRAData(padj = data.frame(simplesum_salmon = res_sal_simplesum_edgeR$tt$FDR, 
                                           row.names = rownames(res_sal_simplesum_edgeR$tt)))
cobra_edgeR <- COBRAData(padj = data.frame(scaledTPM_salmon = res_sal_scaledTPM_edgeR$tt$FDR, 
                                           row.names = rownames(res_sal_scaledTPM_edgeR$tt)),
                         object_to_extend = cobra_edgeR)
cobra_edgeR <- COBRAData(padj = data.frame(simplesum_salmon_avetxl = res_sal_simplesum_avetxl_edgeR$tt$FDR, 
                                           row.names = rownames(res_sal_simplesum_avetxl_edgeR$tt)),
                         object_to_extend = cobra_edgeR)
cobraperf_edgeR <- calculate_performance(cobra_edgeR, aspects = "overlap", thr_venn = 0.05)

cobraplot1_edgeR <- prepare_data_for_plot(cobraperf_edgeR, incltruth = FALSE, 
                                          colorscheme = c("blue", "red", "black"))
plot_overlap(cobraplot1_edgeR, cex = c(1, 0.7, 0.7))
title("GSE72165, edgeR", line = 0)
```

#### Comparison of logFC estimates - all methods

(Back to top)

```
df1 <- Reduce(function(...) merge(..., by = "gene", all = TRUE), 
              list(data.frame(gene = rownames(res_sal_scaledTPM_edgeR$tt),
                              scaledTPM_salmon = res_sal_scaledTPM_edgeR$tt$logFC,
                              stringsAsFactors = FALSE),
                   data.frame(gene = rownames(res_sal_simplesum_edgeR$tt),
                              simplesum_salmon = res_sal_simplesum_edgeR$tt$logFC, 
                              stringsAsFactors = FALSE),
                   data.frame(gene = rownames(res_sal_simplesum_avetxl_edgeR$tt),
                              simplesum_salmon_avetxl =
                                res_sal_simplesum_avetxl_edgeR$tt$logFC,
                              stringsAsFactors = FALSE)))
rownames(df1) <- df1$gene
df1$gene <- NULL
pairs(df1, upper.panel = panel_smooth, lower.panel = panel_cor)
```

#### Comparison of logFC estimates - simplesum vs scaledTPM

(Back to top)

```
df2 <- Reduce(function(...) merge(..., by = "gene", all = TRUE), 
              list(data.frame(gene = rownames(salmon_quant$geneTPM_sal),
                              salmon_quant$geneTPM_sal,
                              stringsAsFactors = FALSE),
                   data.frame(gene = rownames(res_sal_scaledTPM_edgeR$tt),
                              scaledTPM_salmon_logFC = res_sal_scaledTPM_edgeR$tt$logFC,
                              scaledTPM_salmon_logCPM = res_sal_scaledTPM_edgeR$tt$logCPM, 
                              stringsAsFactors = FALSE),
                   data.frame(gene = rownames(res_sal_simplesum_edgeR$tt),
                              simplesum_salmon_logFC = res_sal_simplesum_edgeR$tt$logFC, 
                              simplesum_salmon_logCPM = res_sal_simplesum_edgeR$tt$logCPM, 
                              stringsAsFactors = FALSE),
                   data.frame(gene = rownames(res_sal_simplesum_avetxl_edgeR$tt),
                              simplesum_salmon_avetxl_logFC =
                                res_sal_simplesum_avetxl_edgeR$tt$logFC,
                              simplesum_salmon_avetxl_logCPM = 
                                res_sal_simplesum_avetxl_edgeR$tt$logCPM, 
                              stringsAsFactors = FALSE)))
rownames(df2) <- df2$gene
df2$gene <- NULL
df2$scaledTPM_salmon_logCPMbinary <- Hmisc::cut2(df2$scaledTPM_salmon_logCPM, g = 2)
df2$simplesum_salmon_logCPMbinary <- Hmisc::cut2(df2$simplesum_salmon_logCPM, g = 2)
df2$simplesum_salmon_avetxl_logCPMbinary <- Hmisc::cut2(df2$simplesum_salmon_avetxl_logCPM, g = 2)
df2$sumA <- rowSums(df2[, meta$srr.id[meta$condition == "carotid"]])
df2$sumB <- rowSums(df2[, meta$srr.id[meta$condition == "medulla"]])
df2$allzero_onecond <- "expressed in both groups"
df2$allzero_onecond[union(which(df2$sumA == 0), which(df2$sumB == 0))] <- "expressed in one group"
df2$onecol <- rep("", nrow(df2))

ggplot(df2, aes(x = simplesum_salmon_logFC, y = scaledTPM_salmon_logFC, col = onecol)) + 
  geom_abline(intercept = 0, slope = 1) + 
  geom_point(size = 2, alpha = 0.5) + 
  plot_theme() + ggtitle("GSE72165") + theme(legend.position = "bottom") + 
  scale_color_manual(values = c("blue"), name = "") + 
  theme(legend.background = element_rect(fill = "white"), legend.key = element_blank()) + 
  xlab("simplesum_salmon, logFC") + ylab("scaledTPM_salmon, logFC") + 
  guides(colour = guide_legend(override.aes = list(size = 0)))
```

```
ggplot(df2, aes(x = simplesum_salmon_logFC, y = scaledTPM_salmon_logFC, col = allzero_onecond)) + 
  geom_abline(intercept = 0, slope = 1) + 
  geom_point(size = 2, alpha = 0.5) + 
  plot_theme() + ggtitle("GSE72165") + theme(legend.position = "bottom") + 
  scale_color_manual(values = c("blue", "red"), name = "") + 
  xlab("simplesum_salmon, logFC") + ylab("scaledTPM_salmon, logFC") + 
  guides(colour = guide_legend(override.aes = list(size = 7)))
```

```
ggplot(subset(df2, !is.na(scaledTPM_salmon_logCPMbinary)), 
       aes(x = simplesum_salmon_logFC, y = scaledTPM_salmon_logFC, 
           col = scaledTPM_salmon_logCPMbinary)) + 
  geom_abline(intercept = 0, slope = 1) + 
  geom_point(size = 2, alpha = 0.5) + 
  facet_wrap(~scaledTPM_salmon_logCPMbinary) + 
  plot_theme() + ggtitle("GSE72165") + theme(legend.position = "bottom") + 
  xlab("simplesum_salmon, logFC") + ylab("scaledTPM_salmon, logFC") + 
  scale_color_manual(values = c("red", "blue"), name = "scaledTPM_salmon, logCPM") + 
  guides(colour = guide_legend(override.aes = list(size = 7)))
```

### DESeq2

(Back to top)

```
res_sal_simplesum_deseq2 <- diff_expression_DESeq2(txi = NULL,
                                                   counts = salmon_quant$geneCOUNT_sal_simplesum, 
                                                   meta = meta, cond_name = "condition", 
                                                   level1 = "carotid", 
                                                   level2 = "medulla", 
                                                   sample_name = "srr.id")
res_sal_simplesum_avetxl_deseq2 <- diff_expression_DESeq2(txi = salmon_quant$txi_salmonsimplesum,
                                                          counts = NULL, 
                                                          meta = meta, cond_name = "condition", 
                                                          level1 = "carotid", 
                                                          level2 = "medulla", 
                                                          sample_name = "srr.id")
res_sal_scaledTPM_deseq2 <- diff_expression_DESeq2(txi = NULL,
                                                   counts = salmon_quant$geneCOUNT_sal_scaledTPM, 
                                                   meta = meta, cond_name = "condition", 
                                                   level1 = "carotid", 
                                                   level2 = "medulla", 
                                                   sample_name = "srr.id")
```

#### Diagnostics

(Back to top)

```
dfh <- data.frame(pvalue = c(res_sal_scaledTPM_deseq2$res$pvalue,
                             res_sal_simplesum_avetxl_deseq2$res$pvalue,
                             res_sal_simplesum_deseq2$res$pvalue),
                  mth = c(rep("scaledTPM_salmon, DESeq2", nrow(res_sal_scaledTPM_deseq2$res)),
                          rep("simplesum_salmon_avetxl, DESeq2", nrow(res_sal_simplesum_avetxl_deseq2$res)),
                          rep("simplesum_salmon, DESeq2", nrow(res_sal_simplesum_deseq2$res))))
ggplot(dfh, aes(x = pvalue)) + geom_histogram() + facet_wrap(~mth) + 
  plot_theme() + 
  xlab("p-value") + ylab("count")
```

```
par(mfrow = c(1, 3))
plotDispEsts(res_sal_scaledTPM_deseq2$dsd, main = "scaledTPM_salmon, DESeq2")
plotDispEsts(res_sal_simplesum_avetxl_deseq2$dsd, main = "simplesum_salmon_avetxl, DESeq2")
plotDispEsts(res_sal_simplesum_deseq2$dsd, main = "simplesum_salmon, DESeq2")
```

```
par(mfrow = c(1, 3))
DESeq2::plotMA(res_sal_scaledTPM_deseq2$dsd, main = "scaledTPM_salmon, DESeq2")
DESeq2::plotMA(res_sal_simplesum_avetxl_deseq2$dsd, main = "simplesum_salmon_avetxl, DESeq2")
DESeq2::plotMA(res_sal_simplesum_deseq2$dsd, main = "simplesum_salmon, DESeq2")
```

```
par(mfrow = c(1, 1))
```

#### Comparison of significant genes found with different matrices

(Back to top)

```
cobra_deseq2 <- COBRAData(padj = data.frame(simplesum_salmon = res_sal_simplesum_deseq2$res$padj, 
                                            row.names = rownames(res_sal_simplesum_deseq2$res)))
cobra_deseq2 <- COBRAData(padj = data.frame(scaledTPM_salmon = res_sal_scaledTPM_deseq2$res$padj, 
                                            row.names = rownames(res_sal_scaledTPM_deseq2$res)),
                          object_to_extend = cobra_deseq2)
cobra_deseq2 <- COBRAData(padj = data.frame(simplesum_salmon_avetxl = res_sal_simplesum_avetxl_deseq2$res$padj, 
                                            row.names = rownames(res_sal_simplesum_avetxl_deseq2$res)),
                          object_to_extend = cobra_deseq2)
cobraperf_deseq2 <- calculate_performance(cobra_deseq2, aspects = "overlap", thr_venn = 0.05)

cobraplot1_deseq2 <- prepare_data_for_plot(cobraperf_deseq2, incltruth = FALSE, 
                                           colorscheme = c("blue", "red", "black"))
plot_overlap(cobraplot1_deseq2, cex = c(1, 0.7, 0.7))
title("GSE72165, DESeq2", line = 0)
```

#### Comparison of logFC estimates - all methods

(Back to top)

```
df1 <- Reduce(function(...) merge(..., by = "gene", all = TRUE), 
              list(data.frame(gene = rownames(res_sal_scaledTPM_deseq2$res),
                              scaledTPM_salmon = res_sal_scaledTPM_deseq2$res$log2FoldChange,
                              stringsAsFactors = FALSE),
                   data.frame(gene = rownames(res_sal_simplesum_deseq2$res),
                              simplesum_salmon = res_sal_simplesum_deseq2$res$log2FoldChange, 
                              stringsAsFactors = FALSE),
                   data.frame(gene = rownames(res_sal_simplesum_avetxl_deseq2$res),
                              simplesum_salmon_avetxl =
                                res_sal_simplesum_avetxl_deseq2$res$log2FoldChange,
                              stringsAsFactors = FALSE)))
rownames(df1) <- df1$gene
df1$gene <- NULL
pairs(df1, upper.panel = panel_smooth, lower.panel = panel_cor)
```

#### Comparison of logFC estimates - simplesum vs scaledTPM

(Back to top)

```
df2 <- Reduce(function(...) merge(..., by = "gene", all = TRUE), 
              list(data.frame(gene = rownames(salmon_quant$geneTPM_sal),
                              salmon_quant$geneTPM_sal,
                              stringsAsFactors = FALSE),
                   data.frame(gene = rownames(res_sal_scaledTPM_deseq2$res),
                              scaledTPM_salmon_logFC = res_sal_scaledTPM_deseq2$res$log2FoldChange,
                              scaledTPM_salmon_basemean = res_sal_scaledTPM_deseq2$res$baseMean, 
                              stringsAsFactors = FALSE),
                   data.frame(gene = rownames(res_sal_simplesum_deseq2$res),
                              simplesum_salmon_logFC = res_sal_simplesum_deseq2$res$log2FoldChange, 
                              simplesum_salmon_basemean = res_sal_simplesum_deseq2$res$baseMean, 
                              stringsAsFactors = FALSE),
                   data.frame(gene = rownames(res_sal_simplesum_avetxl_deseq2$res),
                              simplesum_salmon_avetxl_logFC =
                                res_sal_simplesum_avetxl_deseq2$res$log2FoldChange,
                              simplesum_salmon_avetxl_basemean = 
                                res_sal_simplesum_avetxl_deseq2$res$baseMean, 
                              stringsAsFactors = FALSE)))
rownames(df2) <- df2$gene
df2$gene <- NULL
df2$scaledTPM_salmon_basemeanbinary <- Hmisc::cut2(df2$scaledTPM_salmon_basemean, g = 2)
df2$simplesum_salmon_basemeanbinary <- Hmisc::cut2(df2$simplesum_salmon_basemean, g = 2)
df2$simplesum_salmon_avetxl_basemeanbinary <- Hmisc::cut2(df2$simplesum_salmon_avetxl_basemean, g = 2)
df2$sumA <- rowSums(df2[, meta$srr.id[meta$condition == "carotid"]])
df2$sumB <- rowSums(df2[, meta$srr.id[meta$condition == "medulla"]])
df2$allzero_onecond <- "expressed in both groups"
df2$allzero_onecond[union(which(df2$sumA == 0), which(df2$sumB == 0))] <- "expressed in one group"
df2$onecol <- rep("", nrow(df2))

ggplot(df2, aes(x = simplesum_salmon_logFC, y = scaledTPM_salmon_logFC, col = onecol)) + 
  geom_abline(intercept = 0, slope = 1) + 
  geom_point(size = 2, alpha = 0.5) + 
  plot_theme() + ggtitle("GSE72165") + theme(legend.position = "bottom") + 
  scale_color_manual(values = c("blue"), name = "") + 
  theme(legend.background = element_rect(fill = "white"), legend.key = element_blank()) + 
  xlab("simplesum_salmon, logFC") + ylab("scaledTPM_salmon, logFC") + 
  guides(colour = guide_legend(override.aes = list(size = 0)))
```

```
ggplot(df2, aes(x = simplesum_salmon_logFC, y = scaledTPM_salmon_logFC, col = allzero_onecond)) + 
  geom_abline(intercept = 0, slope = 1) + 
  geom_point(size = 2, alpha = 0.5) + 
  plot_theme() + ggtitle("GSE72165") + theme(legend.position = "bottom") + 
  scale_color_manual(values = c("blue", "red"), name = "") + 
  xlab("simplesum_salmon, logFC") + ylab("scaledTPM_salmon, logFC") + 
  guides(colour = guide_legend(override.aes = list(size = 7)))
```

```
ggplot(subset(df2, !is.na(scaledTPM_salmon_basemeanbinary)), 
       aes(x = simplesum_salmon_logFC, y = scaledTPM_salmon_logFC, 
           col = scaledTPM_salmon_basemeanbinary)) + 
  geom_abline(intercept = 0, slope = 1) + 
  geom_point(size = 2, alpha = 0.5) + 
  facet_wrap(~scaledTPM_salmon_basemeanbinary) + 
  plot_theme() + ggtitle("GSE72165") + theme(legend.position = "bottom") + 
  xlab("simplesum_salmon, logFC") + ylab("scaledTPM_salmon, logFC") + 
  scale_color_manual(values = c("red", "blue"), name = "scaledTPM_salmon, base mean") + 
  guides(colour = guide_legend(override.aes = list(size = 7)))
```

## DTU analysis on Salmon counts, with DEXSeq

(Back to top)

```
BPPARAM = MulticoreParam(6)
stopifnot(all(colnames(salmon_quant$txCOUNT_sal) == rownames(meta)))
dxd <- DEXSeqDataSet(countData = round(salmon_quant$txCOUNT_sal), sampleData = meta, 
                     design = ~sample + exon + condition:exon,
                     featureID = rownames(salmon_quant$txCOUNT_sal),
                     groupID = tx2gene$gene[match(rownames(salmon_quant$txCOUNT_sal), 
                                                  tx2gene$transcript)])
dxd <- estimateSizeFactors(dxd)
dxd <- estimateDispersions(dxd, BPPARAM = BPPARAM)
```

```
## using supplied model matrix 
## using supplied model matrix 
## using supplied model matrix 
## using supplied model matrix 
## using supplied model matrix 
## using supplied model matrix 
## using supplied model matrix 
## using supplied model matrix 
## using supplied model matrix 
## using supplied model matrix 
## using supplied model matrix 
## using supplied model matrix
```

```
plotDispEsts(dxd)
```

```
dxd <- testForDEU(dxd, BPPARAM = BPPARAM)
```

```
## using supplied model matrix 
## using supplied model matrix 
## using supplied model matrix 
## using supplied model matrix 
## using supplied model matrix 
## using supplied model matrix
```

```
dxr <- DEXSeqResults(dxd)
qval_dtu_salmon <- perGeneQValue(dxr)
table(qval_dtu_salmon <= 0.05, useNA = "ifany")
```

```
## 
## FALSE  TRUE 
##  9886  1022
```

## Help functions

(Back to top)

```
panel_cor <- function(x, y, digits = 3, cex.cor) {
  ## Panel function to print Pearson and Spearman correlations
  usr <- par("usr")
  on.exit(par(usr))
  par(usr = c(0, 1, 0, 1))
  r1 <- abs(cor(x, y, method = "pearson", use = "complete"))
  txt1 <- format(c(r1, 0.123456789), digits = digits)[1]
  r2 <- abs(cor(x, y, method = "spearman", use = "complete"))
  txt2 <- format(c(r2, 0.123456789), digits = digits)[1]
  text(0.5, 0.35, paste("pearson =", txt1), cex = 1.1)
  text(0.5, 0.65, paste("spearman =", txt2), cex = 1.1)
}
```

```
panel_smooth<-function (x, y, col = "blue", bg = NA, pch = ".", 
                        cex = 0.8, ...) {
  ## Panel function to plot points
  points(x, y, pch = pch, col = col, bg = bg, cex = cex)
}
```

```
plot_theme <- function() {
  ## ggplot2 plotting theme
  theme_grey() +
    theme(legend.position = "right",
          panel.background = element_rect(fill = "white", colour = "black"),
          panel.grid.minor.x = element_blank(),
          panel.grid.minor.y = element_blank(),
          strip.text = element_text(size = 10),
          strip.background = element_rect(fill = NA, colour = "black"),
          axis.text.x = element_text(size = 10),
          axis.text.y = element_text(size = 10),
          axis.title.x = element_text(size = 15),
          axis.title.y = element_text(size = 15),
          plot.title = element_text(colour = "black", size = 20))
}
```

```
calc_lengths_mapping <- function(gtf, cdna_fasta, feature_lengths_file,
                                 tx_gene_file) {
  ## Function to calculate gene and transcript lengths from transcript cDNA 
  ## fasta and gtf file. Also generate mapping between transcript and gene IDs.
  
  suppressPackageStartupMessages(library(GenomicFeatures))
  suppressPackageStartupMessages(library(Biostrings))
  
  ## Gene/transcript lengths ===============================================
  ## Transcripts/genes present in gtf file
  if (!is.null(gtf)) {
    txdb <- makeTxDbFromGFF(gtf, format = "gtf")
    ebg <- exonsBy(txdb, "gene")
    ebt <- exonsBy(txdb, "tx", use.names = TRUE)
    ebg_red <- reduce(ebg)
    gene_length <- sum(width(ebg_red))
    ebt_red <- reduce(ebt)
    tx_length <- sum(width(ebt_red))
  } else {
    tx_length <- c()
    gene_length <- c()
  }  
  
  ## Extend with transcripts from cDNA fasta
  cdna <- readDNAStringSet(gsub("\\.gz", "", cdna_fasta))
  tx_length2 <- width(cdna)
  names(tx_length2) <- sapply(names(cdna), function(i) strsplit(i, " ")[[1]][1])
  tx_length2 <- tx_length2[setdiff(names(tx_length2), names(tx_length))]
  tx_length <- c(tx_length, tx_length2)
  
  ## Gene/transcript mapping ===============================================
  ## Transcripts/genes present in gtf file
  if (!is.null(gtf)) {
    tbg <- transcriptsBy(txdb, "gene")
    tx2gene <- stack(lapply(tbg, function(w) w$tx_name))
    colnames(tx2gene) <- c("transcript", "gene")
  } else {
    tx2gene <- data.frame(transcript = c(), gene = c())
  }  
  
  ## Extend with mappings from cDNA fasta file
  tx <- sapply(names(cdna), function(i) strsplit(i, " ")[[1]][1])
  gn <- sapply(names(cdna), function(i) gsub("gene:", "", strsplit(i, " ")[[1]][4]))
  tx2gene2 <- data.frame(transcript = tx, gene = gn, 
                         stringsAsFactors = FALSE)
  rownames(tx2gene2) <- NULL
  tx2gene2 <- tx2gene2[match(setdiff(tx2gene2$transcript, 
                                     tx2gene$transcript), tx2gene2$transcript), ]
  tx2gene <- rbind(tx2gene, tx2gene2)
  
  gene2tx <- tx2gene[, c("gene", "transcript")]
  
  save(gene_length, tx_length, file = feature_lengths_file)
  save(gene2tx, tx2gene, file = tx_gene_file)
}
```

```
diff_expression_edgeR <- function(counts, meta, cond_name, sample_name, 
                                  gene_length_matrix = NULL) {
  ## Differential expression analysis with edgeR
  
  suppressPackageStartupMessages(library(edgeR))

  ## Prepare DGEList
  counts <- round(counts)
  cts <- counts[rowSums(is.na(counts)) == 0, ]
  cts <- cts[rowSums(cts) != 0, ]
  dge <- 
    DGEList(counts = cts, group = meta[, cond_name][match(colnames(cts), 
                                                          meta[, sample_name])])
  
  ## If average transcript lengths provided, add as offset
  if (!is.null(gene_length_matrix)) {
    egf <- gene_length_matrix[match(rownames(cts), rownames(gene_length_matrix)),
                              match(colnames(cts), colnames(gene_length_matrix))]
    egf <- egf / exp(rowMeans(log(egf)))
    o <- log(calcNormFactors(cts/egf)) + log(colSums(cts/egf))
    dge$offset <- t(t(log(egf)) + o)
  } else {
    dge <- calcNormFactors(dge)
  }
  
  ## Estimate dispersions and fit model
  design <- model.matrix(~dge$samples$group)
  dge <- estimateGLMCommonDisp(dge, design = design)
  dge <- estimateGLMTrendedDisp(dge, design = design)
  dge <- estimateGLMTagwiseDisp(dge, design = design)
  fit <- glmFit(dge, design = design)
  lrt <- glmLRT(fit)
  tt <- topTags(lrt, n = Inf)$table
  return(list(dge = dge, tt = tt))
}
```

```
diff_expression_DESeq2 <- function(txi = NULL, counts, meta, cond_name, 
                                   level1, level2, sample_name) {
  ## Differential expression analysis with DESeq2
  
  suppressPackageStartupMessages(library(DESeq2, 
                                         lib.loc = "/home/charlotte/R/x86_64-pc-linux-gnu-library/3.2"))

  ## If tximport object provided, generate DESeqDataSet from it. Otherwise, 
  ## use the provided count matrix.
  if (!is.null(txi)) {
    txi$counts <- round(txi$counts)
    keep_feat <- rownames(txi$counts[rowSums(is.na(txi$counts)) == 0 & rowSums(txi$counts) != 0, ])
    txi <- lapply(txi, function(w) {
      if (!is.null(dim(w))) w[match(keep_feat, rownames(w)), ]
      else w
      })
    dsd <- DESeqDataSetFromTximport(txi, 
                                    colData = meta[match(colnames(txi$counts), 
                                                         meta[, sample_name]), ],
                                    design = as.formula(paste0("~", cond_name)))
  } else {
    counts <- round(counts)
    cts = counts[rowSums(is.na(counts)) == 0, ]
    cts <- cts[rowSums(cts) != 0, ]
    dsd <- DESeqDataSetFromMatrix(countData = round(cts), 
                                  colData = meta[match(colnames(cts), 
                                                       meta[, sample_name]), ],
                                  design = as.formula(paste0("~", cond_name)))
  }
  
  ## Estimate dispersions and fit model
  dsd <- DESeq(dsd, test = "Wald", fitType = "local", betaPrior = TRUE)
  res <- as.data.frame(results(dsd, contrast = c(cond_name, level2, level1),
                               cooksCutoff = FALSE, independentFiltering = FALSE))
  return(list(dsd = dsd, res = res))
}
```

## Session info

(Back to top)

```
sessionInfo()
```

```
## R version 3.2.2 (2015-08-14)
## Platform: x86_64-pc-linux-gnu (64-bit)
## Running under: Ubuntu 14.04.3 LTS
## 
## locale:
##  [1] LC_CTYPE=C                 LC_NUMERIC=C              
##  [3] LC_TIME=en_CA.UTF-8        LC_COLLATE=en_CA.UTF-8    
##  [5] LC_MONETARY=en_CA.UTF-8    LC_MESSAGES=en_CA.UTF-8   
##  [7] LC_PAPER=en_CA.UTF-8       LC_NAME=C                 
##  [9] LC_ADDRESS=C               LC_TELEPHONE=C            
## [11] LC_MEASUREMENT=en_CA.UTF-8 LC_IDENTIFICATION=C       
## 
## attached base packages:
## [1] parallel  stats4    stats     graphics  grDevices utils     datasets 
## [8] methods   base     
## 
## other attached packages:
##  [1] edgeR_3.12.0               limma_3.26.3              
##  [3] DEXSeq_1.16.7              DESeq2_1.11.6             
##  [5] RcppArmadillo_0.6.400.2.2  Rcpp_0.12.2               
##  [7] SummarizedExperiment_1.0.2 Biobase_2.30.0            
##  [9] GenomicRanges_1.22.3       GenomeInfoDb_1.6.2        
## [11] IRanges_2.4.6              S4Vectors_0.8.6           
## [13] BiocGenerics_0.16.1        BiocParallel_1.4.3        
## [15] dplyr_0.4.3                iCOBRA_0.99.4             
## [17] tximport_0.0.7             ggplot2_1.0.1             
## 
## loaded via a namespace (and not attached):
##  [1] bitops_1.0-6         RColorBrewer_1.1-2   tools_3.2.2         
##  [4] R6_2.1.1             DT_0.1               rpart_4.1-10        
##  [7] KernSmooth_2.23-15   Hmisc_3.17-1         DBI_0.3.1           
## [10] colorspace_1.2-6     nnet_7.3-11          gridExtra_2.0.0     
## [13] formatR_1.2.1        labeling_0.3         caTools_1.17.1      
## [16] scales_0.3.0         genefilter_1.52.0    stringr_1.0.0       
## [19] digest_0.6.9         Rsamtools_1.22.0     shinyBS_0.61        
## [22] foreign_0.8-66       rmarkdown_0.9.2      XVector_0.10.0      
## [25] htmltools_0.3        htmlwidgets_0.5      RSQLite_1.0.0       
## [28] shiny_0.13.0         hwriter_1.3.2        gtools_3.5.0        
## [31] acepack_1.3-3.3      RCurl_1.95-4.7       magrittr_1.5        
## [34] Formula_1.2-1        futile.logger_1.4.1  munsell_0.4.2       
## [37] proto_0.3-10         stringi_1.0-1        yaml_2.1.13         
## [40] MASS_7.3-45          zlibbioc_1.16.0      gplots_2.17.0       
## [43] plyr_1.8.3           grid_3.2.2           gdata_2.17.0        
## [46] shinydashboard_0.5.1 lattice_0.20-33      Biostrings_2.38.3   
## [49] splines_3.2.2        annotate_1.48.0      locfit_1.5-9.1      
## [52] knitr_1.12.3         geneplotter_1.48.0   reshape2_1.4.1      
## [55] biomaRt_2.26.1       futile.options_1.0.0 XML_3.98-1.3        
## [58] evaluate_0.8         latticeExtra_0.6-26  lambda.r_1.1.7      
## [61] httpuv_1.3.3         gtable_0.1.2         assertthat_0.1      
## [64] mime_0.4             xtable_1.8-0         survival_2.38-3     
## [67] AnnotationDbi_1.32.3 cluster_2.0.3        statmod_1.4.23      
## [70] ROCR_1.0-7
```
