# Supplementary material for: Differential analyses for RNA-seq: transcript-level estimates improve gene-level inferences
Source: F1000Res. 2016 Feb 29;4:1521. Originally published 2015 Dec 30. [Version 2] doi: 10.12688/f1000research.7563.2 (PMC4712774; doi:10.12688/f1000research.7563.2)
Supplement: Supplementary file 7 [file f1000research-4-8770-s0006.tgz › 9487b780-1cec-4a38-bd8a-e4c4ed5e7c5a.pdf]

Differential analyses for RNA-seq: transcript-level estimates improve gene-level inferences
Supplementary Material

Charlotte Soneson, Michael I. Love, Mark D. Robinson

Contents

1 Simulation details, sim2 data set **1**

2 Experimental data sets **2**

3 Quantification **2**

4 Deriving offsets from transcript lengths and abundances **3**

5 Generation of incomplete transcriptome annotation **4**

6 Accuracy of abundance estimates among paralogous genes **4**

7 Supplementary Figures **7**

1 Simulation details, sim2 data set

The **sim2** data set consists of simulated sequencing reads from the human chromosome 1. The sequencing parameters as well as underlying TPM values for the 15,677 transcripts in one of the two simulated conditions were estimated using RSEM v1.2.21 [6] from the *ERS326990* sample from the ArrayExpress data set with accession number *E_MTAB_1733*. We simulated three biological replicates from each of two conditions.

Three types of differential abundance were introduced between the two conditions, all assigned to genes with estimated TPMs between 0.1 and 1000 in the reference data set (the "eligible" genes):

- **DGE** (differential gene expression) was introduced for 420 (11%) of the eligible genes. For these genes, the overall gene expression (TPM) was modified, but the relative isoform abundances within the genes were kept constant. The TPMs of the genes in condition 2 were obtained by shuffling the TPMs of the genes in condition 1, in order to create a range of fold changes and keep the total TPM for differential genes constant across samples. All genes with simulated DGE also show DTE (differential transcript expression), but no DTU (differential transcript usage).

- **DTE** (differential transcript expression) was introduced for 422 (11%) of the eligible genes (selected among genes with at least two isoforms). For each of these genes, 10% of the isoforms were selected to be differential between the conditions, again by shuffling their TPMs from condition 1 to generate TPMs in condition 2. In total, 528 transcripts were differentially expressed in these genes. All genes with simulated DTE also show DGE (since the total TPM of the gene is modified) and DTU (since the relative usage of the isoforms is modified). However, the overall fold changes of the DTE genes is often lower than those of the DGE genes, since only a subset of the transcripts are affected.

- **DTU** (differential transcript usage) was introduced for 420 (11%) of the eligible genes (selected among genes with at least two isoforms). For each of these genes, the total TPM was kept constant between

the two conditions, but the relative isoform usage for a given gene with DTU in condition 2 was sampled randomly among the relative isoform usages for all genes in the data set with the same number of isoforms. All genes with simulated DTU also show DTE (since at least one isoform changes expression), but no DGE.

Based on the expected number of reads for each transcript in each condition, we simulated three biological replicates per condition from a negative binomial distribution, using a mean-dispersion relationship estimated from large experimental data sets (see [9] for more information on how these estimates were generated). The individual sample counts were then converted to TPMs and used as inputs to RSEM to simulate 10,000,000 paired-end reads of length 100 bp for each sample.

From the three types of differential genes in this data set, we can also define three sets of "truly differential" features:

- Differentially expressed transcripts
- Genes with between-condition differences in overall expression (the sum of the transcript TPMs)
- Genes with between-condition differences in at least one associated transcript

As we show in the manuscript, it is important to select the type of interest before choosing a quantification and representation method, since different methods have unequal power to detect different types of signals.

2 Experimental data sets

In addition to the two simulated data sets, the results presented in this manuscripts are based on the following four experimental data sets:

- **Bottomly** [1] This data set contains striatal tissue samples from 11 mice of the DBA/2J strain and 10 mice of the C57BL/6J strain. We downloaded the FASTQ files with sequencing reads from SRA (see Supplementary File 4 for exact file names and URLs) and used the GRCm38.82 Ensembl annotation for analysis.
- **GSE64570** [11] We use a subset of the data, consisting of 3 replicates of wildtype zebrafish injected with water, and 3 replicates of tlr5a morphants injected with Flagellin. We downloaded the FASTQ files from SRA (see Supplementary File 5 for exact file names and URLs) and used the GRz10 Ensembl annotation for analysis.
- **GSE69244** [4] Also here, we use a subset of the data, consisting of 3 replicates of 10-month old SAMP8 mice given vehicle diet for 7 months and 3 replicates of 10-month old SAMP8 mice treated with the Alzheimer's disease drug candidate J147 for 7 months. We downloaded the FASTQ files from SRA (see Supplementary File 6 for exact file names and URLs) and used the GRCm38.82 Ensembl annotation for analysis.
- **GSE72165** [3] This data set contains 3 samples each from the carotid body and the adrenal medulla in mouse. We downloaded the FASTQ files from SRA (see Supplementary File 7 for exact file names and URLs) and used the GRCm38.82 Ensembl annotation for analysis.

3 Quantification

Abundances were quantified in two different ways:

- Transcript abundance quantification with **Salmon** [8]. We generated a Salmon index from the cDNA fasta file from Ensembl GRCh37.71 (for the human data sets), Ensembl GRCm38.82 (for the mouse data sets) or Ensembl GRz10 (for the zebrafish data set), and used the quasi-alignment approach to obtain TPM and count estimates for each transcript in each of the samples. In order to estimate the variability in the estimates, we generated 30 bootstrap replicates.

- Gene abundance quantification with **featureCounts** [7], after alignment of the reads to the genome using STAR v2.4.2a [5]. The gene models were defined by the Ensembl GTF files from the same release as the cDNA fasta files used for Salmon.

As an alternative to Salmon, we also applied **kallisto** [2] to obtain TPM and count estimates for transcripts. The results were very similar to those obtained with salmon and are not shown in the manuscript.

Salmon returns estimates of both the TPM and the expected read count for each transcript. Gene-level TPMs are defined as the sum of the TPM estimates of all associated transcripts. For comparison with underlying (true) TPM values, we directly use the estimated (gene- or transcript-level) TPMs returned by Salmon. For DGE analysis, as described in the main manuscript, we define two different "count" matrices (denoted scaledTPM and simplesum) based on the Salmon results. Letting $\widehat{T}_t$ and $\widehat{C}_t$ denote the estimated TPM and read count for a transcript t, respectively, and denoting the set of all transcripts associated with a gene g as $\mathcal{T}_g$, we define the count equivalents for a gene g as follows:

- For scaledTPM,
$$E_g = 10^{-6} \cdot \sum_{t \in \mathcal{T}_g} \widehat{T}_t \cdot \sum_t \widehat{C}_t$$

- For simplesum,
$$E_g = \sum_{t \in \mathcal{T}_g} \widehat{C}_t$$

featureCounts does not return TPM estimates (neither for genes nor for transcripts), but only an observed read count for each gene, which is used as input for the DGE analyses. For comparison with underlying (true) TPM values, we estimate the "scaled FPKM" for each gene. Letting X_g denote the observed count for gene g, and L_g denote the "union-exon length" of the gene (that is, the total number of base pairs covered by the exons of the gene), we define

$$\widehat{FPKM}_g = 10^9 \cdot \frac{X_g}{L_g \cdot \sum_g X_g},$$

and subsequently we define the scaled FPKM value by

$$\widehat{FPKM}^s_g = 10^6 \cdot \frac{\widehat{FPKM}_g}{\sum_g \widehat{FPKM}_g}.$$

4 Deriving offsets from transcript lengths and abundances

Here we motivate using the average transcript length as an offset for performing differential expression on summarized fragment counts from all transcripts of a gene. We use the notation of the Methods Supplement of the *Cuffdiff2* paper [10]. Suppose a single gene G with transcripts indexed by t, two samples a and b with observed fragment counts for the gene G given by X_a and X_b, and underlying, unobserved fragment counts from each transcript t given by X_{ta} and X_{tb}. The effective length of transcript t, the number of positions along the transcript where a fragment can align, is given by $\bar{l}_t$. For notational simplicity, we suppose this is the same for samples, but the following holds for sample-specific effective lengths as well. The true expression of transcript t is given by ρ_{ta} and ρ_{tb} for the two samples.

The fold change in expression for the gene is given by Δ_{ρ_G}. If we knew the true fragment counts for each transcript, we could estimate this using the sum of length corrected counts for all transcripts for sample a and dividing by the same quantity for sample b.

$$\hat{\Delta}_{\rho_G} = \frac{\sum_t X_{ta}/\bar{l}_t}{\sum_t X_{tb}/\bar{l}_t} \quad (1)$$

We define θ_{ta} as the ratio of total expression for gene G from transcript t, such that $\sum_t \theta_{ta} = 1$

$$\theta_{ta} = \frac{\rho_{ta}}{\sum_{t'} \rho_{t'a}}$$

The expected number of fragments coming from transcript t is then given by the following product:

$$E(X_{ta}) = X_a \frac{\theta_{ta}\bar{l}_t}{\sum_{t'} \theta_{t'a}\bar{l}_{t'}}$$

We now return to the numerator of Eq. 1. The expected value of this quantity can be broken down as follows:

$$\begin{aligned} E\left(\sum_t X_{ta}/\bar{l}_t\right) &= \sum_t E(X_{ta})/\bar{l}_t \\ &= \sum_t X_a \frac{\theta_{ta}}{\sum_{t'} \theta_{t'a}\bar{l}_{t'}} \\ &= \frac{X_a}{\sum_{t'} \theta_{t'a}\bar{l}_{t'}} \sum_t \theta_{ta} \\ &= \frac{X_a}{\sum_{t'} \theta_{t'a}\bar{l}_{t'}} \end{aligned}$$

We can therefore estimate Δ_{ρ_G} using expected values for the numerator and denominator:

$$\frac{E\left(\sum_t X_{ta}/\bar{l}_t\right)}{E\left(\sum_t X_{tb}/\bar{l}_t\right)} = \frac{X_a/\sum_{t'} \theta_{t'a}\bar{l}_{t'}}{X_b/\sum_{t'} \theta_{t'b}\bar{l}_{t'}}$$

This final line shows that the ratio of summarized fragment counts for the gene can be corrected for bias due to changes in transcript usage by using the average transcript length for each sample, where the average is weighted by abundances θ_{ta}. As we do not have access to the true abundances of each transcript, estimates of transcript abundance $\hat{\theta}_{ta}$ are used.

5 Generation of incomplete transcriptome annotation

To generate the incomplete transcriptome annotation used to simulate incomplete knowledge about the transcriptome (see Figure 1a), we first randomly selected 20% of the transcripts included in the cDNA fasta file downloaded from Ensembl (restricted to transcripts from chromosome 1 for **sim2**). A new, incomplete transcriptome fasta file was generated by excluding the entries for the selected transcripts. This fasta file was then used to generate the incomplete index for Salmon. In addition, we excluded all entries from the Ensembl GTF file that were annotated with a transcript ID from the selected list. Note that an exon can be included with multiple entries in the Ensembl GTF file if it is part of multiple transcripts, and only the entry corresponding to the excluded transcript was removed. Thus, exonic regions are still included in the GTF file as long as any of the transcripts they are part of is retained. The filtered GTF file was used for counting with featureCounts. The precise R code to generate the incomplete annotation files is included in Dataset 2.

6 Accuracy of abundance estimates among paralogous genes

The two types of abundance quantification pursued in this study (alignment-based with gene-level read counting, alignment-free with transcript-level abundance estimation) can be expected to behave differently, e.g., in their handling of multi-mapping reads. Depending on the settings, such reads are often excluded from traditional gene counting, whereas transcript-level abundance estimation procedures are designed to portion them out appropriately across the matching transcripts. Since this difference may be clearest for sets of similar genes, we analyzed the accuracy of gene-level abundance estimates from Salmon and STAR+featureCounts for several collections of paralogous genes in the simulated data sets. We considered only collections containing at least 10 genes (5 for the **sim2** simulation), at least half of which were truly expressed at a non-trivial level (true TPM > 5). For the **sim2** data set, we considered only subcollections consisting of genes on

chromosome 1. Overall, Salmon showed a higher correlation between the true and observed values for the groups of paralogous genes (a few examples are shown in Supplementary Figures 1-3). However, the difference between the approaches was not strikingly larger among paralogous genes than for the set of all genes (see Dataset 1).

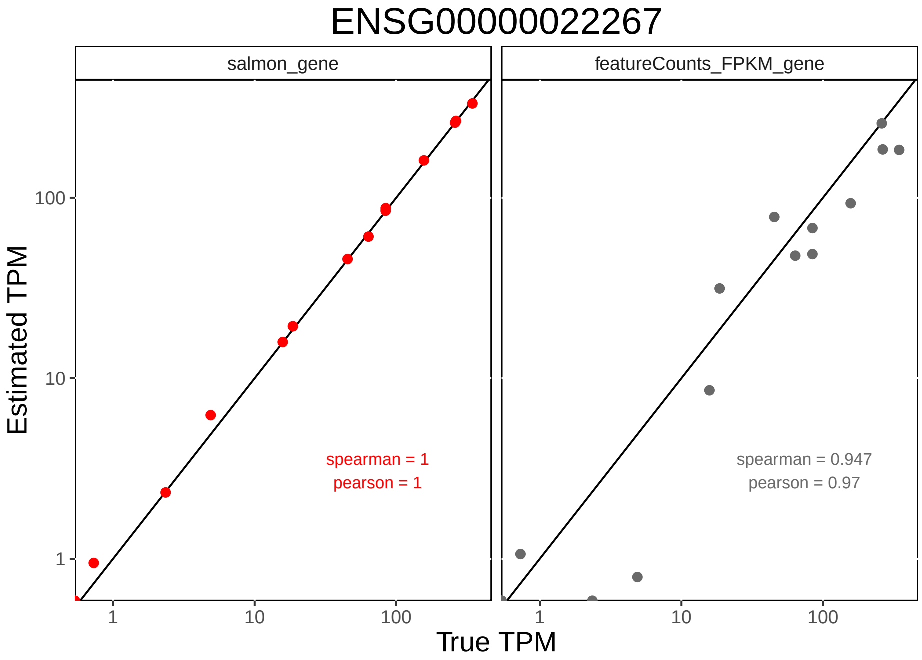

Supplementary Figure 1: (**sim1**) Accuracy of gene-level abundance estimates from Salmon and featureCounts for paralogs of the ENSG00000022267 gene.

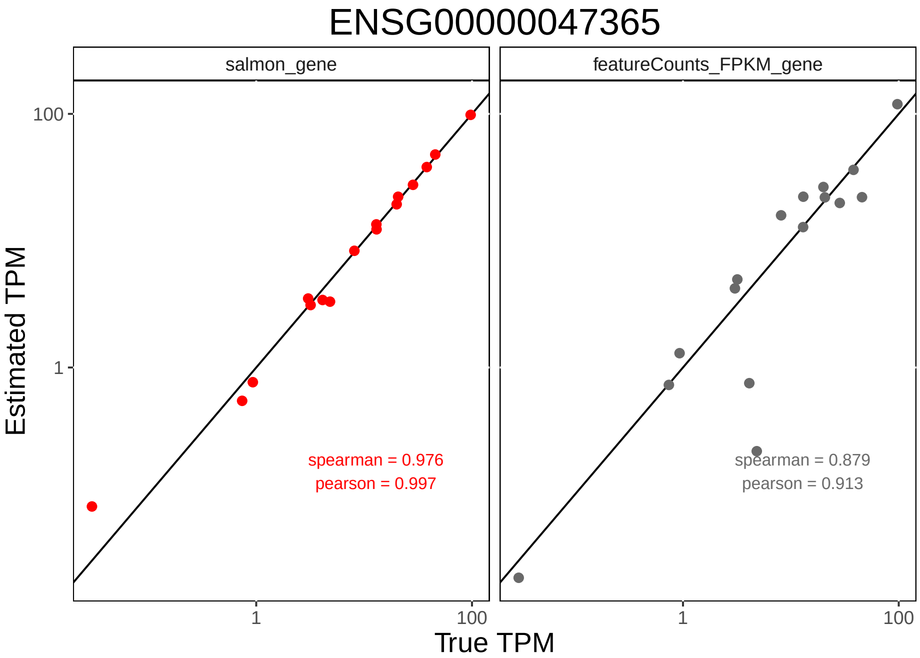

Supplementary Figure 2: (**sim1**) Accuracy of gene-level abundance estimates from Salmon and featureCounts for paralogs of the ENSG00000047365 gene.

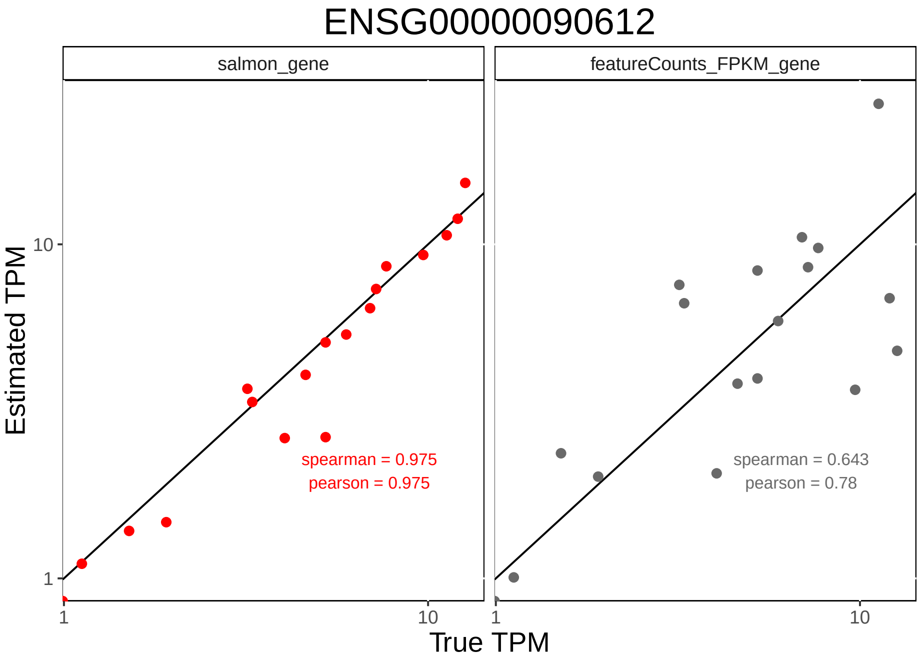

Supplementary Figure 3: (**sim1**) Accuracy of gene-level abundance estimates from Salmon and featureCounts for paralogs of the ENSG00000090612 gene.

7 Supplementary Figures

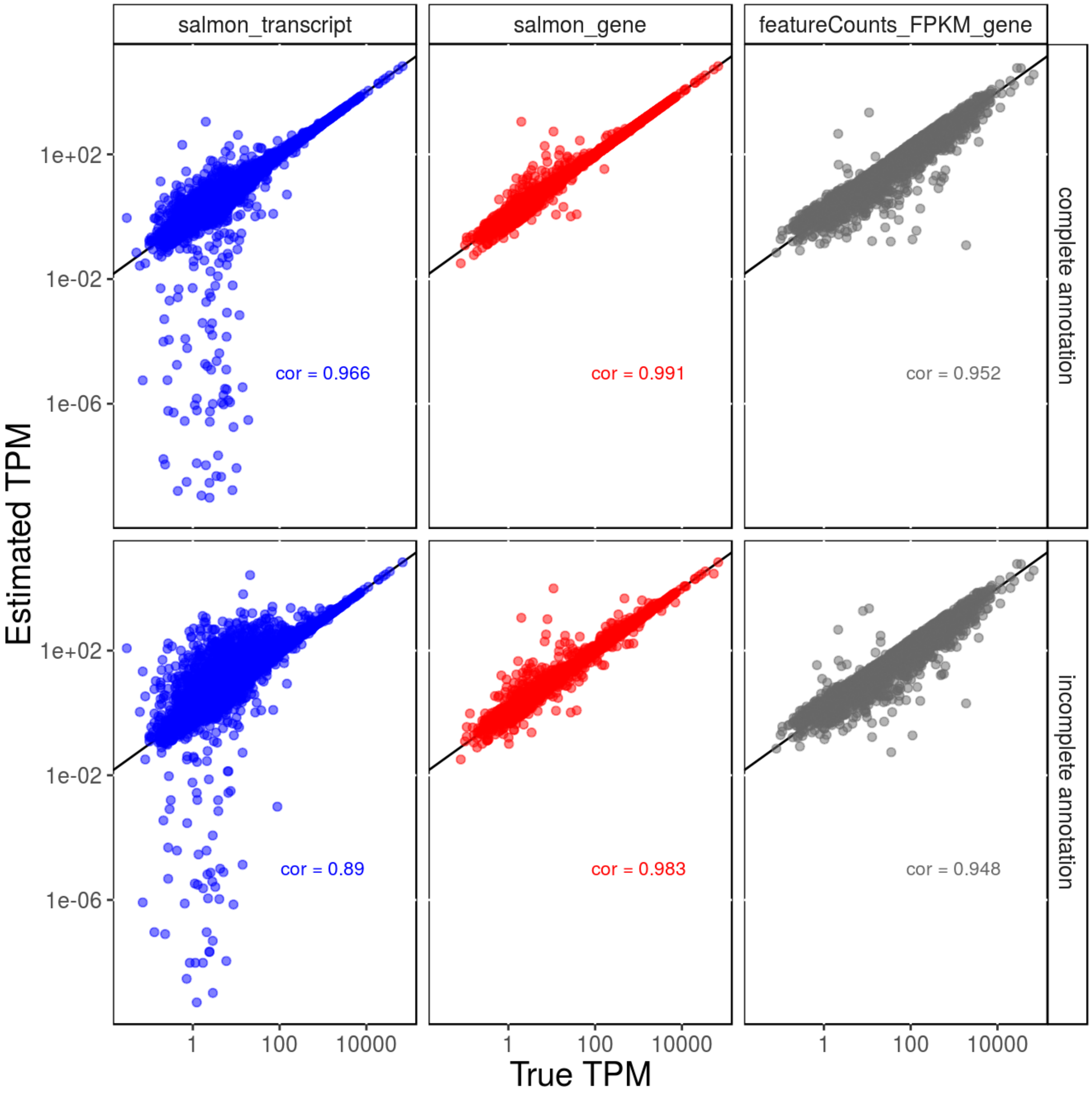

Supplementary Figure 4: Accuracy of transcript and gene TPM estimates from Salmon and featureCounts, restricted to features with non-zero true and estimated TPMs (**sim2**). Spearman correlations are indicated in each panel.

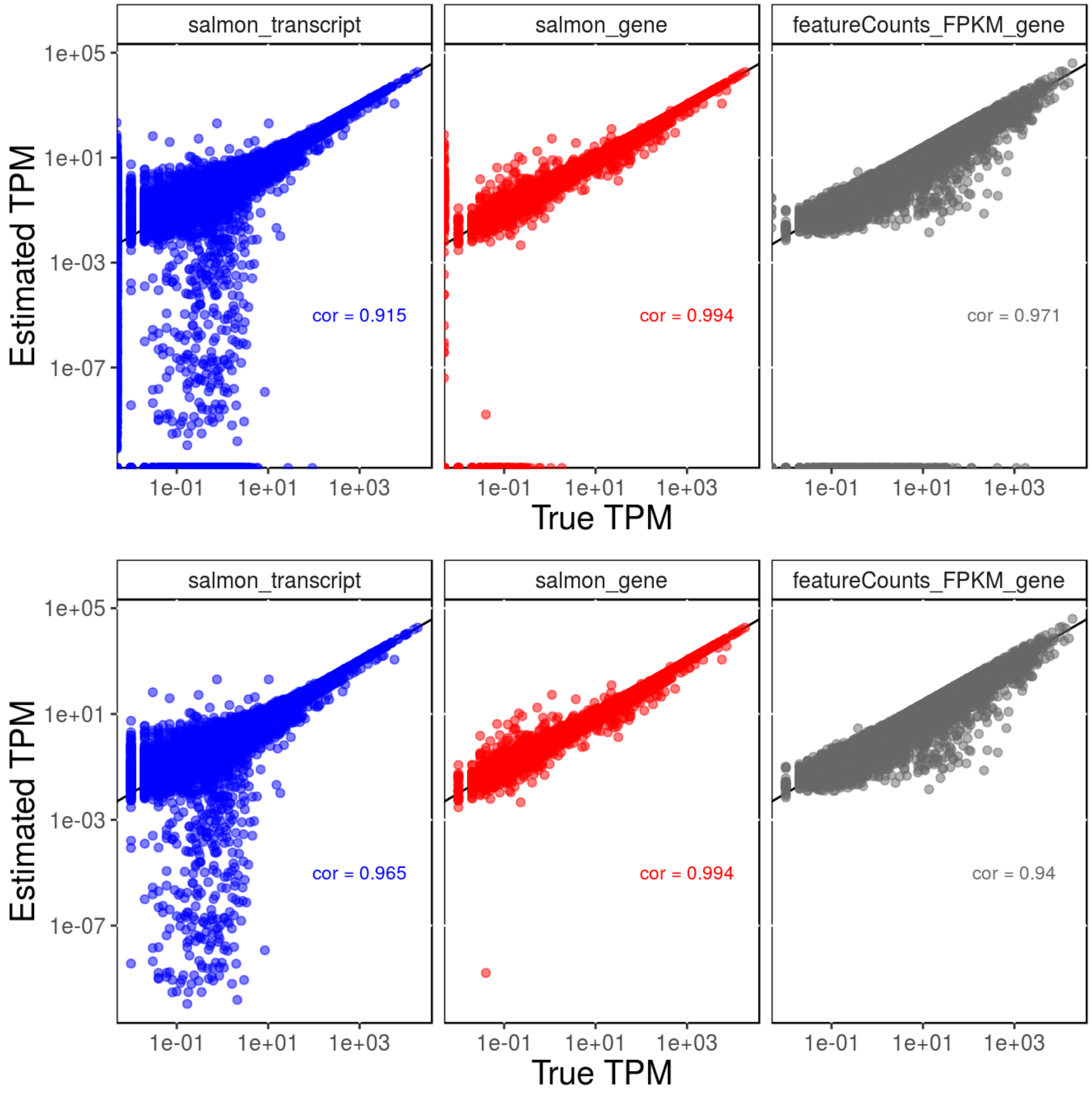

Supplementary Figure 5: Accuracy of transcript and gene TPM estimates from Salmon and featureCounts (**sim1**). Top: all features. Bottom: restricted to features with non-zero true and estimated TPMs. Spearman correlations are indicated in each panel.

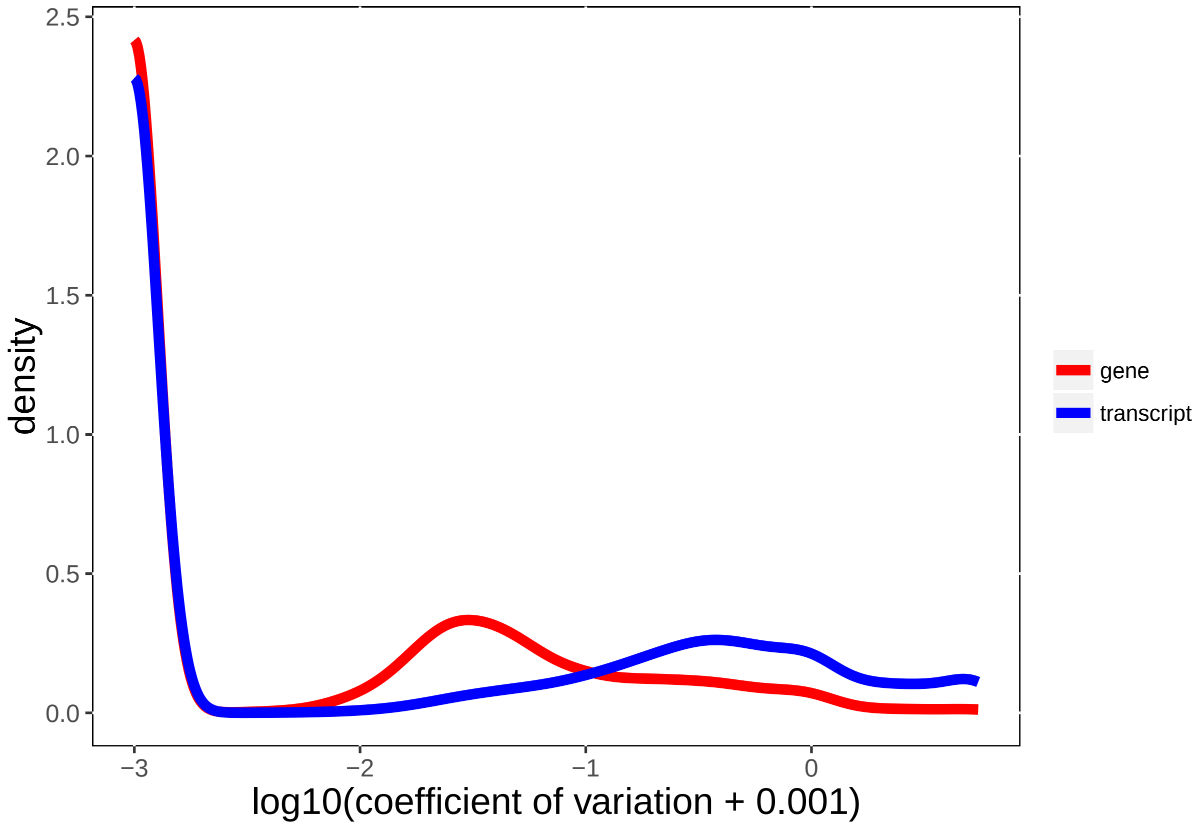

Supplementary Figure 6: Distribution of coefficients of variation for gene and transcript abundance estimates from Salmon (**sim1**), estimated across 30 bootstrap replicates of one of the simulated samples (sample1).

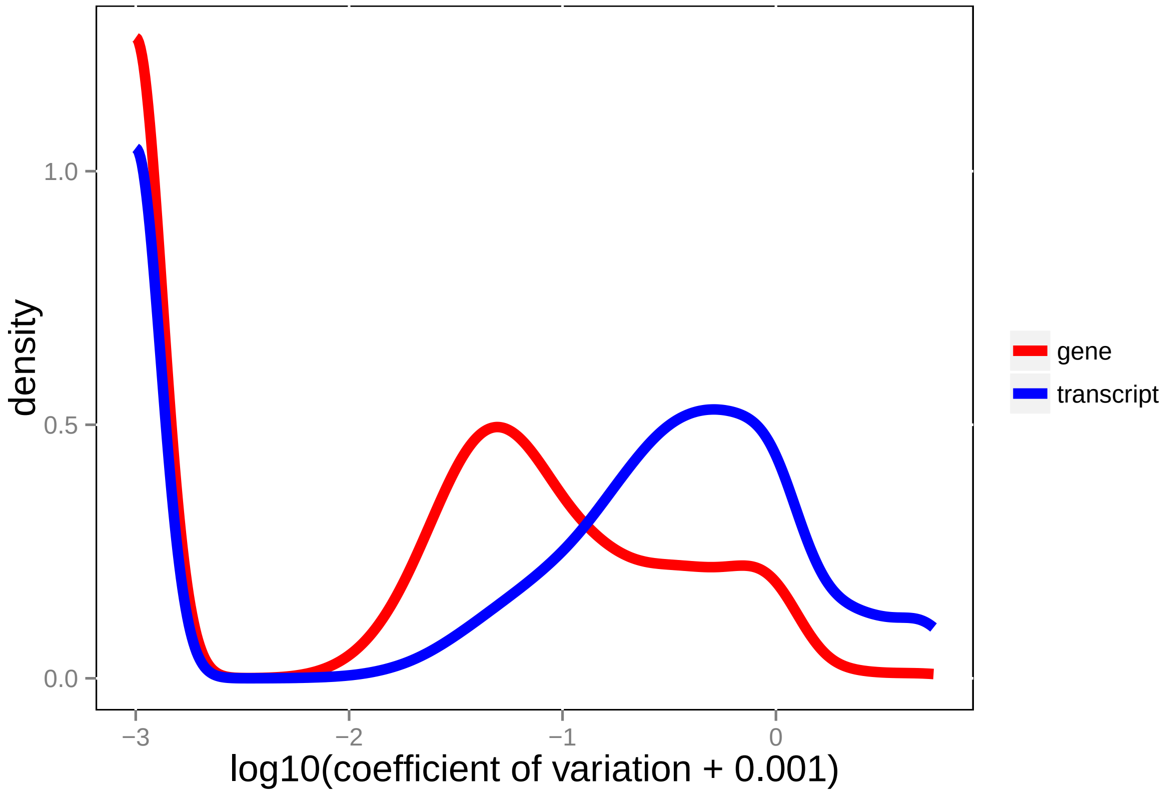

Supplementary Figure 7: Distribution of coefficients of variation for gene and transcript abundance estimates from Salmon (**Bottomly**), estimated across 30 bootstrap replicates of the SRR099223 sample.

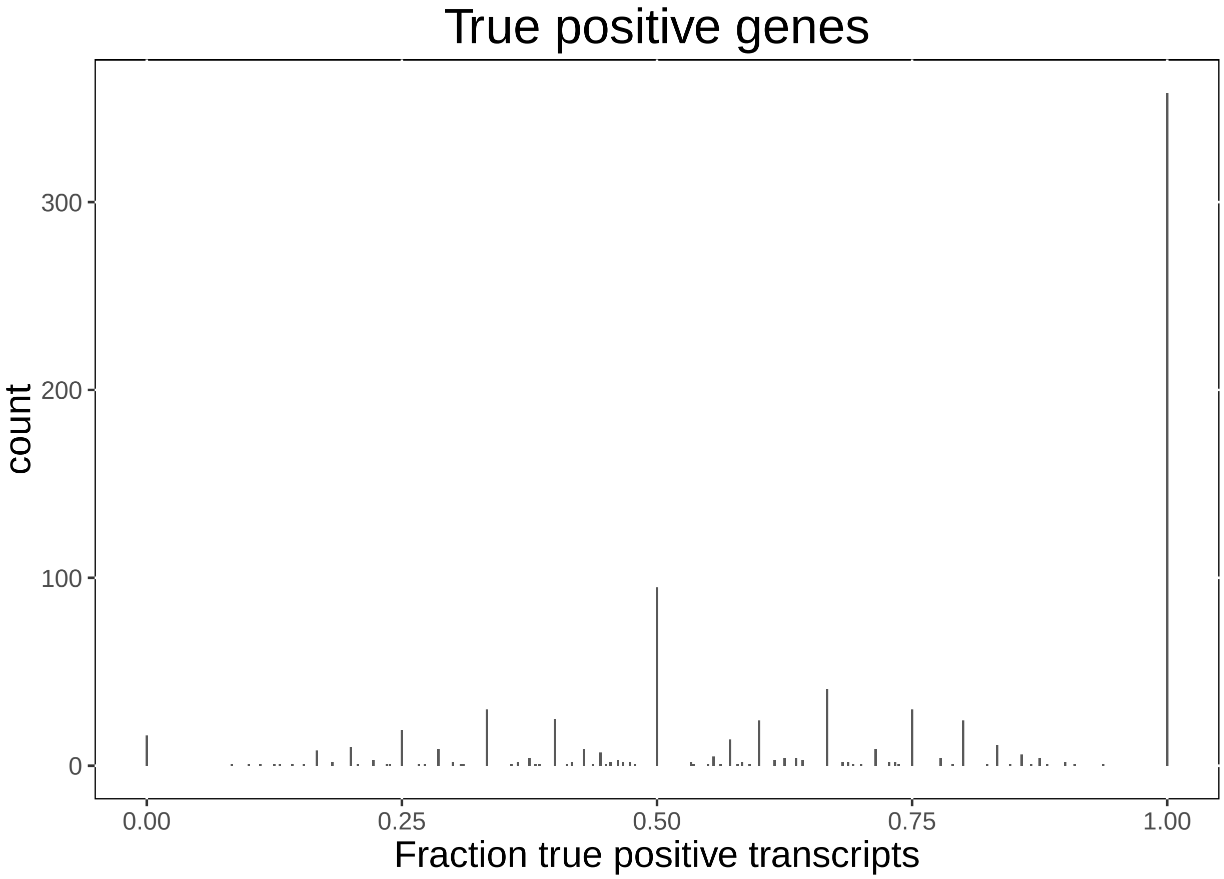

Supplementary Figure 8: Distribution of the fraction of differentially used transcripts that were found for the genes correctly found to harbor DTE, using *edgeR* (**sim2**). While for many of the true positive genes, all of the truly differential transcripts were detected individually as well, this is not true for all the genes, which explains the difference in power between transcript-level DTE and gene-level-summarized DTE that is seen in Figure 2 of the main text.

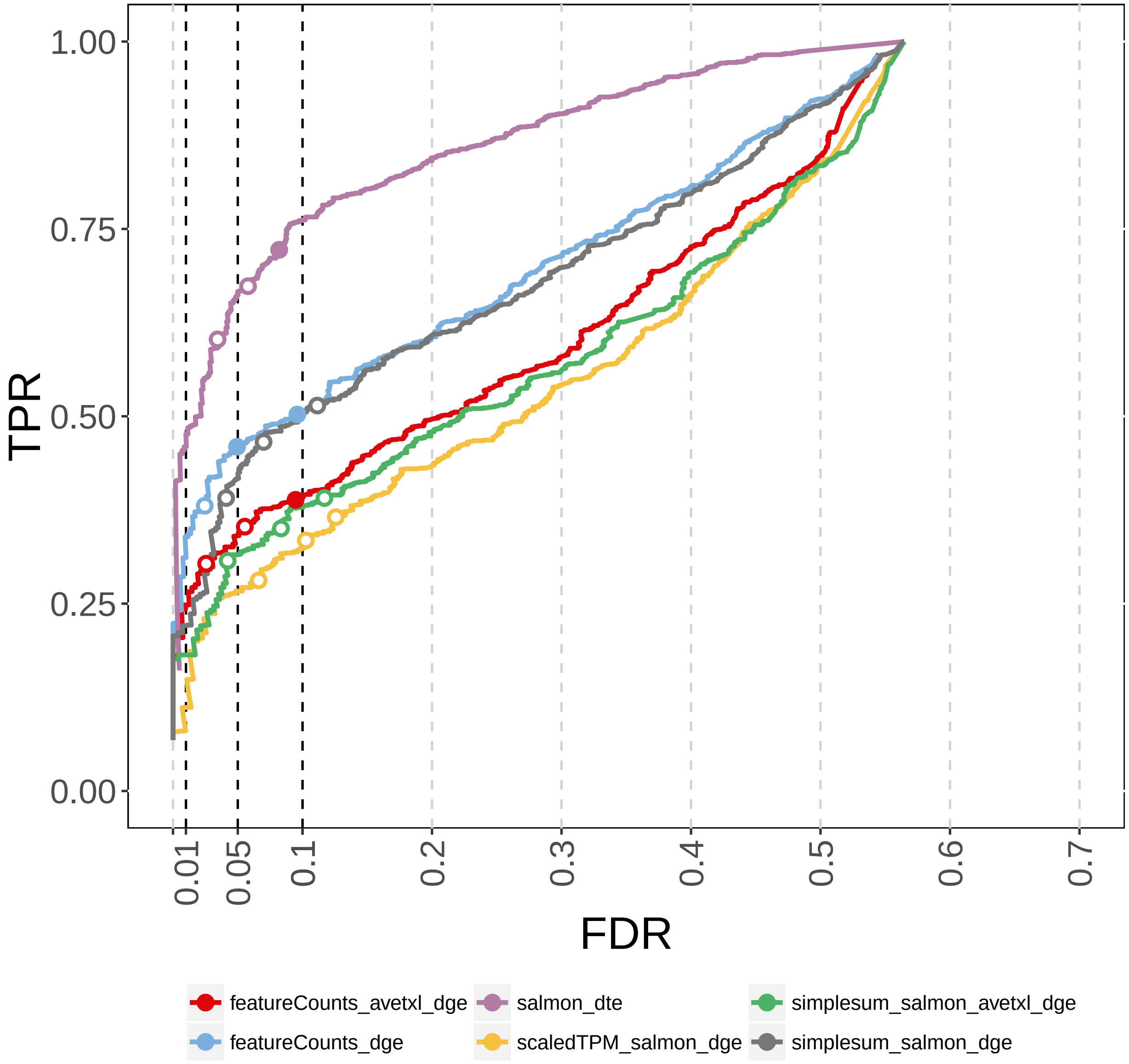

Supplementary Figure 9: Comparison of the ability of different approaches to detect DTE, using *edgeR* (**sim2**). Thus, here, a gene is considered truly differential if any of its corresponding transcripts is truly differentially expressed between conditions. Gene-level quantification followed by differential analysis (i.e., DGE approaches) is less powerful than transcript-level quantification and statistical testing aggregated on gene-level (i.e., DTE approach). This highlights the importance of appropriate specification of the question of interest before starting an analysis. As in the main text, DGE is performed on three gene-level count matrices: one generated by featureCounts, one obtained by scaling gene-level aggregated TPM estimates (scaledTPM_salmon) and one obtained by summing transcript-level counts on the gene level (simplesum_salmon). Moreover, for the featureCounts and simplesum_salmon matrices, DGE is performed using a standard pipeline as well as including offsets calculated from average transcript lengths (avetxl). The DTE analysis was performed on the transcript-level counts obtained by Salmon, and p-values were then aggregated on the gene level.

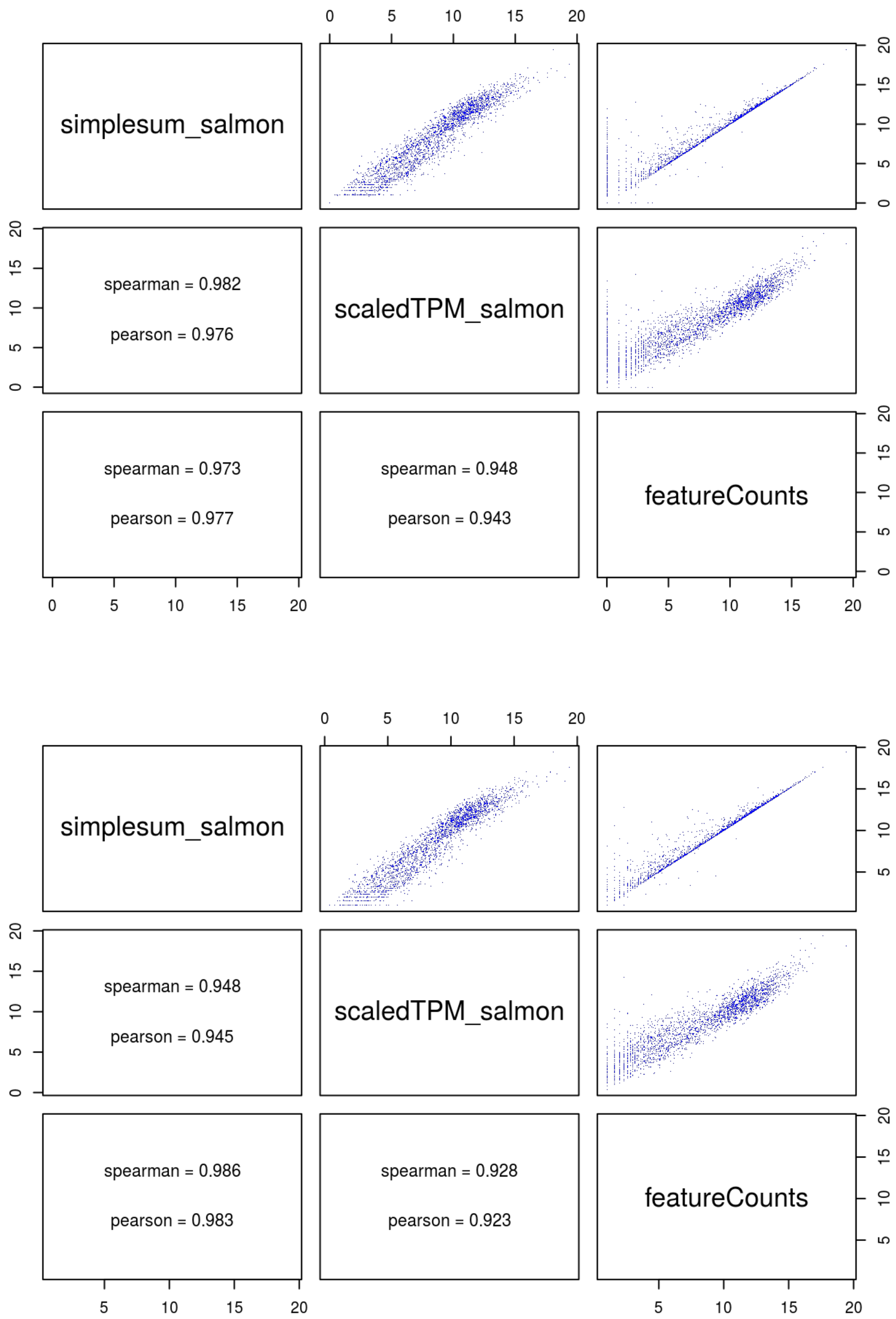

Supplementary Figure 10: Correlation among counts obtained with different approaches in one of the simulated samples (sampleA1) from the **sim2** data set. Top: all genes. Bottom: only genes with non-zero counts with all methods. For visualization and correlation calculations, the counts were log2-transformed after adding a pseudo-count of 1.

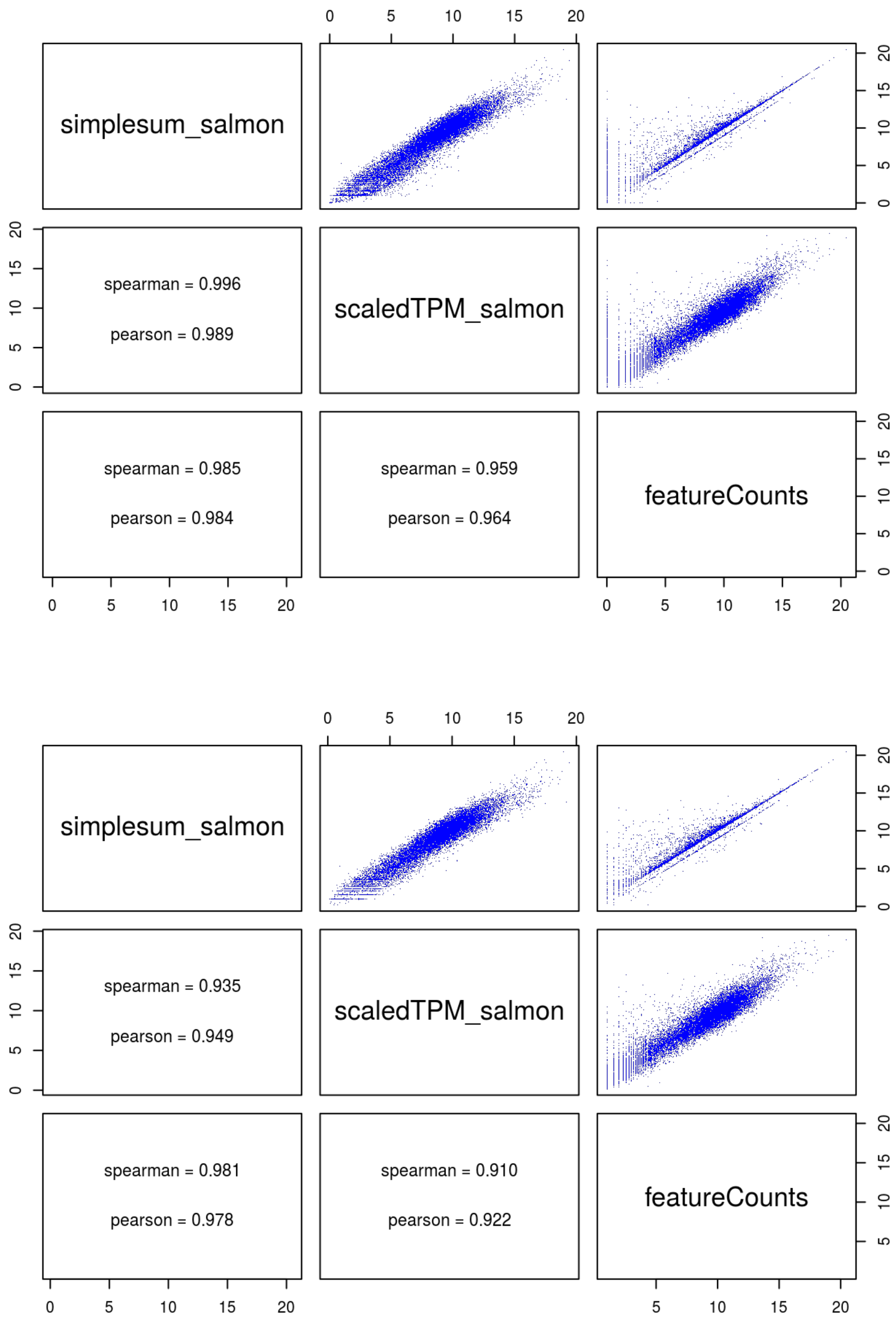

Supplementary Figure 11: Correlation among counts obtained with different approaches in one of the simulated samples (sample1) from the **sim1** data set. Top: all genes. Bottom: only genes with non-zero counts with all methods. For visualization and correlation calculations, the counts were log2-transformed after adding a pseudo-count of 1.

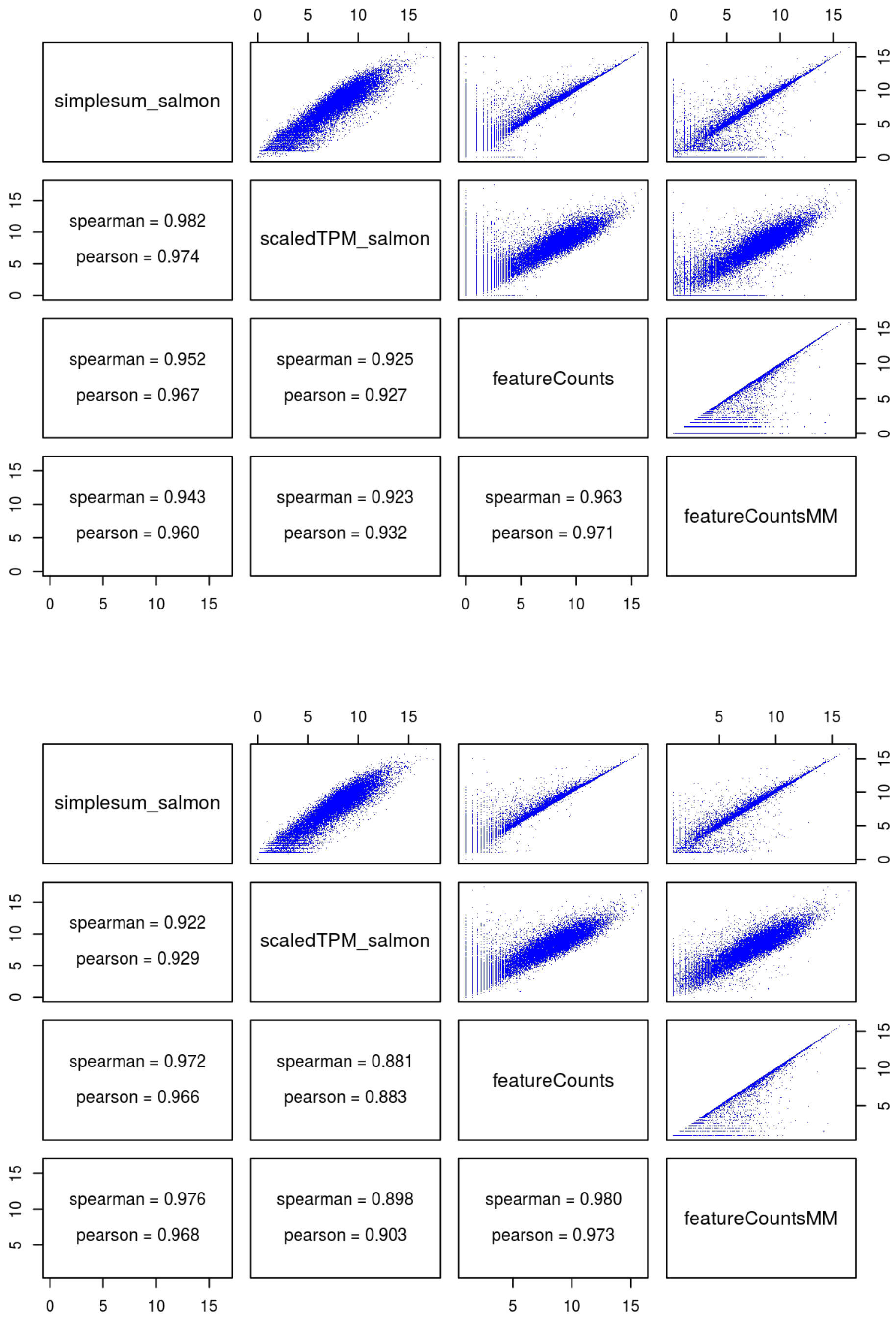

Supplementary Figure 12: Correlation among counts obtained with different approaches in the SRR099223 sample from the **Bottomly** data set. Top: all genes. Bottom: only genes with non-zero counts with all methods. For visualization and correlation calculations, the counts were log2-transformed after adding a pseudo-count of 1. **featureCountsMM** is similar to **featureCounts**, but includes multi-mapping reads by distributing them evenly between the mapping locations.

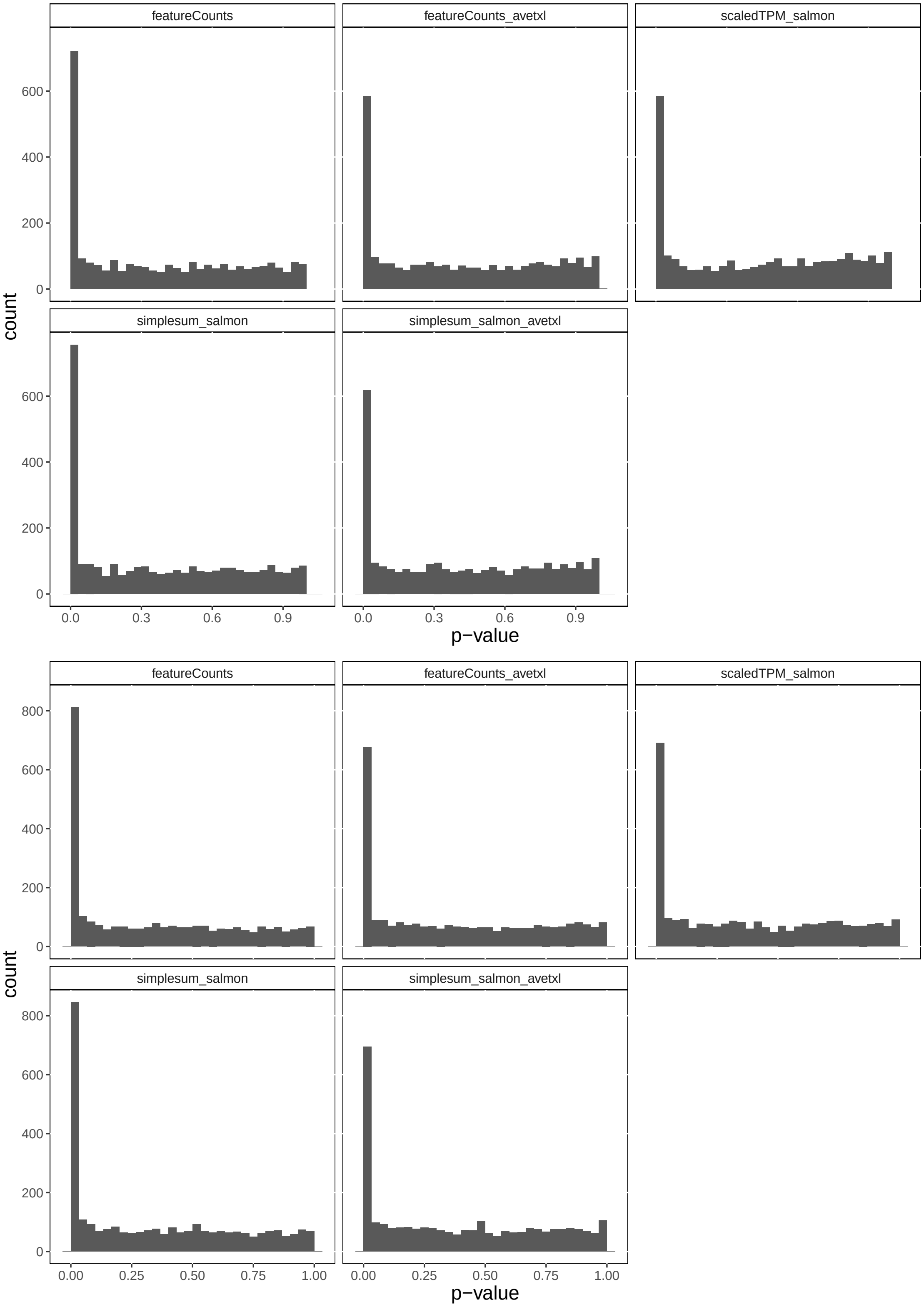

Supplementary Figure 13: Distribution of nominal p-values obtained by performing DGE using *edgeR* (top) and *DESeq2* (bottom) on gene-level count matrices obtained with different approaches (**sim2**).

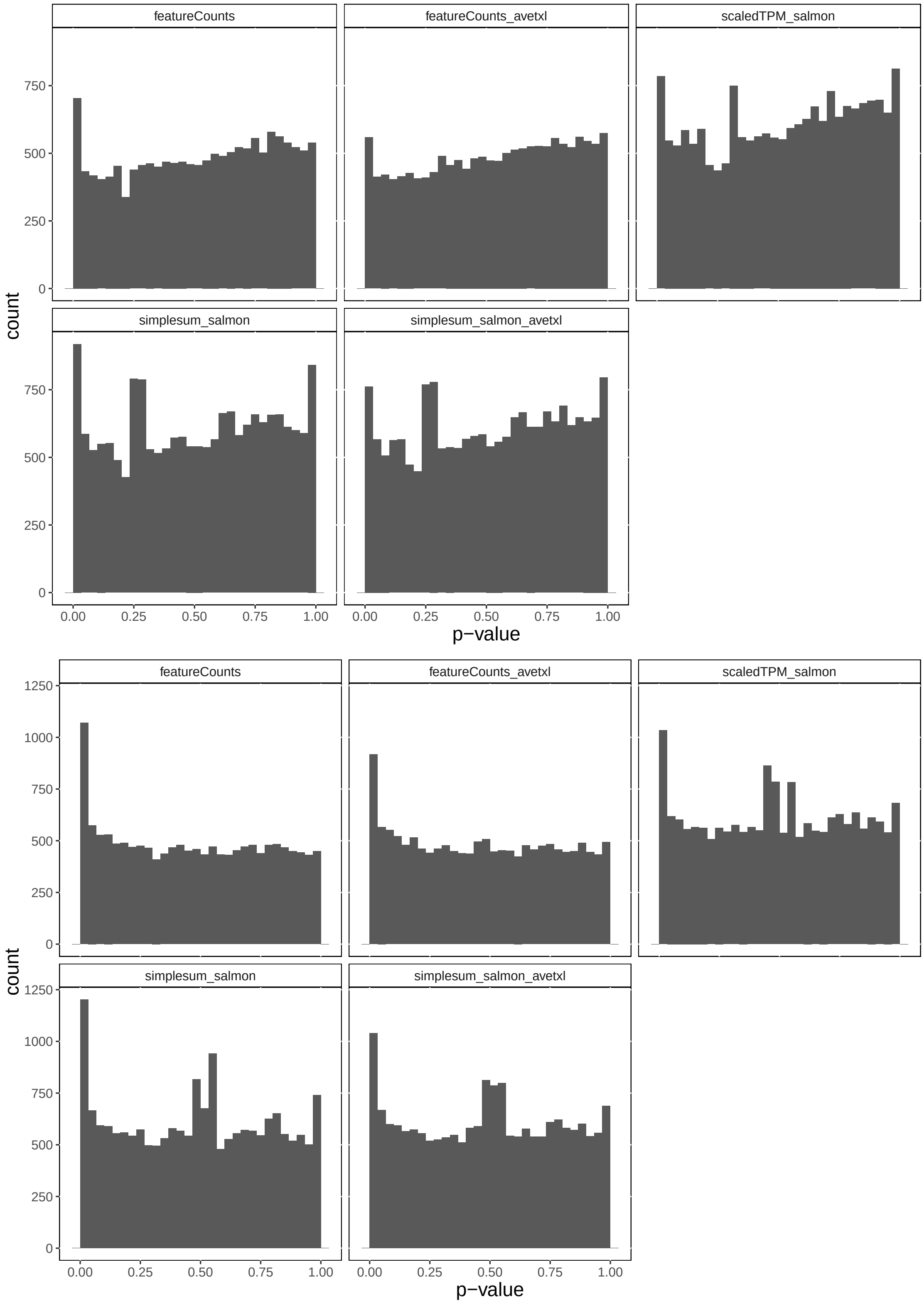

Supplementary Figure 14: Distribution of nominal p-values obtained by performing DGE using *edgeR* (top) and *DESeq2* (bottom) on gene-level count matrices obtained with different approaches (**sim1**).

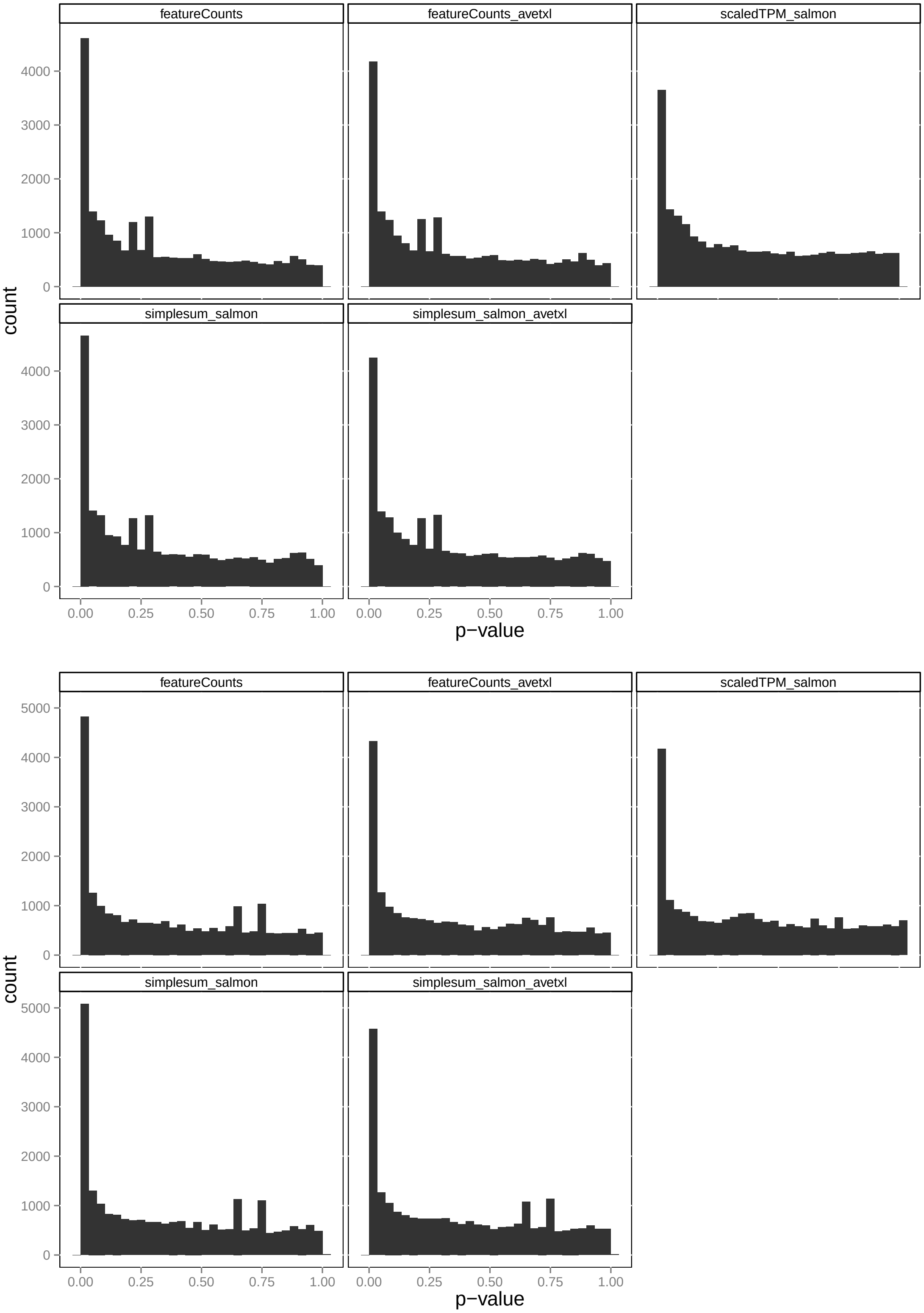

Supplementary Figure 15: Distribution of nominal p-values obtained by performing DGE using *edgeR* (top) and *DESeq2* (bottom) on gene-level count matrices obtained with different approaches (**Bottomly**).

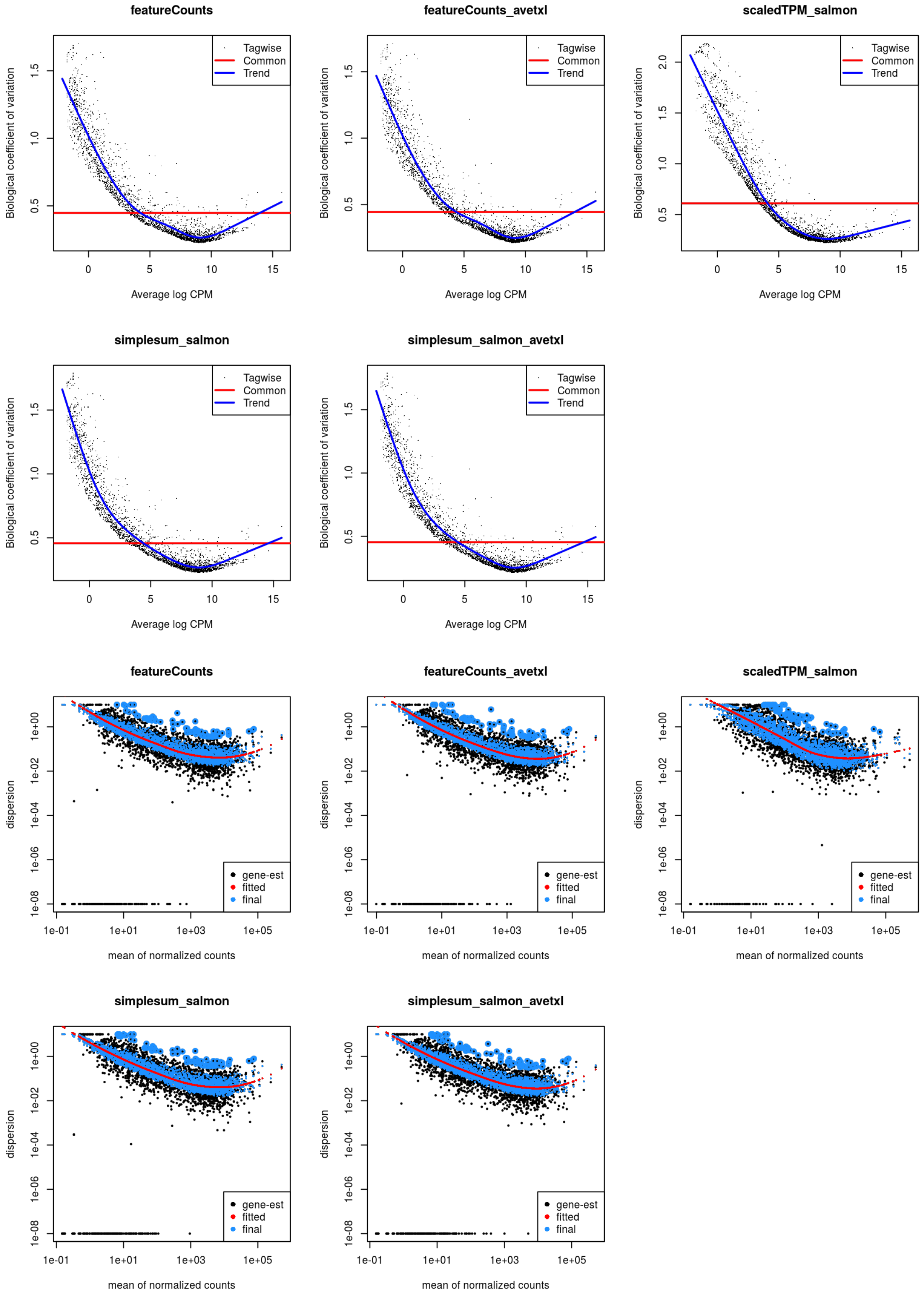

Supplementary Figure 16: Mean-dispersion relationship obtained from *edgeR* (top) and *DESeq2* (bottom) applied to gene-level count matrices obtained with different approaches (**sim2**).

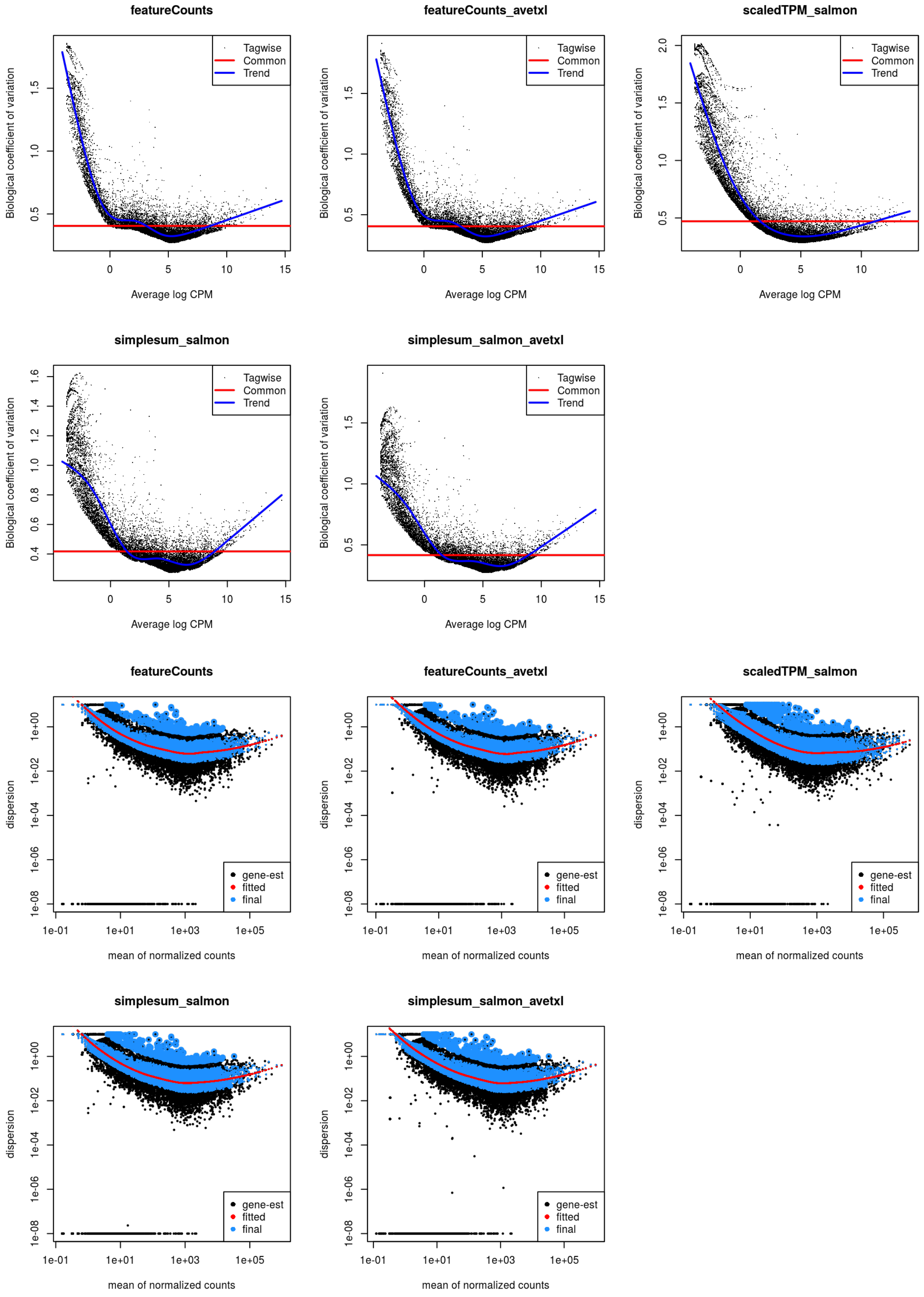

Supplementary Figure 17: Mean-dispersion relationship obtained from *edgeR* (top) and *DESeq2* (bottom) applied to gene-level count matrices obtained with different approaches (**sim1**).

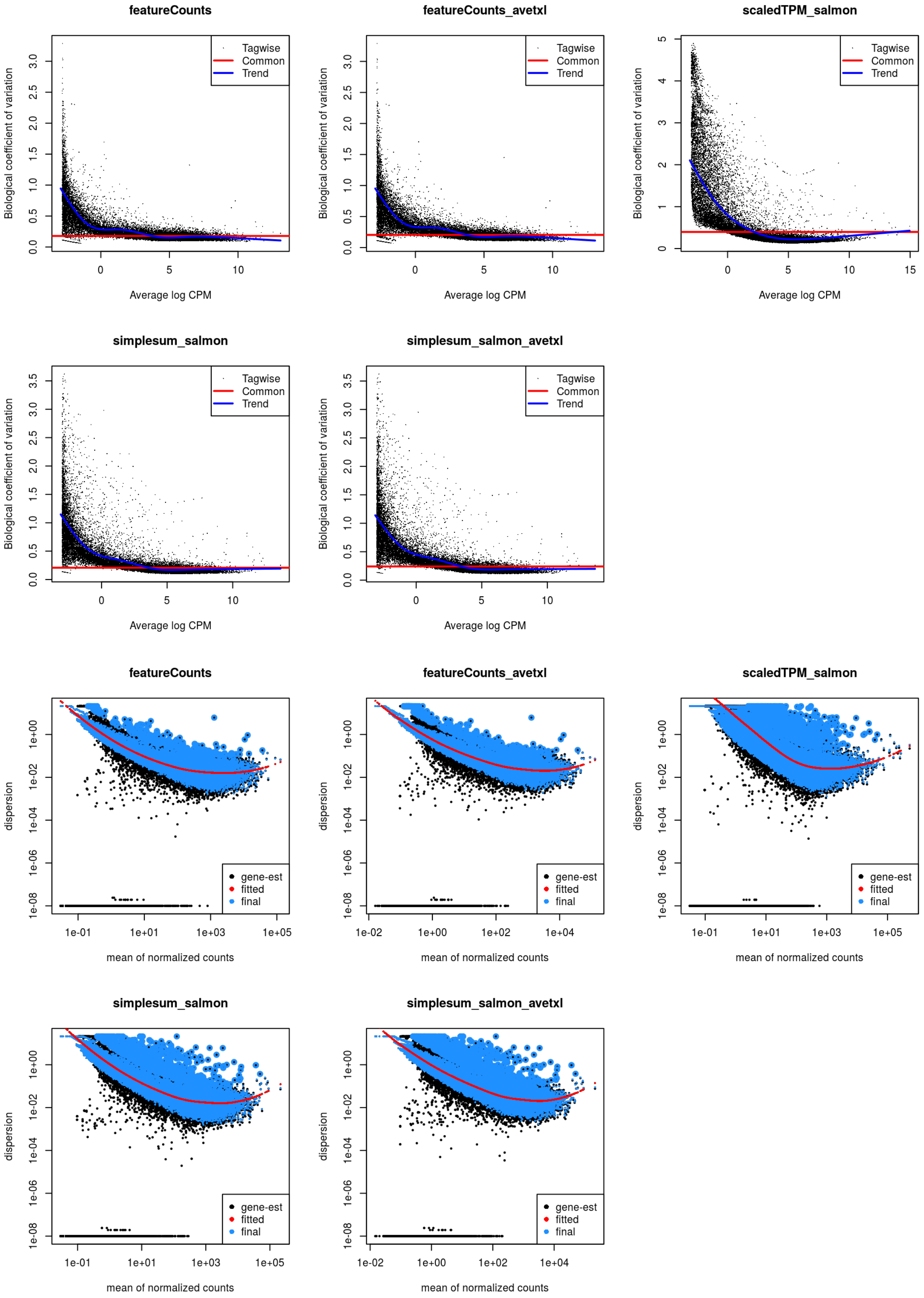

Supplementary Figure 18: Mean-dispersion relationship obtained from *edgeR* (top) and *DESeq2* (bottom) applied to gene-level count matrices obtained with different approaches (**Bottomly**).

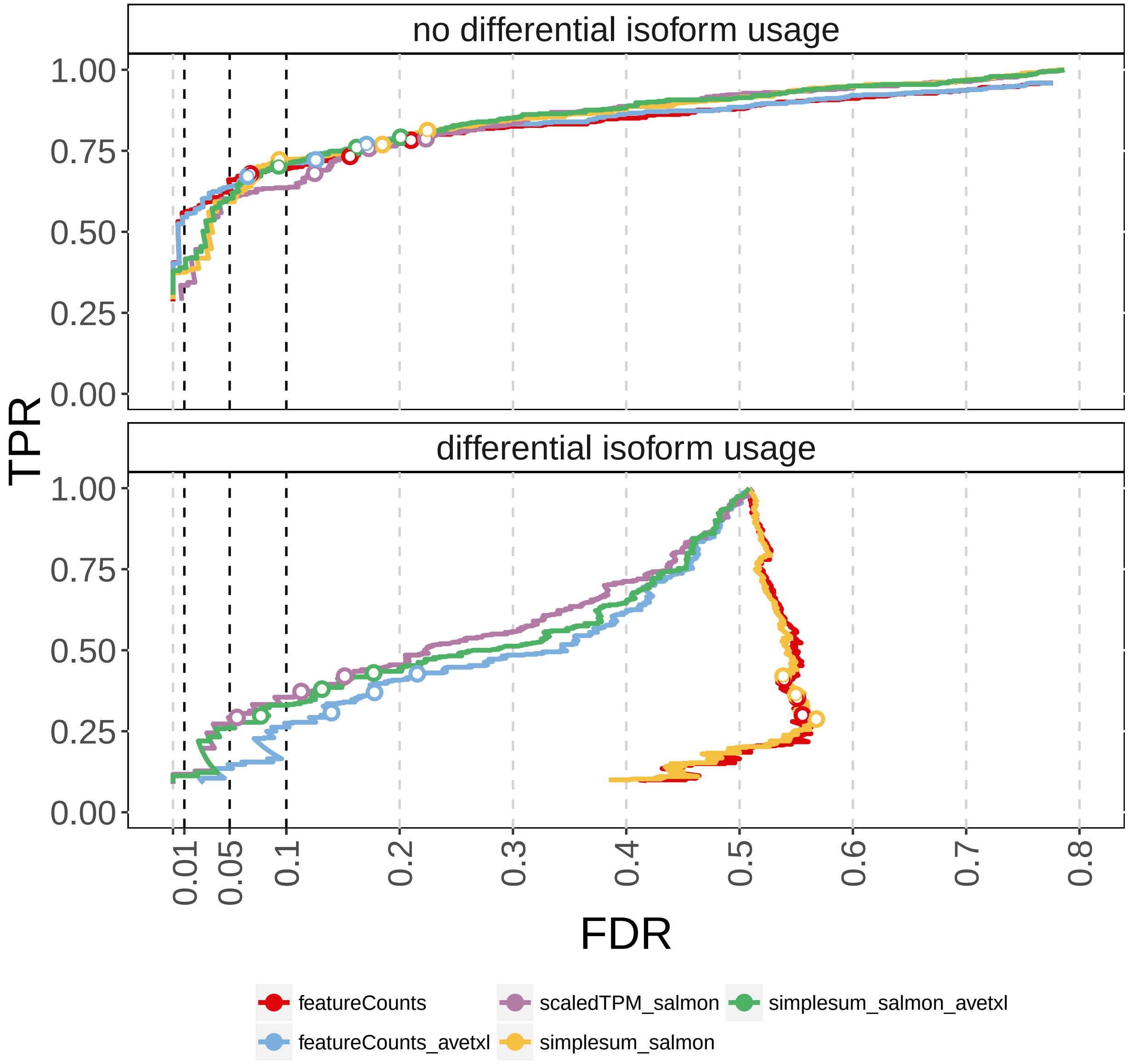

Supplementary Figure 19: Stratification of differential gene expression detection performance by the presence of differential isoform usage, using *DESeq2* (**sim2**).

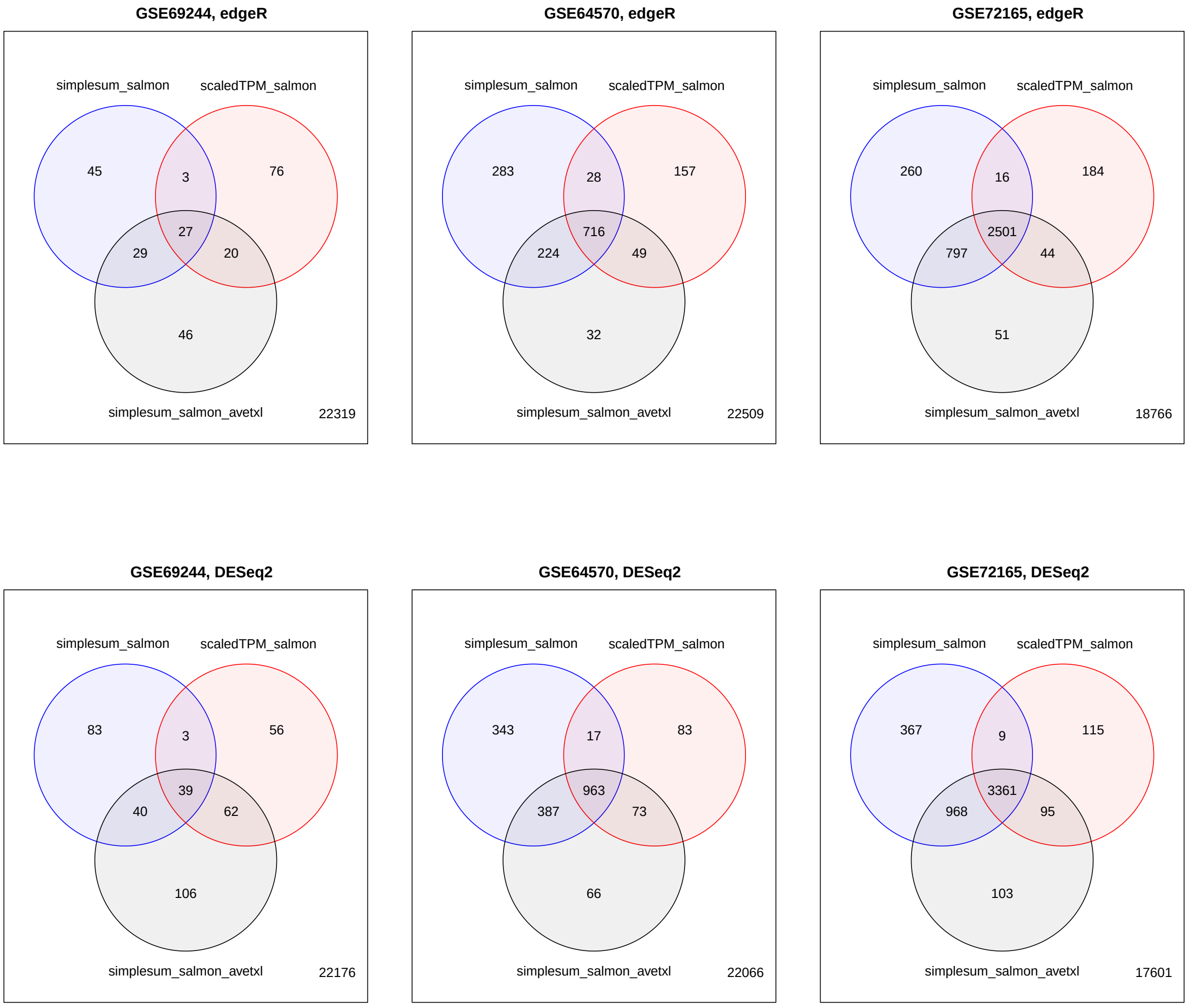

Supplementary Figure 20: Overlaps among collections of significant genes found with *edgeR* (top) and *DESeq2* (bottom) for three different experimental RNA-seq data sets, using three different counting pipelines. In these data sets, the majority of the significant genes are found with all counting methods, suggesting that for many real data sets, simple gene counting produces overall similar results as methods accounting for transcript-level expression.

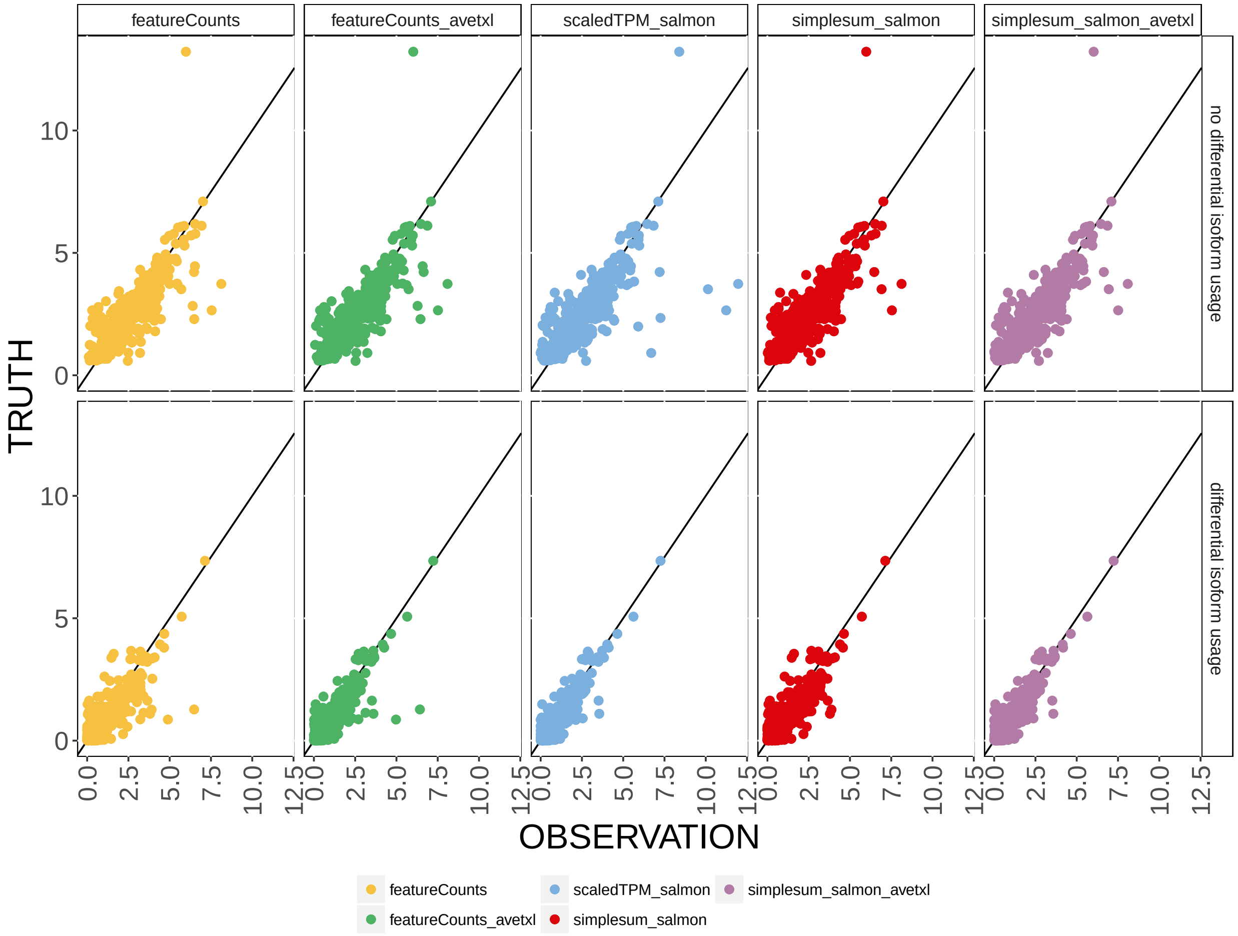

Supplementary Figure 21: Accuracy of overall gene log-fold change estimates based on results from *edgeR*, using different count matrices (**sim2**). Only genes with non-zero true log-fold changes are considered.

Supplementary Table 1: Spearman correlations quantifying the accuracy of the overall gene log-fold change estimates based on different count matrices (**sim2**), shown in Supplementary Figure 21.

	featureCounts	featureCounts_avetxl	scaledTPM_salmon	simplesum_salmon	simplesum_salmon_avetxl
No DTU	0.834	0.839	0.816	0.823	0.828
DTU	0.725	0.788	0.796	0.735	0.783

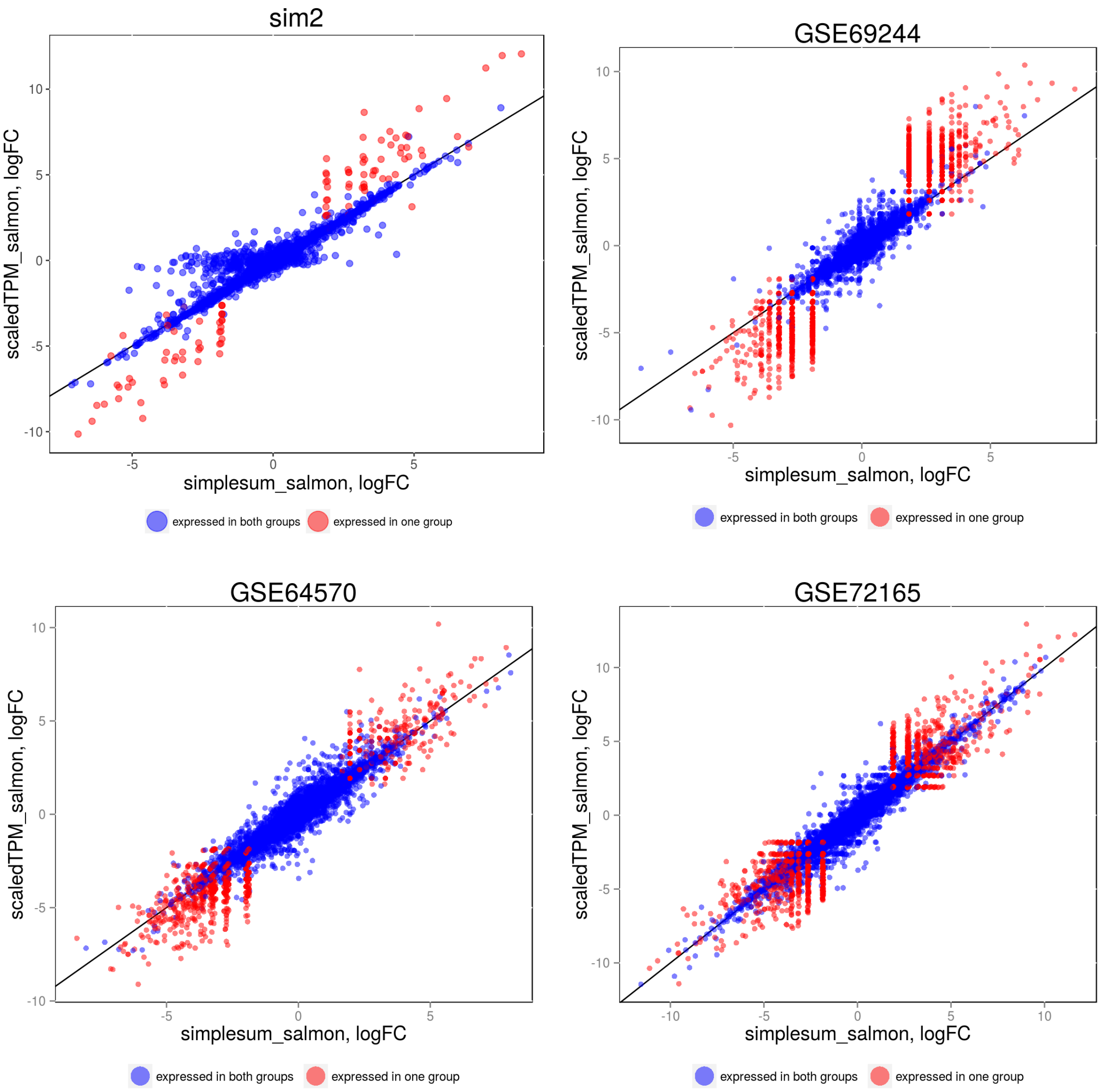

Supplementary Figure 22: Correlations between estimated log2-fold changes obtained with the **scaledTPM** and **simplesum** count matrices, using *edgeR*. The color indicates whether or not the gene is expressed in only one condition.

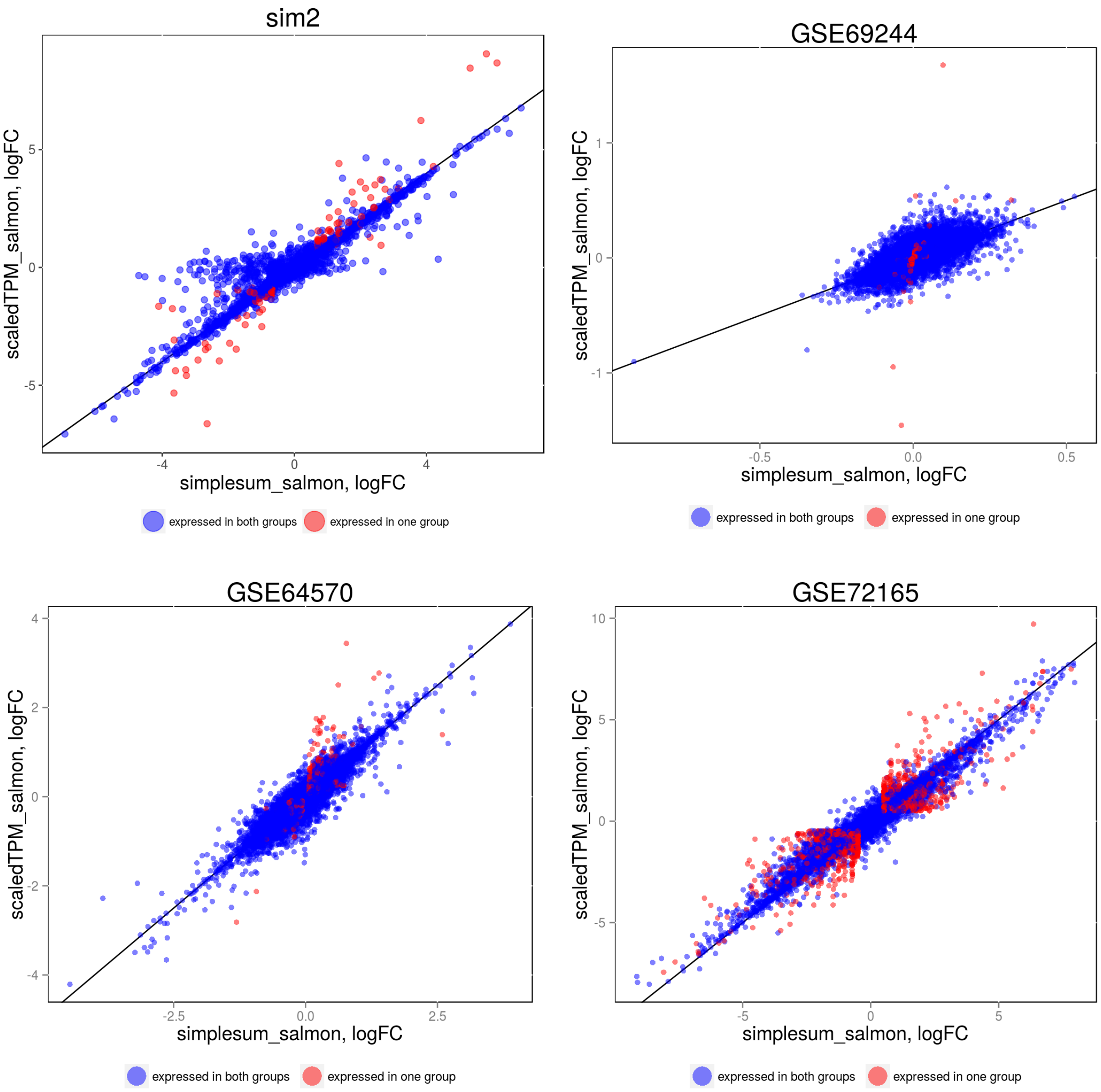

Supplementary Figure 23: Correlations between estimated log2-fold changes obtained with the **scaledTPM** and **simplesum** count matrices, using *DESeq2*. The color indicates whether or not the gene is expressed in only one condition.

References

[1] Daniel Bottomly, Nicole A R Walter, Jessica Ezzell Hunter, Priscila Darakjian, Sunita Kawane, Kari J Buck, Robert P Searles, Michael Mooney, Shannon K McWeeney, and Robert Hitzemann. Evaluating gene expression in C57BL/6J and DBA/2J mouse striatum using RNA-Seq and microarrays. *PloS one*, 6(3):e17820, 2011.

[2] Nicolas Bray, Harold Pimentel, Páll Melsted, and Lior Pachter. Near-optimal RNA-Seq quantification. *arXiv preprint 1505.02710*, may 2015.

[3] Andy J Chang, Fabian E Ortega, Johannes Riegler, Daniel V Madison, and Mark A Krasnow. Oxygen regulation of breathing through an olfactory receptor activated by lactate. *Nature*, 527(7577):240–244, nov 2015.

[4] Antonio Currais, Joshua Goldberg, Catherine Farrokhi, Max Chang, Marguerite Prior, Richard Dargusch, Daniel Daugherty, Aaron Armando, Oswald Quehenberger, Pamela Maher, and David Schubert. A comprehensive multiomics approach toward understanding the relationship between aging and dementia. *Aging*, nov 2015.

[5] Alexander Dobin, Carrie a Davis, Felix Schlesinger, Jorg Drenkow, Chris Zaleski, Sonali Jha, Philippe Batut, Mark Chaisson, and Thomas R Gingeras. STAR: ultrafast universal RNA-seq aligner. *Bioinformatics (Oxford, England)*, 29(1):15–21, jan 2013.

[6] Bo Li and Colin N Dewey. RSEM: accurate transcript quantification from RNA-Seq data with or without a reference genome. *BMC Bioinformatics*, 12(1):323, 2011.

[7] Yang Liao, Gordon K Smyth, and Wei Shi. featureCounts: an efficient general purpose program for assigning sequence reads to genomic features. *Bioinformatics*, 30(7):923–30, apr 2014.

[8] Rob Patro, Geet Duggal, and Carl Kingsford. Accurate, fast, and model-aware transcript expression quantification with Salmon. *bioRxiv*, oct 2015.

[9] Charlotte Soneson and Mauro Delorenzi. A comparison of methods for differential expression analysis of RNA-seq data. *BMC Bioinformatics*, 14(1):91, 2013.

[10] C Trapnell, D G Hendrickson, M Sauvageau, L Goff, J L Rinn, and L Pachter. Differential analysis of gene regulation by HOXA1 at isoform resolution with RNA-seq (supplemental material). *Nature Biotechnology*, 2013.

[11] Shuxin Yang, Rubén Marín-Juez, Annemarie H Meijer, and Herman P Spaink. Common and specific downstream signaling targets controlled by Tlr2 and Tlr5 innate immune signaling in zebrafish. *BMC genomics*, 16(1):547, jan 2015.
