# Supplementary material for: Differential analyses for RNA-seq: transcript-level estimates improve gene-level inferences
Source: F1000Res. 2016 Feb 29;4:1521. Originally published 2015 Dec 30. [Version 2] doi: 10.12688/f1000research.7563.2 (PMC4712774; doi:10.12688/f1000research.7563.2)
Supplement: Peer review history

# Peer review: Referee response for version 1

**DOI:** 10.5256/f1000research.8143.r11745

**Published:** 2016-01-12

**Authors:** Rob Patro

**Affiliations:** 1 Department of Computer Science, Stony Brook University, Stony Brook, NY, USA

In this manuscript, the authors address a few questions of considerable (and perennial) interest in the analysis of RNA-seq data. Specifically, they provide evidence that, using available methods (e.g. DESeq2 / edgeR), assessing differential expression at the gene-level (DGE) is more robust than at the transcript level (DTE). Further, they convincingly argue that estimating abundance at the level of transcripts, and then aggregating these abundances to the gene level leads to improved estimation of differential gene expression. They demonstrate that one of the major factors in this improved estimation is the availability of a sample-specific feature length for each gene (derived from the abundance-weighted length of the expressed transcripts of this gene), which is not possible to obtain with any fixed gene model used by count-based methods. Finally, the authors argue that much of the analysis of interest at the transcript level does not actually require differential transcript expression testing, but rather can be inferred from a combination of DGE and differential transcript usage (DTU); this is an interesting proposition that merits further discussion and analysis. Overall, this is a well-written paper, with extensive and compelling supplementary and supporting data, that addresses a ubiquitous analysis task involving RNA-seq.  It should be of broad interest to the community and makes a valuable contribution. The accompanying software, *tximport*, is user-friendly and makes it easy to apply the type of analysis recommended herein; it too should be widely useful.

**Major comments:**

It would be very useful to provide the equations used for calculating each of the abundance measured considered directly. Section 4 of the supplementary information is useful to this end, but the reader still has to search a bit to see exactly how each metric is computed (though the fantastic R-Markdown included with the figures means that these computations can be found explicitly).

Similarly, it would be useful to the reader to provide a description, in prose, of how specific experiments were performed (again, the reproducible nature of most of these experiments makes tracking down this information possible, but sometimes time-consuming). For example, how, precisely, was removal of transcripts handled at the level of the genome annotation? If a transcript consists only of constitutive exons, were all of those exons retained in the genome annotation used for STAR + featureCounts, while the transcript was removed in the Salmon index?  

The result that transcript-level abundance estimation is more sensitive to the removal of transcripts than gene-level abundance estimation — this seems intuitive. However, I agree with Dr. Floor’s suggestion that:

*"The assertion that “simple counts tended to show a high degree of robustness against incompleteness of the annotation catalog, as evidenced from estimation errors after first removing (at random) 20% of the transcripts” seems misleading since Salmon-derived gene-level abundances actually show a higher Spearman correlation than count-derived gene-level abundances when subjected to removing a random 20% of transcripts.”*

I would suggest rewording this sentence, as the main result seems to be that gene-level analysis is more robust to an incomplete annotation than transcript-level analysis. Transcript-level abundance estimation followed by gene-level analysis seems to perform just as well (actually, better) than gene-level counting in this scenario.

The experiments in the section “Incorporating transcript-level estimates leads to more accurate DGE results” suggests the (reasonable) interpretation that the main benefit of incorporating transcript-level abundance estimates when assessing DGE is a more accurate measure of the “feature” length of the gene. The authors state “ It is important to note that this improvement is entirely attributable to an improved handling of genes with changes in isoform composition between the conditions.” This is supported by the fact that using the abundance-weighted average transcript length (i.e. offsets) with counting based approaches improves the results substantially.  However, one other place where transcript-level abundance estimates are useful in the context of DGE is when assessing the expression of paralogous genes. While most multi-mapping reads that derive from different isoforms of the same gene will be uniquely mappable at the level of the genome, and hence will be included in the counts for that gene, reads that map ambiguously among paralogs may not be. In such cases, count-based methods do not have a principled way of apportioning a read between the paralogs involved, and discarding multi-mapping reads may negatively affect estimation of the abundance of the paralogs, even at the gene level. While this case is likely much less common than mis-estimation of DGE as a result of DTU, it is certainly of biological interest. I would suggest adding an analysis, restricted to sets of paralogous genes, comparing how the different approaches perform in this case. This may help to highlight the importance of not only deriving appropriately weighted and sample-dependent lengths for genes, but also on resolving multi-mapping ambiguity that occurs between genomically distinct loci.

The argument that most transcript-level analyses of interest may be addressed by looking at DGE in conjunction with DTU is an interesting one. It is certainly that case that not all tasks for which DTE is used actually require assessing differential expression at the transcript level. One issue with the DGE + DTU-based analysis which warrants further discussion in the manuscript is that I believe that this approach, too, would require correcting for multiple hypothesis testing. Specifically, one is testing both the DGE and the DTU hypotheses for each gene (or for a relevant subset of interest). The correction here is likely to be less harsh than in the case of assessing DTE, but is still worth discussing.

Minor comments:

As per Dr. Floor’s statement, Salmon (and Sailfish) also incorporate sequence-specific bias correction. Further, RSEM and Salmon (and a few other transcript-level abundance estimation tools) also incorporate the modeling of non-uniform fragment start position distributions. Of course, modeling a non-uniform start position distribution cannot overcome a complete lack of sampling in critical regions that might make determining transcript-level fragment assignment impossible, but it may help in properly apportioning an ambiguously-mapped fragment between transcripts depending on its relative position in each.

One potential added source of variability here is that all Salmon estimates presented in the manuscript make use of Salmon’s quasi-mapping of reads, while the STAR + featureCount pipeline makes use of “traditional” alignments. This is the primary intended usage mode of Salmon, and absolutely does represent a “typical” pipeline for methods that avoid alignment (Salmon, Sailfish, kallisto). However, it would probably be best to mention this as a potential (though likely negligible) additional source of variability.

In the discussion, the authors argue that “... it is therefore imperative to continue improving abundance estimation and inference methods applicable to individual transcripts, since misestimation can propagate to the gene level.” This is, of course, an important and valid suggestion. Another direction, on which it would be useful to get the authors’ thoughts and suggestions, is the development of differential expression tools (at either the transcript or gene level) that can make use of the variance estimates that some tools (like Salmon) can provide. To what extent might incorporating this information help control false positive rates and improve DTE or even DGE estimates?

I have read this submission. I believe that I have an appropriate level of expertise to confirm that it is of an acceptable scientific standard.

# Response

**Published:** 2016-02-19

**Authors:** Mark Robinson

**Affiliations:** University of Zurich, Switzerland

Thank you for taking the time to read and review our paper. As per your suggestions, we have expanded the supplementary pdf document with equations defining each of the abundance measures that we used as well as a detailed description of the generation of the incomplete annotation files. We have also reworded the paragraph discussing the influence of an incomplete annotation catalog, and added further discussion points regarding multiple testing correction for DGE+DTU as well as regarding new methods incorporating variance estimates into the differential analysis. However, a deeper discussion of the latter point falls outside the scope of this article.

We found your suggestion to restrict the abundance accuracy comparison to paralogous genes interesting, and the supplement has been expanded with several examples comparing the accuracy of gene-level abundance estimates from Salmon and from STAR+featureCounts, restricted to sets of paralogous genes. While we see a clear advantage of Salmon for many of the paralogous gene groups, the overall difference is only slightly larger for sets of paralogous genes compared to random sets of genes (as can be seen in the revised version of Dataset 1). In addition, we have added the following text to the relevant section in the results:

“...suggesting that the extent of the problem in many real data sets is limited... though some loss of sensitivity for certain genes may be encountered from discarding multi-mapping fragments, which may be recovered through the use of transcript abundance estimators such as Salmon.”

While we acknowledge that many of the popular transcript abundance estimation methods incorporate some type of bias estimation/correction (albeit not at the fragment level), and that it is definitely an important (and difficult) research area, a thorough discussion on the relative merits of different bias correction approaches is outside the scope of this paper. We have added citations to relevant literature on coverage bias, have simplified and clarified the last paragraph of the discussion, and have reworded the sentence about unidentifiable estimation due to coverage biases:

“While some extent of coverage variability might be alleviated by corrections for sequence- or position-specific biases, there remain cases where transcript expression cannot be inferred from data ( Figure 1C)”

# Peer review: Referee response for version 1

**DOI:** 10.5256/f1000research.8143.r11761

**Published:** 2016-01-04

**Authors:** Stephen N. Floor

**Affiliations:** 1 Department of Molecular and Cell Biology, University of California, Berkeley, Berkeley, CA, USA

Soneson, Love and Robinson tackle a crucial question for analysis of RNA deep sequencing data in this manuscript: what is the role of transcript diversity in the accuracy and statistical power associated with measurements of gene expression? The authors make and convincingly show three claims: gene-level estimation and inferences are more robust than those at the transcript-level, and incorporating transcript-level quantification into gene-level abundance leads to improved differential expression testing.  The claims are convincingly proven, the manuscript is well written, and the subject matter is of considerable interest. Furthermore, the described R package *tximport *should be of broad interest to the RNA deep sequencing community.

Overall comments:

It may be useful to indicate explicitly in the text that the methods are contained within the (excellently written and formatted) supplementary material, as this was not apparent. It might be clearest to create a specific methods section that just references supplementary file 1.

The clarity of scatter plots with more than ~hundreds of points (e.g. Figure 1A) could be improved by using partially transparent points to visualize density.

Introduction:

Paragraph 1: Cufflinks, RSEM and Bitseq are grouped with kallisto and Salmon and it is then stated that some of these methods bypass read alignment. It would be clearer if this were reworded to avoid the ambiguity as to which methods avoid read alignment.

Paragraph 4: The third claim could be presented more clearly. While it is interesting that simple counting performs similarly to transcript-level quantification procedures, it seems more interesting to this reviewer that incorporating transcript-level information improves the accuracy of differential expression testing at the gene level. Perhaps these two concepts can be combined into one more concise point?

Results:

The assertion that “simple counts tended to show a high degree of robustness against incompleteness of the annotation catalog, as evidenced from estimation errors after first removing (at random) 20% of the transcripts” seems misleading since Salmon-derived gene-level abundances actually show a higher Spearman correlation than count-derived gene-level abundances when subjected to removing a random 20% of transcripts. Figure 1a bottom left shows that transcript-level abundances are strongly affected by removal of 20% of transcripts, but that gene-level abundances are not strongly changed whether estimated using counts or Salmon. This statement should be reworded.

Two concerns are raised about DTE. It is certainly true that reads are spread across more features when performing DTE as opposed to DGE.However, it is not apparent why analysis of DTE involves grouping of transcripts together for interpretation. DTE implies analysis at the transcript level and therefore no grouping, while DGE could involve some level of grouping of transcripts or quantification at the gene level from the start. The clarity of this could be improved.

It is a very interesting idea to separately frame questions regarding DGE and DTU, which should be adopted widely, as the two are separable questions.

The authors state one possible workflow towards DGE analysis in the section “Incorporating transcript-level estimates leads to more accurate DGE results.” Alternative pipelines (e.g. cuffdiff) could be presented in brief.

The observation that simplesum and featureCounts results are highly correlated and therefore that statistical methods based on the Negative Binomial distribution can be used on estimated counts seems of greater importance than is emphasized in the text. This should be elaborated upon in the discussion, since this means that estimated counts from kallisto, express, salmon, etc can be used directly by statistical packages assuming a NB distribution (edgeR, DESeq2, etc). This point is frequently debated in discussions of how to rigorously analyze sequencing data. The conclusion here that NB applies to estimated counts is thus quite important.

Please explain the meaning of the name for each curve in the legend for Figure 3 (i.e. specify that “avetxl” means using the offset corresponding to average transcript length.

Discussion:

The assertion that “gene-level results are more accurate, powerful and interpretable than transcript-level results” seems an oversimplification given the result that incorporating transcript-level quantification leads to improved DGE detection performance (e.g. Fig 3).

Please cite at minimum Roberts *et al.*, (2011) regarding sequence bias correction as this has been implemented in cufflinks, express and kallisto. Other relevant papers should also be included here, as attempts have been made to address both positional and sequence-specific bias in RNA sequencing data.

Supplement:

The usability of the supplemental info could be improved by substituting rasterized for vectorized plots for those with ~hundreds of points.

Please explain the meaning of the name for each curve in the legend for Supplemental Figure 5.

I have read this submission. I believe that I have an appropriate level of expertise to confirm that it is of an acceptable scientific standard.

## References

[1] RobertsATrapnellCDonagheyJRinnJLPachterL: Improving RNA-Seq expression estimates by correcting for fragment bias. *Genome Biol*.2011;12(3) : 10.1186/gb-2011-12-3-r22 R22 10.1186/gb-2011-12-3-r22 21410973PMC3129672

# Response

**Published:** 2016-02-19

**Authors:** Mark Robinson

**Affiliations:** University of Zurich, Switzerland

Thank you for taking the time to read and review our paper. In the revised version, we have improved the clarity and usability of the figures and the supplement by using partially transparent points and extending the figure captions. We have also modified the main text to refer more clearly to the method information provided in the supplementary material, and improved the clarity of the text in several places, according to your suggestions.

For additional comments regarding references to bias correction studies, please see responses to Rob Patro.
